# A New Ligand Design Based on London Dispersion Empowers Chiral Bismuth–Rhodium Paddlewheel Catalysts

**DOI:** 10.1021/jacs.1c01972

**Published:** 2021-04-08

**Authors:** Santanu Singha, Michael Buchsteiner, Giovanni Bistoni, Richard Goddard, Alois Fürstner

**Affiliations:** Max-Planck-Institut für Kohlenforschung, 45470 Mülheim/Ruhr, Germany

## Abstract

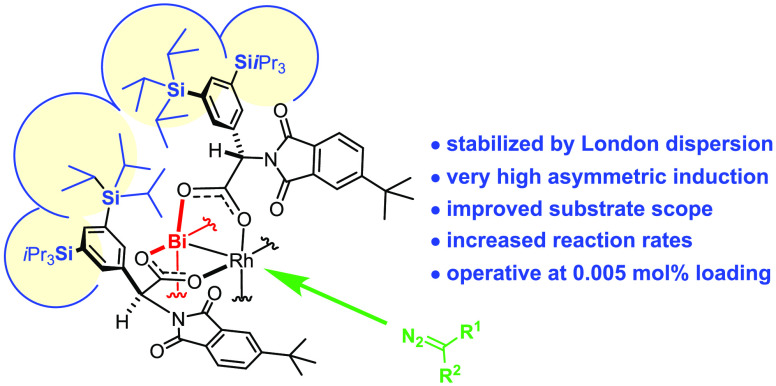

Heterobimetallic bismuth–rhodium paddlewheel complexes with phenylglycine ligands carrying TIPS-groups at the *meta*-positions of the aromatic ring exhibit outstanding levels of selectivity in reactions of donor/acceptor and donor/donor carbenes; at the same time, the reaction rates are much faster and the substrate scope is considerably wider than those of previous generations of chiral [BiRh] catalysts. As shown by a combined experimental, crystallographic, and computational study, the new catalysts draw their excellent application profile largely from the stabilization of the chiral ligand sphere by London dispersion (LD) interactions of the peripheral silyl substituents.

The first chiral dirhodium carbene characterized by X-ray crystallography was derived from diazoester **4a** and [Rh_2_(PTTL)_4_] (**1a**) (Figure 1).^1−4^ This particular paddlewheel complex had been chosen not only for its excellent pedigree in asymmetric catalysis^5−26^ but also because the reasons for its efficiency had been subject to debate.^27−33^ Interestingly, the four *N*-phthalimido substituents on the *tert*-leucine ligands were found to adopt an α,α,α,α-conformation about the carbene ligand occupying an axial site on the dirhodium core; this chiral calyx is maintained in solution.^1,27,28^ Under the premise that the enantiodetermining transition state features a similar overall structure, the data allowed the *sense* of induction in the cyclopropanation of styrene to be explained;^1^ the moderate *level* of induction (78% ee) can also be rationalized by the fairly wide aperture of the chiral binding site, as observed in the solid state. Moreover, the two Rh atoms reside in notably different ligand environments: the *tert*-butyl groups form a narrow but essentially “achiral” pore about Rh_2_: any competing background reaction at this site will reduce the optical purity of the product.^1^

**Figure 1:**
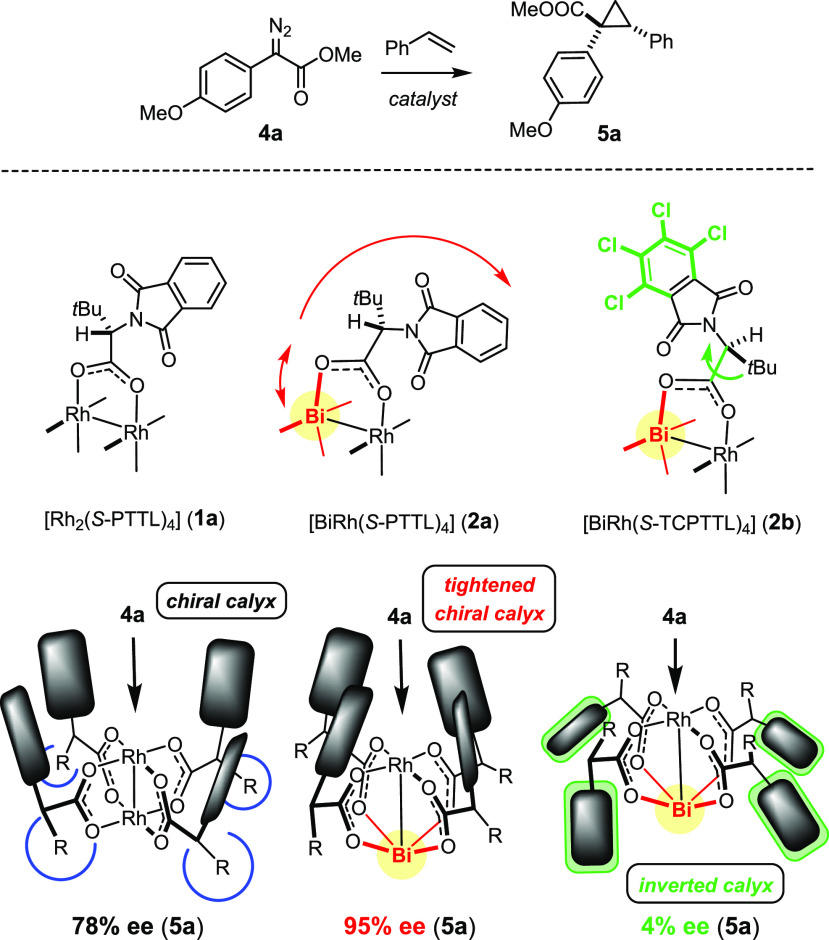
Prior art. The “paddles” in the schematic drawing represent the (substituted) phthalimido groups, R = *t*Bu.

In a first foray to translate these insights into an improved catalyst design, the Rh_2_ center of **1a** was formally replaced by Bi(+2).^34−46^ For its larger radius, this main group element imparts a conical shape onto the ligand sphere, which, in turn, tightens the chiral pocket at the Rh-site. Since Bi(+2) does not decompose the diazo ester, the racemic background reaction is essentially shut off. These effects are thought to synergize; they explain why the heterobimetallic complex [BiRh(PTTL)_4_] (**2a**) led to much improved ee’s in many cases.^34^ Limitations were encountered with less electron-rich donor/acceptor diazo derivatives, which gave rather poor results (see below).^34^

Initial attempts to remedy this issue by tailoring the phthalimido groups largely met with failure. Although perhalogenated phthalimides have proven advantageous in many cases,^47−50^ the heterobimetallic complex **2b** gave almost racemic product. The X-ray structure revealed the likely cause: **2b** features an *inverted* calyx, in which the “chiral” environment envelopes the unreactive Bi(+2) site, whereas the Rh-atom sees the *tert*-butyl substituents.^34^ The substituted phthalimido groups are more encumbered and hence flank the Bi(+2) center, simply because the larger cation provides more space.^34^

An improved design needs to provide rigorous control over the directionality of the chiral ligand sphere while allowing the substitution pattern to be fine-tuned. To this end, we planned to employ London dispersion as a noncovalent but attractive interaction to lock the desirable conformation in place. It is increasingly clear that multiple intramolecular contacts can outweigh steric repulsion and be structure-determining;^51,52^ although this effect might be more prevalent in (organometallic) catalysis than commonly appreciated,^51,53−56^ examples of deliberate use of London dispersion (LD) as a key design principle for the development of new catalysts with improved application profiles are exceedingly rare.^57−59^ Outlined below we present such a case.

To reach this goal, the *tert*-leucine-derived ligands of **2a**,**b**, which are too far apart for any LD among them,^34^ were replaced by phenylglycine derivatives carrying TIPS-groups at the *meta*-positions of the aromatic ring; the latter were expected to mediate a sufficient number of intramolecular contacts to entail stabilization via dispersion interactions (Scheme 1).^60−62^ Commercial benzyl alcohol **6** was O-silylated prior to metal/halogen exchange and quenching of the dilithio species with TIPSCl. Product **7** was elaborated into *tert*-butylsulfinyl imine **8**,^63^ which reacted with vinylmagnesium bromide in the presence of ZnMe_2_ to give **9** as a single isomer (dr ≈ 99:1). The auxiliary was removed and the phthalimide of choice^64^ introduced before the double bond of **10** was cleaved with RuCl_3_ cat./NaIO_4_ to avoid racemization. The resulting acids **11**(65) were reacted with [BiRh(OCOCF_3_)_4_] in boiling toluene and the released trifluoroacetic acid trapped with K_2_CO_3_ in a Soxhlet apparatus to furnish complexes **3** in excellent yield.

**Scheme 1:**
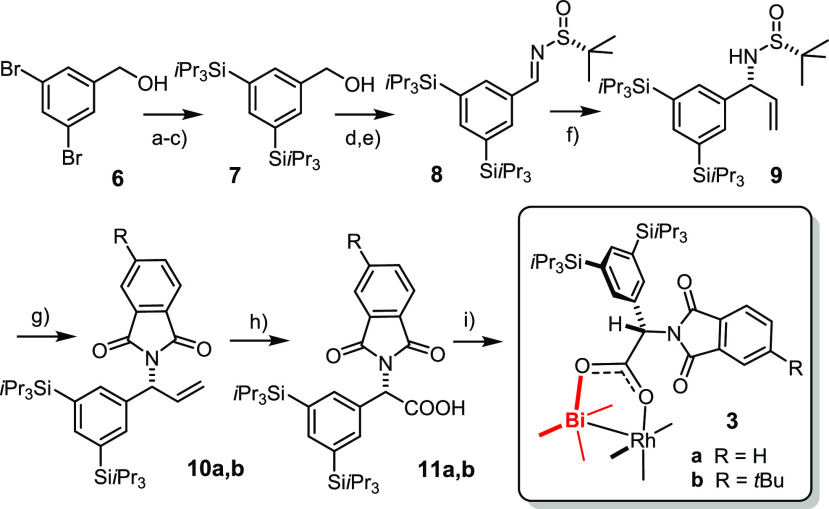
Reagents and conditions: (a) TIPSCl, DBU, CH_2_Cl_2_, 99%; (b) *t*BuLi, THF, −78 °C → rt, then TIPSCl, −20 °C → rt; (c) TBAF, THF, −20 °C → rt, 74% (over both steps); (d) PCC, CH_2_Cl_2_, 94%; (e) (*R*)-*t*BuS(=O)NH_2_, Ti(OEt)_4_, THF, 70 °C, 84%; (f) vinylmagnesium bromide, Me_2_Zn (50 mol %), THF, −78 °C, 87% (dr = 99:1); (g) (i) HCl/1,4-dioxane, MeOH; (ii) aq. NaOH, 90% (iii) (substituted) phthalic anhydride, Et_3_N, toluene, reflux, 75% (**10a**), 73% (**10b**); (h) RuCl_3_·H_2_O (5 mol %), NaIO_4_, CCl_4_, MeCN, H_2_O, 74% (**11a**, 96% ee), 69% (**11b**, 98% ee); (i) [BiRh(OCOCF_3_)_4_], toluene, reflux, 81% (**3a**), 94% (**3b**).

Although **3a** crystallizes well, the many degrees of rotational freedom of the eight peripheral TIPS groups cause disorder. This complication notwithstanding, a data set meeting good crystallographic standards was obtained (see the Supporting Information (SI)). As expected, complex **3a** adopts an α,α,α,α-conformation, in which the silylated aryl rings envelope the Bi-center whereas the phthalimides form a narrow chiral pocket about Rh (Figure 2). The steric demand of the TIPS groups is manifested in innumerous short H···H and H···C contacts in the periphery. The crystallographic data, however, do not allow us to decide whether these contacts mediate LD and are hence stabilizing, or whether they are repulsive as a consequence of steric hindrance.

**Figure 2:**
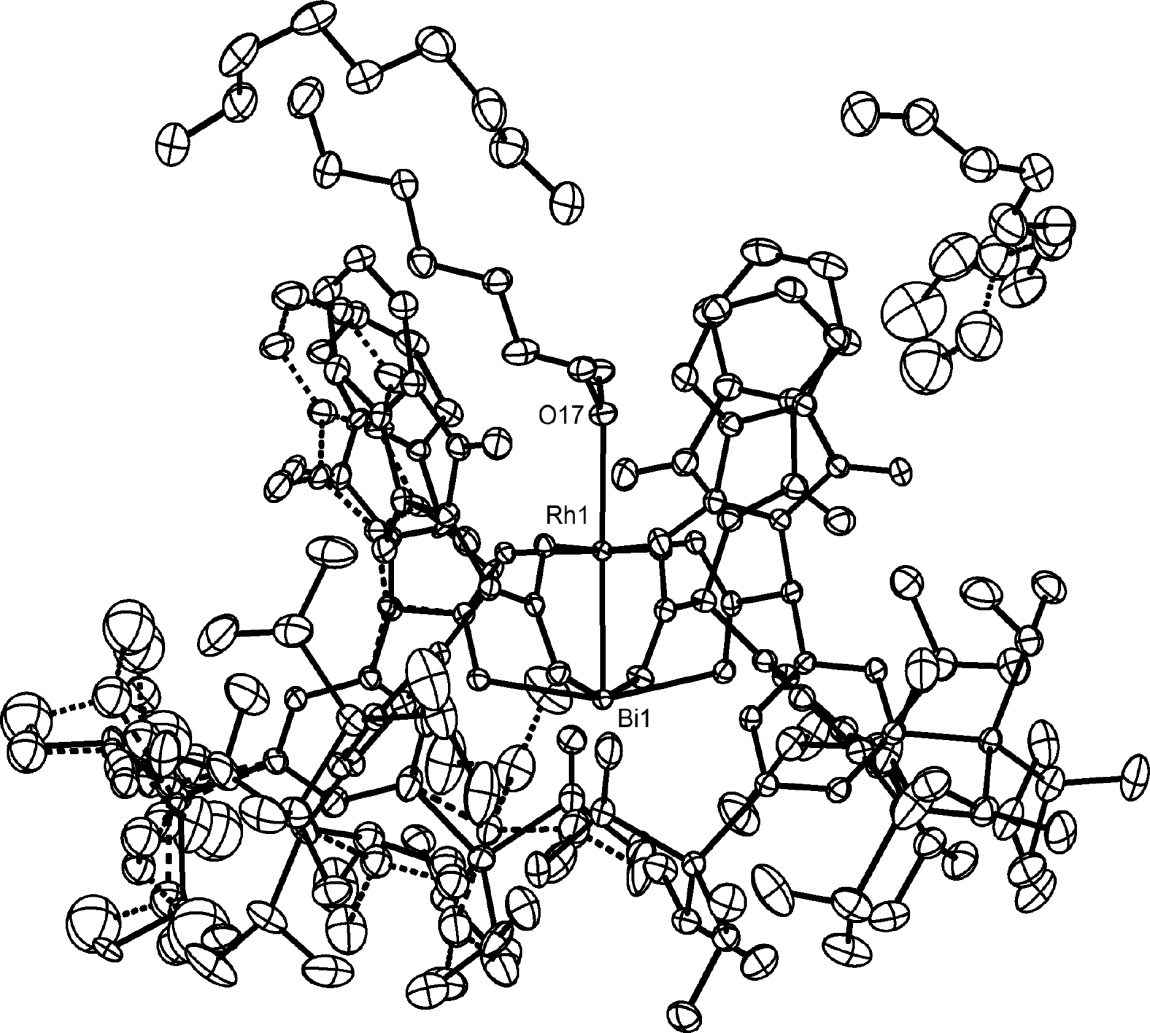
Structure of complex **3a** in the solid state; disordered parts are shown with dashed bonds, H atoms omitted for clarity; for further information, see the SI.

This aspect was addressed by DFT calculations.^66,67^ To this end, the structures of **3a**,**b** were computed with and without D3 (BJ) dispersion correction at the PBE level of theory (see the SI).^68,69^ Details apart, the computations are unambiguous in that dispersion renders the structures notably more compact. In energetic terms, it entails stabilization of **3a** and **3b** by no less than −9.9 and −11.6 kcal·mol^–1^, respectively.^70,71^ LD is therefore clearly a major structure determinant.^72^ Deconvolution validates the original design concept:^73^ the TIPS-groups account for ∼32% of Δ*E*_disp_, whereas the contribution of the *t*Bu groups in **3b** is smaller but still appreciable (∼12%); in structural terms, however, this extra factor visibly tightens the chiral binding site on the Rh-face in **3b** (Figure 3).

**Figure 3:**
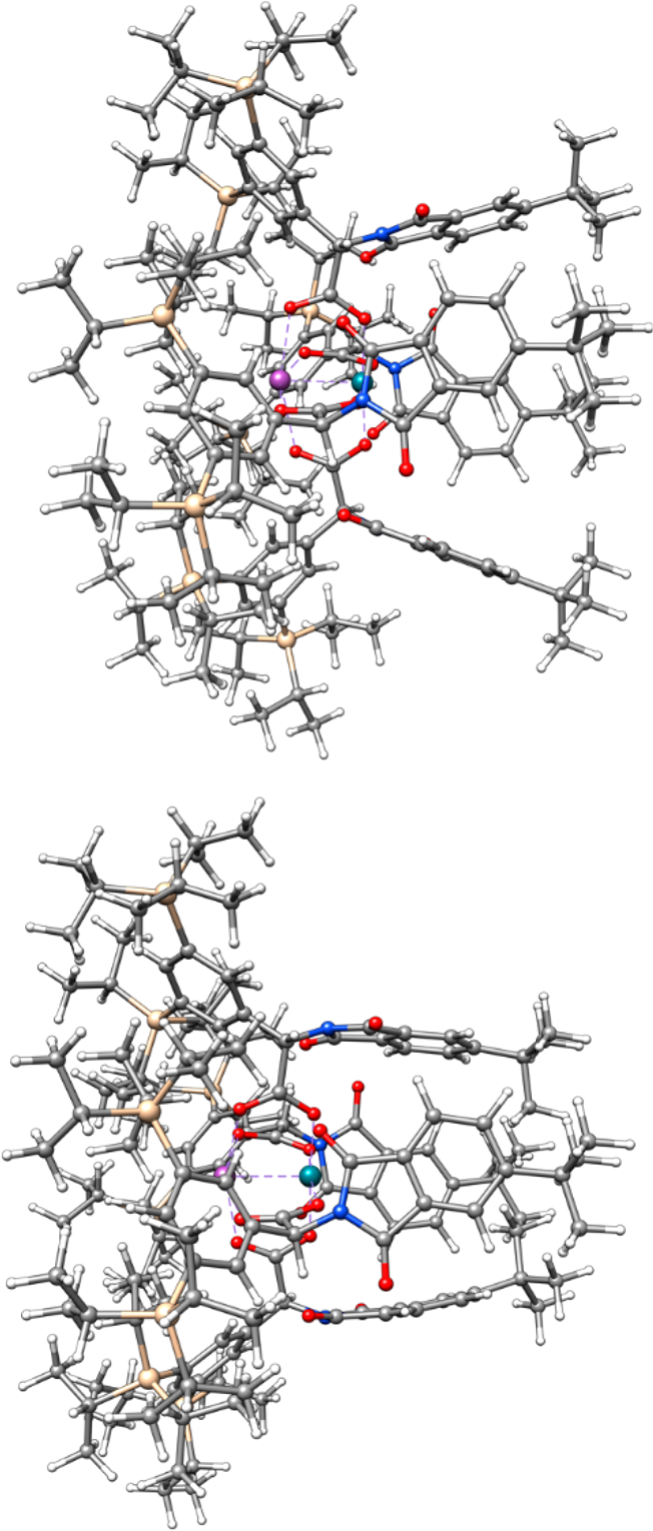
Side-view of the structure of **3b** as computed at the PBE/def2-SVP level of theory without (top) and with (bottom) D3 (BJ) dispersion correction

To explore the catalytic performance of the new heterobimetallic complexes, the cyclopropanation of styrene with the fluorinated diazo derivative **4b** was chosen as a test reaction (Table 1). Neither [Rh_2_(PTTL)_4_] (**1a**) nor [BiRh(PTTL)_4_] (**2a**) gave good results,^34^ whereas the new complex **3b** furnished product **5b** (R = Me) with 90% ee. The corresponding trichloroethyl ester proved even more successful:^74,75^ once again, replacement of **2a** by either of the new complexes led to massive improvements. The fact that **3a** and **3b** both perform very well but **3b** is the better of the two is in excellent accord with the conclusions drawn from the DFT calculations: the peripheral −TIPS groups make the larger contribution to the stabilization of the chiral ligand sphere, but the *t*Bu– substituents on the phthalimide residues pay an additional dividend. This notion is further supported by a control experiment with a catalyst analogous to **3a** but lacking the peripheral TIPS-groups, which gave product **5b′** with only 24% ee (see the SI). The fact that the outcome is fairly independent of the medium (pentane versus CH_2_Cl_2_) can be taken as additional indirect evidence for LD being operative in solution.^76^ Under the optimized conditions, product **5b′** was formed in essentially quantitative yield with 98% ee as a single diastereomer.

**Table 1:**
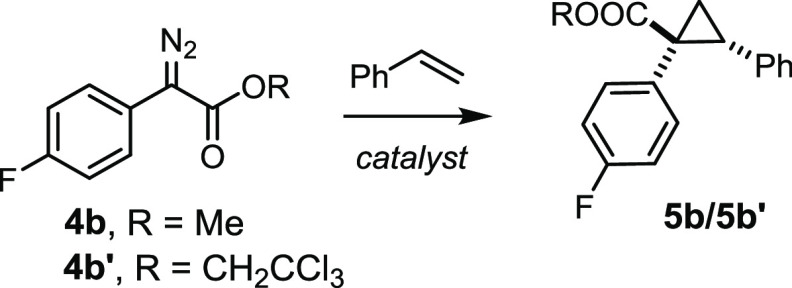
Screening^a^

Entry	R	Catalyst	Mol %	ee
1	Me	**1a**	1	32%^b^
2	Me	**2a**	1	35%^b^
3	Me	**3b**	1	90%^b^
4	CH_2_CCl_3_	**2a**	1	58%
5	CH_2_CCl_3_	**3a**	1	91%
6	CH_2_CCl_3_	**3b**	1	95%
7	CH_2_CCl_3_	**3b**	1	94%^c^
8	CH_2_CCl_3_	**3b**	1	98%^b^
9	CH_2_CCl_3_	**3b**	0.1	98%^b^^,^^e^
10	CH_2_CCl_3_	**3b**	0.005	97%^b^^,^^e^
11	CH_2_CCl_3_	**3b**	0.001	n.d.^d^

a All reactions were performed in pentane at RT; the yield of product (NMR) was ≥90%.

b At −10 °C.

c In CH_2_Cl_2_.

d Incomplete conversion after 24 h.

e 1 mmol scale

Formal replacement of a Rh(+2) center by Bi(+2) usually entails a loss in reactivity.^34−38^ When seen against this backdrop, the rate with which cyclopropane **5a** was formed by **3** even at −10 °C is truly remarkable (Figure 4): conversion was complete within 10 min at 0.25 mol % catalyst loading, whereas it took >3 h with the parent complex **2a**.^77^ The fact that the visibly more crowded catalysts lead to notably higher rates—in contrast to what one would expect from a confined space—indicates stabilizing LD interactions with the incoming substrate. The new design hence entails excellent selectivity and reactivity at the same time. The high solubility of **3a**,**b** in the common solvents even at low temperatures is an additional asset.

**Figure 4:**
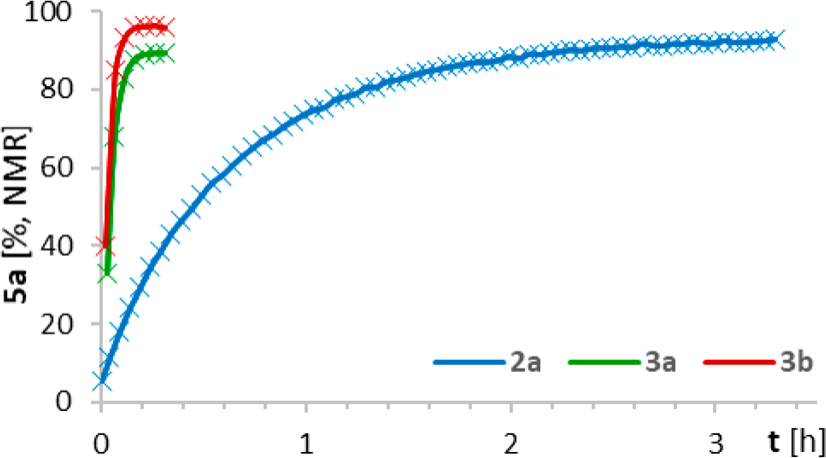
Kinetic profiling: Formation of **5a** using different BiRh-paddlewheel complexes (0.25 mol %, pentane, −10 °C).

These rewarding results suggested that it should be possible to reduce the loading to partly compensate for the high molecular weights of **3a**,**b**. Indeed, no drop in productivity or optical purity was noticed—without any further optimization of the conditions—when the reaction was performed with only 0.005 mol % of **3b** on a mmol scale (Table 1, entry 10).^78^ The robustness of these conditions was confirmed by the additional example shown in Table 2 (entry 6, 1.3 g scale).^79^

**Table 2:**
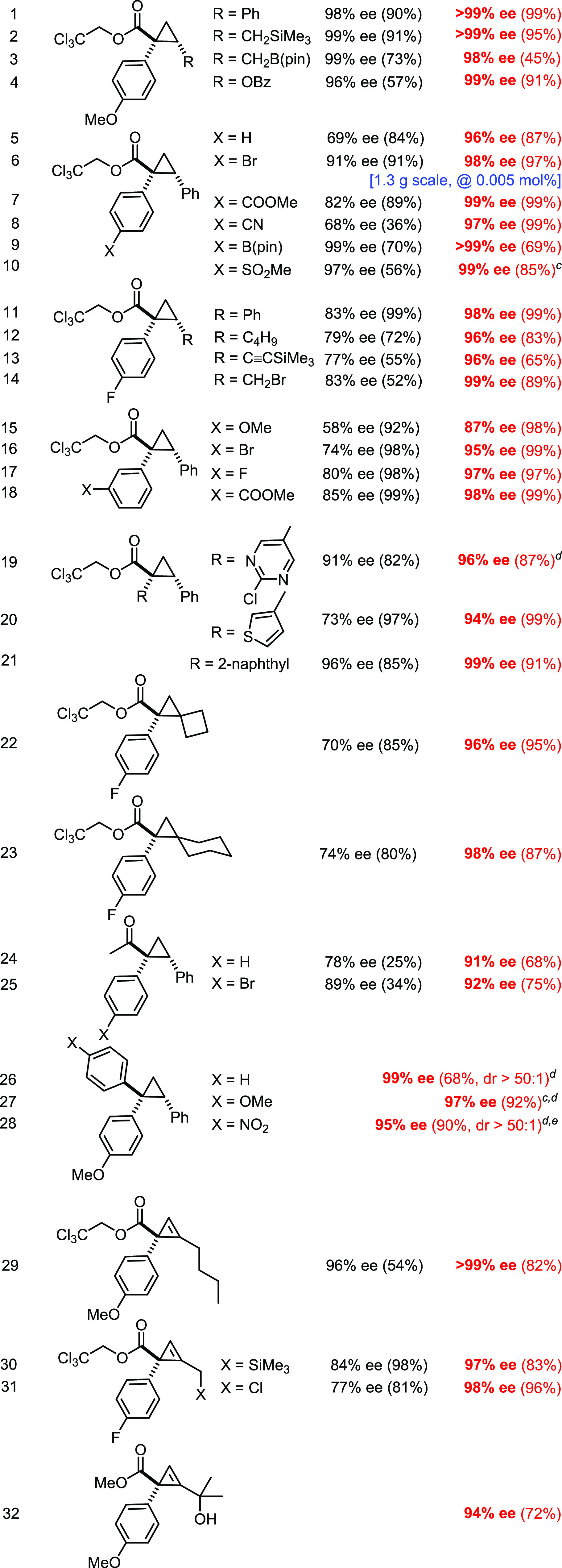
Asymmetric [2 + 1] Cycloadditions^a^^,^^b^

a Color code: black, **2a**; red, **3b**.

b Pentane, −10 °C, 1 mol % catalyst loading.

c In CH_2_Cl_2_/pentane.

d At rt.

e In CH_2_Cl_2_

The scope is broad, the level of induction is invariably excellent, and the reactions are typically very clean; therefore, basically quantitative yields were obtained in most cases (Table 2). α-Aryl-α-diazo esters comprising a strong donor substituent perform so well with the parent complex [BiRh(*S*-PTTL)_4_] (**2a**) that there was no real need to resort to **3a**,**b** (entries 1–4).^34^ However, diazoesters comprising less electron-rich aryl groups strongly benefitted from their use: in all cases investigated, **3b** led to significantly higher levels of induction, with ee’s often approaching 99% (entries 5–21). This striking invariance of the results upon substantial modulation of the diazo compound is deemed another favorable distinguishing feature of the new catalyst.^80^

Excellent results were also obtained with donor/acceptor α-diazo ketones (entries 24, 25), which previously required forcing conditions and recourse to special media.^81^ With catalyst **3b**, these challenging substrates react even at −10 °C. Likewise, donor/donor carbenes are well behaved,^2,82,83^ again furnishing the corresponding cyclopropanes with outstanding levels of enantio- and diastereoselectivity (entries 26–28).

In addition to (substituted) styrenes, many other terminal alkenes proved suitable, including vinyl benzoate, allyltrimethylsilane, and allyl(pinacol)boronate, some of which have been rarely used before. Even allyl bromide reacts cleanly (entry 14): we are unaware of any prior use in asymmetric cyclopropanation. The same is true for *exo*-methylenecyclobutane and -hexane, which furnish spirocyclic products (entries 22, 23); in these cases, the superiority of **3b** is particularly evident.

Although less comprehensive, the foray into the cyclopropenation of terminal alkynes was no less gratifying (entries 29–32). Most notable is the use of propargyl chloride, which leads to a multifunctional building block that promises rich downstream chemistry. Equally remarkable and largely unprecedented is the fact that cyclopropenation of a terminal alkyne is faster than insertion of the carbene intermediate into an unprotected alcohol (entry 32).^84−87^ To put these results into perspective, one has to note that [Rh_2_(esp)_2_]^88^ or [Rh_2_(OTpa)_4_] (OTpa = triphenylacetate) as some of the most proficient achiral catalysts known to date furnished only complex mixtures on reaction with these functionalized alkynes.^89^

Finally, the new catalysts were briefly examined in reactions other than [2 + 1] cycloadditions. Although a more systematic foray has to await further studies, the preliminary data obtained for prototype C–H and Si–H insertion reactions are promising (Table 3). When compared with the current state-of-the-art,^90^ the formation of a benzodihydrofuran by intramolecular insertion of a donor/donor carbene into a methyl ether makes a particularly compelling case (entry 2). Whereas **2a** performed poorly in the particularly taxing reaction of **4a** with Et_3_SiH, **3b** resulted in clean conversion and an excellent overall result.^11,91−98^

**Table 3:**
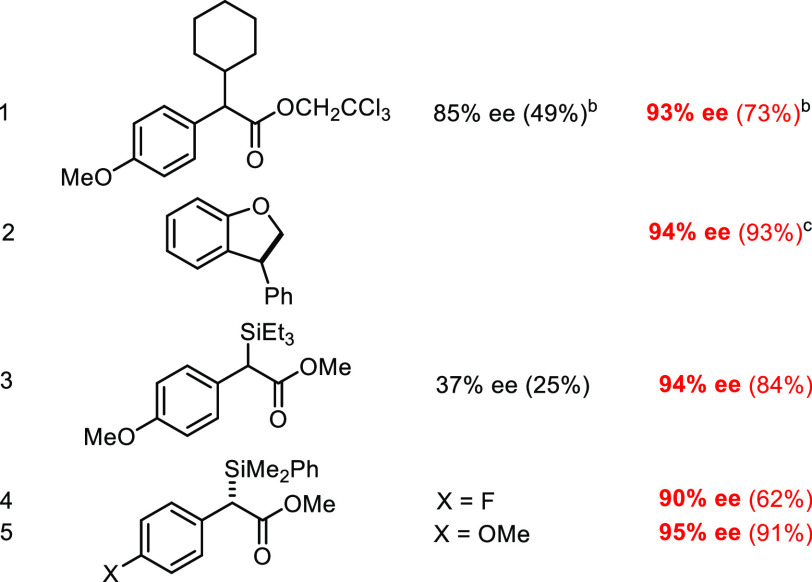
C–H and Si–H Insertion Reactions^a^

a Color code as shown in Table 2; pentane, −10 °C, 1 mol % catalyst loading.

b In cyclohexane.

c With [Rh_2_(*R*-PTAD)_4_]: 56% ee (49%).^90^

In summary, a new class of heterobimetallic paddlewheel complexes is presented, which take advantage of LD to stabilize the shape and directionality of the calyx that forms the chiral binding pocket. **3a**,**b** as the parent complexes of this series excel in cyclopropanation, cyclopropenation, and certain insertion reactions; as many variations of the conceptually new design can be envisaged, one may have high expectations for future generations of catalysts of this type. Opportunities along these and related lines^99,100^ are actively pursued in our laboratory.

## Supporting Information Available

Experimental Section containing supporting crystallographic data, computational data, characterization data, and NMR spectra of new compounds (PDF)

## Accession Codes

CCDC 2063745–2063748 contain the supplementary crystallographic data for this paper. These data can be obtained free of charge via www.ccdc.cam.ac.uk/data_request/cif, or by emailing data_request@ccdc.cam.ac.uk, or by contacting The Cambridge Crystallographic Data Centre, 12 Union Road, Cambridge CB2 1EZ, UK; fax: +44 1223 336033.
