# Supplementary material for: A New Ligand Design Based on London Dispersion Empowers Chiral Bismuth–Rhodium Paddlewheel Catalysts
Source: J Am Chem Soc. 2021 Apr 8;143(15):5666–73. doi: 10.1021/jacs.1c01972 (PMC8154533; doi:10.1021/jacs.1c01972)
Supplement: Supplementary file 1 — ja1c01972_si_001.pdf [file ja1c01972_si_001.pdf]

SUPPORTING INFORMATION

A New Ligand Design Based on London Dispersion Empowers Chiral Bismuth-Rhodium Paddlewheel Catalysts

Santanu Singha,[§] Michael Buchsteiner,[§] Giovanni Bistoni, Richard Goddard, and Alois Fürstner*

Max-Planck-Institut für Kohlenforschung, 45470 Mülheim/Ruhr, Germany

Email: fuerstner@kofo.mpg.de

[§] these authors contributed equally

TABLE OF CONTENTS

Supporting Crystallographic Data....S2

General....S12

Kinetic Studies....S12

Exploratory Studies....S14

Preparation of Heterobimetallic [BiRh] Complexes....S15

[BiRh(OTfa)$_4$]....S15

Preparation of Complexes **3a** and **3b**....S16

Control Experiment with the Catalyst Lacking the Peripheral TIPS-Groups: [BiRh(*S*)-PTPG)$_4$]....S21

Diazo Compounds....S22

Cyclopropanes and Cyclopropenes....S24

C–H and Si–H Insertion Reactions....S56

NH-Insertion and Doyle-Kirmse Reaction....S62

Supporting Computational Information....S64

Optimized Structures in XYZ Format....S65

Spectra....S95

References....S179

Supporting Crystallographic Data

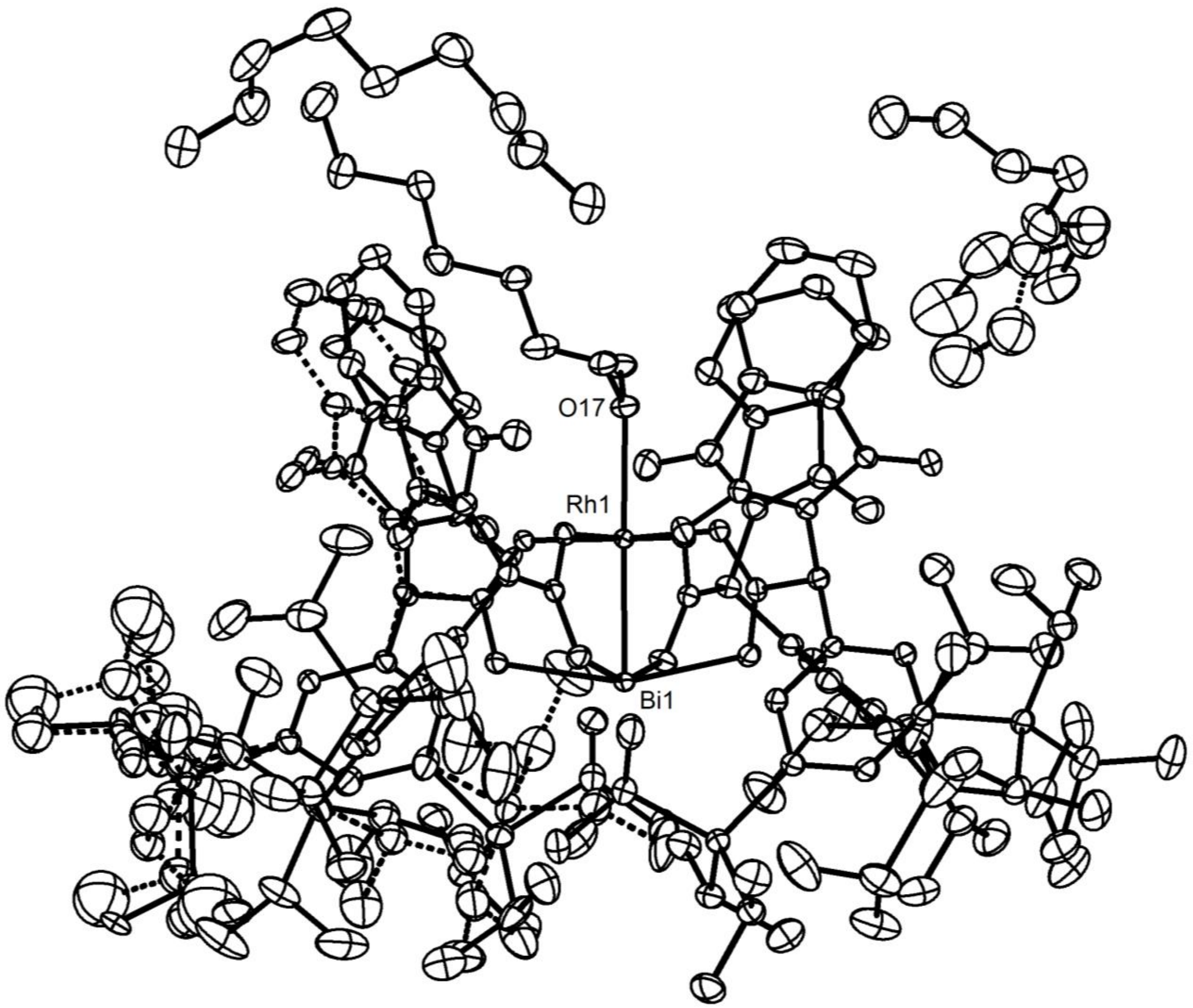

Figure S1. Molecular structure of complex **3a**. Atomic displacement ellipsoids are shown at the 50 % probability level. Disordered parts of the molecule are shown with dashed bonds. H atoms are omitted for clarity.

X-ray Crystal Structure Analysis of Complex 3a: $C_{142}H_{214}BiN_4O_{19}RhSi_8 \cdot 2(C_6H_{14}O_3)$, M_r = 3086.11 g · mol^{-1}, yellow plate, crystal size 0.06 x 0.08 x 0.14 mm^3, triclinic, space group *P*-1 [2], a = 17.791(3) Å, b = 22.062(4) Å, c = 24.399(2) Å, α = 69.943(11)°, β = 72.801(14)°, γ = 66.640(9)°, V = 8116(2) Å^3, T = 100(2) K, Z = 2, D_{calc} = 1.263 g · cm^3, λ = 0.71073 Å, $\mu(Mo\text{-}K_\alpha)$ = 1.308 mm^{-1}, face-indexed absorption correction (*SADABS*, $T_{\min}$ = 0.71878, $T_{\max}$ = 0.86434), Bruker-AXS Mach3 diffractometer with Kappa-CCD detector and FR591 molybdenum rotating

anode X-ray source equipped with Incoatec Helios X-ray optics, 2.630 < θ < 33.073°, 430062 measured reflections, 61295 independent reflections, 48905 reflections with $I > 2\sigma(I)$, $R_{int} = 0.0812$, 99.6 % coverage with an average redundancy of 6.99 to 0.65 Å resolution.

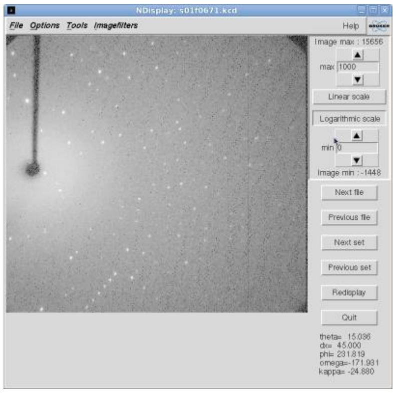

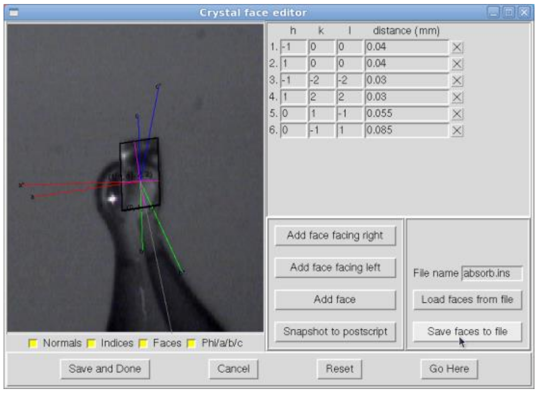

INTENSITY STATISTICS FOR DATASET

Resolution	#Data	#Theory	%Complete	Redundancy	Mean I	Mean I/s	Rmerge	Rsigma
Inf - 2.61	923	958	96.3	11.15	59.39	66.18	0.0440	0.0118
2.61 - 1.76	2159	2159	100.0	10.16	43.46	52.27	0.0344	0.0141
1.76 - 1.40	3079	3081	99.9	9.85	26.81	41.89	0.0406	0.0174
1.40 - 1.22	3153	3153	100.0	9.60	20.38	35.30	0.0483	0.0209
1.22 - 1.11	3038	3038	100.0	9.27	17.12	30.68	0.0548	0.0246
1.11 - 1.03	3171	3171	100.0	8.73	15.04	26.30	0.0603	0.0291
1.03 - 0.97	3066	3066	100.0	8.14	12.51	21.89	0.0722	0.0358
0.97 - 0.92	3196	3196	100.0	7.73	10.44	18.18	0.0861	0.0434
0.92 - 0.88	3120	3120	100.0	7.39	9.26	15.84	0.0976	0.0505
0.88 - 0.85	2707	2707	100.0	7.11	8.21	13.92	0.1086	0.0586
0.85 - 0.82	3162	3162	100.0	6.75	7.58	12.49	0.1240	0.0668
0.82 - 0.79	3664	3664	100.0	6.47	6.91	10.99	0.1364	0.0763
0.79 - 0.77	2778	2778	100.0	6.15	6.61	10.23	0.1495	0.0841
0.77 - 0.75	3031	3031	100.0	5.87	5.90	8.83	0.1782	0.0987
0.75 - 0.73	3442	3442	100.0	5.69	5.28	7.74	0.1966	0.1133
0.73 - 0.71	3718	3718	100.0	5.46	4.69	6.66	0.2200	0.1331
0.71 - 0.70	2142	2142	100.0	5.26	4.30	5.91	0.2394	0.1503
0.70 - 0.68	4479	4479	100.0	5.12	3.84	5.20	0.2757	0.1760
0.68 - 0.67	2451	2451	100.0	4.91	3.44	4.42	0.3069	0.2094
0.67 - 0.66	2595	2595	100.0	4.85	3.23	3.95	0.3408	0.2391
0.66 - 0.65	2221	2427	91.5	4.29	3.08	3.57	0.3561	0.2677
0.75 - 0.65	21048	21254	99.0	5.13	4.07	5.53	0.2566	0.1680
Inf - 0.65	61295	61538	99.6	6.99	11.24	17.20	0.0772	0.0497

A number of low-angle refections were shadowed by the beamstop and removed from the dataset before the final refinement cycles. Part of one solvent diglyme molecule, one 1,3-dioxo-1,3-dihydro-2*H*-isoindol-2-yl group, one isopropyl group on one otherwise not disordered tris-isopropylsilyl group and two tris-isopropylsilyl groups are disordered. One of the tris-isopropylsilyl groups is disordered over two positions (50:50 percent) and one over three positions (60:25:15 percent). The part of the diglyme molecule that is disordered is disordered over two positions (70:30 percent). Atoms of disordered parts were refined with anisotropic atomic displacement parameters when possible, whereby atoms of minor components and atoms in disordered groups in close proximity were refined with isotropic atomic displacment parameters in order to avoid high correlations. For the minor component of one tris-isopropylsilyl group (occupancy 0.15, third of three components, residue 9), the C-C distances of two isopropyl groups were restrained to be equal with an effective standard deviation of 0.005 and the atomic displacement parameters of the carbon atoms of two isopropyl groups were constrained to be equal. The second parameter of WGHT in SHELXL is 10.35 which can be attributed to the disorder in the structure. The environments of the carbon atoms range from tightly-bound atoms close to the centre of the complex to disordered atoms at the periphery. As a result, non-solvent carbon atoms have an Ueq(max)/Ueq(min) range of 10.0. The diffraction data were collected to a resolution of 0.65 Å. We cannot rule out that the residual electron density close to the bismuth atom is a result of anharmonic displacement of the heavy atom (83 electrons) since the diffraction data were corrected for the effects of absorption using indexed faces. In all, diffraction data were collected from six crystals, four were of the enantiopure (99.9% ee) and two racemates. The enantiopure crystals diffracted poorly. The quality of the diffraction data from one of the two racemate crystals (the current crystal) was by far the best. The crystals contain bound (to the rhodium atom) as well as solvent diglyme. The structure was solved by *SHELXT* and refined by full-matrix least-squares (*SHELXL*) against F^2. Hydrogen atoms were refined using a riding model. Refinement of the structure resulted in $R1 = 0.0442$ for 48905 [$I > 2\sigma(I)$] and 0.0670 for all 61295 data, 2074 parameters refined, 4 restraints (C-C bond lengths in two disordered minor component isopropyl groups), $wR2 = 0.0833$, $GooF = S = 1.069$, residual electron density +1.01 (0.58 Å from Bi1) / −2.81 (0.74 Å from Bi1) e · Å^{-3}. **CCDC-2063745**.

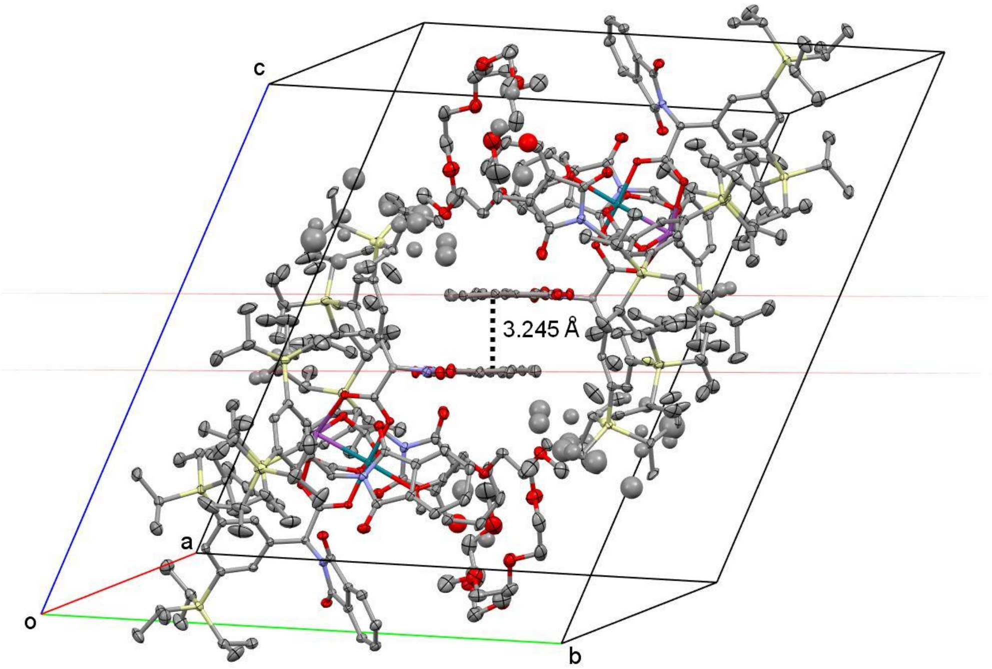

Figure S2. Packing of the molecules of complex **3a** within the triclinic unit cell, showing the arrangement of disordered phthalimido groups on two adjacent molecules.

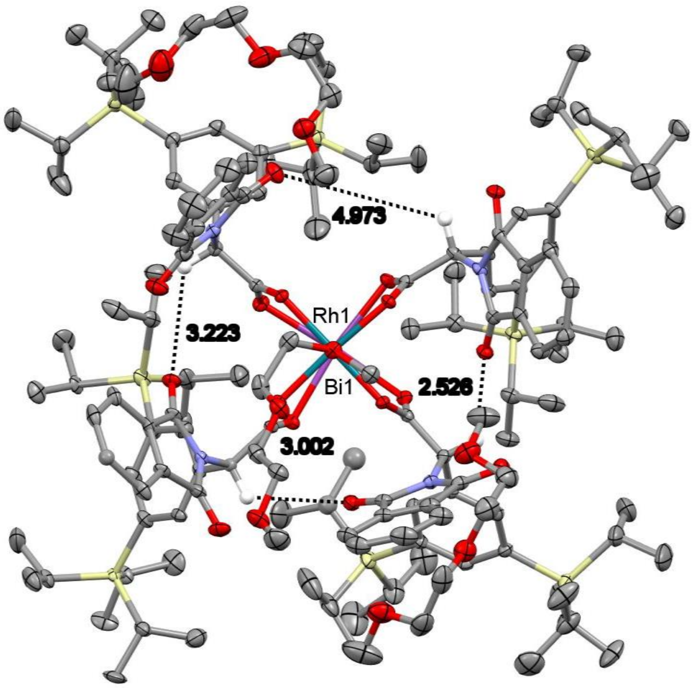

Figure S3. Major component of complex **3a** viewed along the Rh-Bi vector showing possible intramolecular C-H····O interactions between methanetriyl hydrogen atoms and oxygen atoms on adjacent phthalimido groups.

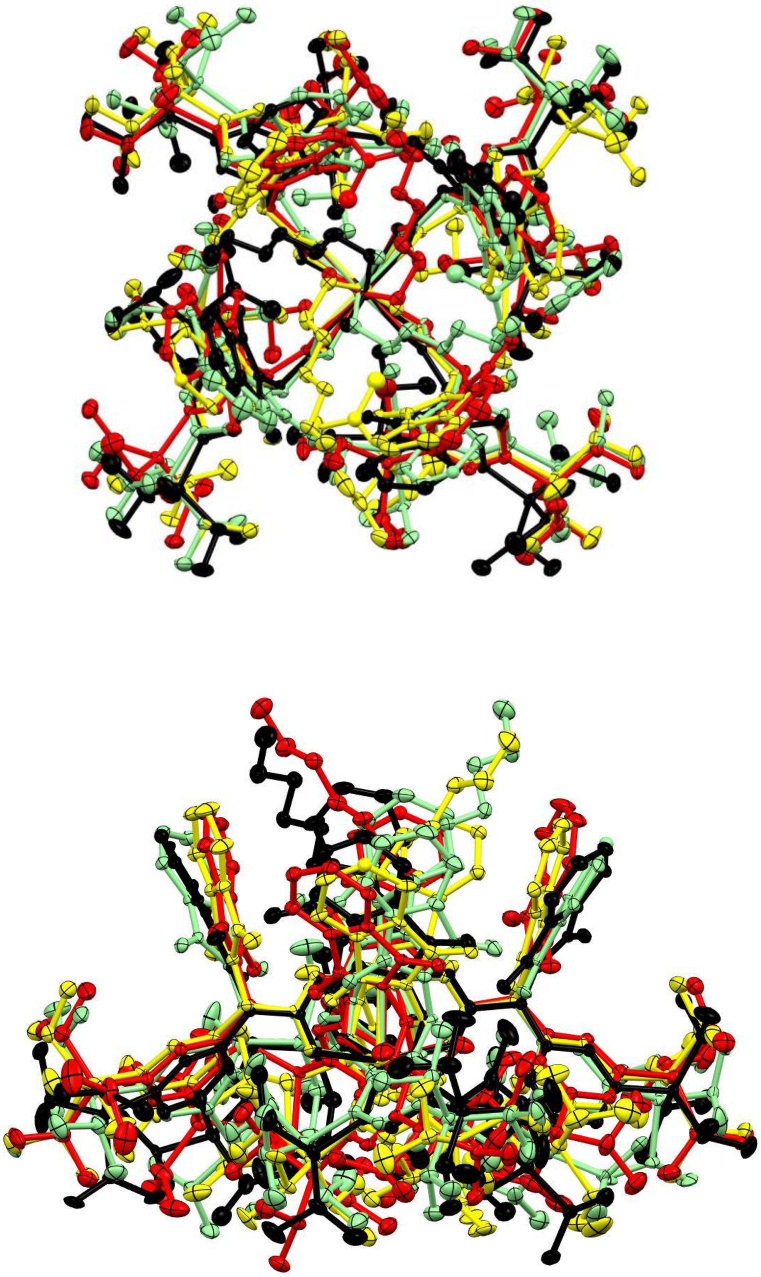

Figure S4. Overlay of central O8RhBi units of four molecules of complex **3a** rotated by respectively 90° about the Rh-Bi direction to illustrate the approximate C_4 symmetry of the molecule (top and side view).

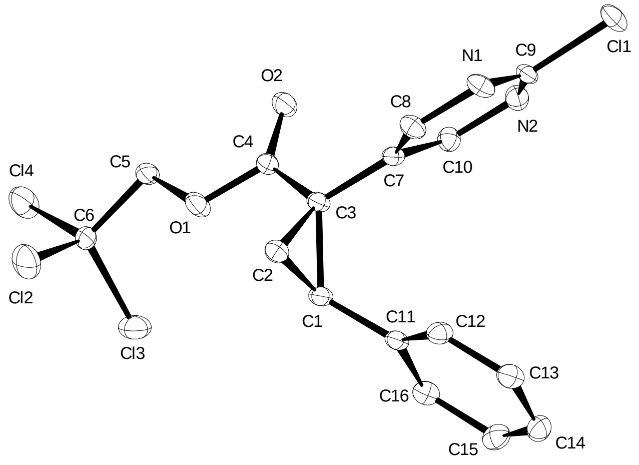

Figure S5. Structure of cyclopropane **S20** in the solid state. H atoms have been removed for clarity.

X-Ray Crystal Structure Analysis of Compound S20. $C_{16}H_{12}Cl_4N_2O_2$, Mr = 406.08 g/mol, colourless plate, crystal size 0.051 x 0.022 x 0.013 mm^3, orthorhombic, space group P212121, [No. 19], *a* = 6.0034(3) Å, *b* = 16.7746(8) Å, *c* = 16.8953(9) Å, *V* = 1701.43(15) Å^3, *T* = 100(2) K, *Z* = 4, *Dcalc* = 1.585 mg·m^{-3}, *λ* = 0.71073 Å, *μ(Mo-Kα)* = 0.707 mm^{-1}, analytical absorption correction (*Tmin* = 0.95449, *Tmax* = 0. 98702), Bruker-AXS Kappa Mach3 diffractometer with APEX-II detector and IμS micro focus X-ray source, 1.711 < θ < 31.047°, 52940 measured reflections, 5435 independent reflections, 4897 reflections with *I>2σ(I)*, Rint = 0.0448.

INTENSITY STATISTICS FOR DATASET (Friedel pairs not merged)

Resolution	#Data	#Theory	%Complete	Redundancy	Mean I	Mean I/s	Rmerge	Rsigma
Inf - 2.88	82	82	100.0	16.62	97.34	108.28	0.0200	0.0071
2.88 - 1.87	198	198	100.0	17.58	45.89	101.13	0.0235	0.0079
1.87 - 1.48	272	272	100.0	18.34	27.65	82.38	0.0275	0.0094
1.48 - 1.30	271	271	100.0	17.53	16.96	64.54	0.0389	0.0122
1.30 - 1.18	277	277	100.0	17.37	17.77	60.79	0.0413	0.0131
1.18 - 1.10	267	267	100.0	15.78	14.90	50.39	0.0512	0.0154
1.10 - 1.03	280	280	100.0	12.56	8.86	34.00	0.0746	0.0238
1.03 - 0.97	331	331	100.0	9.92	7.41	26.80	0.0810	0.0302
0.97 - 0.93	270	270	100.0	8.79	7.02	23.91	0.0911	0.0345
0.93 - 0.90	223	223	100.0	7.95	6.50	21.90	0.0870	0.0383
0.90 - 0.87	257	257	100.0	7.53	5.72	18.91	0.0908	0.0441
0.87 - 0.84	304	304	100.0	6.92	5.88	17.86	0.0890	0.0463
0.84 - 0.81	349	349	100.0	6.69	4.55	14.70	0.1068	0.0581
0.81 - 0.79	265	265	100.0	6.66	4.02	12.44	0.1196	0.0654
0.79 - 0.77	278	278	100.0	6.50	3.54	11.26	0.1352	0.0754
0.77 - 0.75	313	313	100.0	6.32	3.46	10.47	0.1480	0.0807
0.75 - 0.74	181	181	100.0	5.75	2.62	8.06	0.1733	0.1036
0.74 - 0.72	382	382	100.0	5.75	2.78	8.21	0.1771	0.1043
0.72 - 0.71	214	214	100.0	5.76	2.65	7.62	0.1823	0.1107

0.71 - 0.70	221	221	100.0	5.63	2.67	7.71	0.1862	0.1135
0.70 - 0.69	228	249	91.6	3.84	2.29	5.76	0.2085	0.1703
0.79 - 0.69	1817	1838	98.9	5.69	2.91	8.61	0.1655	0.1023
Inf - 0.69	5463	5484	99.6	9.69	10.54	29.87	0.0444	0.0258

The structure was solved by dual space methods (SHELXT) and refined by full-matrix least-squares (SHELXL) against F^2 with aspherical scattering factors for all atoms except Cl applied according to Luebben et al. [*Acta Cryst*. (2019). A75, 50-62] to $R1$= 0.0308 [$I > 2\sigma(I)$], $wR2$ = 0.0673, 224 parameters, 0 restraints, absolute structure parameter according to Parsons, Flack and Wagner = 0.011(16) [1987 quotients] [Parsons, Flack and Wagner, *Acta Cryst*. B69 (2013) 249-259], GooF = S = 1.041, residual electron density 1.15 e · $Å^{-1}$ [0.82 Å from CL4], -0.74 e · $Å^{-1}$ [0.66 Å from CL4]. **CCDC-2063746**

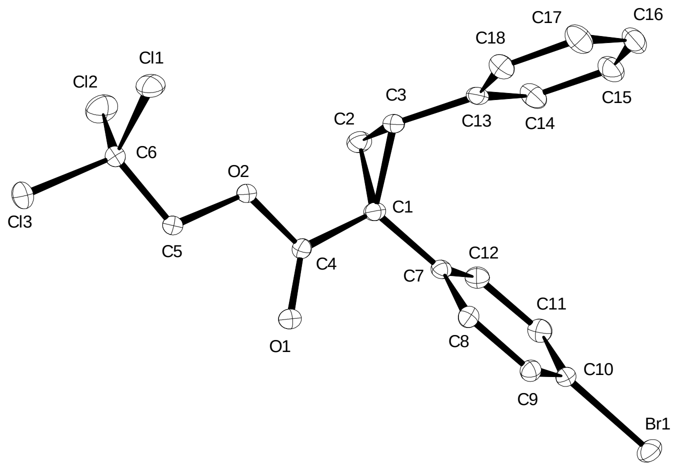

Figure S6. Structure of cyclopropane **S4** in the solid state. H atoms have been removed for clarity

X-Ray Crystal Structure Analysis of Compound S4. $C_{18}H_{14}BrCl_3O_2$, Mr = 448.55 g/mol, colourless plate, crystal size 0.078 x 0.045 x 0.024 mm^3, orthorhombic, space group P212121, [No. 19], a = 5.8058(3) Å, b = 17.1098(9) Å, c = 17.8442(10) Å, V = 1772.57(16) $Å^3$, T = 100(2) K, Z = 4, $Dcalc$ = 1.681 $mg \cdot m^{-3}$, λ = 0.71073 Å, μ ($Mo\text{-}K_\alpha$) = 2.779 mm^{-1}, analytical absorption correction (T_{min} = 0.86072, T_{max} = 0.94978), Bruker-AXS Kappa Mach3 diffractometer with APEX-II detector and IμS micro focus X-ray source, 1.649 < θ < 33.876°, 118713 measured reflections, 7107 independent reflections, 6803 reflections with $I>2\ \sigma(I)$, Rint = 0.0393.

INTENSITY STATISTICS FOR DATASET (Friedel pairs not merged)

Resolution	#Data	#Theory	%Complete	Redundancy	Mean I	Mean I/s	Rmerge	Rsigma
Inf - 2.64	107	107	100.0	22.84	86.50	110.25	0.0273	0.0082
2.64 - 1.75	254	254	100.0	27.52	45.56	117.63	0.0267	0.0075
1.75 - 1.38	358	358	100.0	28.05	28.84	109.24	0.0288	0.0079
1.38 - 1.20	369	369	100.0	27.27	23.00	98.14	0.0296	0.0084
1.20 - 1.09	366	366	100.0	25.92	18.07	86.63	0.0351	0.0093
1.09 - 1.01	364	364	100.0	21.01	12.68	70.67	0.0427	0.0117
1.01 - 0.95	361	361	100.0	18.64	10.67	60.33	0.0443	0.0135
0.95 - 0.90	390	390	100.0	16.84	9.08	52.01	0.0478	0.0156
0.90 - 0.87	283	283	100.0	15.91	9.22	49.43	0.0496	0.0165
0.87 - 0.83	401	401	100.0	15.18	8.02	44.59	0.0557	0.0187
0.83 - 0.80	402	402	100.0	14.74	6.32	37.22	0.0615	0.0222
0.80 - 0.78	288	288	100.0	14.22	4.96	31.45	0.0720	0.0272
0.78 - 0.75	485	485	100.0	13.74	4.88	29.29	0.0762	0.0288
0.75 - 0.73	365	365	100.0	13.09	4.26	25.17	0.0872	0.0338
0.73 - 0.71	413	413	100.0	12.76	3.88	22.40	0.0955	0.0380
0.71 - 0.70	240	240	100.0	12.58	3.38	20.37	0.1030	0.0435
0.70 - 0.68	508	508	100.0	12.20	3.16	18.92	0.1130	0.0476
0.68 - 0.67	246	246	100.0	11.85	3.37	18.93	0.1132	0.0481
0.67 - 0.66	298	298	100.0	11.35	2.56	14.56	0.1317	0.0621
0.66 - 0.65	276	276	100.0	11.23	2.52	14.39	0.1383	0.0647
0.65 - 0.64	365	420	86.9	7.49	2.26	10.90	0.1535	0.0998
0.74 - 0.64	2535	2590	97.9	11.41	3.14	17.98	0.1124	0.0520
Inf - 0.64	7139	7194	99.2	16.55	11.08	47.13	0.0391	0.0157

The structure was solved by dual space methods (SHELXT) and refined by full-matrix least-squares (SHELXL) against F^2 with aspherical scattering factors for all atoms except Cl and Br applied according to Luebben et al. [*Acta Cryst*. (2019). A75, 50-62]. H atom postions were calculated and allowed to ride with extended averaged refined C-H distances. *R1*= 0.0164 [$I>2\sigma(I)$], *wR2* = 0.0377, 220 parameters, 0 restraints, absolute structure parameter according to Parsons, Flack and Wagner = -0.0035(11) [2854 quotients] [Parsons, Flack and Wagner, *Acta Cryst*. B69 (2013) 249-259], GooF = S = 1.051, residual electron density 0.67 e · $Å^{-1}$ [0.76 Å from CL3], -0.29 e · $Å^{-1}$ [0.59 Å from CL3]. **CCDC-2063747**

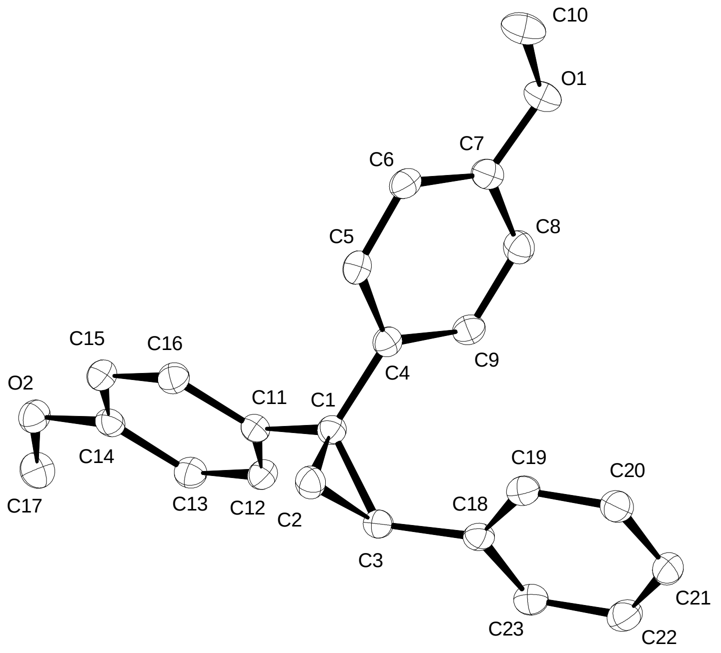

Figure S7. Structure of cyclopropane **S28** in the solid state. H atoms have been removed for clarity.

X-Ray Crystal Structure Analysis of Compound S28. $C_{23}H_{22}O_2$, Mr = 330.40 g/mol, colourless prism, crystal size 0.051 x 0.131 x 0.159 mm^3, monoclinic, space group P21, [No. 4], a = 12.3391(7) Å, b = 5.8750(3) Å, c = 12.7465(7) Å, β = 110.721(2)°, V = 846.25(8) Å^3, T = 100(2) K, Z = 2, $Dcalc$ = 1.270 mg·m^{-3}, λ = 1.54178 Å, μ (Mo-K_α) = 2.779 mm^{-1}, analytical absorption correction (T_{min} = 0. 92109, T_{max} = 0. 97129), Bruker-AXS Kappa Mach3 diffractometer with APEX-II detector and Bruker-Nonius FR591 rotating anode X-ray source, 3.707 < θ < 72.462°, 35434 measured reflections, 3412 independent reflections, 3330 reflections with $I>2\sigma(I)$, Rint = 0.0323.

INTENSITY STATISTICS FOR DATASET (Friedel pairs not merged)

Resolution	#Data	#Theory	%Complete	Redundancy	Mean I	Mean I/s	Rmerge	Rsigma
Inf - 3.29	53	53	100.0	13.53	179.81	110.83	0.0207	0.0077
3.29 - 2.22	118	118	100.0	12.88	54.24	105.59	0.0210	0.0083
2.22 - 1.75	173	173	100.0	13.54	35.22	98.59	0.0222	0.0089
1.75 - 1.52	170	170	100.0	10.54	23.17	79.64	0.0226	0.0106
1.52 - 1.38	175	175	100.0	10.83	14.79	67.73	0.0269	0.0118
1.38 - 1.29	164	164	100.0	10.88	11.56	62.36	0.0317	0.0131
1.29 - 1.20	197	197	100.0	9.73	15.25	64.30	0.0299	0.0132
1.20 - 1.14	168	168	100.0	9.70	18.66	60.93	0.0283	0.0128
1.14 - 1.10	153	153	100.0	9.30	13.18	53.41	0.0342	0.0148

1.10 - 1.06	168	168	100.0	10.82	12.35	52.27	0.0474	0.0150
1.06 - 1.02	166	166	100.0	14.17	11.18	51.26	0.0561	0.0156
1.02 - 0.98	206	206	100.0	12.16	6.84	36.02	0.0630	0.0219
0.98 - 0.96	144	144	100.0	12.47	6.04	34.99	0.0666	0.0234
0.96 - 0.93	208	208	100.0	11.96	6.19	32.47	0.0607	0.0226
0.93 - 0.91	139	139	100.0	11.49	4.56	29.06	0.0775	0.0278
0.91 - 0.89	178	178	100.0	9.85	5.31	31.36	0.0633	0.0283
0.89 - 0.87	176	176	100.0	9.85	3.94	26.61	0.0810	0.0310
0.87 - 0.85	192	192	100.0	8.09	3.64	23.12	0.0812	0.0348
0.85 - 0.83	217	217	100.0	7.59	2.96	21.30	0.0962	0.0395
0.83 - 0.82	119	119	100.0	6.29	3.44	23.12	0.0929	0.0395
0.82 - 0.81	136	155	87.7	3.09	3.17	14.75	0.1029	0.0618
0.91 - 0.81	1018	1037	98.2	7.63	3.75	23.66	0.0798	0.0368
Inf - 0.81	3420	3439	99.4	10.32	14.78	48.75	0.0322	0.0136

The structure was solved by dual space methods (SHELXT) and refined by full-matrix least-squares (SHELXL) against F^2 with aspherical scattering factors for all atoms applied according to Luebben et al. [*Acta Cryst*. (2019). A75, 50-62]. H atom postions were calculated and allowed to ride with extended averaged refined C-H distances. *R1*= 0.0210 [$I>2\sigma(I)$], *wR2* = 0.0525, 231 parameters, 1 restraint, absolute structure parameter according to Parsons, Flack and Wagner = -0.04(8) [1459 quotients] [Parsons, Flack and Wagner, *Acta Cryst*. B69 (2013) 249-259], GooF = S = 1.079, residual electron density 0.11 e · Å^{-1} [0.93 Å from C1], -0.12 e · Å^{-1} [1.09 Å from C3]. **CCDC- 2063748**

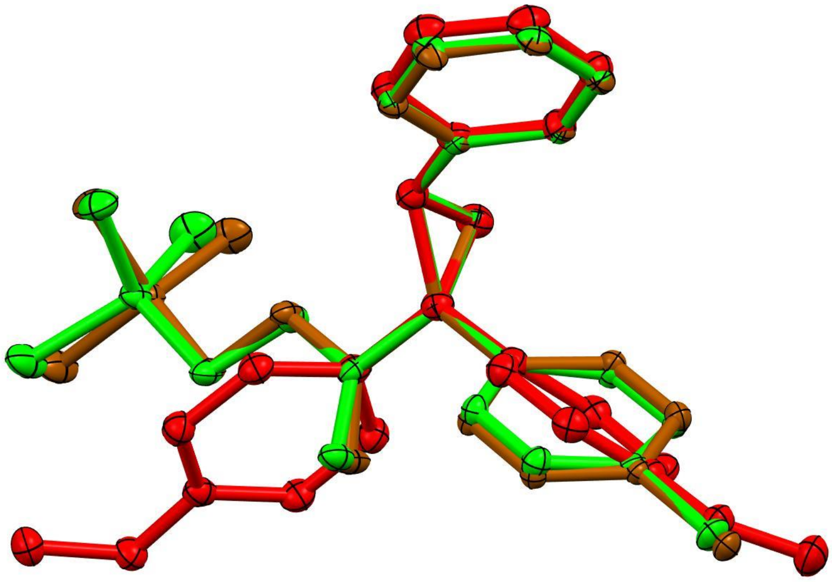

Figure S8. Overlay of the cyclopropane moieties of cyclopropanes **S20** (green), **S4** (brown) and **S28** (red) in their respective crystals, showing the differences in the conformations.

General

Unless stated otherwise, all reactions were carried out under argon atmosphere in flame dried Schlenk glassware. The solvents were purified by distillation over the indicated drying agents under argon: THF (Mg/anthracene), Et_2O (Mg/anthacene), pentane (Na/K), CH_2Cl_2 (CaH_2). MeCN and Et_3N were dried by an absorption solvent purification system based on molecular sieves. Flash chromatography: VWR Chemicals silica gel 40 – 63µm.

NMR spectra were recorded on Bruker DPX 300, AV 400, AV 500 or AV III 600 spectrometers in the solvents indicated; chemical shifts are given in ppm relative to TMS, coupling constants (*J*) in Hz. The solvent signals were used as references and the chemical shifts converted to the TMS scale ($CDCl_3$: δ_C = 77.2 ppm; residual $CHCl_3$: δ_H = 7.26 ppm; CD_2Cl_2: δ_C = 54.0 ppm; residual $CHDCl_2$: δ_H = 5.32 ppm). Proton and carbon assignments were established using HSQC, HMBC and NOESY experiments.

IR: Alpha Platinum ATR (Bruker), wavenumbers ($\tilde{\nu}$) in cm^{-1}.

MS (EI): Finnigan MAT 8200 (70 eV), ESI-MS: ESQ 3000 (Bruker),Thermo Scientific LTQ-FT, or Thermo Scientific Exactive. HRMS: Bruker APEX III FT-MS (7 T magnet), MAT 95 (Finnigan), Thermo Scientific LTQ-FT, or Thermo Scientific Exactive. GC-MS: Shimadzu GCMS-QP2010 Ultra instrument.

HPLC analyses for the determination of enantiomeric excesses were conducted on a Shimadzu LC 2020 instrument equipped with a Shimadzu SPD-M20A UV/VIS detector. Solvents (HPLC grade) were purchased and used as received. The exact conditions are stated separately for each compound.

Optical rotations were measured with an A-Krüss Otronic Model P8000-t polarimeter at a wavelength of 589 nm. The values are given as specific optical rotation with exact temperature, concentration (c/(10 mg/mL)) and solvent.

Unless stated otherwise, all commercially available compounds (abcr, Acros, TCI, Aldrich, Alfa Aesar, Fluoro Chem) were used as received.

Kinetic Studies

General: Reactions were monitored on a Bruker AV 500 NMR spectrometer at −10°C in non-deuterated pentane with coaxial-sample-insert filled with [D_6]-acetone for locking. During the reactions a spectrum was acquired every 3 min. The solvent signals of pentane were suppressed using the WET sequence with selective shaped pulses and ^{13}C decoupling. (Bruker sequence: wetdc).

The acquired spectra were imported into MNOVA 14.1.2 (Mestrelab Research S.L) with the reaction monitoring plugin for further processing. For the data analysis, the baseline of the spectra was corrected with a multiple point baseline correction.

Sample preparation: In flame-dried Schlenk tubes, the following stock solutions were prepared:

Stock solution 1: Methyl p-methoxyphenyldiazoacetate (c = 18 mmol/L) and styrene (c = 91 mmol/L) in pentane

Stock solution 2: Respective [BiRh] catalyst (c = 0.44 mmol/L) in pentane

Typical procedure: A flame-dried NMR tube under Ar was charged with an aliquot of the stock solution 1 (0.4 mL), which was cooled to −50°C in a dry ice/ethanol mixture. The solution was overlaid with an aliquot of stock solution 2 (41 µL) and the walls of the NMR tube were washed with a minimal amount of dry pentane. A capillary of [D_6]-acetone was inserted. The solution was mixed shortly before the start of the measurements and was warmed to −10 °C in the NMR machine. ^{1}H NMR spectra were recorded every 3 min.

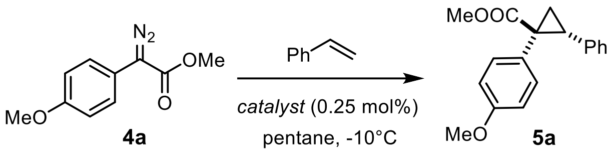

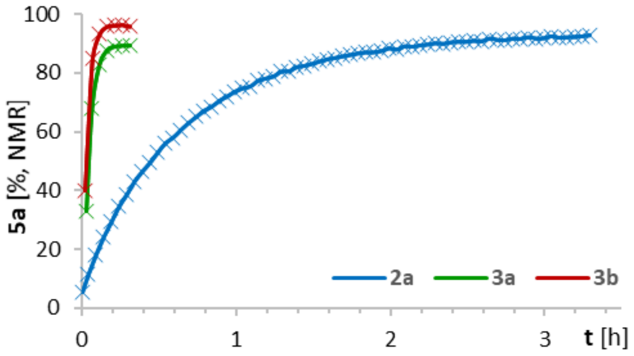

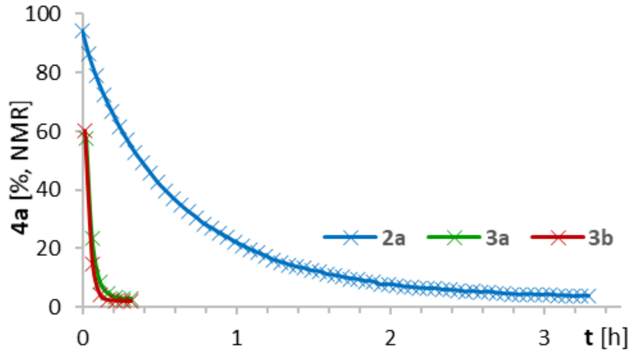

Figure S9. Kinetic data for the formation of the cyclopropane **5a** (top) as well as for the consumption of methyl *p*-methoxyphenyldiazoacetate **4a** (bottom); the reactions were performed with 0.25 mol% of catalyst in pentane at –10°C

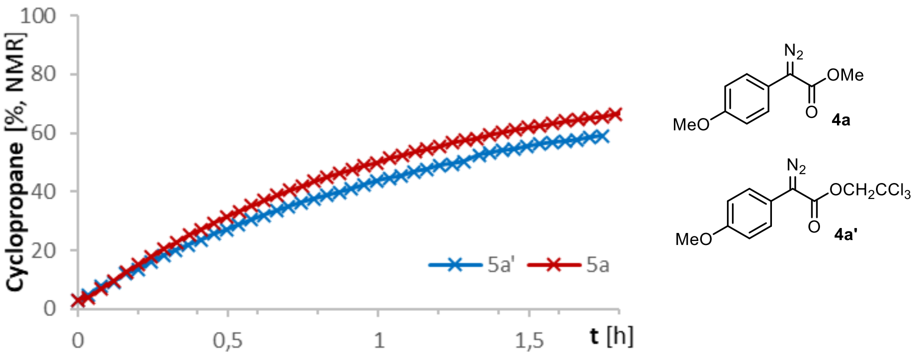

Figure S10. Kinetic data for the formation of cyclopropanes **5a** and **5a'**, showing that methyl 2-(4-methoxyphenyl)-2-diazoacetate (**4a**) and 2,2,2-trichloroethyl 2-(4-methoxyphenyl)-2-diazoacetate (**4a'**) react with very similar rates; the reactions were performed with 0.1 mol% of catalyst **3b** in CH_2Cl_2 at −10°C.

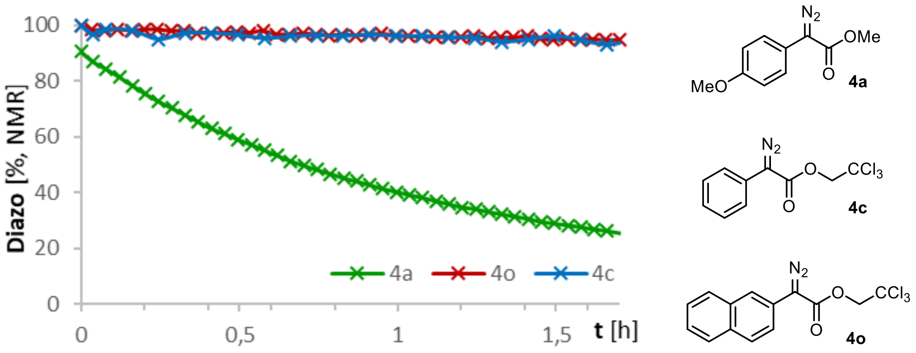

Figure S11. Kinetic data for the consumption of diazo compounds **4a**, **4c** and **4o**, showing the influence of the arene substituent on the reaction rate; the reactions were performed with 0.1 mol% of catalyst **3b** in CH_2Cl_2 at −10°C.

Exploratory Studies

It is well established that chiral dirhodium paddlewheel complexes are hardly adequate for enantioselective –OH or –NH insertion and Doyle-Kirmse reactions, except for special cases or if chiral co-catalysts are added that then largely account for the induction.[1,2,3,4] This inability is inherent and basically rooted in the dissociation of the (chiral) rhodium moiety from the ylide primarily formed before the enantiodetermining [1,2]-H shift does occur.[5] In line with this notion, the few test reactions performed so far with complexes **3** also afforded no or only modest enantioselectivity. Likewise, an attempt at performing an enynone rearrangement/Si–H insertion sequence has so far met with failure.[6] A comprehensive screening, however, has not yet been carried out and must await future studies.

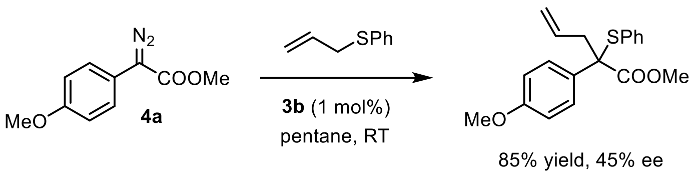

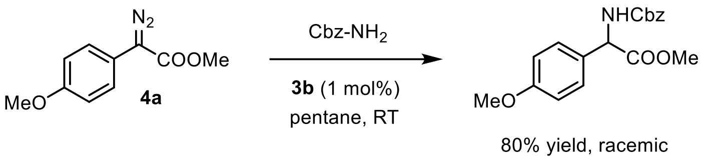

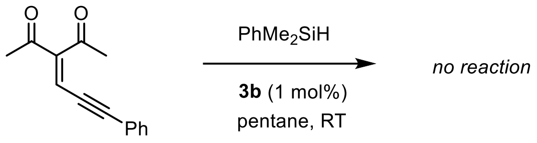

Preparation of Heterobimetallic [BiRh] Complexes

[BiRh($OCOCF_3$)$_4$]

[BiRh($OCOCF_3$)$_4$] was prepared by a modified literature procedure.[7] A flame-dried two-necked round bottom flask equipped with a reflux condenser was charged with [$Rh_2(OCOCF_3)_4$]·2 MeCN (534 mg, 0.722 mmol), which was heated (80°C, 10^{-3} mbar) for 1 h to remove any axially coordinated ligands; during this time, the color of the sample changed from purple to green. Next, $Bi(OCOCF_3)_3$ (415 mg, 0.757 mmol),[8] freshly ground Bi metal (817 mg, 3.91 mmol), toluene (40 mL), Ph_2O (1.1 mL, 6.93 mmol) and trifluoroacetic acid (200 µL, 2.61 mmol) were added. The mixture was stirred at 115 °C bath temperature. After 16 h, ^{19}F NMR showed full conversion of [$Rh_2(OCOCF_3)_4$]. At this point, remaining Bi metal was allowed to settle and the supernatant removed via cannula filtration. The yellow filtrate was concentrated in vacuo. Remaining Ph_2O was sublimed onto a -30°C-cold sublimation finger at 50°C and 10^{-3} mbar. The residue was purified by flash chromatography (silica), eluting with a toluene/MeCN gradient (100:0 → 90:10) to obtain the title compound as a yellow powder (855 mg, 82%). Characterization data matches with the previously reported data.[7]

Preparation of Complexes 3a and 3b

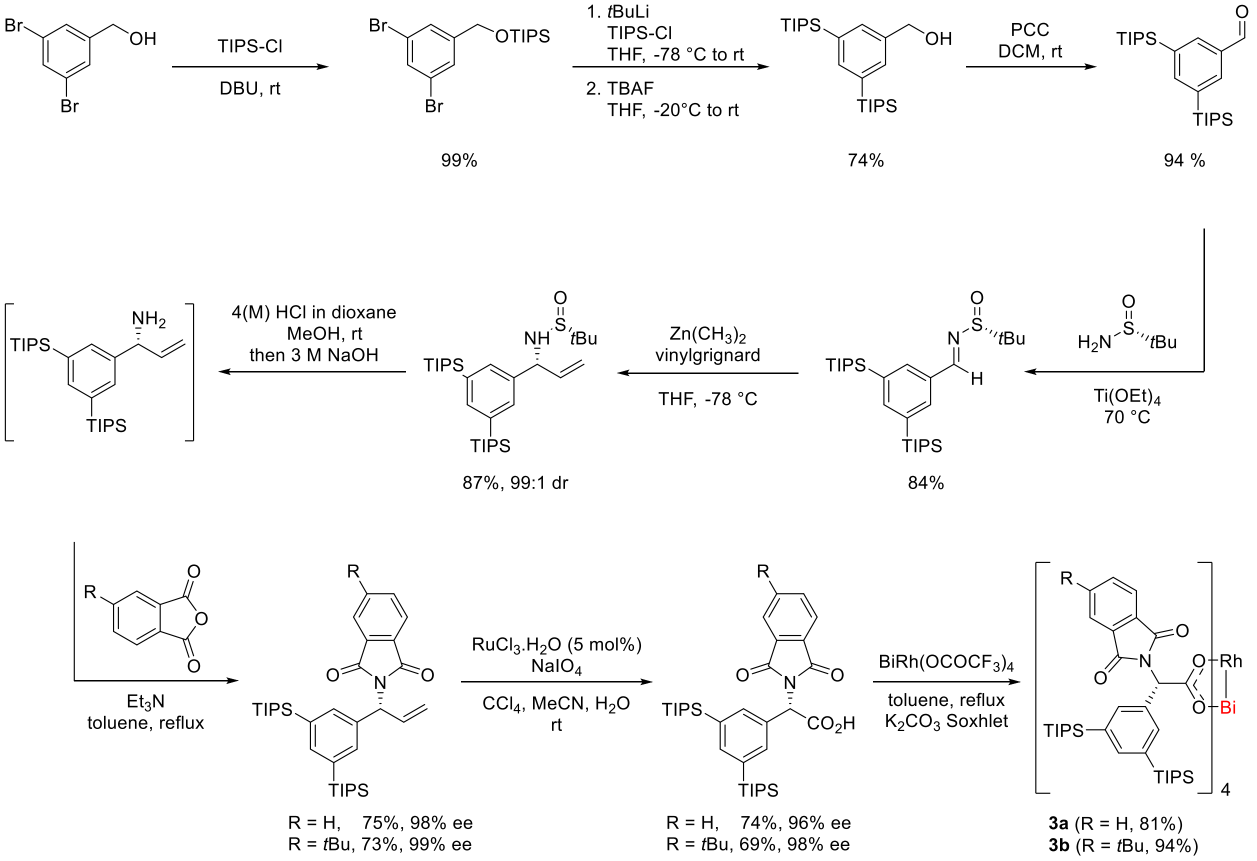

((3,5-Dibromobenzyl)oxy)triisopropylsilane (S1). A 250 mL round bottom Schlenk flask was charged with

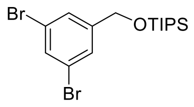

3,5-dibromobenzylalcohol (**6**) (3.00 g, 11.28 mmol), chlorotriisopropylsilane (2.9 mL, 13.54 mmol) and CH_2Cl_2 (50 mL). The solution was cooled to 0°C and DBU (2.2 mL, 14.67 mmol) was added dropwise. After the addition was complete, stirring was continued for 2 h at ambient temperature. The reaction was quenched with HCl (1 M, 30 mL) and the aqueous phase was extracted with CH_2Cl_2. The combined organic layers were dried over Na_2SO_4 and concentrated. Purification of the residue by flash chromatography using pentane as eluent yielded the title compound as a colorless oil (4.72 g, 99%). ^{1}H NMR (400 MHz, $CDCl_3$): δ = 7.53 (t, *J* = 1.8 Hz, 1H), 7.43 (dd, *J* = 1.9, 0.9 Hz, 2H), 4.77 (s, 2H), 1.24 – 1.12 (m, 3H), 1.09 (d, *J* = 6.7 Hz, 18H); ^{13}C NMR (101 MHz, $CDCl_3$): δ = 145.8, 132.4, 127.6, 122.9, 63.8, 18.1, 12.1; IR (ATR): ṽ = 2942, 2865, 1587, 1557, 1461, 1425, 1365, 1198, 1117, 1068, 995, 880, 846, 798, 738, 666, cm^{-1}; HRMS (ESI$^+$) for $C_{16}H_{27}OBr_2Si$ [M+H]$^+$: calcd: 421.01927, found: 421.01905.

(3,5-Bis(triisopropylsilyl)phenyl)methanol (7). A flame dried Schlenk flask was charged with ((3,5-dibromobenzyl)oxy)triisopropylsilane **(S1)** (1.013 g, 2.4 mmol) and THF (20 mL). The solution was cooled to −78°C before *tert*-buthylithium (1.6 M in pentane, 6.6 mL, 10.56 mmol) was added dropwise. After the addition was complete, the mixture was warmed to ambient temperature and stirring continued for 1 h. The solution was cooled to −20°C before chlorotriisopropylsilane (1.28 mL, 6.0 mmol) was added

dropwise. The resulting mixture was warmed to ambient temperature and stirring continued for another 24 h. The reaction was quenched with saturated aqueous NH_4Cl (10 mL) solution and the aqueous phase was extracted with methyl *tert*-butyl ether. The combined organic layers were dried over Na_2SO_4 and concentrated. Analysis of the crude reaction mixture indicated a 5:1 ratio of di- and mono-silylated product; this material was used directly in the next step.

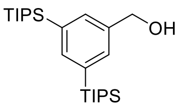

Tetrabutylammonium fluoride (1 M in THF, 2.4 mL, 2.4 mmol) was added dropwise to a solution of the crude product in THF (20 mL) at 0°C. The solution was warmed to ambient temperature and stirring was continued for 30 min. The reaction was quenched with saturated aqueous NH_4Cl solution (10 mL) and the aqueous phase was extracted with EtOAc. The combined organic layers were dried over Na_2SO_4 and concentrated. Purification of the residue by flash chromatography using 10% EtOAc in pentane as eluent yielded the title compound as a white solid after drying in high vacuum (750 mg, 74% over two steps). m.p. = 96-97°C; ^{1}H NMR (400 MHz, $CDCl_3$): δ = 7.56 (t, *J* = 1.3 Hz, 1H), 7.46 (d, *J* = 1.1 Hz, 2H), 4.69 (s, 2H), 1.41 (hept, *J* = 7.4 Hz, 6H), 1.07 (d, *J* = 7.5 Hz, 36H); ^{13}C NMR (101 MHz, $CDCl_3$): δ = 142.2, 138.6, 134.4, 133.8, 66.4, 18.7, 10.9; IR (ATR): ṽ = 3270, 2940, 2863, 1461, 1383, 1366, 1142, 1014. 994, 882, 788, 714 674, 643, 561, 497 cm^{-1}; HRMS (ESI$^+$) for $C_{25}H_{48}OSi_2Na$ $[M+Na]^+$: calcd: 443.31359, found: 443.31334.

3,5-Bis(triisopropylsilyl)benzaldehyde (S2). A round bottom flask was charged with (3,5-bis(triisopropylsilyl)phenyl)methanol **(7)** (740 mg, 1.758 mmol), silica gel, and CH_2Cl_2 (20 mL). PCC (568 mg, 2.637 mmol) was added to this suspension and the mixture was stirred for 2 h until TLC analysis indicated full conversion. The mixture was concentrated and the residue loaded on top of a silica-gel column, eluting the product-containing fractions with 2% Et_2O in pentane to give the title compound as a white solid (694 mg, 94%). m.p. = 49-50°C; ^{1}H NMR (400 MHz, $CDCl_3$): δ = 10.04 (s, 1H), 7.96 (d, *J* = 1.3 Hz, 2H), 7.89 (t, *J* = 1.3 Hz, 1H), 1.44 (h, *J* = 7.4 Hz, 6H), 1.08 (d, *J* = 7.5 Hz, 36H); ^{13}C NMR (101 MHz, $CDCl_3$): δ = 193.7, 148.6, 137.0, 135.0, 134.6, 18.6, 10.9; IR (ATR): ṽ = 2941, 2863, 1699, 1566, 1461, 1383, 1366, 1248, 1217, 1142, 1120, 1073, 1013, 994, 910, 881, 787, 701, 672, 644, 559, 511, 488 cm^{-1}; HRMS (ESI$^+$) for $C_{25}H_{46}OSi_2Na$ $[M+Na]^+$: calcd: 441.29794, found: 441.29786.

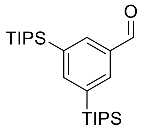

(*R*,*E*)-N-(3,5-Bis(triisopropylsilyl)benzylidene)-2-methylpropane-2-sulfinamide (8). A round bottom flask was charged with 3,5-bis(triisopropylsilyl) benzaldehyde **(S2)** (685 mg, 1.635 mmol), (*R*)-*tert*-butylsulfinamide (218 mg, 1.799 mmol), and THF (20 mL). $Ti(OEt)_4$ (514 µL, 2.45 mmol) was added and the resulting solution was stirred at 75°C for 6 h. The reaction was quenched with brine under vigorous stirring. The resulting suspension was filtered through a plug of Celite® and the filter cake was carefully rinsed with EtOAc. The combined filtrates were washed with brine. The brine layer was extracted once with EtOAc, and the combined organic phases were dried over Na_2SO_4, filtered and concentrated. Purification of the residue by flash chromatography using 5% EtOAc in pentane as eluent yielded the title compound as a white solid (815 mg, 95%). m.p. = 85-86°C; $[\alpha]_D^{20}$ = –19.3 (c = 1.2, $CHCl_3$); ^{1}H NMR (400 MHz, $CDCl_3$): δ = 8.60 (s, 1H), 7.95 (d, *J* = 1.2 Hz, 2H), 7.77 (t, *J* = 1.3 Hz, 1H), 1.42 (h, *J* = 7.5 Hz, 6H), 1.27 (s, 9H), 1.08 (dd, *J* = 7.5, 3.8 Hz, 36H); ^{13}C NMR (101 MHz, $CDCl_3$): δ = 163.7, 146.6, 136.7, 134.7, 132.3, 57.9, 22.8, 18.7, 18.6, 10.9; IR (ATR): ṽ = 2942, 2864, 1595, 1561, 1460, 1362, 1143, 1087, 1013, 995, 919, 878, 793, 750, 704,

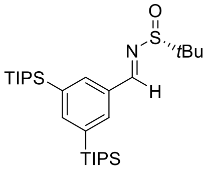

675, 643, 562, 500, 447 cm^{-1}; HRMS (ESI^{+}) for $C_{29}H_{55}NOSSi_2Na$ $[M+Na]^{+}$: calcd: 544.34351, found: 544.34338.

(*R*)-N-((*R*)-1-(3,5-Bis(triisopropylsilyl)phenyl)allyl)-2-methylpropane-2-sulfinamide (9). A flame dried Schlenk flask was charged with vinylmagnesium bromide (1 M in THF, 6.66 mL, 6.66 mmol) and dimethylzinc (1.2 M in toluene, 1.85 mL, 2.222 mmol) and the resulting solution was stirred at 0°C for 10 min. The mixture was cooled to –78°C before a solution of compound **8** (2.32 g, 4.44 mmol) in THF (15 mL) was added via syringe pump over a period of 60 min. Stirring was continued for 6 h at −78°C before the reaction was quenched with saturated aqueous NH_4Cl solution at −78°C. The aqueous phase was extracted with EtOAc, and the combined organic layers were dried over Na_2SO_4 and concentrated. NMR analysis of the crude mixture indicated a diastereomeric ratio of ≈99:1. Purification of the residue by flash chromatography using 20% EtOAc in pentane as eluent yielded the title compound as a white solid (2.14 g, 87%). m.p. =109-110°C; $[\alpha]_D^{20}$ = –40.5 (c = 1.65, $CHCl_3$); ^1H NMR (400 MHz, $CDCl_3$): δ = 7.47 (t, *J* = 1.2 Hz, 1H), 7.36 (d, *J* = 1.2 Hz, 2H), 5.84 (ddd, *J* = 17.0, 10.1, 7.8 Hz, 1H), 5.28 (dt, *J* = 17.0, 1.1 Hz, 1H), 5.14 (dt, *J* = 10.1, 1.0 Hz, 1H), 4.89 (d, *J* = 7.8 Hz, 1H), 1.31 (hept, *J* = 7.0 Hz, 6H), 1.18 (s, 9H), 0.99 (dd, *J* = 7.5, 2.4 Hz, 36H); ^{13}C NMR (101 MHz, $CDCl_3$): δ = 142.2, 139.4, 138.8, 134.2, 134.1, 117.5, 61.7, 55.7, 22.8, 18.7, 18.7, 10.9; IR (ATR): ṽ = 3712, 2941, 2864, 1462, 1369, 1145, 1048, 1015, 993, 918, 881, 788, 673, 645, 561, 496 cm^{-1}; HRMS (ESI^{+}) for $C_{31}H_{59}NOSSi_2Na$ $[M+Na]^{+}$: calcd: 572.37481, found: 572.37492.

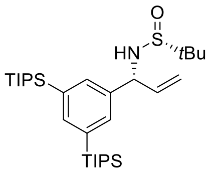

(*R*)-2-(1-(3,5-Bis(triisopropylsilyl)phenyl)allyl)isoindoline-1,3-dione (10a). HCl (4 M in dioxane, 0.56 mL, 2.22 mmol) was added at 0°C to a solution of compound **9** (407 mg, 0.74 mmol) in methanol (HPLC grade, 10 mL). The reaction flask was capped with a rubber septum and the mixture was stirred under air at room temperature for 1 h. The mixture was concentrated under vacuum. Water (20 mL) and CH_2Cl_2 (20 mL) were added to the residue and the pH of the aqueous phase was adjusted to pH ≈ 10 by addition of NaOH (3 M) before it was extracted with CH_2Cl_2 (3 x 20 mL). The combined organic layers were dried over Na_2SO_4 and the solvent was removed in vacuum to give (*R*)-1-(3,5-bis(triisopropylsilyl)phenyl)prop-2-en-1-amine, which was directly used in the next step.

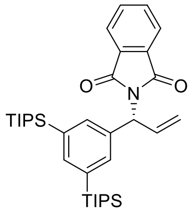

A solution of the crude amine, phthalic anhydride (120 mg, 0.814 mmol) and Et_3N (103 µL, 0.74 mmol) in toluene (20 mL) was stirred at reflux temperature for 36 h, using a Dean-Stark apparatus to collect the released water. Evaporation of the solvent and purification of the residue by flash chromatography using 3% Et_2O in pentane as eluent afforded the title compound as a colorless sticky solid (320 mg, 75% over two steps). m.p. = 86-89°C, $[\alpha]_D^{20}$ = –10.1 (c = 1.07, $CHCl_3$); ^1H NMR (400 MHz, $CDCl_3$): δ = 7.83 (dd, *J* = 5.5, 3.0 Hz, 2H), 7.69 (dd, *J* = 5.5, 3.1 Hz, 2H), 7.54 (d, *J* = 1.1 Hz, 2H), 7.52 (d, *J* = 1.2 Hz, 1H), 6.65 (ddd, *J* = 17.3, 10.2, 7.3 Hz, 1H), 5.96 (d, *J* = 7.2 Hz, 1H), 5.41 – 5.25 (m, 2H), 1.36 (hept, *J* = 7.5 Hz, 6H), 1.03 (dd, *J* = 7.5, 2.4 Hz, 36H); ^{13}C NMR (101 MHz, $CDCl_3$): δ = 167.9, 141.8, 136.3, 135.1, 134.9, 134.0, 133.7, 132.2, 123.3, 118.8, 57.6, 18.7, 18.7, 10.9; IR (ATR): ṽ = 2942, 2864, 1714, 1463, 1377, 1348, 1082,1015, 994, 881, 789, 714, 675, 641, 562, 502 cm^{-1}; HRMS (ESI^{+}) for $C_{35}H_{53}NO_2Si_2Na$ $[M+Na]^{+}$: calcd: 598.35071, found: 598.35162. The product had an *ee* of 98%. [The *ee* was determined by HPLC analysis: 150 mm Chiralpak IB-N-3, 3 µm, ∅ 4.6 mm, *n*-heptane/i-propanol = 99.9:0.1, v = 1.0 mL/min, λ = 220 nm].

2-(3,5-Bis(triisopropylsilyl)phenyl)-2-(1,3-dioxoisoindolin-2-yl)acetic acid (11a). A round bottom flask

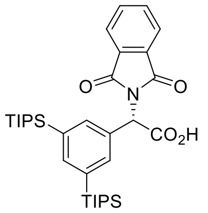

containing a magnetic stir-bar was charged with 2-(1-(3,5-bis(triisopropylsilyl)phenyl)allyl)isoindoline-1,3-dione **(10a)** (308 mg, 0.535 mmol), sodium metaperiodate (571 mg, 2.674 mmol), water (3 mL), acetonitrile (2 mL) and carbon tetrachloride (2 mL). Ruthenium trichloride hydrate (5.5 mg, 0.026 mmol, 5 mol%) was added to this biphasic mixture, which was vigorously stirred for 12 h at ambient temperature. The mixture was diluted with CH_2Cl_2 (10 mL) and the phases were separated. The aqueous layer was extracted with CH_2Cl_2 (3 x 20 mL), the combined extracts were dried over Na_2SO_4, filtered through a Celite® pad and the filtrate was concentrated. The crude product was purified by flash chromatography using 2% MeOH in CH_2Cl_2 as eluent to afford the title compound as a colorless solid (235 mg, 74%). m.p. = 155-160°C; $[\alpha]_D^{20}$ = –0.4 (c = 0.9, $CHCl_3$); ^1H NMR (400 MHz, $CDCl_3$): δ = 7.86 (dd, *J* = 5.5, 3.1 Hz, 2H), 7.71 (dd, *J* = 5.5, 3.0 Hz, 2H), 7.67 (d, *J* = 1.1 Hz, 2H), 7.57 (s, 1H), 6.07 (s, 1H), 1.39 (p, *J* = 7.4 Hz, 6H), 1.04 (d, *J* = 7.5 Hz, 36H); ^{13}C NMR (101 MHz, $CDCl_3$): δ = 173.1, 167.1, 142.7, 137.0, 134.3, 133.9, 132.0, 132.0, 123.8, 56.4, 18.6, 18.6, 10.9; IR (ATR): ṽ = 2942, 2864, 1776, 1713, 1464, 1382, 1253, 1234, 1106, 1015, 964, 909, 882, 792, 724, 680, 670, 641, 563, 528, 505 cm^{-1}; HRMS (ESI^+) for $C_{34}H_{51}NO_4Si_2Na$ $[M+Na]^+$: calcd: 616.32489, found: 616.32568. The optical purity was 96% *ee* as determined by HPLC analysis: 150 mm Chiralpak IA-3, 3 µm, ∅ 4.6 mm, *n*-heptane/2-propanol/TFA = 95/5/0.05, v = 1.0 mL/min, λ = 220 nm.

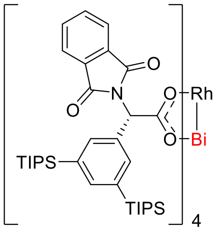

Complex 3a. A mixture of [BiRh($OCOCF_3$)$_4$] (55.3 mg, 0.072 mmol) and 2(*S*)-(3,5-bis(triisopropylsilyl)phenyl)-2-(1,3-dioxoisoindolin-2-yl)acetic acid **(11a)** (215 mg, 0.362 mmol, 96% *ee*) in dry toluene (25 mL) was stirred at reflux temperature for 3 h, passing the condensed vapor through a Soxhlet apparatus filled with K_2CO_3; at this point, ligand exchange was complete as judged by ^{19}F NMR. The mixture was concentrated in vacuum and the residue was purified by flash chromatography using 4% EtOAc in CH_2Cl_2 as eluent to give the title complex as a yellow solid (157 mg, 81%). $[\alpha]_D^{20}$ = 80.8 (c = 1.2, $CHCl_3$); ^1H NMR (400 MHz, $CDCl_3$): δ = 7.88 – 7.81 (m, 8H), 7.65 (s, 8H), 7.63 – 7.57 (m, 8H), 7.49 (s, 4H), 6.38 (s, 4H), 1.41 – 1.25 (m, 24H), 0.98 (dd, *J* = 7.5, 5.3 Hz, 144H); ^{13}C NMR (101 MHz, $CDCl_3$): δ = 181.9, 166.6, 142.0, 137.7, 134.1, 133.7, 132.7, 132.3, 123.5, 57.8, 18.6, 18.6, 10.8; IR (ATR): ṽ = 2942, 2863, 1772, 1715, 1463, 1376, 1333, 1144, 1106, 1015, 993, 881, 806, 782, 738, 724, 712, 675, 643, 561 cm^{-1}; HRMS (ESI^+) for $C_{136}H_{200}BiN_4O_{16}Si_8RhNa$ $[M+ Na]^+$: calcd: 2704.18646, found: 2704.18946.

(*R*)-2-(1-(3,5-Bis(triisopropylsilyl)phenyl)allyl)-5-(*tert*-butyl)isoindoline-1,3-dione (10b). HCl (4 M in

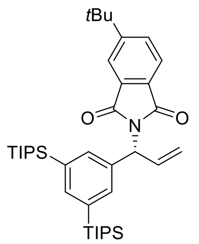

dioxane, 1.68 mL, 6.729 mmol) was added at 0°C under air to a solution of compound **9** (1.234 g, 2.243 mmol) in methanol (HPLC-grade, 20 mL). The flask was capped with a rubber septum and the solution stirred at room temperature for 1 h. The mixture was concentrated under vacuum. Water (20 mL) and CH_2Cl_2 (20 mL) were added to the residue and the pH of the aqueous phase was adjusted to pH ≈ 10 upon addition of aqueous NaOH (3 M) before it was extracted with CH_2Cl_2 (3 x 20 mL). The combined organic layers were dried over Na_2SO_4 and the solvent was removed in vacuum to give (*R*)-1-(3,5-bis(triisopropylsilyl)phenyl)prop-2-en-1-amine, which was used directly in the next step.

5-(*tert*-Butyl)isobenzofuran-1,3-dione (503 mg, 2.467 mmol) and Et_3N (312 µL, 2.243 mmol) were added to the crude amine in toluene (20 mL) and the resulting mixture was stirred at reflux temperature for 36 h while the released water was collected in a Dean-Stark apparatus. Evaporation of the solvent and purification of the residue by flash chromatography using 3% Et_2O in pentane as eluent afforded the title compound as a colorless waxy solid (1.03 g, 73% over two steps). ^{1}H NMR (400 MHz, $CDCl_3$): δ = 7.86 (dd, *J* = 1.7, 0.7 Hz, 1H), 7.75 (dd, *J* = 7.9, 0.7 Hz, 1H), 7.71 (dd, *J* = 7.9, 1.7 Hz, 1H), 7.57 – 7.54 (m, 2H), 7.51 (d, *J* = 1.2 Hz, 1H), 6.65 (ddd, *J* = 17.3, 10.2, 7.3 Hz, 1H), 6.00 – 5.82 (m, 1H), 5.39 – 5.24 (m, 2H), 1.44 – 1.29 (m, 15H), 1.03 (dd, *J* = 7.5, 2.3 Hz, 36H); ^{13}C NMR (101 MHz, $CDCl_3$): δ = 168.3, 167.9, 158.6, 141.8, 136.5, 135.2, 135.1, 133.6, 132.4, 131.0, 129.5, 123.2, 120.5, 118.6, 57.5, 35.9, 31.3, 18.7, 18.6, 10.9; IR (ATR): ṽ = 2942, 2864, 1771, 1714, 1620, 1462, 1369, 1326, 1255, 1135, 1087, 994, 922, 880, 790, 753, 675, 642, 563, 502 cm^{-1}; HRMS (ESI$^+$) for $C_{39}H_{61}NO_2Si_2Na$ [M+Na]$^+$: calcd: 654.41331, found: 654.41370. The product was obtained in 98% *ee* [The *ee* was determined by HPLC analysis: 150 mm Chiralpak IB-N-3, 3 µm, ∅ 4.6 mm, *n*-heptan/2-propanol = 99.9/0.1, v = 1.0 mL/min, 298 K, λ = 220 nm].

(*S*)-2-(3,5-Bis(triisopropylsilyl)phenyl)-2-(5-(*tert*-butyl)-1,3-dioxoisoindolin-2-yl)acetic acid (11b). A round bottom flask containing a magnetic stir-bar was charged with compound **10b** (830 mg, 1.313 mmol), sodium metaperiodate (1.404 g, 6.57 mmol), water (9 mL), acetonitrile (6 mL), and carbon tetrachloride (6 mL). Ruthenium trichloride hydrate (13.6 mg, 5 mol%) was added and the biphasic mixture was vigorously stirred for 12 h at ambient temperature. The mixture was diluted with CH_2Cl_2 (20 mL), the phases were separated, and the aqueous layer repeatedly extracted with CH_2Cl_2. The combined extracts were dried over Na_2SO_4, filtered through a Celite® pad and the filtrate was concentrated. The crude product was purified by flash chromatography using 2% MeOH in CH_2Cl_2 as eluent to afford the title compound as a colorless solid (590 mg, 69%). m.p. = 117-119°C; $[\alpha]_D^{20}$ = 12.0 (c = 1.8, $CHCl_3$); ^{1}H NMR (400 MHz, $CDCl_3$): δ = 7.81 (d, *J* = 1.5 Hz, 1H), 7.70 (d, *J* = 7.9 Hz, 1H), 7.64 (dd, *J* = 8.0, 1.7 Hz, 1H), 7.59 (d, *J* = 1.2 Hz, 2H), 7.49 (d, *J* = 1.3 Hz, 1H), 5.97 (s, 1H), 1.38 – 1.24 (m, 15H), 0.96 (d, *J* = 7.5 Hz, 36H); ^{13}C NMR (101 MHz, $CDCl_3$): δ = 173.4, 167.6, 167.2, 159.0, 142.6, 137.0, 137.0, 133.8, 132.2, 131.3, 129.3, 123.6, 120.9, 56.4, 35.9, 31.3, 18.7, 18.6, 10.9; IR (ATR): ṽ = 2942, 2864, 1776, 1715, 1462, 1371, 1254, 1138, 1103, 1015, 994, 881, 790, 753, 676, 642, 563, 500 cm^{-1}; HRMS (ESI$^+$) for $C_{38}H_{59}NO_4Si_2Na$ [M+Na]$^+$: calcd: 672.38749, found: 672.38859. The optical purity (98% *ee*) was determined by HPLC analysis: Chiralpak IB-N-3, 3 µm, ∅ 4.6 mm, *n*-heptane/2-propanol/TFA = 99/1/0.05, v = 1.0 mL/min, λ = 220 nm.

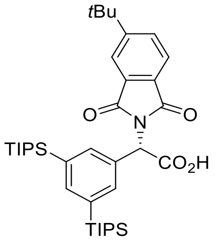

Complex 3b. A solution of [BiRh($OCOCF_3$)$_4$] (72 mg, 0.094 mmol) and (*S*)-2-(3,5-bis(triisopropylsilyl)-phenyl)-2-(5-(*tert*-butyl)-1,3-dioxoisoindolin-2-yl)acetic acid **(11b)** (306 mg, 0.471 mmol; 98% *ee*) in toluene (25 mL) was stirred at reflux temperature for 3 h, passing the condensed vapors through a Soxhlet apparatus filled with K_2CO_3. The progress of the reaction was monitored by ^{19}F NMR, which indicated complete conversion of the starting complex after 3 h. The mixture was concentrated in vacuum and the residue was purified by flash chromatography using 60% CH_2Cl_2 in pentane as eluent to furnish the desired complex as a yellow solid (258 mg, 94%). NMR spectra were recorded at 80°C; at lower temperature only very broad signals with poor resolution were observed. $[\alpha]_D^{20}$ = 80.2 (c = 1.2, $CHCl_3$); ^{1}H NMR (600 MHz, $CDCl_3$, 353K): δ = 7.90 (s, 4H), 7.78 – 7.72

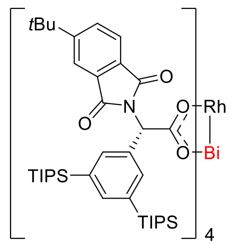

(m, 12H), 7.62 (dd, *J* = 7.9, 1.7 Hz, 4H), 7.56 (d, *J* = 1.3 Hz, 4H), 6.38 (s, 4H), 1.42 – 1.33 (m, 60H), 1.04 (dd, *J* = 7.5, 5.7 Hz, 144H); ^{13}C NMR (151 MHz, $CDCl_3$, 353K): δ = 182.1, 167.0, 166.7, 158.3, 142.1, 138.0, 134.6, 133.3, 133.0, 130.5, 130.1, 123.3, 120.7, 58.0, 35.9, 31.4, 18.9, 11.2; IR (ATR): ṽ = 2942, 2864, 1774, 1714, 1462, 1368, 1329, 1104, 1014, 881, 807, 783, 756, 643, 564, 509 cm^{-1}; HRMS (ESI$^+$) for $C_{152}H_{232}BiN_4O_{16}RhSi_8Na$ [M+ Na]$^+$: calcd: 2928.43686, found: 2928.43753.

Control Experiment with the Catalyst Lacking the Peripheral TIPS-Groups: [BiRh(*S*)-PTPG)$_4$]

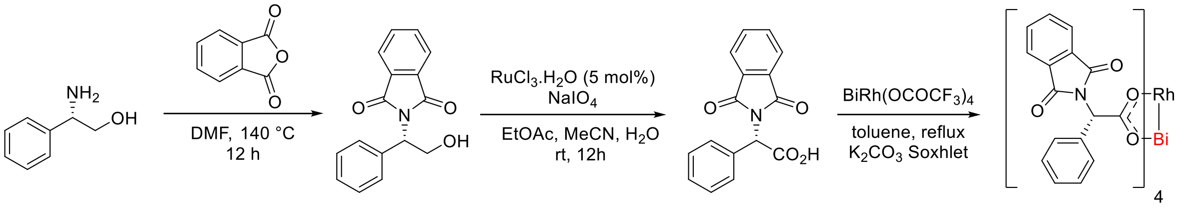

BiRh(*S*-PTPG)$_4$, 97%

2(*S*)-2-(1,3-Dioxoisoindolin-2-yl)-2-phenylacetic acid was synthesized in a two steps sequence described below. Characterization data matched with the literature reported data.[9]

A mixture of (*S*)-2-amino-2-phenylethan-1-ol (1018 mg, 7.420 mmol) and phthalic anhydride (1099 mg, 7.420 mmol) was stirred at 140 °C for 12 h. After cooling, water was added and the product was extracted with EtOAc. The combined organic layers were dried over Na_2SO_4 and the solvent was evaporated to give crude (*S*)-2-(2-hydroxy-1-phenylethyl)isoindoline-1,3-dione which was used directly in the next step.

Crude (*S*)-2-(2-hydroxy-1-phenylethyl)isoindoline-1,3-dione thus obtained was dissolved in EtOAc (30 mL) and CH_3CN (30 mL). A solution of $NaIO_4$ (6.51 g, 30.421 mmol) in water (45 mL) and ruthenium trichloride hydrate (33.8 mg, 2.2 mol%) were added and the mixture was vigorously stirred for 12 h. The mixture was then diluted with EtOAc and water and extracted with EtOAc. The combined organic layers were dried over Na_2SO_4 and the solvent was evaporated. Purification opf the residue by flash chromatography using 5% MeOH in CH_2Cl_2 as eluent gave the desired product as a white solid (1.30 mg, 62% yield).

Complex BiRh(*S*-PTPG)$_4$ (S3). A mixture of [BiRh($OCOCF_3$)$_4$] (54.3 mg, 0.071 mmol) and 2(*S*)-2-(1,3-dioxoisoindolin-2-yl)-2-phenylacetic acid (100 mg, 0.356 mmol) in dry toluene (25 mL) was stirred at reflux temperature for 3 h, passing the condensed vapor through a Soxhlet apparatus filled with K_2CO_3; at this point, ligand exchange was complete as judged by ^{19}F NMR. The mixture was concentrated in vacuum and the residue was purified by flash chromatography using 2% MeOH in CH_2Cl_2 as eluent to give the title complex as a yellow solid (99 mg, 97%). ^{1}H NMR (400 MHz, $CDCl_3$): δ = 7.80 (dd, *J* = 5.38, 3.04 Hz, 8H), 7.59 (dd, *J* = 5.45, 2.83 Hz, 8H), 7.55 – 7.50 (m, 8H), 7.32 – 7.14 (m, 12H), 6.23 (s, 4H); ^{13}C NMR (101 MHz, $CDCl_3$): δ = 182.5, 167.1, 136.4, 134.0, 132.1, 130.0, 128.4, 128.2, 123.6, 57.4; IR (ATR): ṽ = 1771, 1712, 1644, 1597, 1467, 1377, 1333, 1260, 1107, 1075, 954, 910, 892, 716, 696, 648, 530 cm^{-1}; HRMS (ESI$^+$) for $C_{64}H_{40}Bi_1N_4O_{16}Rh_1Na_1$ [M + Na]$^+$: calcd: 1455.11904, found: 1455.12001.

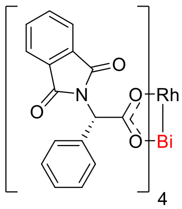

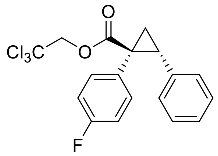

Using this complex, cyclopropane **5b’** was obtained at RT in 94% yield and 24% ee.

Diazo Compounds

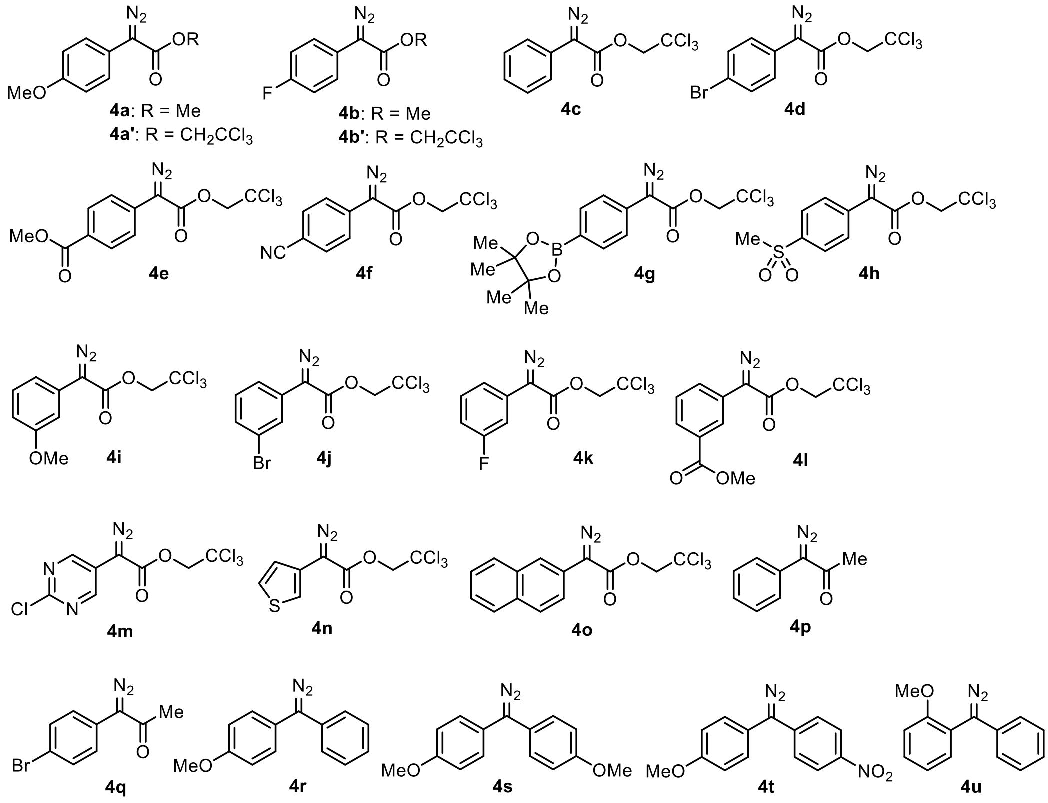

Diazo compounds **4a**, **4b** are known compounds and were prepared according to the literature procedure.[10] Characterization data matched with the reported data.

Diazo compounds **4a’**, **4b’**, **4c**, **4d** are known compounds and were prepared according to the literature procedure.[11] Characterization data matched with the reported data.

Diazo compounds **4e**, **4g**, **4i**, **4j**, **4m, 4o** are known compounds and were prepared according to the literature procedure.[12] Characterization data matched with the reported data.

Diazo compounds **4p**, **4q** are known compounds and were prepared according to the literature procedure.[13] Characterization data matched with the reported data.

Diazo compounds **4h** was prepared by adapting a literature procedure.[11] Characterization data is given below.

Diazo compounds **4f**, **4k**, **4l** were prepared by adapting a literature procedure.[12] Characterization data is given below.

Diazo compounds **4r**, **4t** are known compounds and were prepared according to the literature procedure.[14] Characterization data matched with the reported data.

Diazo compound **4s** is a known compounds and was prepared according to the literature procedure.[15] Characterization data matched with the reported data.

Diazo compound **4u** is a known compound and was prepared according to the literature procedure.[16] Characterization data matched with the reported data.

2,2,2-Trichloroethyl 2-(4-cyanophenyl)-2-diazoacetate (4f). Prepared from 4-iodobenzonitrile (962 mg, 4.43 mmol) as a yellow solid (735 mg, 69%). ^{1}H NMR (400 MHz, $CDCl_3$): δ = 7.72 – 7.59 (m, 4H), 4.93 (s, 2H); ^{13}C NMR (101 MHz, $CDCl_3$): δ = 162.3, 132.9, 130.8, 123.7, 118.7, 109.5, 94.9, 74.1 (C-N_2 was not detected); IR (ATR): ṽ = 2961, 2220, 2099, 1714, 1602, 1506, 1377, 1343, 1237, 1184, 1136, 1084, 1037, 926, 838, 827, 793, 731, 708, 576, 551, 496; HRMS (ESI$^+$) for $C_{11}H_6Cl_3N_3O_2Na$ [M+Na$^+$]$^+$: calcd: 339.94178, found: 339.94172.

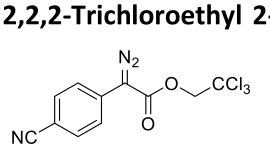

2,2,2-Trichloroethyl 2-diazo-2-(4-(methylsulfonyl)phenyl)acetate (4h). Prepared from 2-(4-(methylsulfonyl)phenyl)acetic acid (1.52 g, 7.12 mmol) as a yellow solid (560 mg, 21%). ^{1}H NMR (400 MHz, $CDCl_3$): δ = 8.00 – 7.90 (m, 2H), 7.75 – 7.67 (m, 2H), 4.94 (s, 2H), 3.06 (s, 3H); ^{13}C NMR (101 MHz, $CDCl_3$): δ = 162.3, 137.7, 131.9, 128.3, 123.8, 94.9, 74.2, 44.7 (C=N_2 was not detected); IR (ATR): ṽ = 2926, 2096, 1702, 1499, 1337, 1304, 1285, 1237, 1147, 1092, 1033, 958, 787, 769, 734, 715, 564, 521, 503; HRMS (ESI$^+$) for $C_{11}H_9N_2O_4SCl_3Na$ [M+Na$^+$]$^+$: calcd: 392.92408, found: 392.92450.

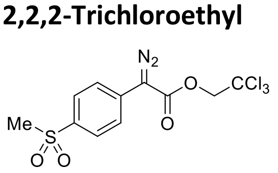

2,2,2-Trichloroethyl 2-diazo-2-(3-fluorophenyl)acetate (4k). Prepared from 1-fluoro-3-iodobenzene (945 mg, 4.26 mmol) as a yellow solid (1.06 g, 80%). ^{1}H NMR (400 MHz, $CDCl_3$): δ = 7.42 – 7.29 (m, 2H), 7.19 (ddd, *J* = 8.0, 1.9, 0.9 Hz, 1H), 6.91 (tdd, *J* = 8.3, 2.5, 0.9 Hz, 1H), 4.92 (s, 2H); ^{13}C NMR (101 MHz, $CDCl_3$): δ = 164.6, 162.6 (d, *J* = 79.2 Hz), 130.7 (d, *J* = 9.0 Hz), 127.3 (d, *J* = 9.4 Hz), 119.1 (d, *J* = 3.0 Hz), 113.2 (d, *J* = 21.5 Hz), 111.4 (d, *J* = 25.3 Hz), 95.1, 74.1, (C=N_2 was not detected); ^{19}F NMR (282 MHz, $CDCl_3$): δ = –111.4; IR (ATR): ṽ = 3019, 2093, 1701, 1611, 1580, 1493, 1445, 1383, 1351, 1231, 1211, 1170, 1130, 1090, 1049, 890, 874, 815, 774, 730, 709, 680, 577, 499; HRMS (EI) for $C_{10}H_6N_2O_2Cl_3F$ [M]$^+$: calcd: 309.94734, found: 309.94710.

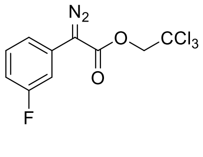

Methyl 3-(1-diazo-2-oxo-2-(2,2,2-trichloroethoxy)ethyl)benzoate (4l). Prepared from methyl 3-iodobenzoate (806 mg, 3.08 mmol) as a yellow solid (980 mg, 91%). ^{1}H NMR (400 MHz, $CDCl_3$): δ = 8.10 (t, *J* = 1.9 Hz, 1H), 7.89 (dt, *J* = 7.7, 1.3 Hz, 1H), 7.77 (ddd, *J* = 8.0, 2.1, 1.1 Hz, 1H), 7.53 – 7.45 (m, 1H), 4.93 (s, 2H), 3.93 (s, 3H); ^{13}C NMR (101 MHz, $CDCl_3$): δ = 166.6, 163.2, 131.2, 129.4, 128.5, 127.5, 125.6, 124.8, 95.1, 74.1, 52.5 (C-N_2 was not detected); IR (ATR): ṽ = 2947, 2865, 2085, 1710, 1385, 1244, 1155, 1052, 783, 751, 707, 676, 581; HRMS (ESI$^+$) for $C_{12}H_9N_2O_4Cl_3Na$ [M+Na$^+$]$^+$: calcd: 372.95201, found: 372.95244.

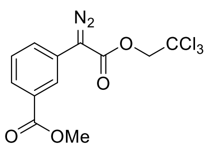

Cyclopropanes and Cyclopropenes

General procedure (Small Scale). An oven dried jacketed Schlenk flask equipped with a magnetic stir bar was charged with the [BiRh] catalyst (0.001 mmol, 1 mol%) under argon. The alkene or alkyne substrate (0.5 mmol) and pentane (1 mL) were added and the resulting solution cooled to −10°C. A solution of the diazo compound (0.1 mmol) in pentane (3 mL) was added dropwise over 10 min. The resulting mixture was stirred at −10°C until TLC analysis indicated the complete consumption of the diazo compound. For work up, the mixture was absorbed on silica, which was loaded on top of a silica column. Purification by flash chromatography (*n*-pentane/Et_2O or hexanes/EtOAc) afforded the desired cyclopropane or cyclpropene product.

Gram Scale Reaction with Reduced Catalyst Loading. 2,2,2-Trichloroethyl (1*S*,2*R*)-1-(4-bromophenyl)-2-phenylcyclopropane-1-carboxylate (S4).

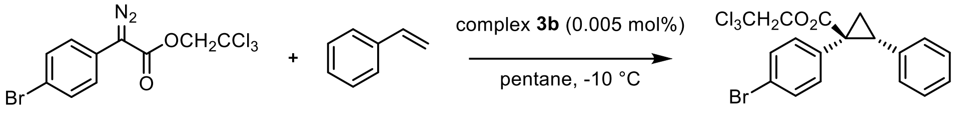

4d 5.0 equiv. **S4** (1.31 g, 97%, 98% ee)

An oven dried jacketed Schlenk flask equipped with a magnetic stir bar was charged under argon with complex **3b** (750 µL of 0.2 mmol/L stock solution in pentane, 0.005 mol%), styrene (1.73 mL, 15 mmol) and pentane (5 mL). The resulting solution was cooled to −10°C before a solution of 2,2,2-trichloroethyl 2-diazo-2-(4-bromophenyl)acetate (**4d**) (1.12 g, 3 mmol) in pentane (20 mL) was added dropwise over 30 min. Once the addition was complete, stirring was continued at −10°C for 24 h. The mixture was concentrated in the presence of silica and the loaded silica then added on top of a column of silica gel. The product was eluted with *n*-pentane/Et_2O to give the title compound as a colorless solid (1.31 g, 97%, 98% *ee*). m.p. = 87-89°C; $[\alpha]_D^{20}$ = –4.3 (c = 4.0, $CHCl_3$); ^1H NMR (400 MHz, $CDCl_3$): δ = 7.22 – 7.15 (m, 2H), 7.06 – 6.98 (m, 3H), 6.91 – 6.82 (m, 2H), 6.73 (dd, *J* = 6.8, 2.9 Hz, 2H), 4.75 (d, *J* = 11.9 Hz, 1H), 4.57 (d, *J* = 11.9 Hz, 1H), 3.14 (dd, *J* = 9.4, 7.5 Hz, 1H), 2.21 (dd, *J* = 9.4, 5.2 Hz, 1H), 1.89 (dd, *J* = 7.5, 5.2 Hz, 1H); ^{13}C NMR (101 MHz, $CDCl_3$): δ = 171.7, 135.3, 133.8, 133.1, 131.0, 128.2, 128.2, 127.0, 121.7, 95.1, 74.5, 36.7, 34.1, 20.3; IR (ATR): ṽ = 1731, 1488, 1428, 1363, 1232, 1206, 1149, 1094, 1051, 1009, 969, 804, 804, 770, 710, 695, 575, 549, 500 cm^{-1}; HRMS (ESI^+) for $C_{18}H_{14}Cl_3BrO_2Na$ $[M+Na^+]^+$: calcd: 468.91351, found: 468.91346.

The optical purity (98% *ee*) was determined by HPLC analysis: Daicel 150 mm Chiralpak IA-3, ∅ 4.6 mm, *n*-heptane/i-propanol = 98/2, v = 1.0 mL/min, λ = 220 nm, t(minor) = 3.45 min, t(major) = 4.04 min.]

When the same compound was prepared according to the general procedure with catalyst **2a**, a yield of 91% and an optical purity of 91% *ee* was obtained.

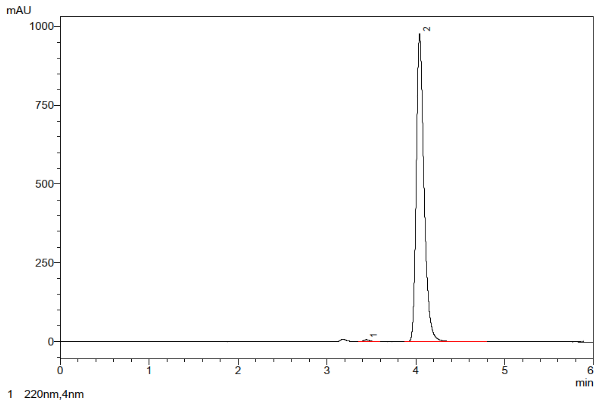

1 220nm,4nm

PDA Ch1 220nm

Peak #	Ret. Time	Area %
1	3.45	0.60
2	4.04	99.40
Total		100.00

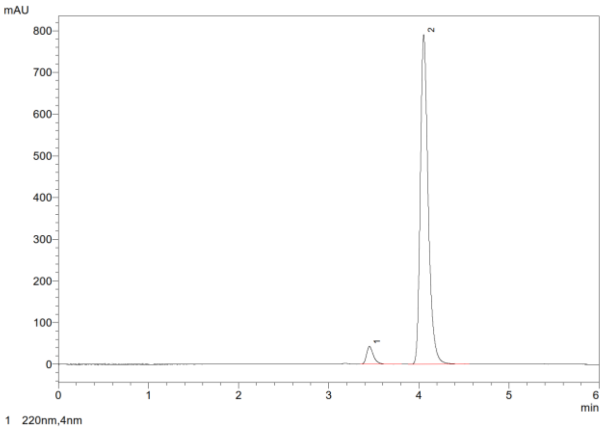

1 220nm,4nm

PDA Ch1 220nm

Peak #	Ret. Time	Area %
1	3.45	4.59
2	4.05	95.41
Total		100.00

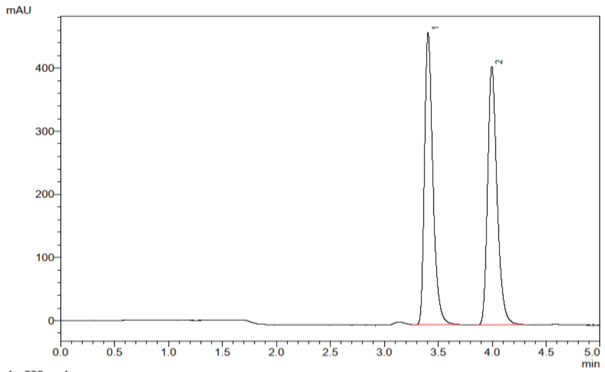

1 220nm,4nm

PDA Ch1 220nm

Peak #	Ret. Time	Area %
1	3.41	49.92
2	4.00	50.08
Total		100.00

Figure S12. HPLC traces of compound **S4**: with complex **3b** (top, left); with complex **2a** (top, right); the corresponding racemate (bottom).

The following compounds were prepared analogously

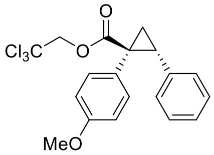

2,2,2-Trichloroethyl (1*S*,2*R*)-1-(4-methoxyphenyl)-2-phenylcyclopropane-1-carboxylate (5a'). Prepared according to the general procedure as a colorless oil; with complex **2a**: 90% yield, 98% *ee*; with complex **3b**: 99% yield, >99% *ee*. [The *ee* was determined by HPLC analysis: Daicel 150 mm Chiralpak IA-3, ∅ 4.6 mm, *n*-heptane/i-propanol = 98/2, v = 1.0 mL/min, λ = 220 nm, t(minor) = 4.00 min, t(major) = 4.51 min.] $[\alpha]_D^{20}$ = –0.45 (c = 3.8, $CHCl_3$); ^1H NMR (400 MHz, $CDCl_3$): δ = 7.09 (dd, *J* = 5.0, 1.9 Hz, 3H), 7.02 – 6.93 (m, 2H), 6.84 – 6.75 (m, 2H), 6.70 – 6.62 (m, 2H), 4.83 (d, *J* = 11.9 Hz, 1H), 4.65 (d, *J* = 11.9 Hz, 1H), 3.72 (s, 3H), 3.18 (dd, *J* = 9.4, 7.4 Hz, 1H), 2.26 (dd, *J* = 9.4, 5.0 Hz, 1H), 1.95 (dd, *J* = 7.4, 5.0 Hz, 1H); ^{13}C NMR (101 MHz, $CDCl_3$): δ = 172.5, 158.8, 136.0, 133.2, 128.3, 128.0, 126.7, 125.9, 113.3, 95.3, 74.5, 55.2, 36.7, 34.0, 20.6; IR (ATR): ṽ = 1730, 1612, 1515, 1456, 1441, 1294, 1239, 1209, 1177, 1149, 1109, 1094, 1052, 1032, 970, 831, 797, 770, 744, 712, 695, 613, 572, 553 cm^{-1}; HRMS (ESI^+) for $C_{19}H_{18}Cl_3O_3$ $[M+H]^+$: calcd: 399.03160, found: 399.03160.

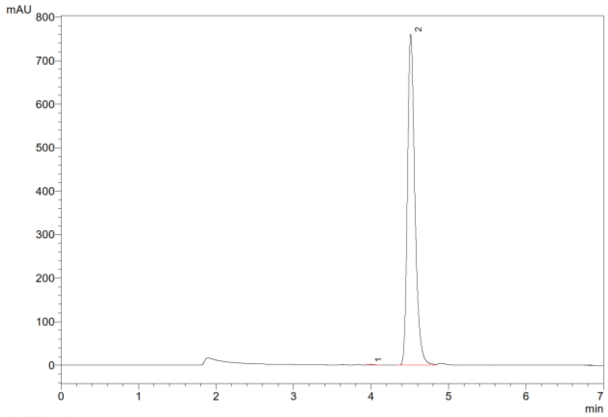

1 220nm,4nm

PDA Ch1 220nm

Peak #	Ret. Time	Area %
1	4.00	0.11
2	4.51	99.89
Total		100.00

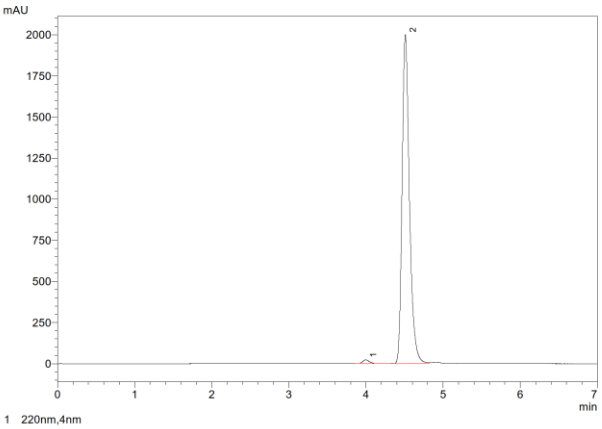

1 220nm,4nm

PDA Ch1 220nm

Peak #	Ret. Time	Area %
1	4.00	1.09
2	4.51	98.91
Total		100.00

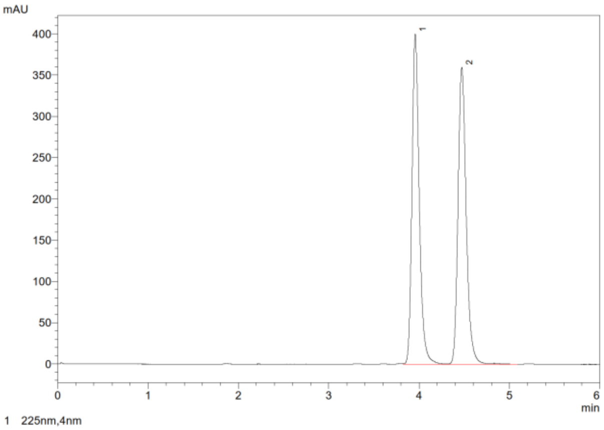

1 225nm,4nm

PDA Ch1 225nm

Peak #	Ret. Time	Area %
1	3.96	49.85
2	4.47	50.15
Total		100.00

Figure S13. HPLC traces of compound **5a'**: with complex **3b** (top, left); with complex **2a** (top, right); the corresponding racemate (bottom).

2,2,2-Trichloroethyl (1*S*,2*R*)-1-(4-fluorophenyl)-2-phenylcyclopropane-1-carboxylate (5b'). Prepared according to the general procedure as a white solid; with complex **2a**: 99% yield, 83% *ee*; with complex **3b**: 99% yield, 98% *ee.* [The *ee* was determined by HPLC analysis: Daicel 150 mm Chiralpak IA-3, ∅ 4.6 mm, *n*-heptane/i-propanol = 98/2, v = 1.0 mL/min, λ = 220 nm, t(minor) = 3.29 min, t(major) = 3.55 min]. m. p. = 83-85°C; $[\alpha]_D^{20}$ = + 4.6 (c = 1.5, $CHCl_3$); ^1H NMR (400 MHz, $CDCl_3$): δ = 7.13 – 7.08 (m, 3H), 7.06 – 7.00 (m, 2H), 6.87 – 6.77 (m, 4H), 4.83 (dd, *J* = 11.9, 0.7 Hz, 1H), 4.66 (d, *J* = 12.0 Hz, 1H), 3.22 (dd, *J* = 9.4, 7.4 Hz, 1H), 2.29 (dd, *J* = 9.4, 5.2 Hz, 1H), 1.98 (dd, *J* = 7.4, 5.2 Hz, 1H); ^{13}C NMR (101 MHz, $CDCl_3$): δ = 172.1, 163.3, 160.8, 135.5, 133.7 (d, *J* = 8.2 Hz), 129.8 (d, *J* = 3.1 Hz), 128.2 (d, *J* = 15.9 Hz), 126.9, 114.8 (d, *J* = 21.4 Hz), 95.1, 74.5, 36.6, 34.1, 20.5; ^{19}F NMR (282 MHz, $CDCl_3$): δ = –114.6; IR (ATR): ṽ = 1733, 1604, 1510, 1437, 1235, 1212, 1152, 1055, 833, 810, 783, 771, 710, 694, 579, 543 cm^{-1}; HRMS (ESI^+) for $C_{18}H_{14}Cl_3FO_2Na$ $[M+Na]^+$: calcd: 408.99356, found: 408.99326.

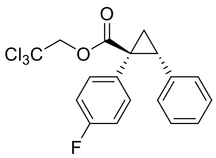

Control Experiment using the Catalyst without the peripheral TIPS-groups: the use of complex **BiRh(*S*-PTPG)₄ (S3)** at room temperature furnished product **5b'** in 94% yield and **24% ee**.

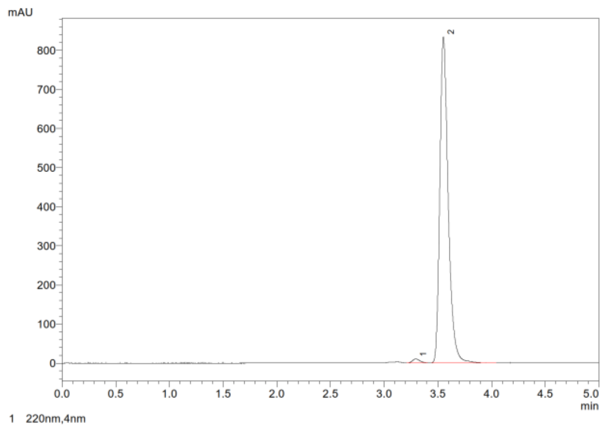

PDA Ch1 220nm

Peak #	Ret. Time	Area %
1	3.29	1.04
2	3.55	98.96
Total		100.00

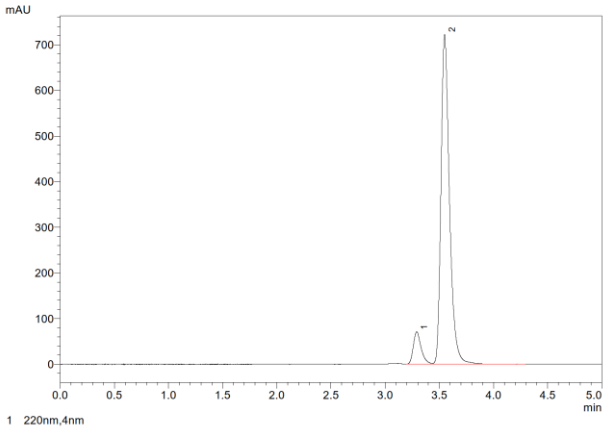

PDA Ch1 220nm

Peak #	Ret. Time	Area %
1	3.29	8.35
2	3.55	91.65
Total		100.00

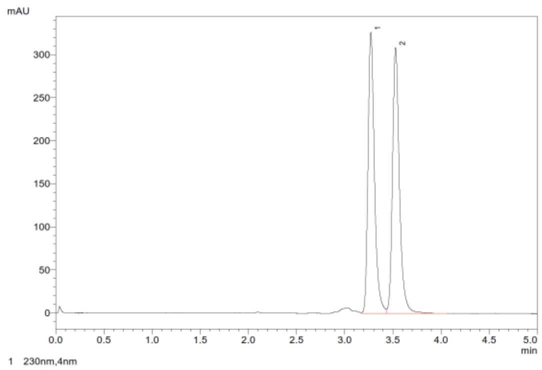

PDA Ch1 230nm

Peak #	Ret. Time	Area %
1	3.27	49.39
2	3.53	50.61
Total		100.00

Figure S14. HPLC traces of compound **5b'**: with complex **3b** (top, left); with complex **2a** (top, right); the corresponding racemate (bottom).

2,2,2-Trichloroethyl (1*S*,2*S*)-1-(4-methoxyphenyl)-2-((trimethylsilyl)methyl)cyclopropane-1-carboxylate

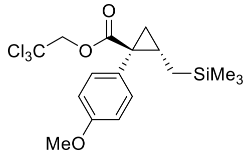

(S5). Prepared according to the general procedure as a colorless oil; with complex **2a**: 91% yield, 99% *ee;* with complex **3b**: 95% yield, 99% *ee*. [The *ee* was determined by HPLC analysis: Daicel 150 mm Chiralpak IC-3, Ø 4.6 mm, n-heptane/2-propanol = 90/10, v = 1.0 mL/min, λ = 220 nm, t(major) = 2.63 min, t(minor) = 3.22 min.] $[\alpha]_D^{20}$ = +35.3 (c = 0.9, $CHCl_3$); ^1H NMR (400 MHz, $CDCl_3$): δ = 7.24 – 7.18 (m, 2H), 6.91 – 6.83 (m, 2H), 4.77 (d, *J* = 11.9 Hz, 1H), 4.56 (d, *J* = 11.9 Hz, 1H), 3.81 (s, 3H), 1.98 – 1.85 (m, 2H), 1.08 (q, *J* = 3.1 Hz, 1H), 0.85 (ddd, *J* = 14.5, 2.8, 1.3 Hz, 1H), 0.01 (s, 9H), -0.32 – -0.47 (m, 1H); ^{13}C NMR (101 MHz, $CDCl_3$): δ = 173.5, 158.8, 133.0, 127.6, 113.5, 95.4, 74.3, 55.3, 32.9, 26.9, 23.7, 18.3, -1.3; ^{29}Si NMR (99 MHz, $CDCl_3$): δ = 2.4; IR (ATR): ṽ = 2953, 1729, 1516, 1244, 1164, 1123, 1034, 834, 709, 572; HRMS (ESI^+) for $C_{17}H_{23}O_3Cl_3Si_1Na_1$ $[M+Na^+]^+$: calcd: 431.03743, found: 431.03706.

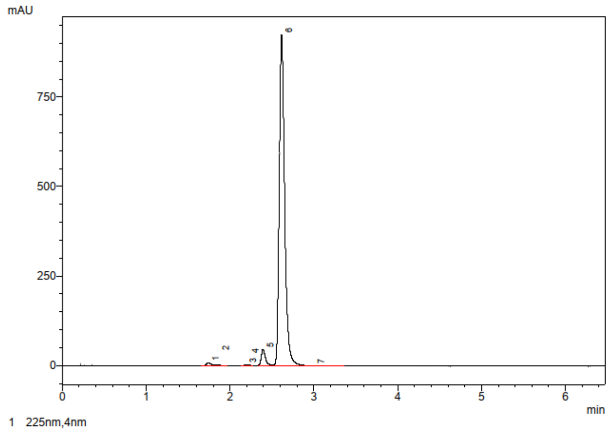

PDA Ch1 225nm

Peak #	Ret. Time	Area %	Name
1	1,74	0,78	
2	1,86	0,18	
3	2,18	0,12	
4	2,22	0,11	
5	2,39	3,71	
6	2,62	94,89	1. Enantiomer
7	3,00	0,20	2. Enantiomer
Total		100,00	

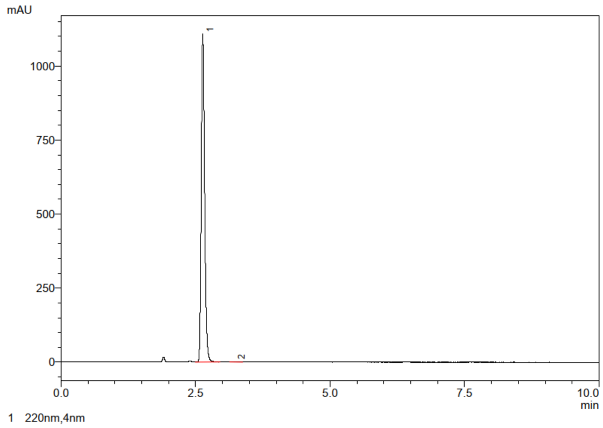

PDA Ch1 220nm

Peak #	Ret. Time	Area %	Name
1	2.63	99.93	1. Enantiomer, ee=99%
2	3.22	0.07	2. Enantiomer
Total		100.00	

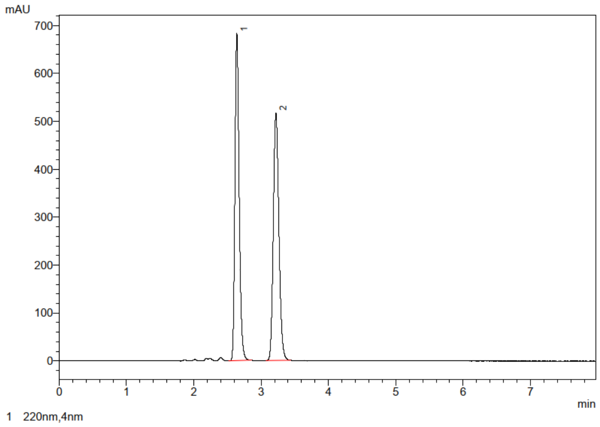

PDA Ch1 220nm

Peak #	Ret. Time	Area %	Name
1	2.64	50.02	1. Enantiomer
2	3.22	49.98	2. Enantiomer
Total		100.00	

Figure S15. HPLC traces of compound **S5**: with complex **3b** (top, left); with complex **2a** (top, right); the corresponding racemate (bottom).

2,2,2-Trichloroethyl (1*S*,2*S*)-1-(4-methoxyphenyl)-2-((4,4,5,5-tetramethyl-1,3,2-dioxaborolan-2-yl)methyl)cyclopropane-1-carboxylate (S6). Prepared to the general procedure as a colorless oil; with complex **2a**: 73% yield, 99% *ee*, with complex **3b**: 45% yield, 98% *ee*. [The *ee* was determined by HPLC analysis: Daicel 150 mm Chiralpak IC-3, Ø 4.6 mm, n-heptane/2-propanol = 90/10, v = 1.0 mL/min, λ = 220 nm, t(major) = 2.98 min, t(minor) = 3.54 min.] $[\alpha]_D^{20}$ = −15.6 (c = 0.9, $CHCl_3$); ^1H NMR (500 MHz, $CDCl_3$): δ = 7.25 – 7.20 (m, 2H), 6.86 – 6.81 (m, 2H), 4.86 (d, *J* = 12.0 Hz, 1H), 4.51 (d, *J* = 11.9 Hz, 1H), 3.79 (s, 3H), 2.04 – 1.98 (m, 1H), 1.88 (dd, *J* = 9.0, 4.3 Hz, 1H), 1.23 (s, 6H), 1.22 (s, 6H), 1.17 (dd, *J* = 6.9, 4.3 Hz, 1H), 0.68 (dd, *J* = 16.5, 6.6 Hz, 1H), 0.39 (dd, *J* = 16.5, 7.9 Hz, 1H); ^{13}C NMR (101 MHz, $CDCl_3$): δ = 173.5, 158.9, 132.9, 127.4, 113.5, 95.4, 83.4, 74.2, 55.4, 33.1, 25.9, 25.0, 24.9, 23.0, 12.7; ^{11}B NMR (128 MHz, $CDCl_3$): δ = 33.8; IR (ATR): ṽ = 2978, 1732, 1516, 1322, 1243, 1142, 1035, 967, 795, 709, 574; HRMS (ESI$^+$) for $C_{20}H_{26}O_5Cl_3B_1Na_1$ [M+Na$^+$]$^+$: calcd: 485.08311, found: 485.08315.

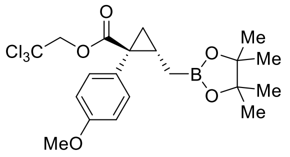

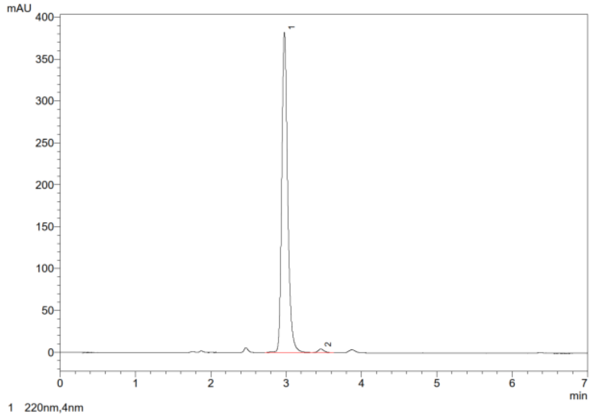

PDA Ch1 220nm

Peak #	Ret. Time	Area %
1	2.98	98.91
2	3.46	1.09
Total		100.00

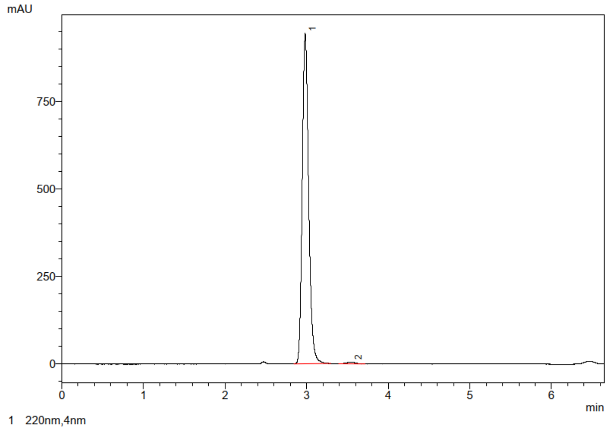

PDA Ch1 220nm

Peak #	Ret. Time	Area %	Name
1	2.98	99.25	1. Enantiomer, ee=99%
2	3.54	0.75	2. Enantiomer
Total		100.00	

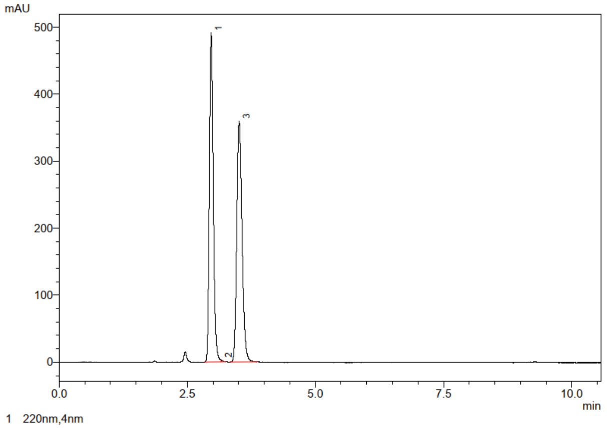

PDA Ch1 220nm

Peak #	Ret. Time	Area %	Name
1	2.96	50.38	1. Enantiomer
2	3.16	0.10	
3	3.51	49.52	2. Enantiomer
Total		100.00	

Figure S16. HPLC traces of compound **S6**: with complex **3b** (top, left); with complex **2a** (top, right); the corresponding racemate (bottom).

(1*S*,2*R*)-2-(4-methoxyphenyl)-2-((2,2,2-trichloroethoxy)carbonyl)cyclopropyl benzoate (*S7*). Prepared according to the general procedure as a colorless oil; with complex **2a**: 57% yield, 96% *ee*; with complex **3b**: 91% yield, 99% *ee*. [The *ee* was determined by HPLC analysis: Daicel 150 mm Chiralpak IA-3, Ø 4.6 mm, n-heptane/2-propanol = 98/2, v = 1.0 mL/min, λ = 225 nm, t(major) = 8.17 min, t(minor) = 7.49 min.] $[\alpha]_D^{20}$ = −26.4 (c = 1.2, $CHCl_3$); ^1H NMR (400 MHz, $CDCl_3$): δ = 7.69 – 7.61 (m, 2H), 7.53 – 7.44 (m, 1H), 7.36 – 7.23 (m, 4H), 6.85 – 6.77 (m, 2H), 5.13 (dd, *J* = 7.2, 4.6 Hz, 1H), 4.85 (d, *J* = 11.9 Hz, 1H), 4.64 (d, *J* = 11.9 Hz, 1H), 3.75 (s, 3H), 2.21 (dd, *J* = 7.2, 6.2 Hz, 1H), 1.92 (dd, *J* = 6.3, 4.7 Hz, 1H); ^{13}C NMR (101 MHz, $CDCl_3$): δ = 170.9, 166.8, 159.3, 133.4, 132.5, 129.6, 129.3, 128.5, 124.8, 113.8, 95.0, 74.5, 58.5, 55.4, 33.7, 20.3; IR (ATR): ṽ = 2959, 1729, 1516, 1240, 1092, 1031, 796, 708, 575; HRMS (ESI^+) for $C_{20}H_{17}O_5Cl_3Na$ $[M+Na^+]^+$: calcd: 465.00338, found: 465.00369.

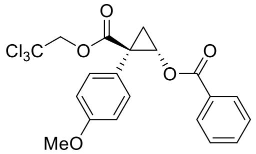

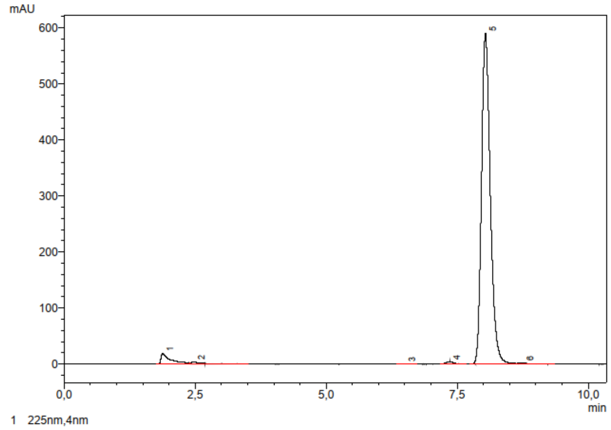

PDA Ch1 225nm

Peak #	Ret. Time	Area %	Name
1	1,88	3,39	
2	2,48	0,80	
3	6,50	0,12	
4	7,35	0,63	1. Enantiomer
5	8,04	94,67	2. Enantiomer 98.7 % ee
6	8,75	0,39	
Total		100,00	

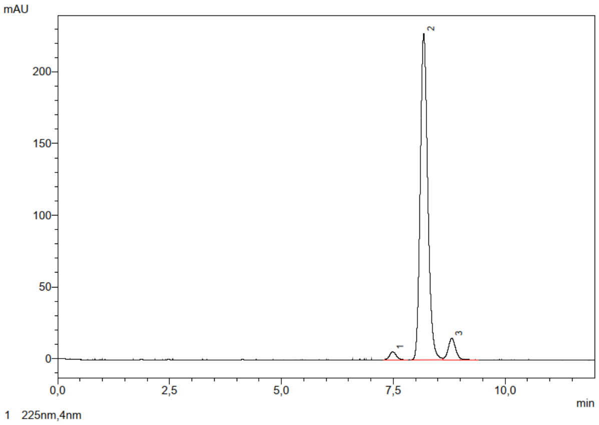

PDA Ch1 225nm

Peak #	Ret. Time	Area %	Name
1	7,49	2,06	1. Enantiomer
2	8,18	91,91	2. Enantiomer 95.6 % ee
3	8,81	6,03	
Total		100,00	

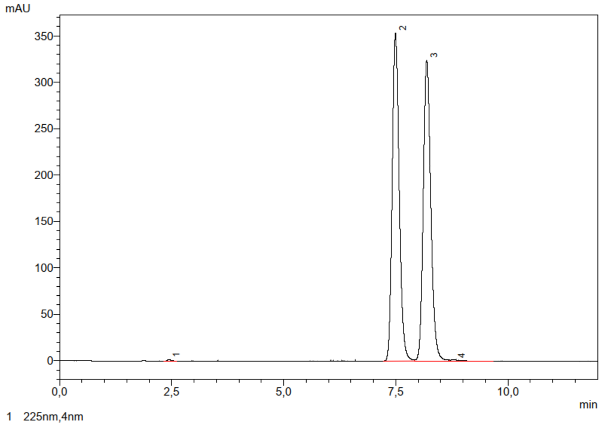

PDA Ch1 225nm

Peak #	Ret. Time	Area %	Name
1	2,44	0,10	
2	7,49	49,68	1. Enantiomer
3	8,19	49,92	2. Enantiomer
4	8,80	0,30	
Total		100,00	

Figure S17. HPLC traces of compound **S7**: with complex **3b** (top, left); with complex **2a** (top, right); the corresponding racemate (bottom).

2,2,2-Trichloroethyl (1*S*,2*R*)-1,2-diphenylcyclopropane-1-carboxylate (S8). Prepared according to the general procedure as a white solid; with complex **2a**: 84% yield, 69% *ee*; with complex **3b**: 87% yield, 96% *ee*. [The *ee* was determined by HPLC analysis: Daicel 150 mm Chiralpak IB-N-3, ∅ 4.6 mm, *n*-heptane/i-propanol = 99/1, v = 1.0 mL/min, λ = 220 nm, t(major) = 4.38 min, t(minor) = 7.81 min]. m. p. = 67-70°C; $[\alpha]_D^{20}$ = +8.8 (c = 3.0, $CHCl_3$); ^{1}H NMR (400 MHz, $CDCl_3$): δ = 7.22 – 7.11 (m, 3H), 7.11 – 7.03 (m, 5H), 6.81 (dd, *J* = 6.7, 2.9 Hz, 2H), 4.85 (d, *J* = 11.9 Hz, 1H), 4.66 (d, *J* = 11.9 Hz, 1H), 3.23 (dd, *J* = 9.4, 7.4 Hz, 1H), 2.29 (dd, *J* = 9.4, 5.1 Hz, 1H), 2.02 (dd, *J* = 7.4, 5.1 Hz, 1H); ^{13}C NMR (101 MHz, $CDCl_3$): δ =172.2, 135.9, 133.8, 132.2, 128.2, 127.9, 127.8, 127.4, 126.7, 95.2, 74.5, 37.4, 34.0, 20.4; IR (ATR): ṽ = 1730, 1499, 1433, 1378, 1239, 1208, 1149, 1096, 1051, 969, 810, 782, 761, 712, 693, 571, 550 cm^{-1}; HRMS (ESI$^+$) for $C_{18}H_{15}Cl_3O_2Na$ [M+Na]$^+$: calcd: 391.00298, found: 391.00275.

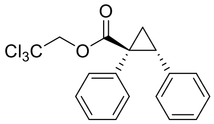

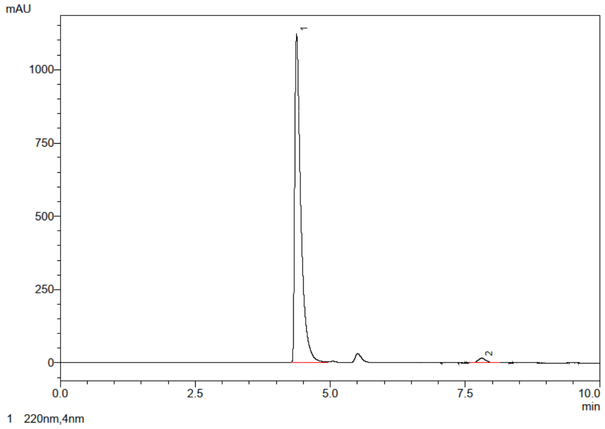

PDA Ch1 220nm

Peak #	Ret. Time	Area %
1	4.38	98.18
2	7.81	1.82
Total		100.00

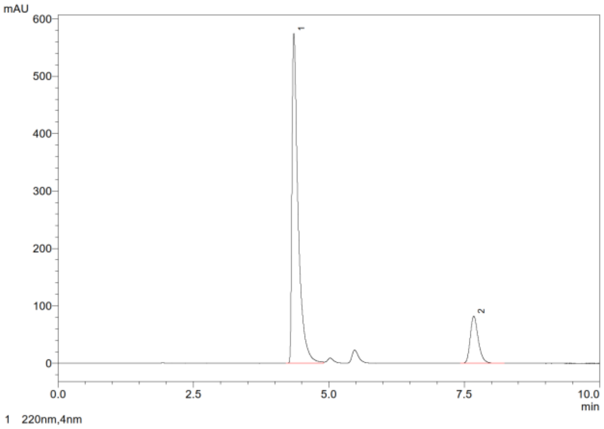

PDA Ch1 220nm

Peak #	Ret. Time	Area %
1	4.35	84.36
2	7.68	15.64
Total		100.00

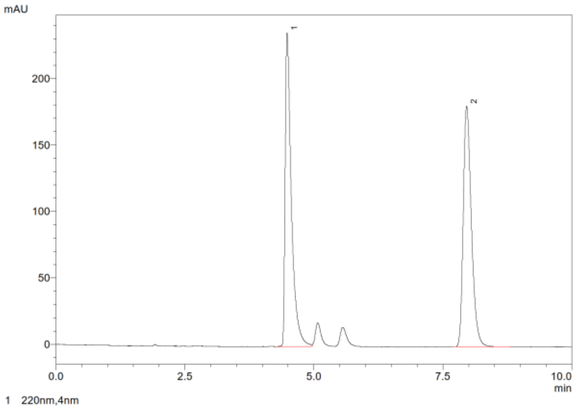

PDA Ch1 220nm

Peak #	Ret. Time	Area %
1	4.48	49.87
2	7.96	50.13
Total		100.00

Figure S18. HPLC traces of compound **S8**: with complex **3b** (top, left); with complex **2a** (top, right); the corresponding racemate (bottom).

Methyl 4-((1*S*,2*R*)-2-phenyl-1-((2,2,2-trichloroethoxy)carbonyl)cyclopropyl)benzoate (S9). Prepared according to the general procedure as a colorless oil; with complex **2a**: 89% yield, 82% *ee*; with complex **3b**: 99% yield, 99% *ee*. [The *ee* was determined by HPLC analysis: Daicel 150 mm Chiralpak IC-3, ∅ 4.6 mm, *n*-heptane/2-propanol = 90/10, v = 1.0 mL/min, λ = 220 nm, t(major) = 4.78 min, t(minor) = 5.64 min.]. ^{1}H NMR (400 MHz, $CDCl_3$): δ = 7.84 – 7.76 (m, 2H), 7.21 – 7.10 (m, 2H), 7.10 – 7.04 (m, 3H), 6.85 – 6.75 (m, 2H), 4.83 (d, *J* = 11.9 Hz, 1H), 4.64 (d, *J* = 11.9 Hz, 1H), 3.86 (s, 3H), 3.26 (dd, *J* = 9.4, 7.5 Hz, 1H), 2.30 (dd, *J* = 9.4, 5.2 Hz, 1H), 2.09 – 2.01 (m, 1H); ^{13}C NMR (101 MHz, $CDCl_3$): δ = 171.6, 167.0, 139.2, 135.2, 132.2, 129.2, 129.1, 128.2, 127.0, 95.0, 74.6, 60.6, 52.2, 37.2, 34.3, 20.3; IR (ATR): ṽ = 2953, 1718, 1611, 1435, 1275, 1240, 1152, 1105, 809, 752, 705, 573; HRMS (ESI^+) for $C_{20}H_{17}Cl_3O_4Na$ $[M+Na^+]^+$: calcd: 449.00846, found: 449.00802.

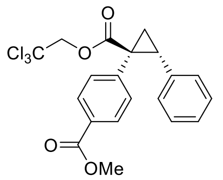

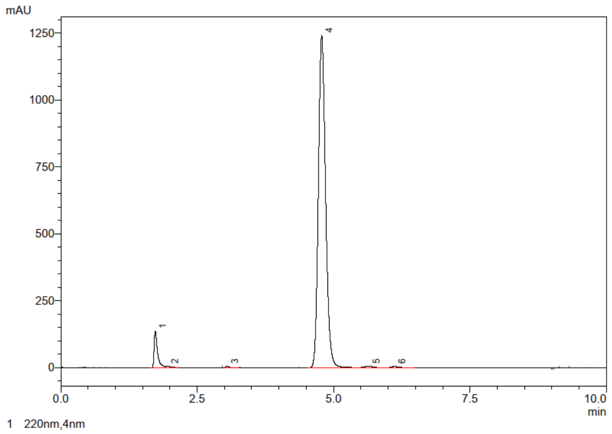

PDA Ch1 220nm

Peak #	Ret. Time	Area %	Name
1	1.73	4.84	
2	1.96	0.30	
3	3.05	0.17	
4	4.78	93.64	1. Enantiomer 98.7 % ee
5	5.65	0.61	2. Enantiomer
6	6.12	0.43	
Total		100.00	

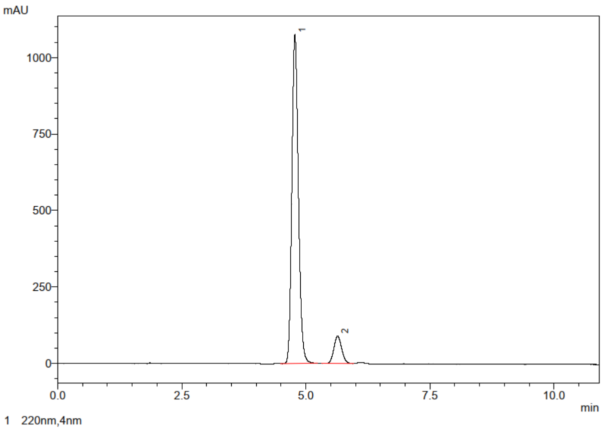

PDA Ch1 220nm

Peak #	Ret. Time	Area %	Name
1	4.78	90.94	1. Enantiomer, ee=82%
2	5.64	9.06	2. Enantiomer
Total		100.00	

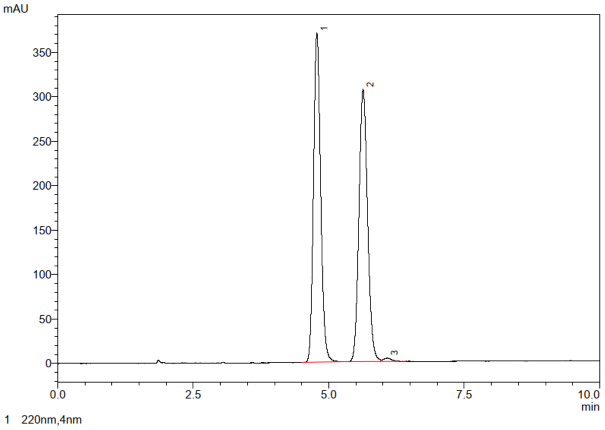

PDA Ch1 220nm

Peak #	Ret. Time	Area %	Name
1	4.78	49.72	1. Enantiomer
2	5.63	49.66	2. Enantiomer
3	6.08	0.62	
Total		100.00	

Figure S19. HPLC traces of compound **S9**: with complex **3b** (top, left); with complex **2a** (top, right); the corresponding racemate (bottom).

2,2,2-Trichloroethyl (1*S*,2*R*)-1-(4-cyanophenyl)-2-phenylcyclopropane-1-carboxylate (S10). Prepared

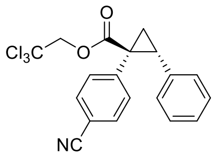

according to the general procedure as a colorless oil; with complex **2a**: 36% yield, 68% *ee*; with complex **3b**: 99% yield, 97% *ee*. [The *ee* was determined by HPLC analysis: Daicel 150 mm Chiralpak IA-3, ∅ 4.6 mm, *n*-heptane/2-propanol = 90/10, v = 1.0 mL/min, λ = 220 nm, t(major) = 4.66 min, t(minor) = 4.14 min.] $[\alpha]_D^{20}$ = −11.1 (c = 1, $CHCl_3$); ^{1}H NMR (400 MHz, $CDCl_3$): δ = 7.44 (d, *J* = 8.1 Hz, 2H), 7.23 – 7.16 (m, 2H), 7.16 – 7.05 (m, 3H), 6.83 – 6.76 (m, 2H), 4.83 (d, *J* = 11.9 Hz, 1H), 4.65 (d, *J* = 11.9 Hz, 1H), 3.29 (dd, *J* = 9.4, 7.5 Hz, 1H), 2.33 (dd, *J* = 9.4, 5.4 Hz, 1H), 2.08 – 2.03 (m, 1H); ^{13}C NMR (101 MHz, $CDCl_3$): δ = 171.1, 139.6, 134.8, 132.9, 131.7, 128.3, 128.1, 127.3, 118.8, 111.4, 94.9, 74.6, 37.1, 34.4, 20.1; IR (ATR): ṽ = 2957, 2229, 1732, 1374, 1239, 1152, 1094, 1049, 808, 713, 695, 601, 567; HRMS (EI) for $C_{19}H_{14}NO_2Cl_3$ $[M^+]^+$: calcd: 393.00846, found: 393.00872.

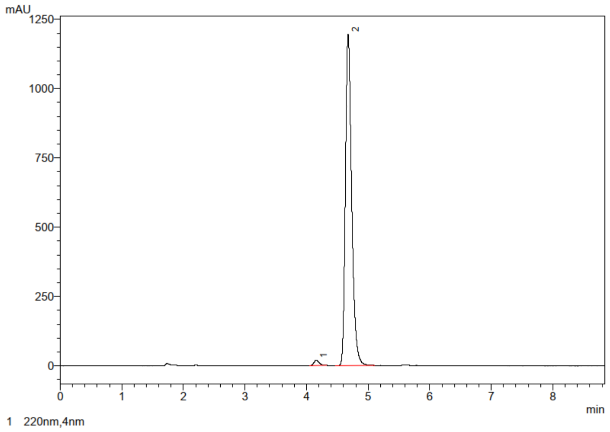

PDA Ch1 220nm

Peak #	Ret. Time	Area %	Name
1	4.16	1.30	1. Enantiomer
2	4.67	98.70	2. Enantiomer, ee=97%
Total		100.00	

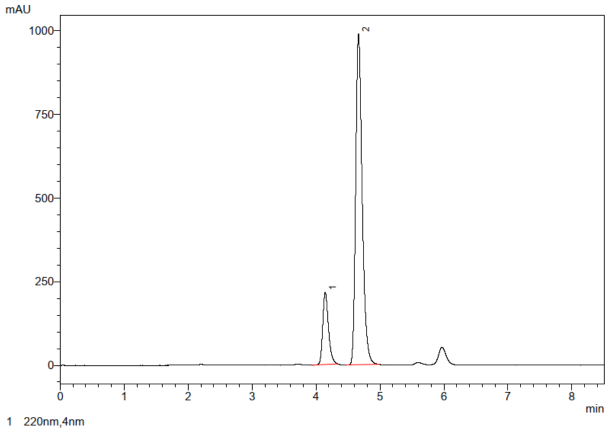

PDA Ch1 220nm

Peak #	Ret. Time	Area %	Name
1	4.14	16.10	1. Enantiomer
2	4.66	83.90	2. Enantiomer, ee=68%
Total		100.00	

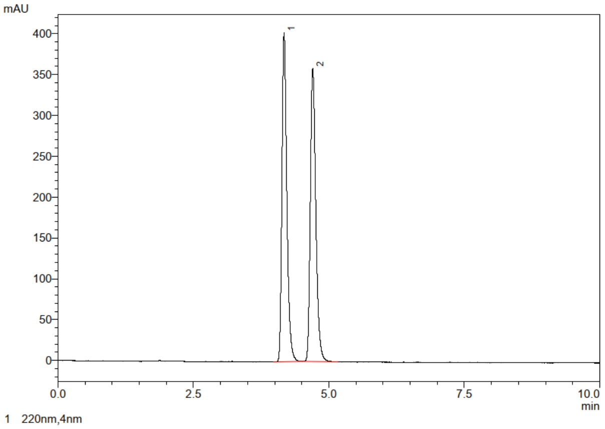

PDA Ch1 220nm

Peak #	Ret. Time	Area %	Name
1	4.17	50.02	1. Enantiomer
2	4.70	49.98	2. Enantiomer
Total		100.00	

Figure S20. HPLC traces of compound **S10**: with complex **3b** (top, left); with complex **2a** (top, right); the corresponding racemate (bottom).

2,2,2-Trichloroethyl (1*S*,2*R*)-2-phenyl-1-(4-(4,4,5,5-tetramethyl-1,3,2-dioxaborolan-2-yl)phenyl)cyclopropane-1-carboxylate (S11). Prepared according to the general procedure as a colorless oil; with complex **2a**: 70% yield, 99% *ee*; with complex **3b**: 69% yield, 99% *ee*. [The *ee* was determined by HPLC analysis: Daicel 150 mm Chiralpak IB-N-3, ∅ 4.6 mm, *n*-heptane/2-propanol = 98/2, v = 1.0 mL/min, λ = 225 nm, t(major) = 3.50 min, t(minor) = 5.20 min.] $[\alpha]_D^{20}$ = −4.3 (c = 1.1, $CHCl_3$); ^1H NMR (400 MHz, $CDCl_3$): δ = 7.60 – 7.53 (m, 2H), 7.13 – 7.02 (m, 5H), 6.85 – 6.75 (m, 2H), 4.84 (d, *J* = 11.9 Hz, 1H), 4.63 (d, *J* = 11.9 Hz, 1H), 3.22 (dd, *J* = 9.4, 7.5 Hz, 1H), 2.27 (dd, *J* = 9.4, 5.1 Hz, 1H), 2.07 – 1.97 (m, 1H), 1.31 (s, 12H); ^{13}C NMR (101 MHz, $CDCl_3$): δ = 172.1, 136.9, 135.7, 134.3, 131.5, 128.3, 128.1, 126.8, 95.2, 83.9, 74.5, 37.5, 34.2, 25.1, 25.0, 20.4 (C-B was not detected); ^{11}B NMR (128 MHz, $CDCl_3$): δ = 30.3; IR (ATR): ṽ = 2977, 1738, 1612, 1397, 1358, 1323, 1238, 1152, 1097, 1052, 1017, 856, 808, 703, 652, 570; HRMS (EI) for $C_{24}H_{26}BO_4Cl_3$ $[M^+]^+$: calcd: 494.09842, found: 494.09923.

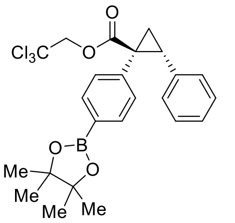

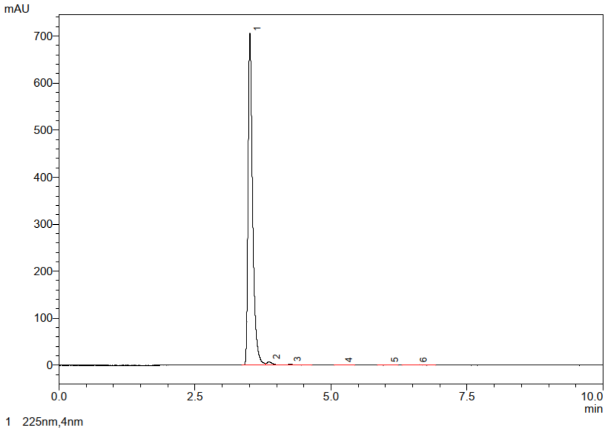

PDA Ch1 225nm

Peak #	Ret. Time	Area %	Name
1	3.51	97.51	1. Enantiomer, ee=99%
2	3.86	1.39	
3	4.25	0.41	
4	5.19	0.15	2. Enantiomer
5	6.04	0.14	
6	6.56	0.41	
Total		100.00	

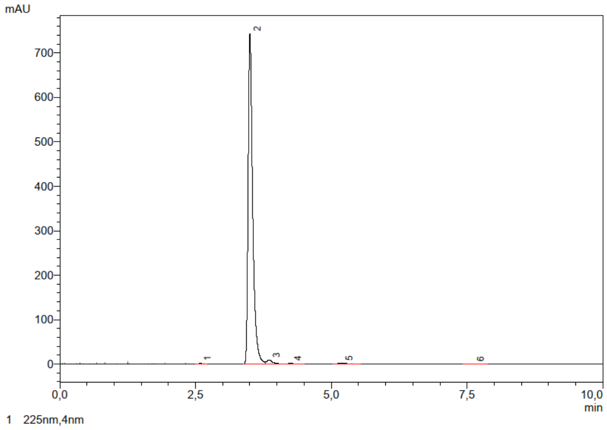

PDA Ch1 225nm

Peak #	Ret. Time	Area %	Name
1	2,58	0,16	
2	3,50	97,34	1. Enantiomer 98.9 % ee
3	3,86	1,63	
4	4,25	0,24	
5	5,20	0,52	2. Enantiomer
6	7,61	0,11	
Total		100,00	

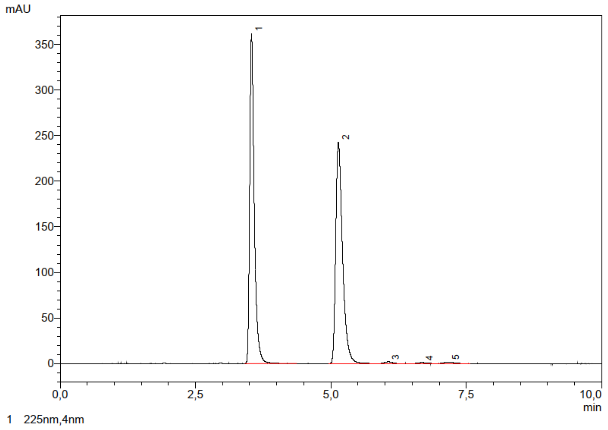

PDA Ch1 225nm

Peak #	Ret. Time	Area %	Name
1	3,54	49,23	1. Enantiomer
2	5,14	49,21	2. Enantiomer
3	6,06	0,56	
4	6,68	0,40	
5	7,17	0,60	
Total		100,00	

Figure S21. HPLC traces of compound **S11**: with complex **3b** (top, left); with complex **2a** (top, right); the corresponding racemate (bottom).

2,2,2-Trichloroethyl (1*S*,2*R*)-1-(4-(methylsulfonyl)phenyl)-2-phenylcyclopropane-1-carboxylate (S12).

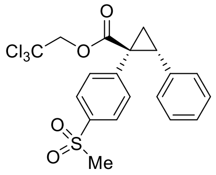

Prepared according to the general procedure in CH_2Cl_2/pentane (1/5) as a colorless oil; with complex **2a**: 56% yield, 97% *ee*; with complex **3b**: 85% yield, 99% *ee*. [The *ee* was determined by HPLC analysis: Daicel 150 mm Chiralpak IB-N-3, ∅ 4.6 mm, *n*-heptane/2-propanol = 90/10, v = 1.0 mL/min, λ = 225 nm, t(major) = 14.21 min, t(minor) = 16.22 min.] $[\alpha]_D^{20}$ = +2.5 (c = 0.9, $CHCl_3$); ^1H NMR (400 MHz, $CDCl_3$): δ = 7.74 – 7.65 (m, 2H), 7.31 – 7.24 (m, 2H), 7.13 – 7.04 (m, 3H), 6.84 – 6.75 (m, 2H), 4.82 (d, *J* = 11.9 Hz, 1H), 4.66 (d, *J* = 11.9 Hz, 1H), 3.31 (dd, *J* = 9.4, 7.5 Hz, 1H), 2.96 (s, 3H), 2.36 (dd, *J* = 9.4, 5.4 Hz, 1H), 2.11 – 2.04 (m, 1H); ^{13}C NMR (101 MHz, $CDCl_3$): δ = 171.1, 140.6, 139.4, 134.7, 133.2, 128.4, 128.1, 127.4, 126.9, 95.0, 74.6, 44.7, 37.0, 34.3, 20.1; IR (ATR): ṽ = 2930, 1732, 1312, 1240, 1149, 1095, 1049, 956, 771, 713, 597, 553; HRMS (ESI$^+$) for $C_{19}H_{17}O_4SCl_3Na$ [M+Na$^+$]$^+$: calcd: 468.98054, found: 468.98098.

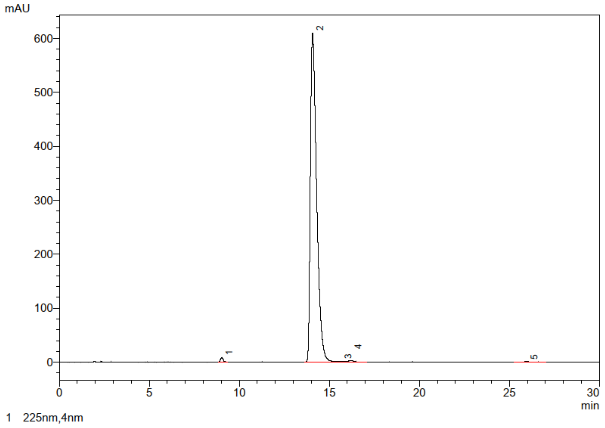

PDA Ch1 225nm

Peak #	Ret. Time	Area %	Name
1	9,03	0,58	
2	14,08	98,54	1. Enantiomer 99.0 % ee
3	15,64	0,17	
4	16,20	0,48	2. Enantiomer
5	25,98	0,23	
Total		100,00	

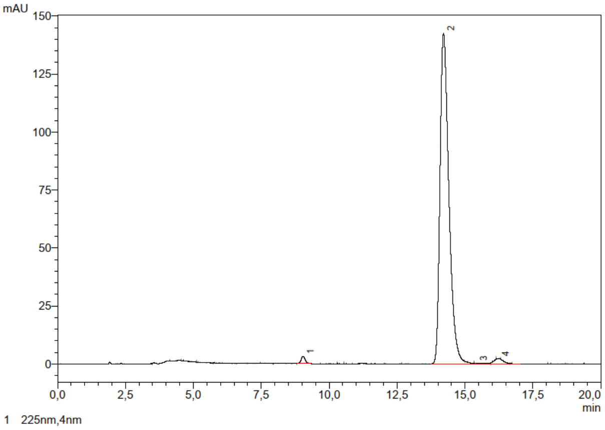

PDA Ch1 225nm

Peak #	Ret. Time	Area %	Name
1	9,04	0,96	
2	14,21	97,16	1. Enantiomer 96.5 % ee
3	15,42	0,17	
4	16,22	1,71	2. Enantiomer
Total		100,00	

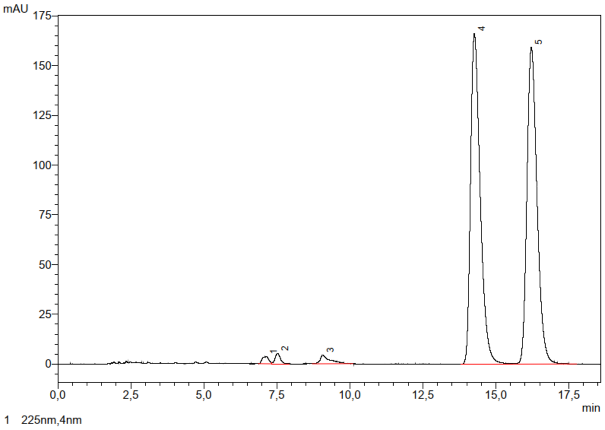

PDA Ch1 225nm

Peak #	Ret. Time	Area %	Name
1	7,14	0,72	
2	7,53	0,85	
3	9,08	1,32	
4	14,26	48,51	1. Enantiomer
5	16,21	48,60	2. Enantiomer
Total		100,00	

Figure S22. HPLC traces of compound **S12**: with complex **3b** (top, left); with complex **2a** (top, right); the corresponding racemate (bottom).

2,2,2-Trichloroethyl (1*S*,2*S*)-2-butyl-1-(4-fluorophenyl)cyclopropane-1-carboxylate (S13). Prepared according to the general procedure as a colorless oil; with complex **2a**: 72% yield, 79% *ee*; with complex **3b**: 83% yield, 96% *ee*. [The *ee* was determined by HPLC analysis: Daicel 150 mm Chiralcel OJ-3, ∅ 4.6 mm, *n*-heptane/2-propanol = 98/2, v = 1.0 mL/min, λ = 220 nm, t(major) = 3.60 min, t(minor) = 2.83 min.] $[\alpha]_D^{20}$ = +11.8 (c = 1.1, $CHCl_3$); ^1H NMR (400 MHz, $CDCl_3$): δ = 7.31 – 7.23 (m, 2H), 7.09 – 6.96 (m, 2H), 4.78 (d, *J* = 11.9 Hz, 1H), 4.56 (d, *J* = 11.9 Hz, 1H), 1.93 (dddd, *J* = 11.0, 8.7, 6.6, 4.5 Hz, 1H), 1.86 (ddd, *J* = 9.1, 4.0, 0.6 Hz, 1H), 1.44 – 1.31 (m, 3H), 1.26 (dtd, *J* = 15.2, 7.5, 1.9 Hz, 2H), 1.18 (dd, *J* = 6.7, 4.0 Hz, 1H), 0.83 (t, *J* = 7.2 Hz, 3H), 0.67 – 0.51 (m, 1H); ^{13}C NMR (101 MHz, $CDCl_3$): δ = 173.0, 162.2 (d, *J* = 245.9 Hz), 133.1 (d, *J* = 8.1 Hz), 131.3 (d, *J* = 3.4 Hz), 115.1 (d, *J* = 21.5 Hz), 95.2, 74.4, 33.0, 31.3, 30.1, 29.7, 22.6, 22.3, 14.1; ^{19}F NMR (282 MHz, $CDCl_3$): δ = –115.09; IR (ATR): ṽ = 2930, 1733, 1512, 1253, 1222, 1158, 1103, 1046, 837, 804, 755, 719, 573; HRMS (ESI^+) for $C_{16}H_{18}O_2FCl_3$ $[M+Na^+]^+$: calcd: 366.03509, found: 366.03536.

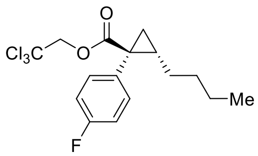

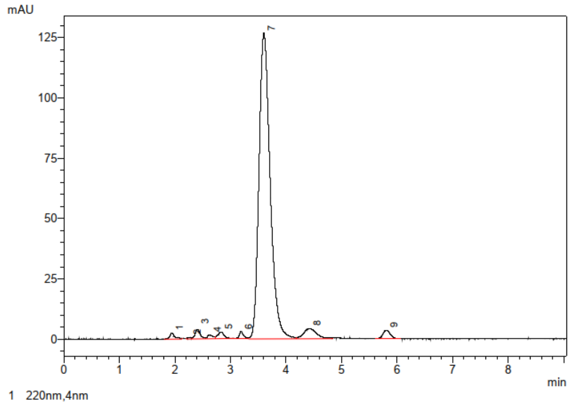

PDA Ch1 220nm

Peak #	Ret. Time	Area %	Name
1	1,95	0,85	
2	2,26	0,18	
3	2,41	1,31	
4	2,63	0,56	
5	2,83	1,06	1. Enantiomer
6	3,19	0,94	
7	3,60	89,89	2. Enantiomer 97.7 % ee
8	4,42	3,58	
9	5,81	1,64	
Total		100,00	

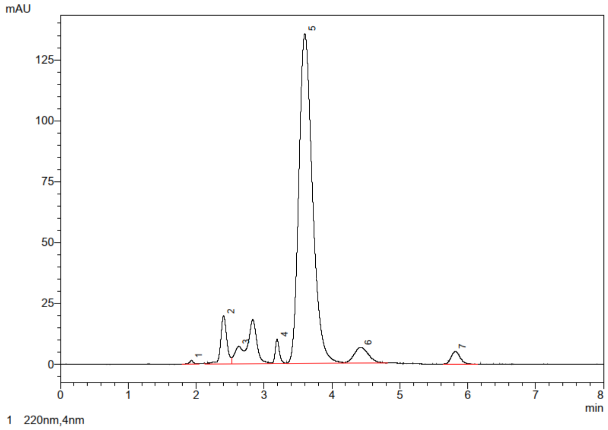

PDA Ch1 220nm

Peak #	Ret. Time	Area %	Name
1	1,93	0,31	
2	2,40	5,17	
3	2,63	8,95	1. Enantiomer + Verunreinigung
4	3,19	1,77	
5	3,60	77,82	2. Enantiomer 79 % ee
6	4,43	3,99	
7	5,81	1,98	
Total		100,00	

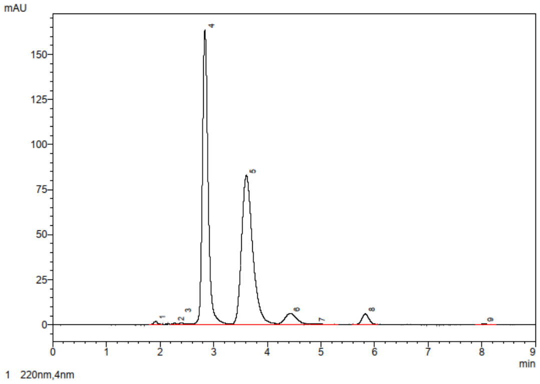

PDA Ch1 220nm

Peak #	Ret. Time	Area %	Name
1	1,92	0,40	
2	2,27	0,18	
3	2,40	0,33	
4	2,84	46,52	1. Enantiomer
5	3,61	46,36	2. Enantiomer
6	4,43	3,75	
7	4,90	0,21	
8	5,83	2,09	
9	8,04	0,16	
Total		100,00	

Figure S23. HPLC traces of compound **S13**: with complex **3b** (top, left); with complex **2a** (top, right); the corresponding racemate (bottom).

2,2,2-Trichloroethyl (1*S*,2*S*)-1-(4-fluorophenyl)-2-((trimethylsilyl)ethynyl)cyclopropane-1-carboxylate (S14).

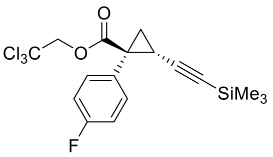

Prepared according to the general procedure as a colorless oil; with complex **2a**: 55% yield, 77% *ee*; with complex **3b**: 65% yield, 96% *ee*. [The *ee* was determined by HPLC analysis: Daicel 150 mm Chiralpak IB-N-3, ∅ 4.6 mm, *n*-heptane/2-propanol = 98/2, v = 1.0 mL/min, λ = 220 nm, t(major) = 2.53 min, t(minor) = 2.77 min.] $[\alpha]_D^{20}$ = +155.8 (c = 0.9, $CHCl_3$); ^1H NMR (400 MHz, $CDCl_3$): δ = 7.41 – 7.30 (m, 2H), 7.08 – 6.97 (m, 2H), 4.75 (d, *J* = 11.9 Hz, 1H), 4.60 (d, *J* = 11.9 Hz, 1H), 2.52 (dd, *J* = 9.3, 6.7 Hz, 1H), 2.12 – 2.02 (m, 1H), 1.65 (dd, *J* = 6.7, 4.4 Hz, 1H), -0.06 (s, 9H); ^{13}C NMR (101 MHz, $CDCl_3$): δ = 171.0, 162.5 (d, *J* = 246.4 Hz), 133.4 (d, *J* = 8.2 Hz), 130.2 (d, *J* = 3.4 Hz), 114.9 (d, *J* = 21.6 Hz), 103.4, 94.8, 88.3, 74.7, 34.9, 24.0, 19.4, -0.3; ^{19}F NMR (282 MHz, $CDCl_3$): δ = –114.5; ^{29}Si NMR (60 MHz, $CDCl_3$): δ = -18.3; IR (ATR): ṽ = 2959, 2165, 1738, 1513, 1235, 1159, 1047, 875, 837, 806, 757, 718, 644, 578; HRMS (EI) for $C_{17}H_{18}O_2SiFCl_3$ $[M]^+$: calcd: 406.01202, found: 406.01169.

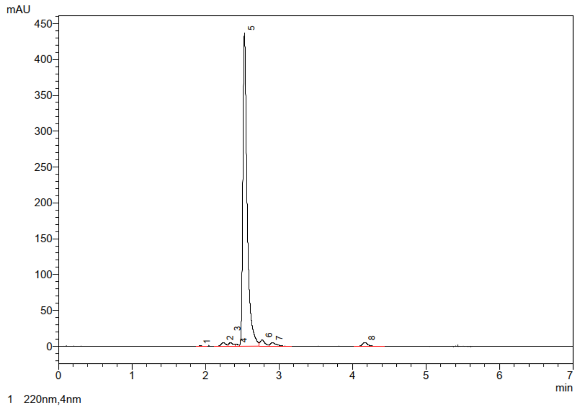

PDA Ch1 220nm

Peak #	Ret. Time	Area %	Name
1	1,93	0,15	
2	2,24	1,25	
3	2,34	1,12	
4	2,43	0,38	
5	2,53	92,00	1. Enantiomer 95.6 % ee
6	2,77	2,08	2. Enantiomer
7	2,91	1,53	
8	4,17	1,49	
Total		100,00	

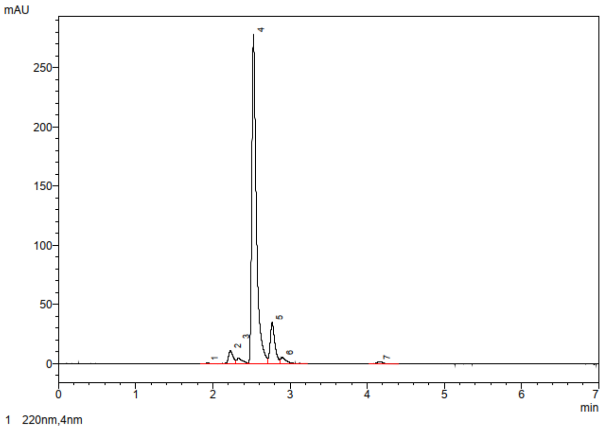

PDA Ch1 220nm

Peak #	Ret. Time	Area %	Name
1	1,93	0,20	
2	2,23	3,41	
3	2,34	1,84	
4	2,53	81,15	1. Enantiomer 76.9 % ee
5	2,77	10,60	2. Enantiomer
6	2,90	2,04	
7	4,16	0,78	
Total		100,00	

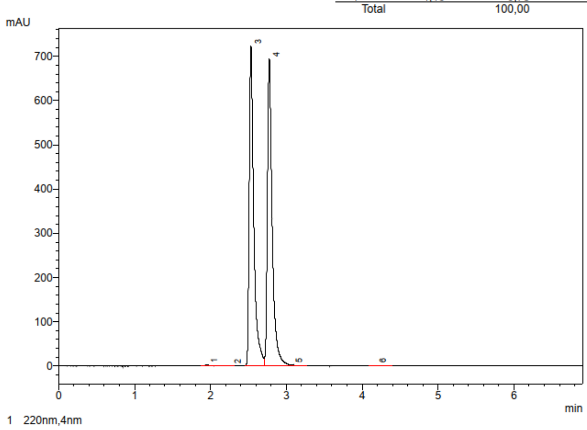

PDA Ch1 220nm

Peak #	Ret. Time	Area %	Name
1	1,95	0,15	
2	2,27	0,11	
3	2,53	49,29	1. Enantiomer
4	2,77	50,14	2. Enantiomer
5	3,08	0,18	
6	4,18	0,13	
Total		100,00	

Figure S24. HPLC traces of compound **S14**: with complex **3b** (top, left); with complex **2a** (top, right); the corresponding racemate (bottom).

2,2,2-Trichloroethyl (1*S*,2*S*)-2-(bromomethyl)-1-(4-fluorophenyl)cyclopropane-1-carboxylate (S15).

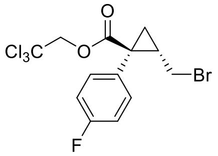

Prepared according to the general procedure as a white sticky solid; with complex **2a**: 52% yield, 83% *ee*; with complex **3b**: 89% yield, 99% *ee*. [The *ee* was determined by HPLC analysis: Daicel 150 mm Chiralpak OJ-3R, Ø 4.6 mm, CH_3CN/water = 70/30, v = 0.5 mL/min, λ = 210 nm, t(minor) = 10.62 min, t(major) = 9.78 min]. $[\alpha]_D^{20}$ = −9.4 (c = 1.0, $CHCl_3$); ^1H NMR (400 MHz, $CDCl_3$): δ = 7.44 – 7.32 (m, 2H), 7.13 – 6.98 (m, 2H), 4.80 (d, *J* = 11.9 Hz, 1H), 4.58 (d, *J* = 11.9 Hz, 1H), 3.02 (d, *J* = 7.5 Hz, 2H), 2.54 – 2.42 (m, 1H), 2.02 (dd, *J* = 9.2, 5.0 Hz, 1H), 1.39 (dd, *J* = 6.8, 5.0 Hz, 1H); ^{13}C NMR (101 MHz, $CDCl_3$): δ = 171.5, 162.7 (d, *J* = 247.4 Hz), 133.3 (d, *J* = 8.4 Hz), 129.1 (d, *J* = 3.2 Hz), 115.5 (d, *J* = 21.6 Hz), 94.9, 74.6, 36.2, 32.2, 30.5, 22.5; ^{19}F NMR (282 MHz, $CDCl_3$): δ = -113.6; IR (ATR): ṽ = 1734, 1512, 1247, 1223, 1159, 1045, 840, 803, 755, 718, 570, 543; HRMS (ESI^+) for $C_{13}H_{11}{}^{79}BrCl_3FO_2Na$ $[M+Na^+]^+$: calcd: 424.88843, found: 424.88844.

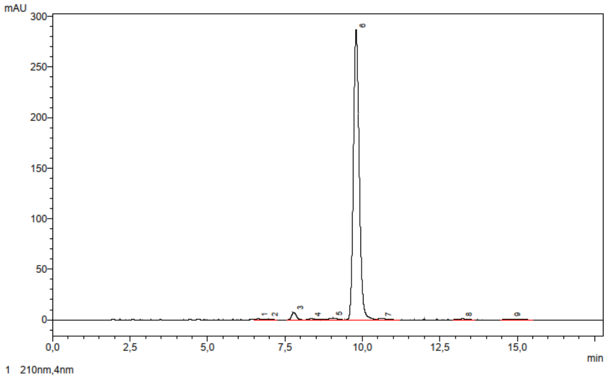

PDA Ch1 210nm

Peak #	Ret. Time	Area %	Name
1	6,63	0,27	
2	6,97	0,26	
3	7,79	1,94	
4	8,35	0,50	
5	9,05	0,85	
6	9,80	94,44	1. Enantiomer
7	10,63	0,66	2. Enantiomer 98.6 % ee
8	13,23	0,49	
9	14,79	0,59	
Total		100,00	

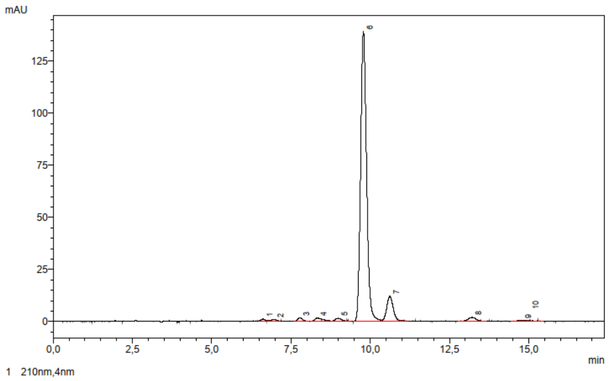

PDA Ch1 210nm

Peak #	Ret. Time	Area %	Name
1	6,62	0,48	
2	6,96	0,54	
3	7,78	0,83	
4	8,33	1,33	
5	8,98	0,99	
6	9,78	85,58	1. Enantiomer 82.7 % ee
7	10,62	8,11	2. Enantiomer
8	13,21	1,52	
9	14,78	0,41	
10	15,01	0,21	
Total		100,00	

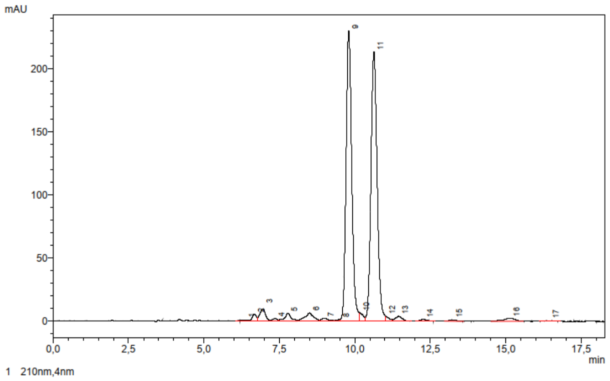

PDA Ch1 210nm

Peak #	Ret. Time	Area %	Name
1	6,37	0,22	
2	6,68	0,80	
3	6,96	1,83	
4	7,36	0,33	
5	7,78	1,34	
6	8,51	1,92	
7	8,99	0,54	
8	9,50	0,14	
9	9,80	44,34	1. Enantiomer
10	10,17	0,78	
11	10,63	44,66	2. Enantiomer
12	11,02	0,42	
13	11,45	0,90	
14	12,28	0,29	
15	13,25	0,29	
16	15,14	1,00	
17	16,45	0,19	
Total		100,00	

Figure S25. HPLC traces of compound **S15**: with complex **3b** (top, left); with complex **2a** (top, right); the corresponding racemate (bottom).

2,2,2-Trichloroethyl (1*S*,2*R*)-1-(3-methoxyphenyl)-2-phenylcyclopropane-1-carboxylate (S16). Prepared according to the general procedure as a colorless oil; with complex **2a**: 92% yield, 58% *ee*; with complex **3b**: 98% yield, 87% *ee*. [The *ee* was determined by HPLC analysis: Daicel 150 mm Chiralpak IB-N-3, ∅ 4.6 mm, *n*-heptane/2-propanol = 98/2, v = 1.0 mL/min, λ = 220 nm, t(major) = 3.93 min, t(minor) = 4.24 min.] $[\alpha]_D^{20}$ = +17.8 (c = 1.2, $CHCl_3$); ^{1}H NMR (500 MHz, $CDCl_3$): δ = 7.09 (dd, *J* = 5.0, 1.9 Hz, 3H), 7.05 (t, *J* = 7.9 Hz, 1H), 6.85 – 6.80 (m, 2H), 6.68 (dddd, *J* = 7.0, 3.6, 2.1, 1.0 Hz, 2H), 6.56 (dd, *J* = 2.6, 1.6 Hz, 1H), 4.86 (d, *J* = 11.9 Hz, 1H), 4.64 (d, *J* = 11.9 Hz, 1H), 3.59 (s, 3H), 3.20 (dd, *J* = 9.4, 7.4 Hz, 1H), 2.26 (dd, *J* = 9.4, 5.1 Hz, 1H), 2.00 (dd, *J* = 7.5, 5.1 Hz, 1H); ^{13}C NMR (101 MHz, $CDCl_3$): δ = 172.2, 159.0, 135.9, 135.3, 128.7, 128.2, 128.0, 126.8, 124.6, 117.6, 113.6, 95.2, 74.5, 55.2, 37.3, 34.0, 20.5; IR (ATR): ṽ = 2957, 1732, 1584, 1433, 1238, 1147, 1043, 804, 694, 572; HRMS (ESI^+) for $C_{19}H_{17}O_3Cl_3Na$ $[M+Na^+]^+$: calcd: 421.01355, found: 421.01384.

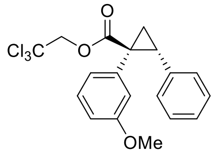

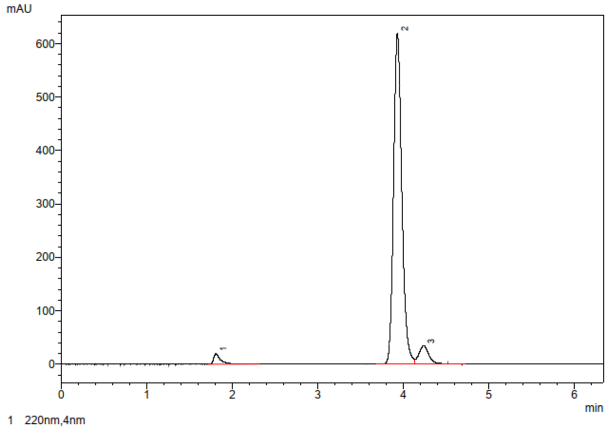

PDA Ch1 220nm

Peak #	Ret. Time	Area %	Name
1	1,81	2,48	
2	3,93	91,36	1. Enantiomer 87.4 % ee
3	4,24	6,16	2. Enantiomer
Total		100,00	

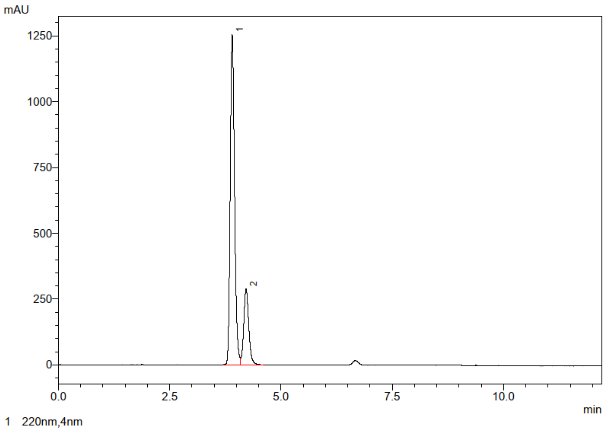

PDA Ch1 220nm

Peak #	Ret. Time	Area %	Name
1	3.91	79.04	1. Enantiomer, ee=58%
2	4.22	20.96	2. Enantiomer
Total		100.00	

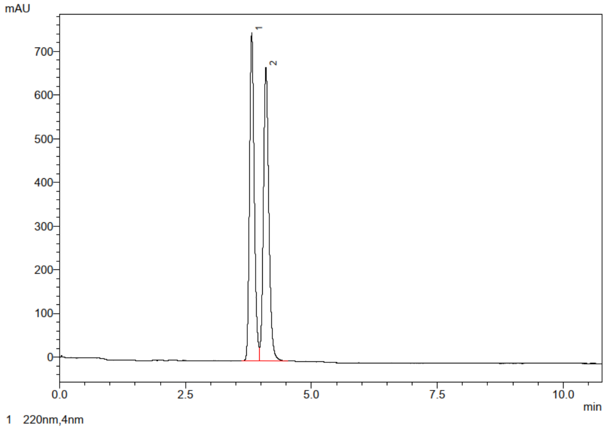

PDA Ch1 220nm

Peak #	Ret. Time	Area %	Name
1	3.82	49.43	1. Enantiomer
2	4.10	50.57	2. Enantiomer
Total		100.00	

Figure S26. HPLC traces of compound **S16**: with complex **3b** (top, left); with complex **2a** (top, right); the corresponding racemate (bottom).

2,2,2-Trichloroethyl (1*S*,2*R*)-1-(3-bromophenyl)-2-phenylcyclopropane-1-carboxylate (S17). Prepared according to the general procedure as a colorless oil; with complex **2a**: 98% yield, 74% *ee*; with complex **3b**: 99% yield, 95% *ee*. [The *ee* was determined by HPLC analysis: Daicel 150 mm Chiralcel OJ-3, ∅ 4.6 mm, *n*-heptane/2-propanol = 95/5, v = 1.0 mL/min, λ = 220 nm, t(major) = 5.66 min, t(minor) = 4.18 min.] $[\alpha]_D^{20}$ = −10.5 (c = 1.2, $CHCl_3$); ^{1}H NMR (400 MHz, $CDCl_3$): δ = 7.30 – 7.21 (m, 2H), 7.17 – 7.06 (m, 3H), 7.02 – 6.91 (m, 2H), 6.86 – 6.79 (m, 2H), 4.85 (d, *J* = 11.8 Hz, 1H), 4.64 (d, *J* = 11.9 Hz, 1H), 3.23 (dd, *J* = 9.4, 7.5 Hz, 1H), 2.28 (dd, *J* = 9.4, 5.2 Hz, 1H), 2.00 (dd, *J* = 7.5, 5.2 Hz, 1H); ^{13}C NMR (101 MHz, $CDCl_3$): δ = 171.7, 136.3, 135.2, 135.1, 130.9, 130.6, 129.3, 128.2, 128.2, 127.1, 121.7, 95.1, 74.6, 36.8, 34.2, 20.2; IR (ATR): ṽ = 3032, 1731, 1374, 1238, 1151, 1108, 1049, 813, 765, 697, 572; HRMS (ESI$^+$) for $C_{18}H_{14}O_2{}^{79}BrCl_3Na$ [M+Na$^+$]$^+$: calcd: 468.91351, found: 468.91391.

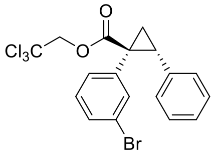

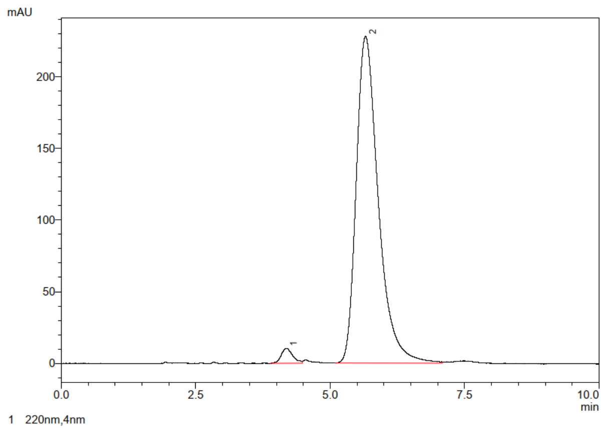

PDA Ch1 220nm

Peak #	Ret. Time	Area %	Name
1	4.18	2.29	1. Enantiomer
2	5.66	97.71	2. Enantiomer, ee=95%
Total		100.00	

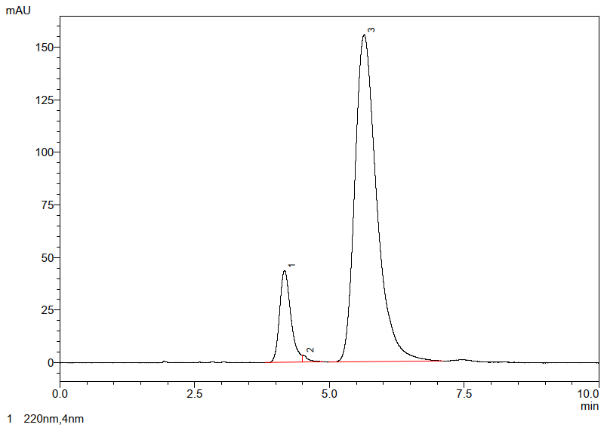

PDA Ch1 220nm

Peak #	Ret. Time	Area %	Name
1	4.17	12.53	1. Enantiomer
2	4.51	0.46	
3	5.64	87.01	2. Enantiomer, ee=74%
Total		100.00	

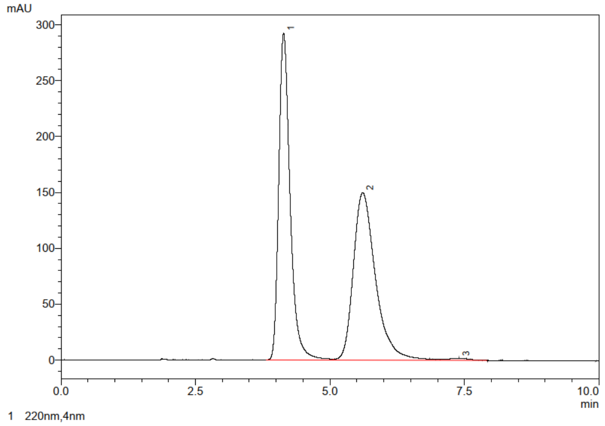

PDA Ch1 220nm

Peak #	Ret. Time	Area %	Name
1	4.14	49.68	1. Enantiomer
2	5.61	49.73	2. Enantiomer
3	7.40	0.59	
Total		100.00	

Figure S27. HPLC traces of compound **S17**: with complex **3b** (top, left); with complex **2a** (top, right); the corresponding racemate (bottom).

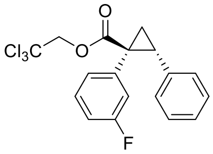

2,2,2-Trichloroethyl (1*S*,2*R*)-1-(3-fluorophenyl)-2-phenylcyclopropane-1-carboxylate (S18). Prepared according to the general procedure as a colorless oil; with complex **2a**: 98% yield, 80% *ee*; with complex **3b**: 97% yield, 97% *ee*. [The *ee* was determined by HPLC analysis: Daicel 150 mm Chiralpak IB-N-3, ∅ 4.6 mm, *n*-heptane/2-propanol = 98/2, v = 1.0 mL/min, λ = 220 nm, t(major) = 3.83 min, t(minor) = 4.51 min.] $[\alpha]_D^{20}$ = +21.5 (c = 1.1, $CHCl_3$); ^1H NMR (400 MHz, $CDCl_3$): δ = 7.15 – 7.04 (m, 4H), 6.81 (d, *J* = 0.5 Hz, 5H), 4.83 (d, *J* = 11.9 Hz, 1H), 4.66 (d, *J* = 11.9 Hz, 1H), 3.24 (dd, *J* = 9.4, 7.5 Hz, 1H), 2.28 (dd, *J* = 9.4, 5.2 Hz, 1H), 2.01 (dd, *J* = 7.5, 5.2 Hz, 1H); ^{13}C NMR (101 MHz, $CDCl_3$): δ = 171.7, 162.3 (d, *J* = 245.3 Hz), 136.4 (d, *J* = 7.7 Hz), 135.3, 129.2 (d, *J* = 8.4 Hz), 128.2, 128.1, 127.9 (d, *J* = 2.8 Hz), 127.0, 119.1 (d, *J* = 21.5 Hz), 114.5 (d, *J* = 21.1 Hz), 95.1, 74.6, 37.0 (d, *J* = 2.4 Hz), 34.2, 20.3; ^{19}F NMR (470 MHz, $CDCl_3$): δ = –114.2; IR (ATR): ṽ = 3031, 1732, 1588, 1443, 1239, 1217, 1144, 1096, 1048, 806, 742, 711, 692, 571, 524; HRMS (EI) for $C_{18}H_{14}O_2FCl_3$ $[M^+]^+$: calcd: 386.00379, found: 386.00412.

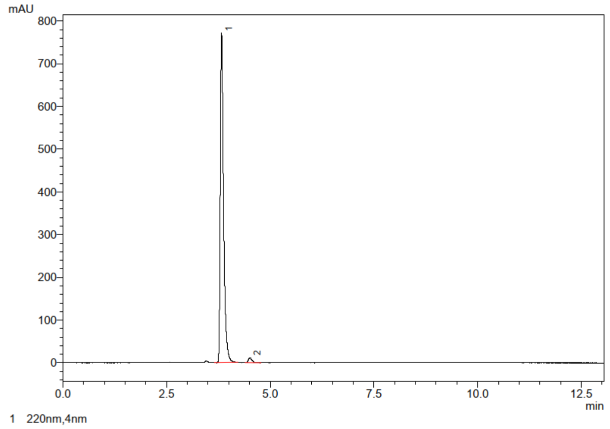

PDA Ch1 220nm

Peak #	Ret. Time	Area %	Name
1	3.83	98.37	1. Enantiomer, ee=97%
2	4.51	1.63	2. Enantiomer
Total		100.00	

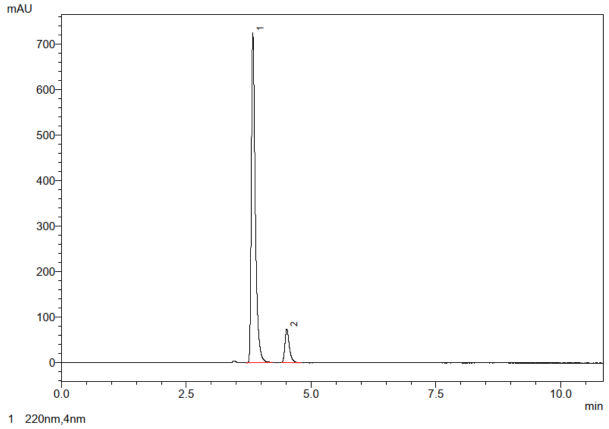

PDA Ch1 220nm

Peak #	Ret. Time	Area %	Name
1	3.83	89.90	1. Enantiomer, ee=80%
2	4.51	10.10	2. Enantiomer
Total		100.00	

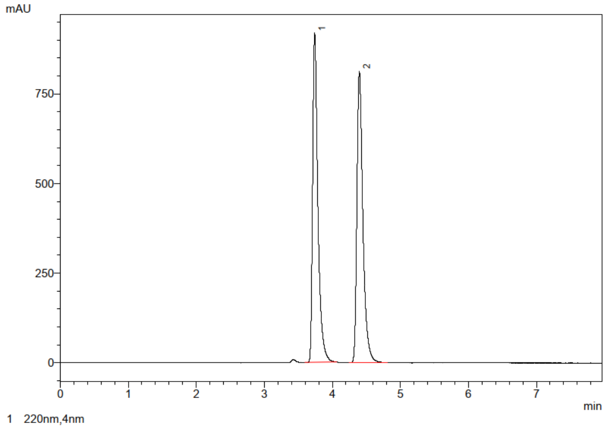

PDA Ch1 220nm

Peak #	Ret. Time	Area %	Name
1	3.74	49.86	1. Enantiomer
2	4.40	50.14	2. Enantiomer
Total		100.00	

Figure S28. HPLC traces of compound **S18**: with complex **3b** (top, left); with complex **2a** (top, right); the corresponding racemate (bottom).

Methyl 3-((1*S*,2*R*)-2-phenyl-1-((2,2,2-trichloroethoxy)carbonyl)cyclopropyl)benzoate (S19). Prepared according to the general procedure as a colorless oil; with complex **2a**: 99% yield, 85% *ee*; with complex **3b**: 99% yield, 98% *ee*. [The *ee* was determined by HPLC analysis: Daicel 150 mm Chiralpak IB-N-3, ∅ 4.6 mm, *n*-heptane/2-propanol = 90/10, v = 1.0 mL/min, λ = 220 nm, t(major) = 4.03 min, t(minor) = 5.26 min.] $[\alpha]_D^{20}$ = +17.8 (c = 1.2, $CHCl_3$); ^{1}H NMR (400 MHz, $CDCl_3$): δ = 7.86 (dt, *J* = 1.7, 1.0 Hz, 1H), 7.81 (ddd, *J* = 6.0, 2.8, 1.7 Hz, 1H), 7.19 – 7.13 (m, 2H), 7.10 – 7.03 (m, 3H), 6.85 – 6.76 (m, 2H), 4.84 (d, *J* = 11.9 Hz, 1H), 4.63 (d, *J* = 11.9 Hz, 1H), 3.87 (s, 3H), 3.26 (dd, *J* = 9.4, 7.4 Hz, 1H), 2.32 (dd, *J* = 9.4, 5.3 Hz, 1H), 2.08 (dd, *J* = 7.4, 5.3 Hz, 1H); ^{13}C NMR (101 MHz, $CDCl_3$): δ = 171.8, 167.0, 137.1, 135.3, 134.5, 132.9, 129.9, 128.8, 128.3, 128.1, 127.9, 127.0, 95.1, 74.6, 52.2, 37.0, 34.2, 20.2; IR (ATR): ṽ = 2952, 1719, 1435, 1287, 1261, 1238, 1207, 1151, 1098, 1052, 806, 778, 749, 714, 698, 572; HRMS (ESI$^+$) for $C_{20}H_{17}O_4Cl_3Na$ [M+Na$^+$]$^+$: calcd: 449.00846, found: 449.00893.

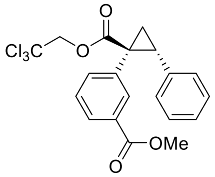

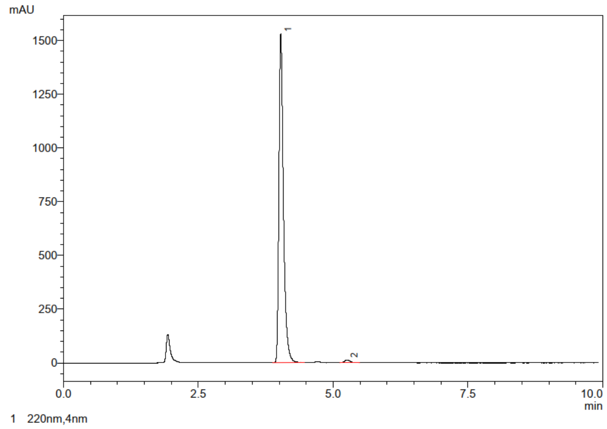

PDA Ch1 220nm

Peak #	Ret. Time	Area %	Name
1	4.03	98.91	1. Enantiomer ee=98%
2	5.26	1.09	2. Enantiomer
Total		100.00	

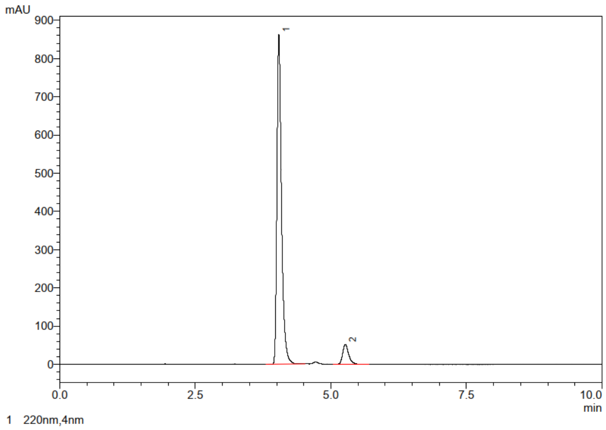

PDA Ch1 220nm

Peak #	Ret. Time	Area %	Name
1	4.04	92.63	1. Enantiomer, ee=85%
2	5.27	7.37	2. Enantiomer
Total		100.00	

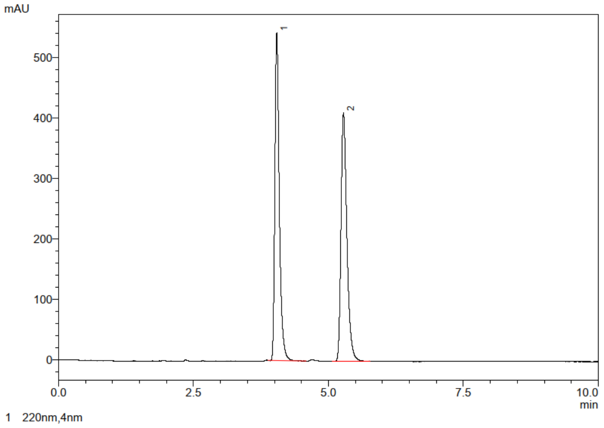

PDA Ch1 220nm

Peak #	Ret. Time	Area %	Name
1	4.04	49.86	1. Enantiomer
2	5.28	50.14	2. Enantiomer
Total		100.00	

Figure S29. HPLC traces of compound **S19**: with complex **3b** (top, left); with complex **2a** (top, right); the corresponding racemate (bottom).

2,2,2-Trichloroethyl (1*S*,2*R*)-1-(2-chloropyrimidin-5-yl)-2-phenylcyclopropane-1-carboxylate (S20).

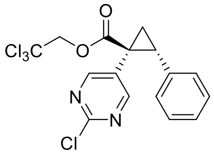

Prepared according to the general procedure at room temperature as a colorless oil; with complex **2a**: 82% yield, 91% *ee*; with complex **3b**: 87% yield, 96% *ee*. [The *ee* was determined by HPLC analysis: Daicel 150 mm Chiralpak IA-3, ∅ 4.6 mm, *n*-heptane/2-propanol = 90/10, v = 1.0 mL/min, λ = 220 nm, t(major) = 7.01 min, t(minor) = 5.39 min.] $[\alpha]_D^{20}$ = −3.5 (c = 1.0, $CHCl_3$); ^1H NMR (400 MHz, $CDCl_3$): δ = 8.29 (s, 2H), 7.23 – 7.12 (m, 3H), 6.91 – 6.84 (m, 2H), 4.85 (d, *J* = 11.9 Hz, 1H), 4.68 (d, *J* = 11.9 Hz, 1H), 3.35 (dd, *J* = 9.4, 7.6 Hz, 1H), 2.41 (dd, *J* = 9.4, 5.6 Hz, 1H), 2.12 – 2.02 (m, 1H); ^{13}C NMR (101 MHz, $CDCl_3$): δ = 170.0, 162.2, 160.1, 133.4, 128.8, 128.1, 128.0, 127.2, 94.6, 74.6, 33.9, 31.6, 18.8; IR (ATR): ṽ = 2956, 1727, 1540, 1403, 1244, 1144, 1109, 1082, 1057, 782, 766, 710, 639, 574, 494; HRMS (ESI^+) for $C_{16}H_{12}N_2O_2Cl_4Na$ $[M+Na^+]^+$: calcd: 426.95451, found: 426.95493.

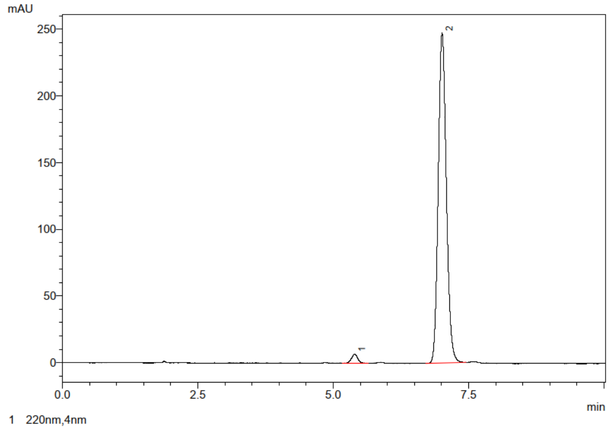

PDA Ch1 220nm

Peak #	Ret. Time	Area %	Name
1	5.39	2.10	1. Enantiomer
2	7.01	97.90	2. Enantiomer, ee=96%
Total		100.00	

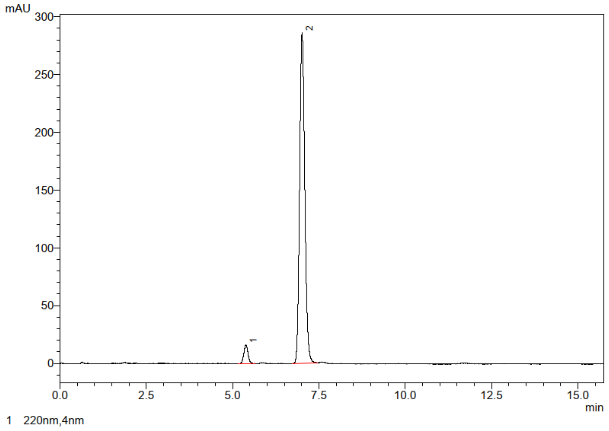

PDA Ch1 220nm

Peak #	Ret. Time	Area %	Name
1	5.39	4.40	1. Enantiomer
2	7.01	95.60	2. Enantiomer, ee=91%
Total		100.00	

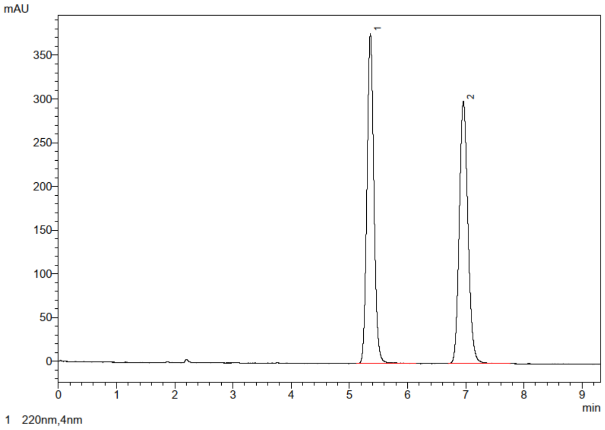

PDA Ch1 220nm

Peak #	Ret. Time	Area %	Name
1	5.36	50.03	1. Enantiomer
2	6.96	49.97	2. Enantiomer
Total		100.00	

Figure S30. HPLC traces of compound **S20**: with complex **3b** (top, left); with complex **2a** (top, right); the corresponding racemate (bottom).

2,2,2-Trichloroethyl (1*S*,2*R*)-2-phenyl-1-(thiophen-3-yl)cyclopropane-1-carboxylate (S21). Prepared according to the general procedure as a colorless sticky solid; with complex **2a**: 97% yield, 73% *ee*; with complex **3b**: 99% yield, 94% *ee.* [The *ee* was determined by HPLC analysis: Daicel 150 mm Chiralpak IB-N-3, ∅ 4.6 mm, *n*-heptane/i-propanol = 99/1, v = 1.0 mL/min, λ = 220 nm, t(major) = 5.01 min, t(minor) = 9.63 min.] $[\alpha]_D^{20}$ = +4.6 (c = 3.7, $CHCl_3$); ^{1}H NMR (400 MHz, $CDCl_3$): δ = 7.14 – 7.09 (m, 3H), 7.03 (dd, *J* = 5.0, 3.0 Hz, 1H), 6.97 (dd, *J* = 3.0, 1.3 Hz, 1H), 6.89 (dd, *J* = 6.6, 3.0 Hz, 2H), 6.75 (dt, *J* = 5.0, 1.1 Hz, 1H), 4.87 (dd, *J* = 11.9, 0.8 Hz, 1H), 4.68 (d, *J* = 11.9 Hz, 1H), 3.18 (dd, *J* = 9.4, 7.5 Hz, 1H), 2.28 (dd, *J* = 9.4, 5.1 Hz, 1H), 2.02 (dd, *J* = 7.4, 5.1 Hz, 1H); ^{13}C NMR (101 MHz, $CDCl_3$): δ = 171.8, 135.7, 134.7, 130.4, 128.2, 128.0, 126.9, 125.8, 124.6, 95.2, 74.5, 34.4, 32.4, 20.6; IR (ATR): ṽ = 1730, 1604, 1454, 1433, 1377, 1245, 1213, 1197, 1144, 1095, 1053, 975, 818, 790, 765, 693, 645, 571, 535 cm^{-1}; HRMS (ESI$^+$) for $C_{16}H_{13}Cl_3O_2SNa$ [M+Na]$^+$: calcd: 396.95941, found: 396.95900.

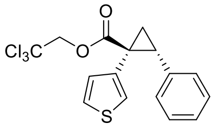

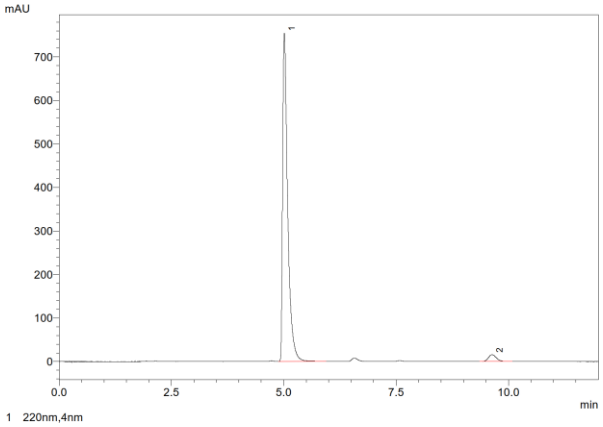

PDA Ch1 220nm

Peak #	Ret. Time	Area %
1	5.01	96.81
2	9.63	3.19
Total		100.00

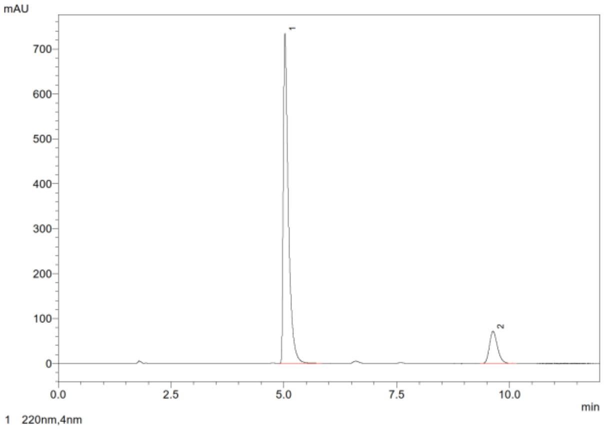

PDA Ch1 220nm

Peak #	Ret. Time	Area %
1	5.02	86.59
2	9.64	13.41
Total		100.00

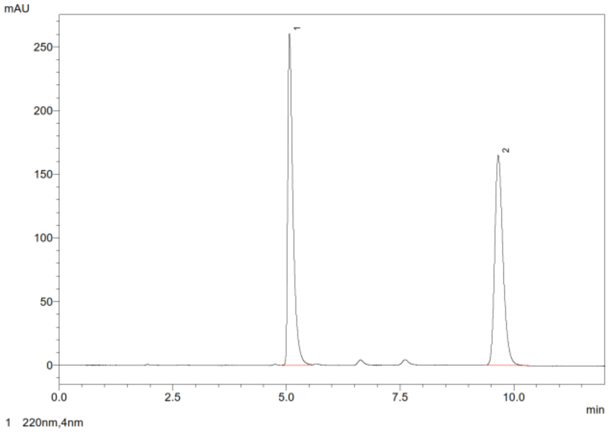

PDA Ch1 220nm

Peak #	Ret. Time	Area %
1	5.07	50.00
2	9.66	50.00
Total		100.00

Figure S31. HPLC traces of compound **S21**: with complex **3b** (top, left); with complex **2a** (top, right); the corresponding racemate (bottom).

2,2,2-Trichloroethyl (1*S*,2*R*)-1-(naphthalen-2-yl)-2-phenylcyclopropane-1-carboxylate (*S*22). Prepared according to the general procedure as a white solid; with complex **2a**: 85% yield, 96% *ee*; with complex **3b**: 91% yield, 99% *ee*. [The *ee* was determined by HPLC analysis: Daicel 150 mm Chiralpak IA-3, ∅ 4.6 mm, *n*-heptane/i-propanol = 98/2, v = 1.0 mL/min, λ = 220 nm, t(minor) = 3.89 min, t(major) = 4.29 min]. m. p. = 100-102°C; $[\alpha]_D^{20}$ = –40.2 (c = 3.5, $CHCl_3$); ^{1}H NMR (400 MHz, $CDCl_3$): δ = 7.78 – 7.65 (m, 3H), 7.55 (d, *J* = 8.5 Hz, 1H), 7.42 (dt, *J* = 6.3, 3.4 Hz, 2H), 7.11 (dt, *J* = 8.6, 1.6 Hz, 1H), 7.06 – 6.98 (m, 3H), 6.91 – 6.78 (m, 2H), 4.88 (dd, *J* = 11.9, 1.2 Hz, 1H), 4.63 (dd, *J* = 12.0, 1.0 Hz, 1H), 3.30 (dd, *J* = 9.2, 7.6 Hz, 1H), 2.38 (ddd, *J* = 9.4, 5.1, 1.1 Hz, 1H), 2.21 – 2.10 (m, 1H); ^{13}C NMR (101 MHz, $CDCl_3$): δ =172.3, 135.7, 133.1, 132.7, 131.7, 130.9, 130.2, 128.3, 128.0, 127.9, 127.7, 127.2, 126.8, 126.0, 125.8, 95.2, 74.5, 37.5, 34.2, 20.5; IR (ATR): ṽ = 1723, 1448, 1376, 1245, 1150, 1129, 1100, 1082, 1054, 979, 966, 908, 866, 834, 800, 748, 715, 964, 652, 575, 546, 478 cm^{-1}; HRMS (ESI$^+$) for $C_{22}H_{17}Cl_3O_2Na$ [M+Na]$^+$: calcd: 441.01863, found: 441.01828.

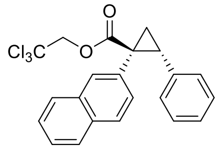

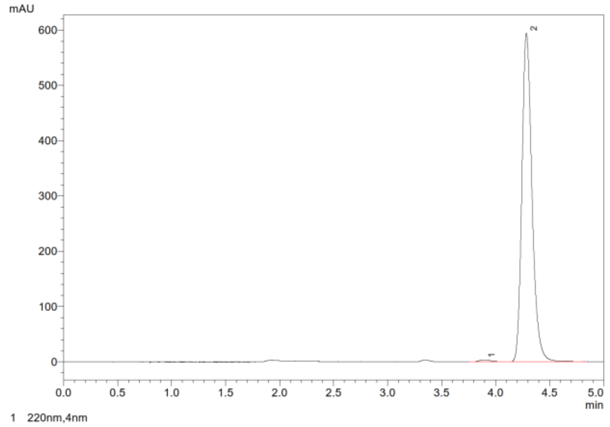

PDA Ch1 220nm

Peak #	Ret. Time	Area %
1	3.89	0.72
2	4.29	99.28
Total		100.00

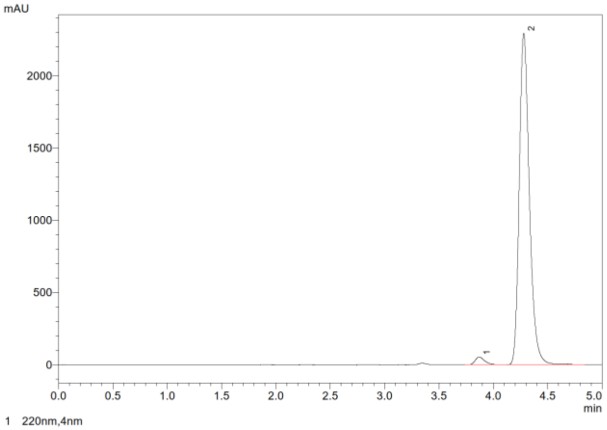

PDA Ch1 220nm

Peak #	Ret. Time	Area %
1	3.87	2.21
2	4.28	97.79
Total		100.00

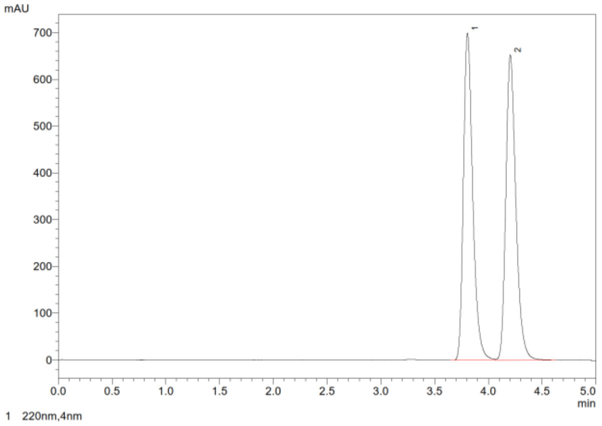

PDA Ch1 220nm

Peak #	Ret. Time	Area %
1	3.81	49.95
2	4.20	50.05
Total		100.00

Figure S32. HPLC traces of compound **S22**: with complex **3b** (top, left); with complex **2a** (top, right); the corresponding racemate (bottom).

2,2,2-Trichloroethyl (*S*)-1-(4-fluorophenyl)spiro[2.3]hexane-1-carboxylate (S23). Prepared according to the general procedure as a colorless oil; with complex **2a**: 85% yield, 70% *ee*; with complex **3b**: 95% yield, 96% *ee*. [The *ee* was determined by HPLC analysis: Daicel 150 mm Chiralpak OJ-3, ∅ 4.6 mm, *n*-heptane/2-propanol = 98/2, v = 1.0 mL/min, λ = 220 nm, t(major) = 6.14 min, t(minor) = 4.33 min.] $[\alpha]_D^{20}$ = −9.4 (c = 1.1, $CHCl_3$); ^1H NMR (400 MHz, $CDCl_3$): δ = 7.35 – 7.23 (m, 2H), 7.07 – 6.96 (m, 2H), 4.87 (dd, *J* = 11.9, 1.1 Hz, 1H), 4.51 (d, *J* = 1.2 Hz, 1H), 2.51 – 2.39 (m, 1H), 2.39 – 2.25 (m, 1H), 2.16 – 2.02 (m, 2H), 2.01 – 1.89 (m, 2H), 1.67 – 1.56 (m, 1H), 1.55 – 1.48 (m, 1H); ^{13}C NMR (101 MHz, $CDCl_3$): δ = 170.9, 162.1 (d, *J* = 245.7 Hz), 132.7 (d, *J* = 8.2 Hz), 131.9 (d, *J* = 3.3 Hz), 115.1 (d, *J* = 21.5 Hz), 95.1, 74.4, 37.4, 36.5, 29.5, 28.5, 27.8, 16.0; ^{19}F NMR (282 MHz, $CDCl_3$): δ = –115.2; IR (ATR): ṽ = 2952, 1730, 1512, 1312, 1179, 1092, 1047, 836, 806, 751, 719, 573, 535; HRMS (EI) for $C_{15}H_{14}O_2FCl_3$ $[M]^+$: calcd: 350.00379, found: 350.00410.

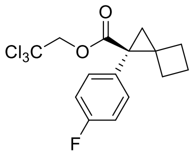

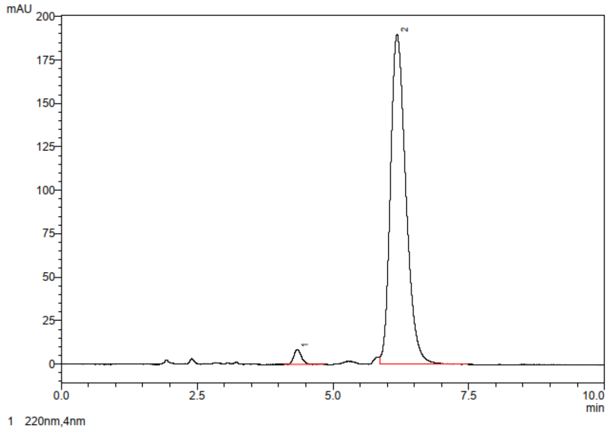

PDA Ch1 220nm

Peak #	Ret. Time	Area %	Name
1	4.35	2.16	1. Enantiomer
2	6.18	97.84	2. Enantiomer, ee=96%
Total		100.00	

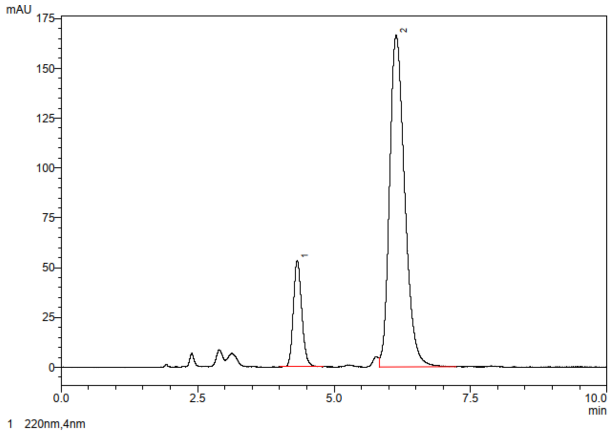

PDA Ch1 220nm

Peak #	Ret. Time	Area %	Name
1	4.33	15.12	1. Enantiomer
2	6.14	84.88	2. Enantiomer, ee=70%
Total		100.00	

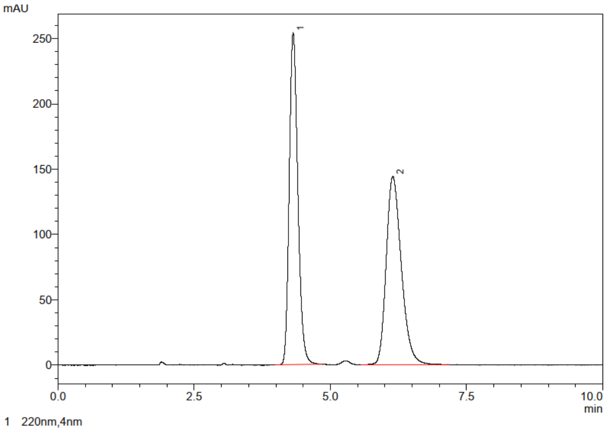

PDA Ch1 220nm

Peak #	Ret. Time	Area %	Name
1	4.32	50.10	1. Enantiomer
2	6.15	49.90	2. Enantiomer
Total		100.00	

Figure S33. HPLC traces of compound **S23**: with complex **3b** (top, left); with complex **2a** (top, right); the corresponding racemate (bottom).

2,2,2-Trichloroethyl (*S*)-1-(4-fluorophenyl)spiro[2.5]octane-1-carboxylate (S24). Prepared according to the general procedure as a colorless oil; with complex **2a**: 80% yield, 74% *ee*; with complex **3b**: 87% yield, 98% *ee*. [The *ee* was determined by HPLC analysis: Daicel 150 mm Chiralpak IA-3, ∅ 4.6 mm, *n*-heptane/2-propanol = 99.9/0.1, v = 1.0 mL/min, λ = 220 nm, t(major) = 4.94 min, t(minor) = 6.10 min.] $[\alpha]_D^{20}$ = −42.5 (c = 1.1, $CHCl_3$); ^1H NMR (400 MHz, $CDCl_3$): δ = 7.42 – 7.34 (m, 2H), 7.02 – 6.93 (m, 2H), 4.77 (d, *J* = 12.0 Hz, 1H), 4.54 (d, *J* = 11.9 Hz, 1H), 1.77 – 1.40 (m, 7H), 1.39 – 1.29 (m, 3H), 1.17 (dd, *J* = 4.9, 1.0 Hz, 1H), 0.70 (dt, *J* = 10.2, 3.2 Hz, 1H); ^{13}C NMR (101 MHz, $CDCl_3$): δ = 170.3, 162.1 (d, *J* = 245.9 Hz), 133.2 (d, *J* = 8.1 Hz), 132.4 (d, *J* = 3.5 Hz), 114.8 (d, *J* = 21.1 Hz), 95.0, 74.7, 39.6, 34.8, 34.1, 31.0, 26.2, 25.9, 25.5, 24.7; ^{19}F NMR (282 MHz, $CDCl_3$): δ = –115.3; IR (ATR): ṽ = 2929, 1732, 1510, 1225, 1167, 1119, 1051, 837, 804, 756, 718, 575; HRMS (EI) for $C_{17}H_{18}O_2FCl_3$ $[M]^+$: calcd: 378.03509, found: 378.03532.

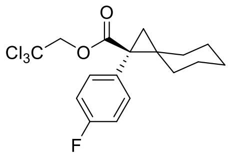

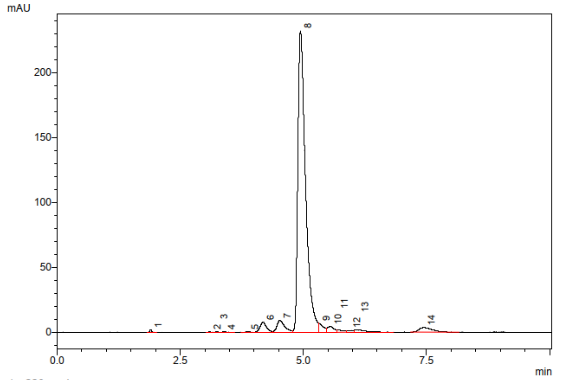

1 220nm,4nm

PDA Ch1 220nm

Peak #	Ret. Time	Area %	Name
1	1.90	0.17	
2	3.10	0.04	
3	3.24	0.07	
4	3.39	0.08	
5	3.87	0.12	
6	4.18	2.55	
7	4.52	3.50	
8	4.94	86.07	1. Enantiomer
9	5.32	1.63	
10	5.55	1.39	
11	5.68	0.59	
12	5.93	0.43	
13	6.10	1.04	2. Enantiomer
14	7.45	2.32	
Total		100.00	

ee = 97.6 %

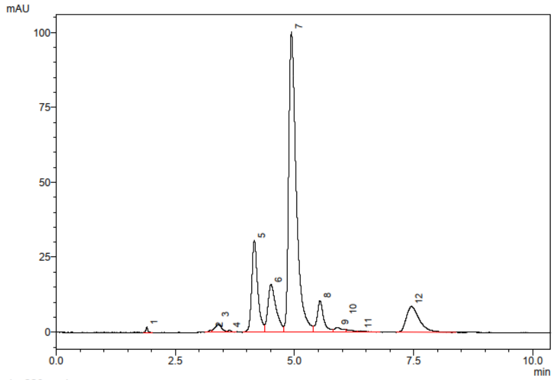

1 220nm,4nm

PDA Ch1 220nm

Peak #	Ret. Time	Area %	Name
1	1.90	0.25	
2	3.27	0.18	
3	3.40	1.40	
4	3.63	0.19	
5	4.16	14.10	
6	4.51	10.10	
7	4.94	58.18	1. Enantiomer
8	5.54	5.32	
9	5.90	0.97	
10	6.08	0.44	
11	6.39	0.30	
12	7.46	8.59	2. Enantiomer
Total		100.00	

ee = 74.3 %

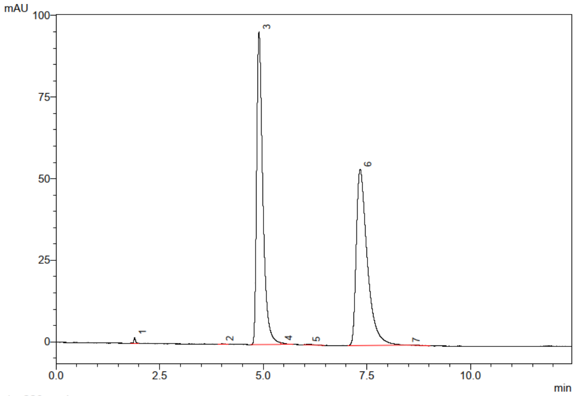

1 220nm,4nm

PDA Ch1 220nm

Peak #	Ret. Time	Area %	Name
1	1.90	0.24	
2	4.01	0.06	
3	4.90	49.63	1. Enantiomer
4	5.43	0.21	
5	6.09	0.10	
6	7.34	49.71	2. Enantiomer
7	8.51	0.05	
Total		100.00	

Figure S34. HPLC traces of compound **S24**: with complex **3b** (top, left); with complex **2a** (top, right); the corresponding racemate (bottom).

1-((1*S*,2*R*)-1,2-diphenylcyclopropyl)ethan-1-one (S25). Prepared according to the general procedure as a colorless oil; with complex **2a**: 25% yield, 78% *ee*; with complex **3b**: 68% yield, 91% *ee*. [The *ee* was determined by HPLC analysis: Daicel 150 mm Chiralcel OJ-3, ∅ 4.6 mm, *n*-heptane/i-propanol = 98/2, v = 1.0 mL/min, λ = 220 nm, t(minor) = 4.65 min, t(major) = 6.49 min.] $[\alpha]_D^{20}$ = +136.5 (c = 1.1, $CHCl_3$); ^1H NMR (400 MHz, $CDCl_3$): δ = 7.19 – 7.15 (m, 3H), 7.10 – 6.98 (m, 5H), 6.79 – 6.73 (m, 2H), 3.15 (dd, *J* = 9.2, 7.3 Hz, 1H), 2.11 (dd, *J* = 9.2, 4.3 Hz, 1H), 2.02 (s, 3H), 1.82 (dd, *J* = 7.3, 4.3 Hz, 1H); ^{13}C NMR (101 MHz, $CDCl_3$): δ = 208.0, 137.0, 136.7, 132.2, 128.3, 128.1, 127.8, 127.4, 126.4, 46.1, 35.1, 30.1, 23.6; IR (ATR): ṽ = 1687, 1600, 1496, 1419, 1372, 1354, 1240, 1207, 1148, 981, 882, 756, 696, 677, 644, 544, 509 cm^{-1}; HRMS (ESI^+) for $C_{17}H_{16}ONa$ $[M+Na]^+$: calcd: 259.10933, found: 259.10924.

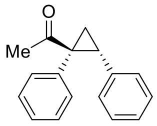

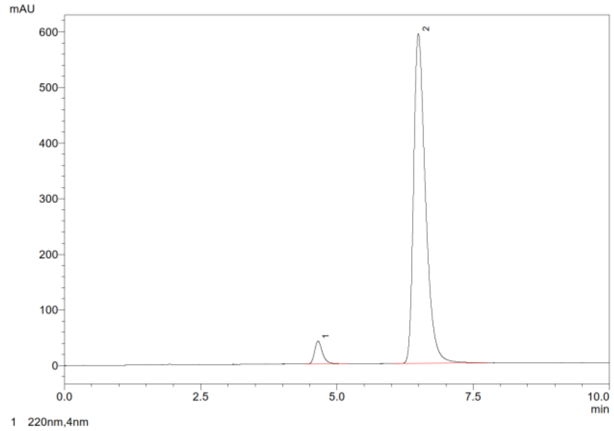

PDA Ch1 220nm

Peak #	Ret. Time	Area %
1	4.65	4.36
2	6.49	95.64
Total		100.00

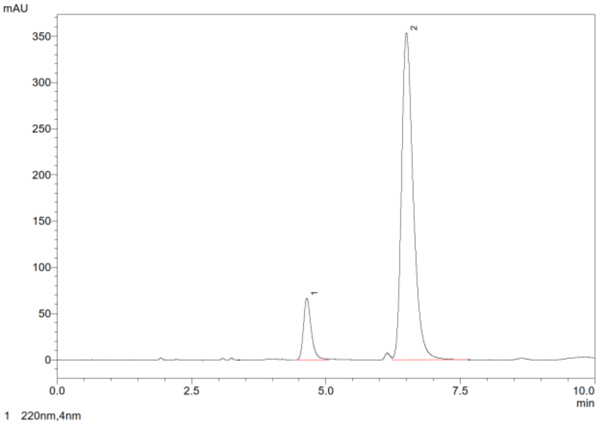

PDA Ch1 220nm

Peak #	Ret. Time	Area %
1	4.64	11.12
2	6.50	88.88
Total		100.00

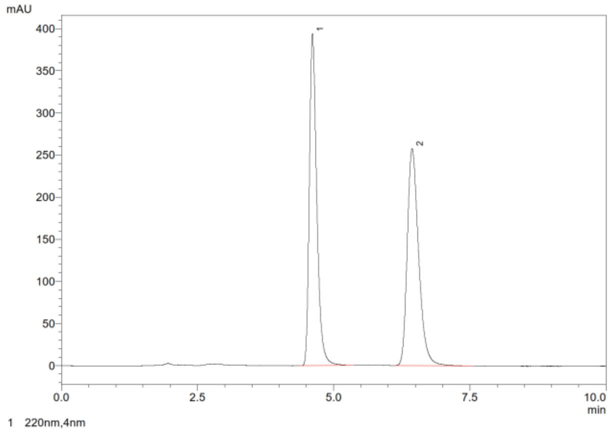

PDA Ch1 220nm

Peak #	Ret. Time	Area %
1	4.61	50.03
2	6.44	49.97
Total		100.00

Figure S35. HPLC traces of compound **S25**: with complex **3b** (top, left); with complex **2a** (top, right); the corresponding racemate (bottom).

1-((1*S*,2*R*)-1-(4-bromophenyl)-2-phenylcyclopropyl)ethan-1-one (S26). Prepared according to the general procedure as a colorless oil; with complex **2a**: 34% yield, 89% *ee*; with complex **3b**: 75% yield, 92% *ee*. [The *ee* was determined by HPLC analysis: Daicel 150 mm Chiralcel OJ-3, ∅ 4.6 mm, *n*-heptane/i-propanol = 98/2, v = 1.0 mL/min, λ = 220 nm, t(minor) = 5.32 min, t(major) = 10.74 min.] $[\alpha]_D^{20}$ = +44.6 (c = 2.1, $CHCl_3$); ^1H NMR (400 MHz, $CDCl_3$): δ = 7.34 – 7.27 (m, 2H), 7.13 – 7.05 (m, 3H), 6.93 – 6.87 (m, 2H), 6.79 – 6.72 (m, 2H), 3.14 (dd, *J* = 9.2, 7.3 Hz, 1H), 2.12 (dd, *J* = 9.2, 4.4 Hz, 1H), 2.00 (s, 3H), 1.78 (dd, *J* = 7.3, 4.4 Hz, 1H); ^{13}C NMR (101 MHz, $CDCl_3$): δ = 207.0, 136.4, 135.9, 133.8, 131.6, 128.1, 128.1, 126.7, 121.7, 45.4, 35.1, 30.0, 23.5; IR (ATR): ṽ = 1687, 1604, 1485, 1445, 1420, 1395, 1354, 1236, 1207, 1147, 1084, 1071, 1010, 982, 836, 773, 747, 695, 613, 541 cm^{-1}; HRMS (ESI^+) for $C_{17}H_{15}BrONa$ $[M+Na]^+$: calcd: 337.01986, found: 337.01954.

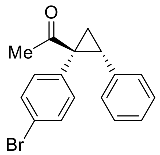

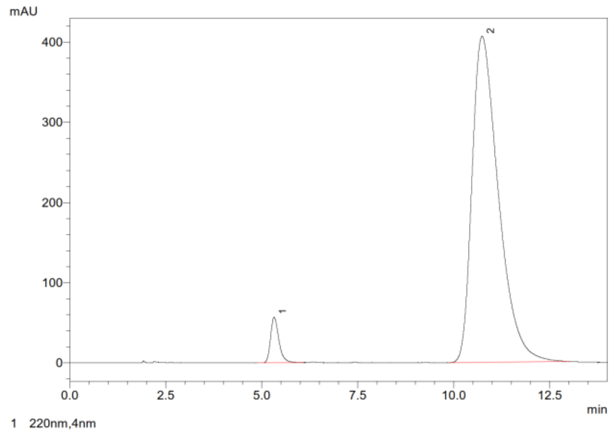

PDA Ch1 220nm

Peak #	Ret. Time	Area %
1	5.32	4.22
2	10.74	95.78
Total		100.00

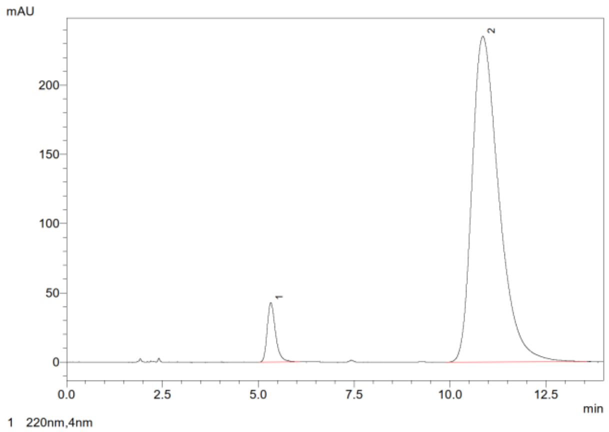

PDA Ch1 220nm

Peak #	Ret. Time	Area %
1	5.33	5.33
2	10.86	94.67
Total		100.00

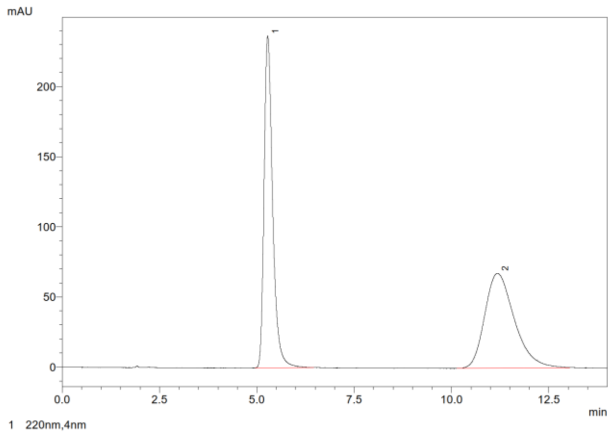

PDA Ch1 220nm

Peak #	Ret. Time	Area %
1	5.28	50.27
2	11.18	49.73
Total		100.00

Figure S36. HPLC traces of compound **S26**: with complex **3b** (top, left); with complex **2a** (top, right); the corresponding racemate (bottom).

((1*R*,2*R*)-1-(4-Methoxyphenyl)cyclopropane-1,2-diyl)dibenzene (*S*27). Prepared at room temperature according to the general procedure as a white solid; with complex **3b**: 68% yield, 99% *ee*. [The *ee* was determined by HPLC analysis: Daicel 150 mm Chiralpak IG-3, Ø 4.6 mm, n-heptane/2-propanol = 98/2, v = 1.0 mL/min, λ = 230 nm, t(minor) = 2.93 min, t(major) = 3.49 min]. m. p. = 55-58°C; $[\alpha]_D^{20}$ = +100.9 (c = 0.8, $CHCl_3$); ^1H NMR (400 MHz, $CDCl_3$): δ = 7.28 – 7.26 (m, 4H), 7.20 – 7.03 (m, 4H), 7.03 – 6.98 (m, 2H), 6.90 – 6.82 (m, 2H), 6.70 – 6.62 (m, 2H), 3.71 (s, 3H), 2.81 (dd, *J* = 9.0, 6.6 Hz, 1H), 1.93 (dd, *J* = 6.6, 5.3 Hz, 1H), 1.80 (dd, *J* = 9.0, 5.3 Hz, 1H); ^{13}C NMR (101 MHz, $CDCl_3$): δ = 158.1, 147.6, 139.0, 132.5, 132.4, 128.5, 128.1, 127.8, 127.3, 125.9, 125.7, 113.5, 55.2, 38.7, 32.8, 21.4; IR (ATR): ṽ = 3001, 1601, 1510, 1444, 1290, 1242, 1174, 1030, 843, 819, 722, 759, 727, 694, 551, 514; HRMS (EI^+) for $C_{22}H_{20}O$ $[M]^+$: calcd: 300.15087, found: 300.15123.

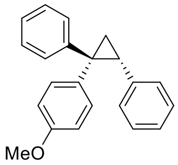

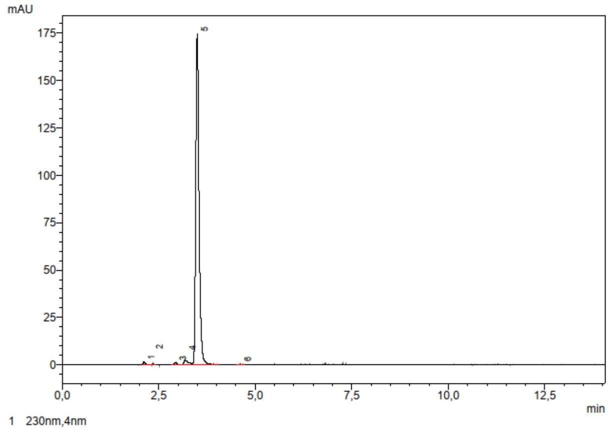

PDA Ch1 230nm

Peak #	Ret. Time	Area %	Name
1	2,11	0,52	
2	2,33	0,13	
3	2,93	0,52	1. Enantiomer
4	3,18	1,79	
5	3,49	96,93	2. Enantiomer 98.9 % ee
6	4,61	0,11	
Total		100,00	

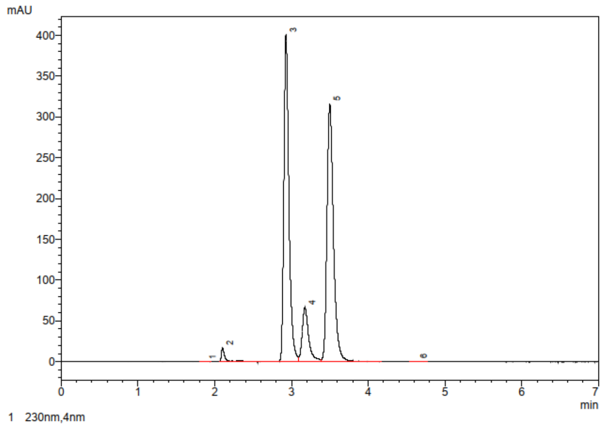

PDA Ch1 230nm

Peak #	Ret. Time	Area %	Name
1	1,88	0,03	
2	2,10	1,50	
3	2,93	44,14	1. Enantiomer
4	3,18	9,38	
5	3,50	44,84	2. Enantiomer
6	4,63	0,11	
Total		100,00	

Figure S37. HPLC traces of compound **S27**: with complex **3b** (left); the corresponding racemate (right).

(*R*)-4,4'-(2-Phenylcyclopropane-1,1-diyl)bis(methoxybenzene) (S28). Prepared at room temperature according to the general procedure in CH_2Cl_2/pentane = 1/9 as a white solid; with complex **3b**: 92% yield, 97% *ee*. [The *ee* was determined by HPLC analysis: Daicel 150 mm Chiralpak IA-3, Ø 4.6 mm, n-heptane/2-propanol = 98/2, v = 1.0 mL/min, λ = 220 nm, t(minor) = 4.27 min, t(major) = 4.66 min]. m. p. = 127-131°C; $[\alpha]_D^{20}$ = +105.2 (c = 1, $CHCl_3$); ^1H NMR (400 MHz, $CDCl_3$): δ = 7.25 – 7.16 (m, 2H), 7.14 – 7.02 (m, 3H), 7.02 – 6.96 (m, 2H), 6.89 – 6.84 (m, 2H), 6.84 – 6.79 (m, 2H), 6.69 – 6.61 (m, 2H), 3.77 (s, 3H), 3.70 (s, 3H), 2.76 (dd, *J* = 9.0, 6.5 Hz, 1H), 1.88 (dd, *J* = 6.5, 5.2 Hz, 1H), 1.73 (dd, *J* = 9.0, 5.3 Hz, 1H); ^{13}C NMR (101 MHz, $CDCl_3$): δ = 158.0, 157.9, 139.9, 139.2, 133.0, 132.1, 128.5, 128.1, 127.8, 125.6, 113.9, 113.5, 55.5, 55.2, 38.1, 32.4, 21.1; IR (ATR): ṽ = 2957, 1716, 1605, 1509, 1455, 1290, 1241, 1173, 1022, 836, 814, 744, 736, 696, 603, 559, 546, 511; HRMS (EI^+) for $C_{23}H_{22}O_2$ $[M]^+$: calcd: 330.16143, found: 330.16114.

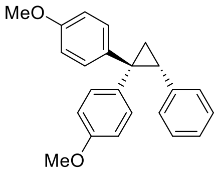

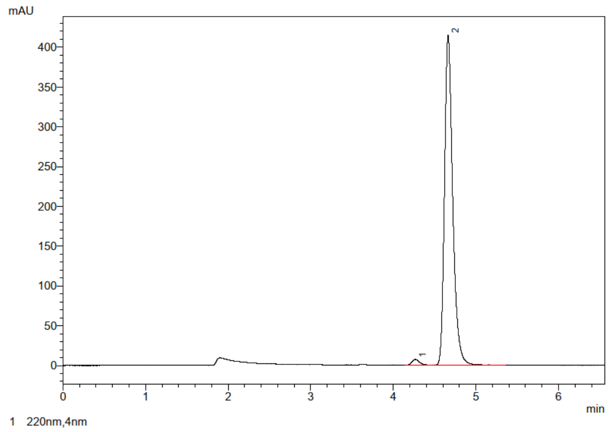

PDA Ch1 220nm

Peak #	Ret. Time	Area %	Name
1	4.27	1.55	1. Enantiomer
2	4.66	98.45	2. Enantiomer, ee=97%
Total		100.00	

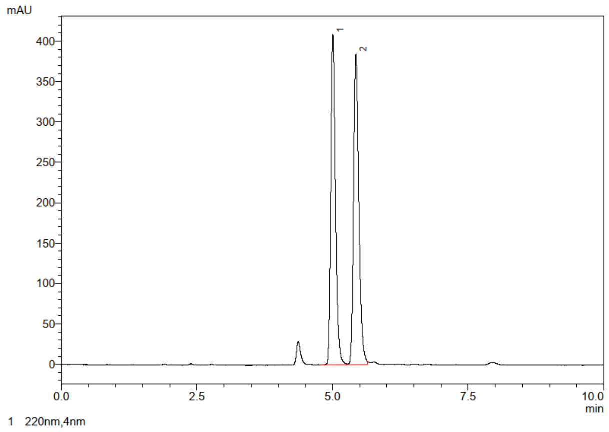

PDA Ch1 220nm

Peak #	Ret. Time	Area %	Name
1	5.01	48.53	1. Enantiomer
2	5.43	51.47	2. Enantiomer
Total		100.00	

Figure S38. HPLC traces of compound **S28**: with complex **3b** (left); the corresponding racemate (right).

1-Methoxy-4-((1*R*,2*R*)-1-(4-nitrophenyl)-2-phenylcyclopropyl)benzene (*S*29). Prepared at room temperature according to the general procedure in CH_2Cl_2 as a white solid; with complex **3b**: 90% yield, 95% *ee*. [The *ee* was determined by HPLC analysis: Daicel 150 mm Chiralpak IA-3, Ø 4.6 mm, n-heptane/2-propanol = 95/5, v = 1.0 mL/min, λ = 220 nm, t(minor) = 5.15 min, t(major) = 6.19 min]. m. p. = 103-104°C; $[\alpha]_D^{20}$ = +199 (c = 1.9, $CHCl_3$); ^{1}H NMR (400 MHz, $CDCl_3$): δ = 8.14 – 8.06 (m, 2H), 7.35 – 7.26 (m, 2H), 7.18 – 7.03 (m, 3H), 7.01 – 6.90 (m, 2H), 6.91 – 6.79 (m, 2H), 6.74 – 6.66 (m, 2H), 3.73 (s, 3H), 2.85 (dd, *J* = 9.1, 6.8 Hz, 1H), 2.08 (dd, *J* = 6.9, 5.6 Hz, 1H), 1.89 (dd, *J* = 9.2, 5.7 Hz, 1H); ^{13}C NMR (101 MHz, $CDCl_3$): δ = 158.6, 155.2, 145.9, 137.8, 132.7, 130.5, 128.0, 128.0, 127.4, 126.2, 123.7, 113.9, 55.3, 38.4, 34.5, 22.6; IR (ATR): ṽ = 2924, 1591, 1503, 1455, 1342, 1327, 1295, 1241, 1177, 1109, 1027, 965, 858, 841, 809, 773, 756, 729, 695, 613, 553, 505; HRMS (ESI$^+$) for $C_{22}H_{19}NO_3Na$ [M+Na]$^+$: calcd: 368.12571, found: 368.12568.

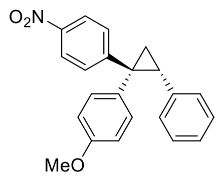

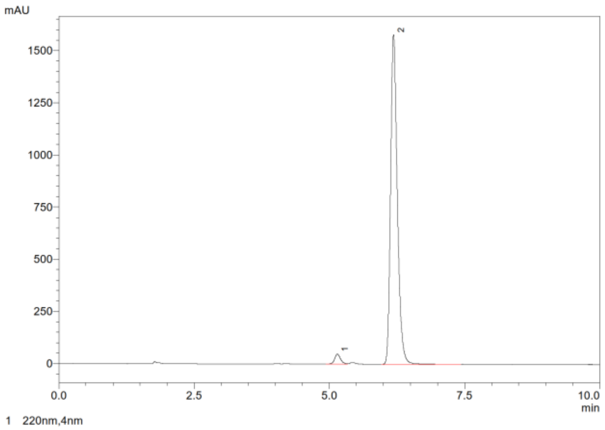

PDA Ch1 220nm

Peak #	Ret. Time	Area %
1	5.15	2.65
2	6.19	97.35
Total		100.00

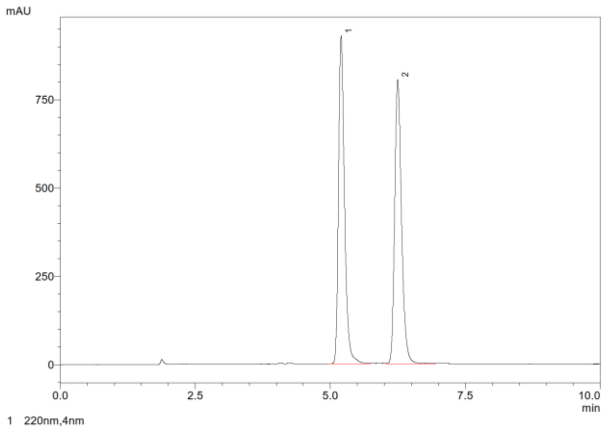

PDA Ch1 220nm

Peak #	Ret. Time	Area %
1	5.20	50.38
2	6.25	49.62
Total		100.00

Figure S39. HPLC traces of compound **S29**: with complex **3b** (left); the corresponding racemate (right).

Cyclopropenes.

2,2,2-Trichloroethyl (*S*)-2-butyl-1-(4-methoxyphenyl)cycloprop-2-ene-1-carboxylate (S30). Prepared according to the general procedure as a colorless oil: with complex **2a**: 54% yield, 96% *ee*; with complex **3b**: 82% yield, >99% *ee*.. [The *ee* was determined by HPLC analysis: Daicel 150 mm Chiralpak IC-3, Ø 4.6 mm, n-heptane/2-propanol = 98/2, v = 1.0 mL/min, λ = 220 nm, t(major) = 6.91 min, t(minor) = 9.62 min.]

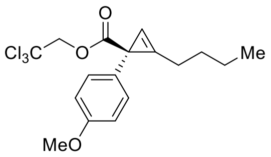

$[\alpha]_D^{20}$ = +13.1 (c = 0.8, $CHCl_3$); ^{1}H NMR (400 MHz, $CDCl_3$): δ = 7.27 – 7.20 (m, 2H), 6.88 – 6.80 (m, 2H), 6.67 (t, *J* = 1.5 Hz, 1H), 4.75 (s, 1H), 4.74 (s, 1H), 3.79 (s, 3H), 2.58 (tt, *J* = 7.2, 1.5 Hz, 2H), 1.66 – 1.58 (m, 2H), 1.45 – 1.28 (m, 2H), 0.88 (t, *J* = 7.3 Hz, 3H); ^{13}C NMR (101 MHz, $CDCl_3$): δ = 174.1, 158.3, 133.1, 129.5, 121.1, 113.6, 96.4, 95.6, 74.4, 55.4, 32.3, 29.0, 24.3, 22.4, 13.9; IR (ATR): ṽ = 2933, 2110, 1730, 1610, 1511, 1245, 1169, 1032, 836, 795, 768, 712, 573; HRMS (ESI$^+$) for $C_{17}H_{19}Cl_3O_3Na$ [M+Na$^+$]$^+$: calcd: 399.02920, found: 399.02908.

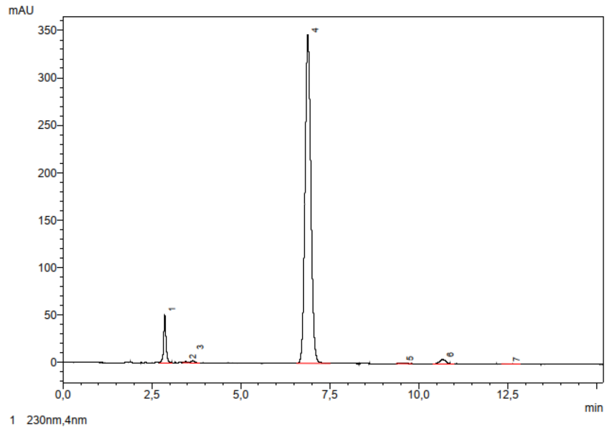

PDA Ch1 230nm

Peak #	Ret. Time	Area %	Name
1	2,86	5,49	
2	3,44	0,19	
3	3,65	0,32	
4	6,88	92,47	1. Enantiomer 99.7 % ee
5	9,55	0,16	2. Enantiomer
6	10,68	1,27	
7	12,54	0,10	
Total		100,00	

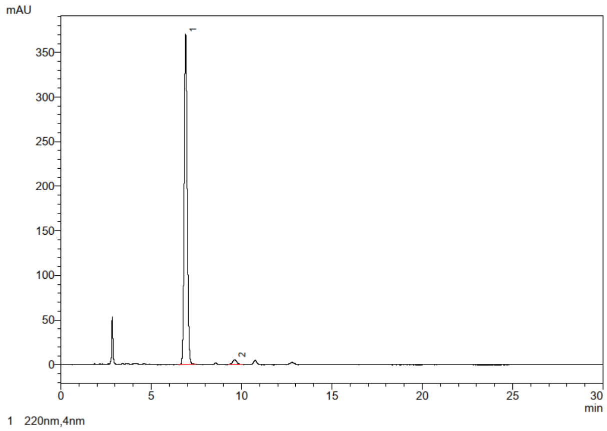

PDA Ch1 220nm

Peak #	Ret. Time	Area %	Name
1	6.91	98.07	1. Enantiomer, ee=96%
2	9.62	1.93	2. Enantiomer
Total		100.00	

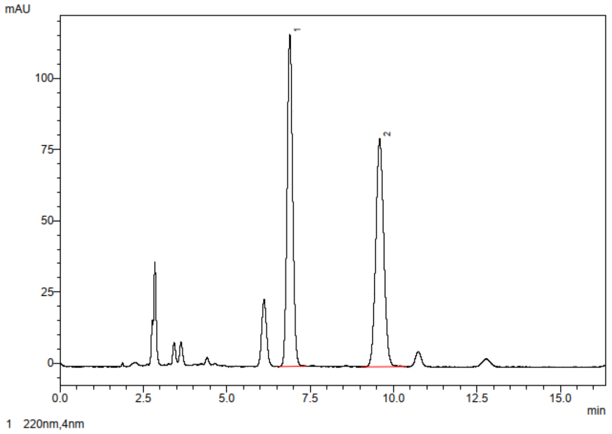

PDA Ch1 220nm

Peak #	Ret. Time	Area %	Name
1	6.89	50.24	1. Enantiomer
2	9.59	49.76	2. Enantiomer
Total		100.00	

Figure S40. HPLC traces of compound **S30**: with complex **2a** (top, left); with complex **3b** (top, right); the corresponding racemate (bottom).

2,2,2-Trichloroethyl (*R*)-1-(4-fluorophenyl)-2-((trimethylsilyl)methyl)cycloprop-2-ene-1-carboxylate (S31).

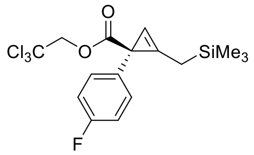

Prepared according to the general procedure as a colorless oil: with complex **2a**: 98% yield, 84% *ee*; with complex **3b**: 83% yield, 97% *ee*. [The *ee* was determined by HPLC analysis: Daicel 150 mm Chiralpak IC-3, ∅ 4.6 mm, *n*-heptane/2-propanol = 98/2, v = 1.0 mL/min, λ = 220 nm, t(major) = 2.60 min, t(minor) = 2.89 min.] $[\alpha]_D^{20}$ = +39.9 (c = 1.1, $CHCl_3$); ^1H NMR (400 MHz, $CDCl_3$): δ = 7.30 – 7.24 (m, 2H), 7.02 – 6.94 (m, 2H), 6.54 (t, *J* = 1.1 Hz, 1H), 4.80 (d, *J* = 12.0 Hz, 1H), 4.70 (d, *J* = 12.0 Hz, 1H), 2.07 – 1.90 (m, 2H), -0.03 (s, 9H); ^{13}C NMR (101 MHz, $CDCl_3$): δ = 174.0, 161.7 (d, *J* = 244.6 Hz), 136.8 (d, *J* = 3.1 Hz), 130.1 (d, *J* = 8.1 Hz), 119.7, 114.9 (d, *J* = 21.1 Hz), 95.5, 93.5, 74.4, 32.7, 14.4, –1.4; ^{19}F NMR (282 MHz, $CDCl_3$): δ = –116.6; ^{29}Si NMR (79 MHz, $CDCl_3$): δ = 2.0; IR (ATR): ṽ = 2955, 1731, 1603, 1509, 1250, 1175, 1093, 839, 769, 712, 572; HRMS (EI) for $C_{16}H_{18}O_2FCl_3Si$ $[M]^+$: calcd: 394.01202, found: 394.01170.

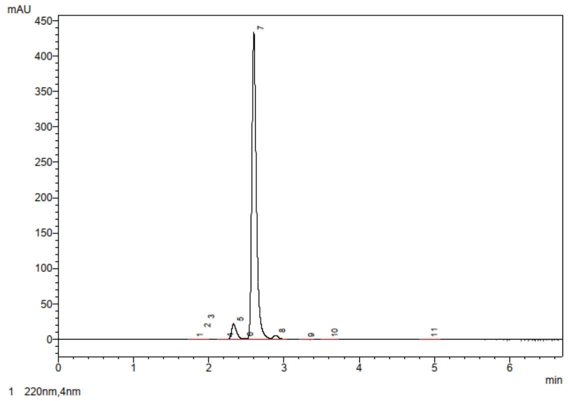

PDA Ch1 220nm

Peak #	Ret. Time	Area %	Name
1	1.79	0.11	
2	1.89	0.13	
3	1.94	0.13	
4	2.20	0.11	
5	2.33	5.24	
6	2.46	0.19	
7	2.60	92.07	1. Enantiomer 96.7 % ee
8	2.89	1.54	2. Enantiomer
9	3.27	0.17	
10	3.58	0.12	
11	4.91	0.21	
Total		100.00	

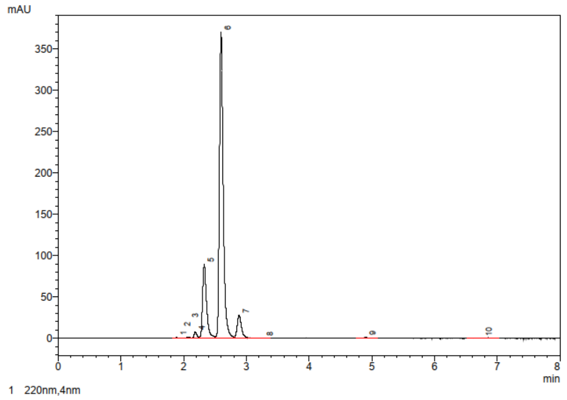

PDA Ch1 220nm

Peak #	Ret. Time	Area %	Name
1	1.89	0.12	
2	1.95	0.11	
3	2.08	0.25	
4	2.18	1.16	
5	2.33	18.61	
6	2.60	72.66	1. Enantiomer 83.9 % ee
7	2.88	6.37	2. Enantiomer
8	3.27	0.11	
9	4.90	0.28	
10	6.76	0.33	
Total		100.00	

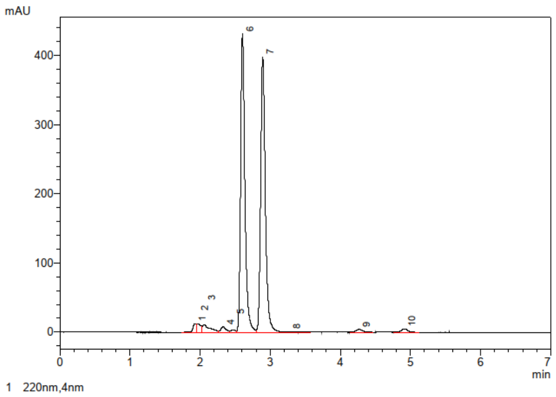

PDA Ch1 220nm

Peak #	Ret. Time	Area %	Name
1	1,93	0,98	
2	1,96	1,38	
3	2,06	2,06	
4	2,33	1,07	
5	2,47	0,41	
6	2,60	45,99	1. Enantiomer
7	2,89	46,28	2. Enantiomer
8	3,27	0,28	
9	4,27	0,75	
10	4,91	0,80	
Total		100,00	

Figure S41. HPLC traces of compound **S31**: with complex **3b** (top, left); with complex **2a** (top, right); the corresponding racemate (bottom).

2,2,2-Trichloroethyl (*S*)-1-(4-fluorophenyl)-2-methylenecyclopropane-1-carboxylate was isolated as a side product: with complex **3b**: 17 % yield. ^{1}H NMR (400 MHz, $CDCl_3$): δ = 7.43 – 7.35 (m, 2H), 7.06 – 6.95 (m, 2H), 6.33 (t, *J* = 2.7 Hz, 1H), 4.76 (d, *J* = 11.9 Hz, 1H), 4.63 (d, *J* = 11.9 Hz, 1H), 2.64 (dd, *J* = 9.4, 2.7 Hz, 1H), 1.85 (dd, *J* = 9.4, 2.8 Hz, 1H), 0.16 (s, 9H); ^{13}C NMR (101 MHz, $CDCl_3$): δ = 170.5, 162.3 (d, *J* = 246.4 Hz), 140.6, 133.2 (d, *J* = 3.2 Hz), 131.1 (d, *J* = 8.4 Hz), 119.2, 115.3 (d, *J* = 21.6 Hz), 95.1, 74.4, 30.4, 21.4, -0.9; ^{19}F NMR (282 MHz, $CDCl_3$): δ = –114.6.

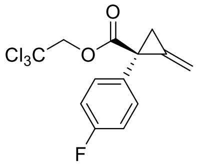

2,2,2-Trichloroethyl (*R*)-2-(chloromethyl)-1-(4-fluorophenyl)cycloprop-2-ene-1-carboxylate (S32). Prepared according to the general procedure as a colorless oil: with complex **2a**: 81% yield, 77% *ee*; with complex **3b**: 96% yield, 98% *ee*. [The *ee* was determined by HPLC analysis: Daicel 150 mm Chiralpak IA-3, Ø 4.6 mm, n-heptane/2-propanol = 98/2, v = 1.0 mL/min, λ = 220 nm, t(major) = 5.97 min, t(minor) = 7.60 min.] $[\alpha]_D^{20}$ = +39.9 (c = 1.1, $CHCl_3$); ^{1}H NMR (400 MHz, $CDCl_3$): δ = 7.36 – 7.26 (m, 2H), 7.10 (t, *J* = 1.5 Hz, 1H), 7.06 – 6.96 (m, 2H), 4.79 (d, *J* = 12.0 Hz, 1H), 4.73 (d, *J* = 11.9 Hz, 1H), 4.60 (dd, *J* = 15.2, 1.6 Hz, 1H), 4.53 (dd, *J* = 15.2, 1.5 Hz, 1H); ^{13}C NMR (101 MHz, $CDCl_3$): δ = 172.2, 162.1 (d, *J* = 245.8 Hz), 134.9 (d, *J* = 3.1 Hz), 130.2 (d, *J* = 8.1 Hz), 117.6, 115.3 (d, *J* = 21.5 Hz), 101.6, 95.2, 74.6, 35.7, 35.3; ^{19}F NMR (282 MHz, $CDCl_3$): δ = -115.2; IR (ATR): ṽ = 3144, 1733, 1604, 1509, 1274, 1219, 1186, 1159, 1037, 844, 811, 773, 711, 571, 544; HRMS (EI) for $C_{13}H_9O_2FCl_4$ $[M]^+$: calcd: 355.93352, found: 355.93348.

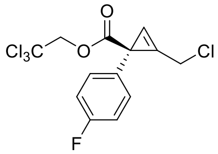

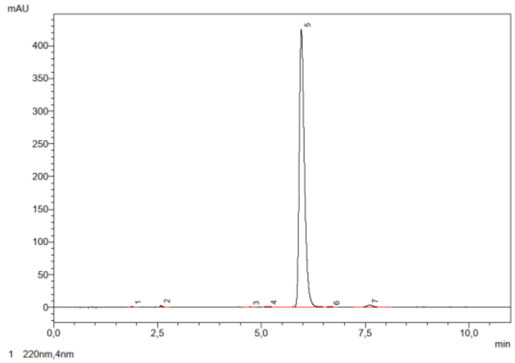

PDA Ch1 220nm

Peak #	Ret. Time	Area %	Name
1	1,88	0,13	
2	2,58	0,31	
3	4,73	0,10	
4	5,15	0,40	
5	5,97	97,73	1. Enantiomer 97.8 % ee
6	6,67	0,23	
7	7,60	1,11	2. Enantiomer
Total		100,00	

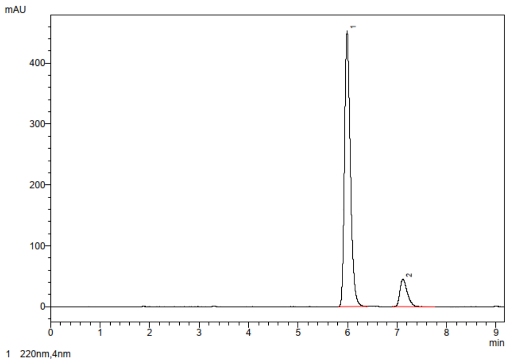

PDA Ch1 220nm

Peak #	Ret. Time	Area %	Name
1	5.99	88.72	1. Enantiomer, ee=77%
2	7.12	11.28	2. Enantiomer
Total		100.00	

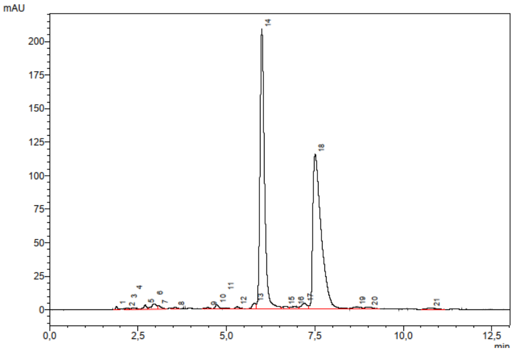

Figure S42. HPLC traces of compound **S32**: complex **2a** (top, left); with complex **3b** (top, right); the corresponding racemate (bottom).

Methyl (*R*)-2-(2-hydroxypropan-2-yl)-1-(4-methoxyphenyl)cycloprop-2-ene-1-carboxylate (S33).

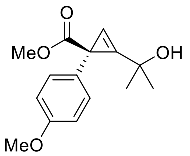

Prepared at RT according to the general procedure as a colorless oil; with complex **3b**: 72% yield, 94% *ee*. [The *ee* was determined by HPLC analysis: Daicel 150 mm Chiralpak IA-3, Ø 4.6 mm, n-heptane/2-propanol = 95/5, v = 1.0 mL/min, λ = 220 nm, t(major) = 8.86 min, t(minor) = 8.26 min; The racemic compound was prepared using a racemic mixture of **3b**]. $[\alpha]_D^{20}$ = +67.9 (c = 1.0, $CHCl_3$); ^{1}H NMR (400 MHz, $CDCl_3$): δ = 7.26 – 7.17 (m, 2H), 6.88 – 6.80 (m, 2H), 6.79 (s, 1H), 3.79 (s, 3H), 3.70 (s, 3H), 1.48 (s, 3H), 1.38 (s, 3H); ^{13}C NMR (101 MHz, $CDCl_3$): δ = 176.3, 158.4, 132.9, 129.4, 127.1, 113.7, 97.0, 69.1, 55.4, 52.5, 34.9, 29.2, 28.4; IR (ATR): ṽ = 3434, 2980, 1715, 1611, 1511, 1460, 1437, 1373, 1288, 1243, 1213, 1175, 1026, 1005, 969, 897, 854, 831, 809, 770, 589, 527; HRMS (ESI$^+$) for $C_{15}H_{18}O_4Na$ [M+Na$^+$]$^+$: calcd: 285.10973, found: 285.10963.

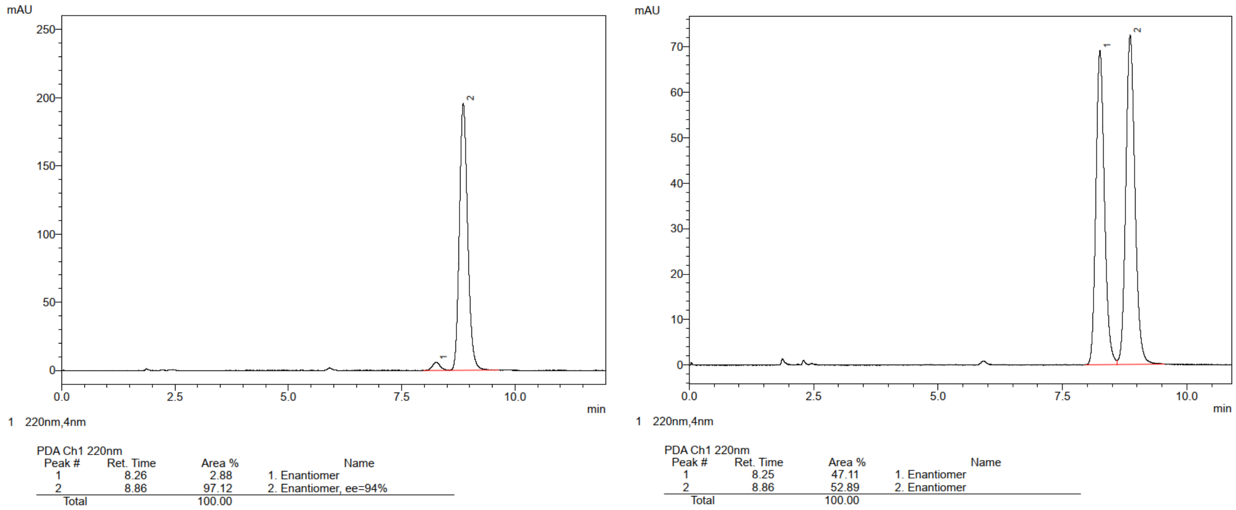

Figure S43. HPLC traces of compound **S33**: with complex **3b** (left); the corresponding racemate (right).

C–H and Si–H Insertion Reactions

General Procedure. An oven dried jacketed Schlenk flask equipped with a magnetic stir bar was charged with the [BiRh] catalyst (0.001 mmol, 1 mol%) under argon. For C-H insertion, cyclohexane (1 mL) was added and a solution of the diazo derivative (0.1 mmol) in cyclohexane was added dropwise over 30 minutes. The resulting mixture was stirred at ambient temperature until TLC analysis indicated the complete consumption of the diazo compound. For Si-H insertion, the silane substrate (0.25 mmol) and pentane (1 mL) were added to the catalyst and the resulting solution was cooled to −10°C. A solution of the diazo compound (0.1 mmol) in pentane (3 mL) was added dropwise over 60 min. The resulting mixture was stirred at −10°C until TLC analysis indicated the complete consumption of the diazo compound. For work up, the mixture was absorbed on silica, which was loaded on top of a silica column. Purification by flash chromatography (n-pentane/Et_2O or hexanes/EtOAc) afforded the desired products.

(+)-2,2,2-Trichloroethyl 2-cyclohexyl-2-(4-methoxyphenyl)acetate (S34). Prepared as a colorless oil at ambient temperature in cyclohexane as the solvent according to the general procedure; with complex **2a**: 49% yield, 85% *ee*; with complex **3b**: 73% yield, 93% *ee*. [The *ee* was determined by HPLC analysis: Daicel 150 mm Chiralcel OJ-3, Ø 4.6 mm, n-heptane/2-propanol = 98/2, v = 1.0 mL/min, λ = 230 nm, t(minor) = 2.98 min, t(major) = 3.57 min]. $[\alpha]_D^{20}$ = +12.5 (c = 1.1, $CHCl_3$); ^1H NMR (400 MHz, $CDCl_3$): δ = 7.30 – 7.24 (m, 2H), 6.88 – 6.82 (m, 2H), 4.76 (d, *J* = 12.0 Hz, 1H), 4.61 (d, *J* = 12.0 Hz, 1H), 3.79 (s, 3H), 3.32 (d, *J* = 10.7 Hz, 1H), 2.13 – 1.96 (m, 1H), 1.92 – 1.81 (m, 1H), 1.75 (dtd, *J* = 13.1, 3.6, 1.7 Hz, 1H), 1.69 – 1.55 (m, 2H), 1.43 – 1.23 (m, 2H), 1.22 – 1.03 (m, 3H), 0.76 (dtd, *J* = 14.9, 12.1, 3.3 Hz, 1H); ^{13}C NMR (101 MHz, $CDCl_3$): δ = 172.6, 159.1, 129.9, 129.1, 114.1, 95.1, 74.2, 58.0, 55.4, 40.9, 32.1, 30.4, 26.4, 26.1, 26.0; IR (ATR): ṽ = 2926, 2852, 1745, 1611, 1511, 1448, 1245, 1177, 1119, 1035, 833, 790, 753, 719, 572, 530, 433; HRMS (ESI^+) for $C_{17}H_{21}O_3Cl_3Na_1$ $[M + Na]^+$: calcd: 401.04485, found: 401.04528.

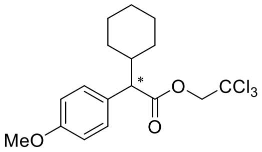

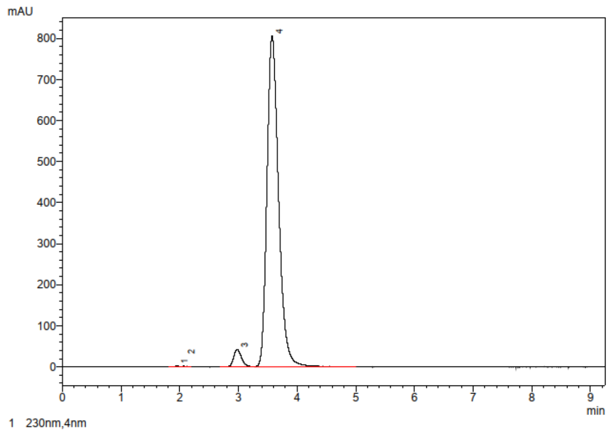

PDA Ch1 230nm

Peak #	Ret. Time	Area %	Name
1	1,95	0,10	
2	2,07	0,10	
3	2,98	3,43	1. Enantiomer
4	3,57	96,37	2. Enantiomer 93.1 % ee
Total		100,00	

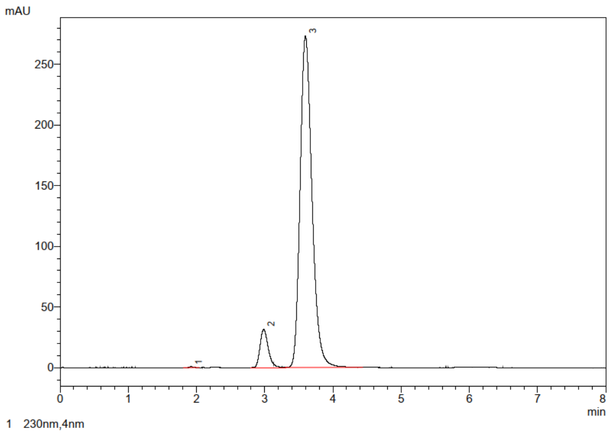

PDA Ch1 230nm

Peak #	Ret. Time	Area %	Name
1	1.92	0.10	
2	2.98	7.41	1. Enantiomer
3	3.60	92.49	2. Enantiomer 85.2 % ee
Total		100.00	

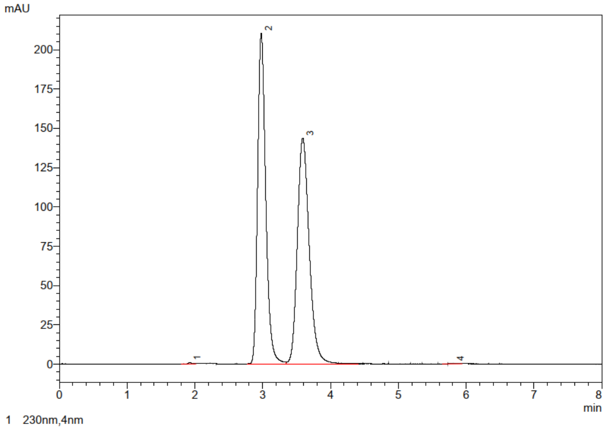

PDA Ch1 230nm

Peak #	Ret. Time	Area %	Name
1	1.93	0.12	
2	2.98	49.83	1. Enantiomer
3	3.59	49.94	2. Enantiomer
4	5.81	0.11	
Total		100.00	

Figure S44. HPLC traces of compound **S34**: with complex **3b** (top, left); with complex **2a** (top, right); the corresponding racemate (bottom).

(S)-3-phenyl-2,3-dihydrobenzofuran (S35). An oven dried jacketed Schlenk flask equipped with a magnetic stir bar was charged with complex **3b** (3.8 mg, 0.001 mmol, 1 mol%) under argon. Pentane (5 mL) was added and the resulting solution cooled to −10°C. A solution of the diazo compound (39.6 mg, 0.17 mmol) in pentane (3 mL) was added dropwise over 30 min. The resulting mixture was stirred at −10°C for 2 h. For work up, the mixture was absorbed on silica, which was loaded on top of a silica column. Purification by flash chromatography (hexanes/EtOAc = 15/1) afforded the desired product as a colorless solid (93% yield, 94% *ee*). [The *ee* was determined by HPLC analysis: Daicel 150 mm Chiralcel OJ-3, Ø 4.6 mm, n-heptane/i-propanol = 95/5, v = 1.0 mL/min, λ = 220 nm, t(minor) = 5.24 min, t(major) = 8.17 min.] mp = 65-66°C; $[\alpha]_D^{20}$ = −21.4 (c = 1.0, $CHCl_3$); ^1H NMR (400 MHz, $CDCl_3$): δ = 7.36 – 7.29 (m, 2H), 7.29 – 7.15 (m, 4H), 7.06 – 6.99 (m, 1H), 6.92 – 6.82 (m, 2H), 4.91 (dd, *J* = 9.6, 8.8 Hz, 1H), 4.68 (dd, *J* = 9.5, 7.4 Hz, 1H), 4.43 (dd, *J* = 8.8, 7.4 Hz, 1H); ^{13}C NMR (101 MHz, $CDCl_3$): δ = 160.4, 143.0, 130.7, 129.0, 128.7, 128.0, 127.2, 125.4, 121.0, 109.8, 79.3, 48.7; IR (ATR): ṽ = 2961, 1594, 1477, 1458, 1229, 1096, 1014, 977, 944, 862, 822, 754, 698, 613, 559, 508, 438; HRMS (EI) for $C_{14}H_{12}O$ $[M]^+$: calcd: 196.08827, found: 196.08820.

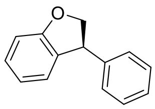

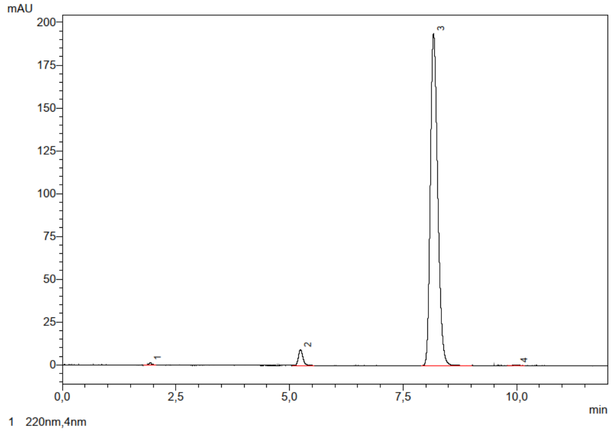

PDA Ch1 220nm

Peak #	Ret. Time	Area %	Name
1	1,93	0,31	
2	5,24	2,82	1. Enantiomer
3	8,17	96,72	2. Enantiomer 94.3 % ee
4	10,00	0,15	
Total		100,00	

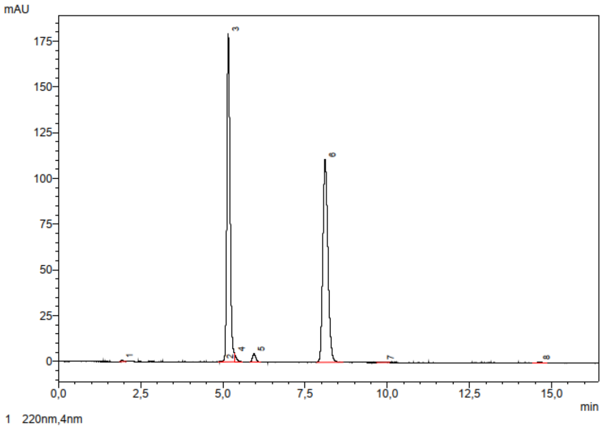

PDA Ch1 220nm

Peak #	Ret. Time	Area %	Name
1	1,94	0,15	
2	5,00	0,15	
3	5,17	48,54	1. Enantiomer
4	5,35	0,80	
5	5,95	1,36	
6	8,11	48,72	2. Enantiomer
7	9,88	0,14	
8	14,62	0,14	
Total		100,00	

Figure S45. HPLC traces of compound **S35**: with complex **3b** (left); the corresponding racemate (right).

(−)-Methyl-2-(4-methoxyphenyl)-2-(triethylsilyl)acetate (S36). Prepared according to the general procedure as a colorless oil; with complex **2a**: 25% yield, 37% *ee*; with complex **3b**: 84% yield, 94% *ee*. [The *ee* was determined by HPLC analysis: Daicel 150 mm Chiralpak IB-N-3, Ø 4.6 mm, n-heptane/2-propanol = 96/4, v = 1.0 mL/min, λ = 220 nm, t(minor) = 2.65 min, t(major) = 3.25 min.] $[\alpha]_D^{20}$ = –95.5 (c = 3.3, $CHCl_3$); ^1H NMR (400 MHz, $CDCl_3$): δ = 7.31 – 7.22 (m, 2H), 6.87 – 6.78 (m, 2H), 3.78 (s, 3H), 3.66 (s, 3H), 3.47 (s, 1H), 0.90 (t, *J* = 7.9 Hz, 9H), 0.58 (dtd, *J* = 8.3, 7.5, 3.8 Hz, 6H); ^{13}C NMR (101 MHz, $CDCl_3$): δ = 174.2, 157.8, 129.6, 128.7, 113.7, 55.4, 51.4, 41.8, 7.2, 2.9; IR (ATR): ṽ = 2951, 2877, 1720, 1610, 1509, 1462, 1434, 1338, 1279, 1244, 1197, 1179, 1143, 1037, 1007, 908, 863, 830, 797, 750, 709, 527; HRMS (ESI$^+$) for $C_{16}H_{26}O_3Si_1Na_1$ $[M+Na]^+$: calcd: 317.15434, found: 317.15412.

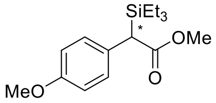

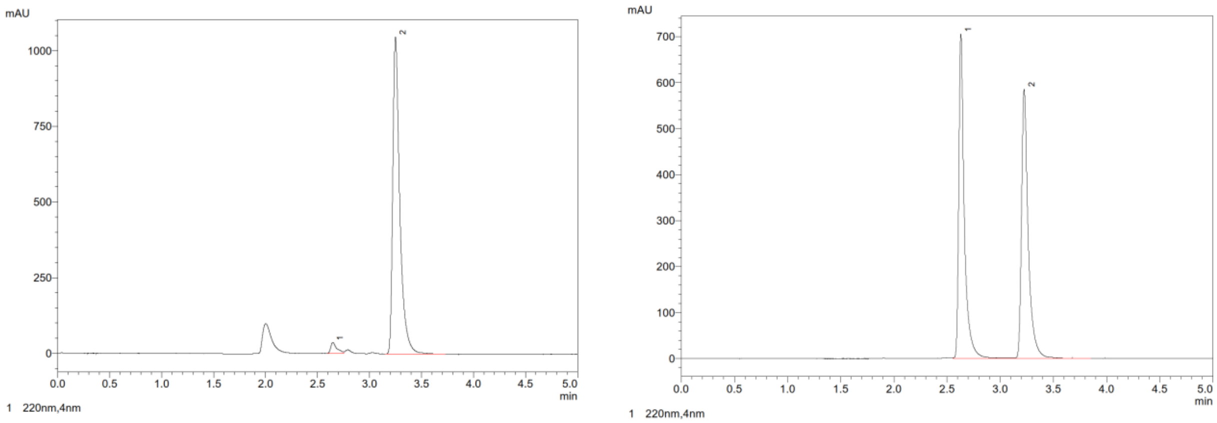

PDA Ch1 220nm

Peak #	Ret. Time	Area %
1	2.65	2.98
2	3.25	97.02
Total		100.00

PDA Ch1 220nm

Peak #	Ret. Time	Area %
1	2.63	50.14
2	3.23	49.86
Total		100.00

Figure S46. HPLC traces of compound **S36**: with complex **3b** (left); the corresponding racemate (right).

(−)-Methyl-2-(dimethyl(phenyl)silyl)-2-(4-fluorophenyl)acetate (S37). Prepared according to the general procedure as a colorless oil; with complex **3b**: 62% yield, 90% *ee*. [The *ee* was determined by HPLC analysis: Daicel 150 mm Chiralpak IB-N-3, Ø 4.6 mm, n-heptane/2-propanol = 99/1, v = 1.0 mL/min, λ = 220 nm, t(minor) = 3.02 min, t(major) = 3.42 min.]

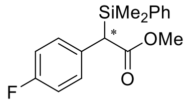

$[\alpha]_D^{20}$ = –31.5 (c = 1.9, $CHCl_3$); ^1H NMR (400 MHz, $CDCl_3$): δ = 77.46 – 7.28 (m, 5H), 7.22 – 7.11 (m, 2H), 6.96 – 6.84 (m, 2H), 3.60 (s, 1H), 3.58 (s, 3H), 0.35 (d, *J* = 5.4 Hz, 6H); ^{13}C NMR (101 MHz, $CDCl_3$): δ = 173.2, 161.3 (d, *J* = 243.9 Hz), 135.3, 134.1, 131.7 (d, *J* = 3.0 Hz), 129.9, 129.8, 127.9, 114.9 (d, *J* = 21.1 Hz), 51.5, 45.3, -4.1, -4.4; ^{19}F NMR (282 MHz, $CDCl_3$): δ = -117.77 IR (ATR): ṽ = 2952, 1718, 1604, 1505, 1430, 1330, 1298, 1274, 1222, 1197, 1146, 1116, 1089, 1014, 912, 867, 835, 814, 785, 732, 698, 661, 520; HRMS (ESI^+) for $C_{17}H_{19}F_1O_2Si_1Na_1$ $[M+Na]^+$: calcd: 325.10306, found: 325.10283.

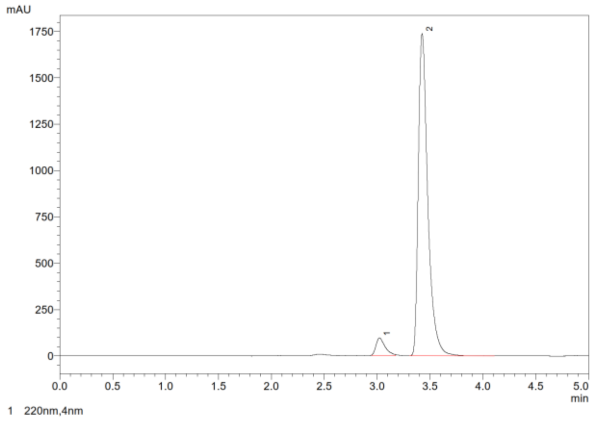

PDA Ch1 220nm

Peak #	Ret. Time	Area %
1	3.02	5.05
2	3.42	94.95
Total		100.00

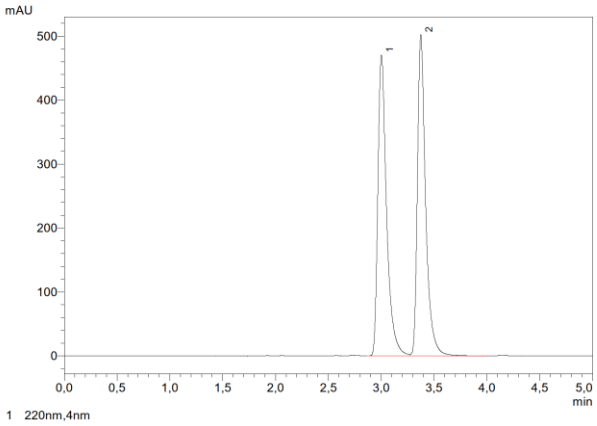

PDA Ch1 220nm

Peak #	Ret. Time	Area %	Name
1	3,00	49,80	1. Enantiomer
2	3,37	50,20	2. Enantiomer
Total		100,00	

Figure S47. HPLC traces of compound **S37**: with complex **3b** (left); the corresponding racemate (right).

(−)-Methyl-2-(dimethyl(phenyl)silyl)-2-(4-methoxyphenyl)acetate (S38). Prepared according to the general procedure as a colorless oil; with complex **3b**: 91% yield, 95% *ee*. [The *ee* was determined by HPLC analysis: Daicel 150 mm Chiralpak IC-3, Ø 4.6 mm, n-heptane/2-propanol = 95/5, v = 1.0 mL/min, λ = 220 nm, t(minor) = 4.17 min, t(major) = 4.74 min.] $[\alpha]_D^{20}$ = −24.9 (c = 1.7, $CHCl_3$); ^1H NMR (400 MHz, $CDCl_3$): δ = 7.43 – 7.28 (m, 5H), 7.17 – 7.09 (m, 2H), 6.82 – 6.74 (m, 2H), 3.78 (s, 3H), 3.55 (d, *J* = 1.2 Hz, 4H), 0.34 (d, *J* = 8.0 Hz, 6H); ^{13}C NMR (101 MHz, $CDCl_3$): δ = 173.5, 157.8, 135.8, 134.1, 129.7, 129.5, 128.1, 127.8, 113.6, 55.3, 51.4, 45.0, -3.9, -4.3; IR (ATR): ṽ = 2951, 1717, 1610, 1509, 1463, 1429, 1338, 1304, 1281, 1245, 1198, 1179, 1145, 1115, 1036, 1010, 910, 866, 834, 811, 781, 732, 699, 529; HRMS (ESI$^+$) for $C_{18}H_{22}O_3Si_1Na_1$ $[M+Na]^+$: calcd: 337.12304, found: 337.12274.

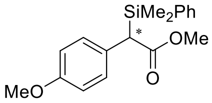

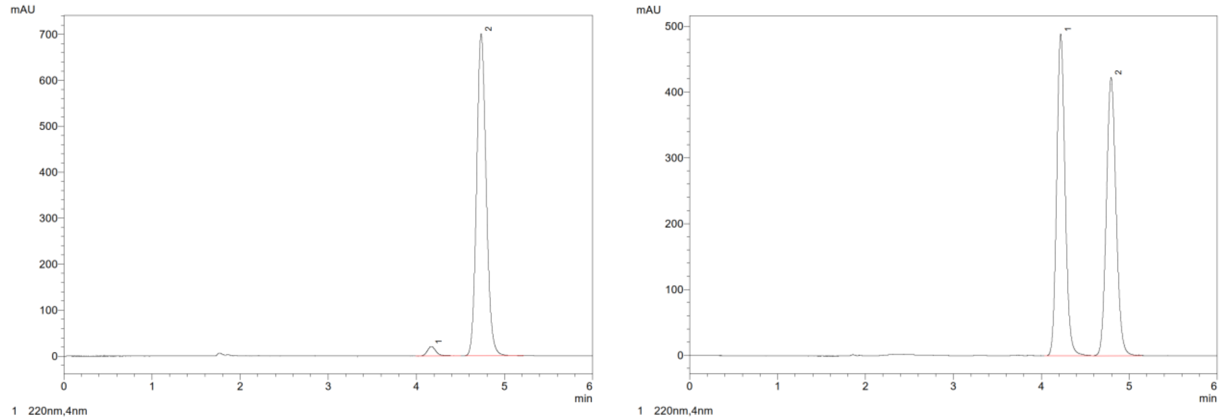

PDA Ch1 220nm

Peak #	Ret. Time	Area %
1	4.17	2.62
2	4.74	97.38
Total		100.00

PDA Ch1 220nm

Peak #	Ret. Time	Area %
1	4.22	50.04
2	4.79	49.96
Total		100.00

Figure S48. HPLC traces of compound **S38**: with complex **3b** (left); the corresponding racemate (right).

NH-Insertion and Doyle-Kirmse Reaction

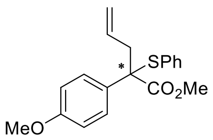

(+)-Methyl 2-(4-methoxyphenyl)-2-(phenylthio)pent-4-enoate (S39). An oven dried Schlenk flask equipped with a magnetic stir bar was charged with complex **3b** (2.9 mg, 0.001 mmol, 1 mol%) and allyl phenyl sulfide (22 µL, 0.15 mmol) under argon. Pentane (1 mL) was introduced before a solution of the diazo compound (20.6 mg, 0.1 mmol) in pentane (3 mL) was added dropwise over 30 min. The resulting mixture was stirred at rt for 2 h. For work up, the mixture was absorbed on silica, which was loaded on top of a silica column. Purification by flash chromatography (n-pentane/Et_2O = 7/1) afforded the desired product as a colorless oil. With complex **3b**: 85% yield, 45% *ee*. [The *ee* was determined by HPLC analysis: Daicel 150 mm Chiralpak IA-3, Ø 4.6 mm, n-heptane/2-propanol = 98/2, v = 1.0 mL/min, λ = 220 nm, t(minor) = 4.76 min, t(major) = 5.45 min]. $[\alpha]_D^{20}$ = +47.0 (c = 2.8, $CHCl_3$); ^1H NMR (400 MHz, $CDCl_3$): δ = 7.29 (ddd, *J* = 6.70, 4.80, 2.69 Hz, 1H), 7.23 – 7.16 (m, 6H), 6.84 – 6.80 (m, 2H), 5.90 (dddd, *J* = 16.78, 10.28, 7.34, 6.32 Hz, 1H), 5.15 – 5.01 (m, 2H), 3.80 (s, 3H), 3.69 (s, 3H), 2.84 (qdt, *J* = 14.45, 6.31, 1.35 Hz, 2H); ^{13}C NMR (101 MHz, $CDCl_3$): δ = 172.6, 158.9, 136.9, 133.4, 131.9, 131.0, 129.3, 128.8, 128.6, 118.8, 113.5, 64.2, 55.4, 52.7, 40.7; IR (ATR): ṽ = 1727, 1608, 1581, 1510, 1438, 1250, 1214, 1180, 1121, 1033, 910, 824, 729, 692, 534, 497; HRMS (ESI$^+$) for $C_{19}H_{20}O_3SNa$ $[M+Na^+]^+$: calcd: 351.10254, found: 351.10284.

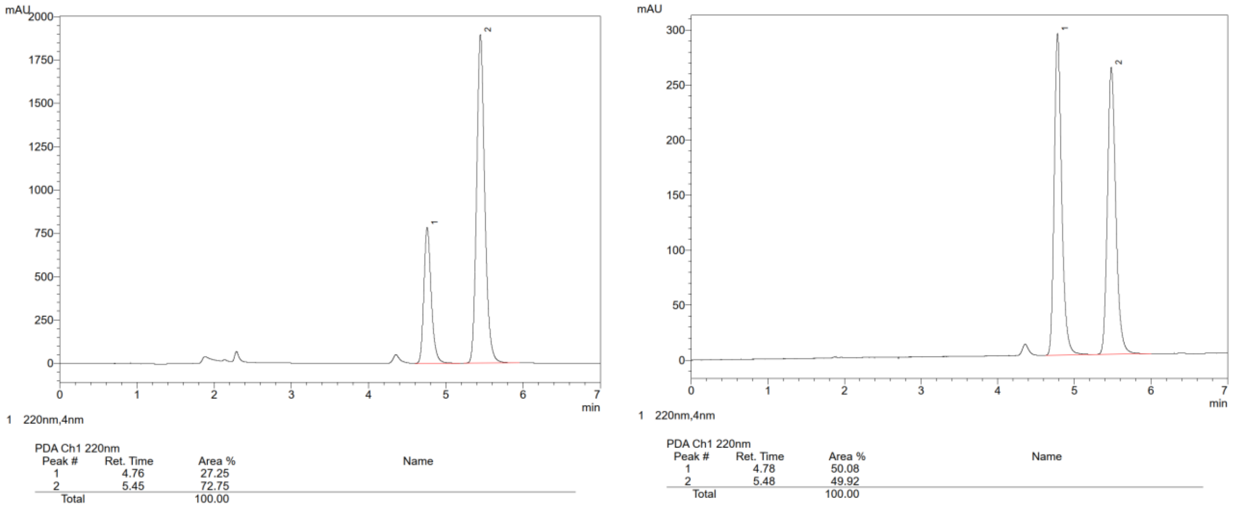

Figure S49. HPLC traces of compound **S39**: with complex **3b** (left); the corresponding racemate (right).

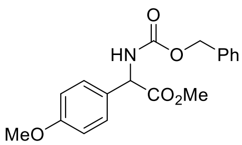

Methyl 2-(((benzyloxy)carbonyl)amino)-2-(4-methoxyphenyl)acetate (S40). An oven dried Schlenk flask equipped with a magnetic stir bar was charged with complex **3b** (2.9 mg, 0.001 mmol, 1 mol%) and benzyl carbamate (22.7 mg, 0.15 mmol) under argon. CH_2Cl_2 (1 mL) was introduced before a solution of the diazo compound (20.6 mg, 0.1 mmol) in CH_2Cl_2 (3 mL) was added dropwise over 30 min. The resulting mixture was stirred at rt for 2 h. For work up, the mixture was absorbed on silica, which was loaded on top of a silica column. Purification by flash chromatography (n-pentane/EtOAc = 3/1) afforded the desired product as a colorless solid. With complex **3b**: 80% yield, 0% *ee* [The *ee* was determined by HPLC analysis: Daicel 150 mm

Chiralpak IA-3, Ø 4.6 mm, n-heptane/2-propanol = 90/10, v = 1.0 mL/min, λ = 220 nm, t_1 = 13.07 min, t_2 = 13.98 min]; ^{1}H NMR (400 MHz, $CDCl_3$): δ = 7.35 – 7.11 (m, 7H), 6.80 (d, *J* = 8.34 Hz, 2H), 5.71 (d, *J* = 7.24 Hz, 1H), 5.23 (d, *J* = 12.44 Hz, 1H), 5.11 – 4.93 (m, 2H), 3.71 (s, 3H), 3.64 (s, 3H); ^{13}C NMR (101 MHz, $CDCl_3$): δ = 171.7, 159.9, 155.5, 136.3, 128.8, 128.7, 128.5, 128.3, 128.3, 114.5, 67.2, 57.5, 55.4, 52.9; IR (ATR): ṽ = 1712, 1611, 1510, 1438, 1323, 1305, 1233, 1212, 1175, 1049, 909, 832, 796, 728, 697, 544; HRMS (ESI$^+$) for $C_{18}H_{19}NO_5SNa$ [M+Na$^+$]$^+$: calcd: 352.11554, found: 352.11544.

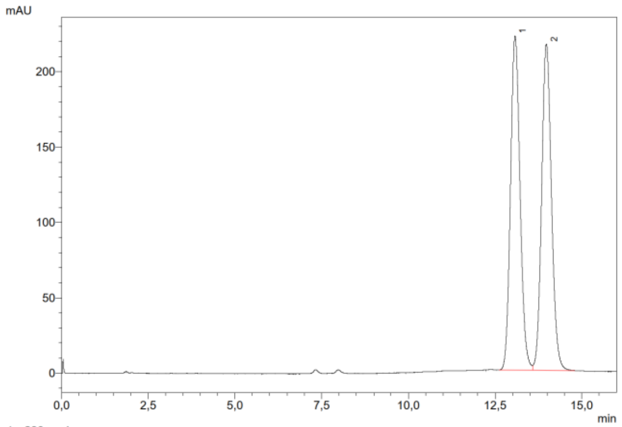

1 230nm,4nm

PDA Ch1 230nm

Peak #	Ret. Time	Area %
1	13,07	49,81
2	13,98	50,19
Total		100,00

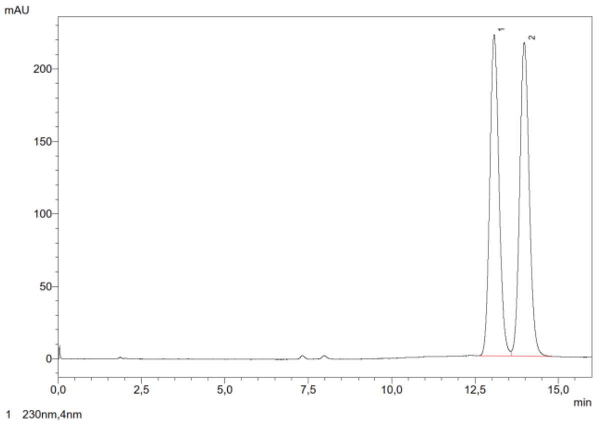

1 230nm,4nm

PDA Ch1 230nm

Peak #	Ret. Time	Area %
1	13,07	49,81
2	13,98	50,19
Total		100,00

Figure S50. HPLC traces of compound **S40**: with complex **3b** (left); the corresponding racemate (right).

Supporting Computational Information

Computational Details and Methodological Aspects

All calculations were carried out using a development version of the ORCA suite of programs based on version 4.2.[17]

For **3a** and **3b,** geometry optimizations were carried out at the PBE level of theory with and without the inclusion of the D3[18] (with Becke-Johnson damping)[19] dispersion correction. The def2-SVP basis set[20] was used for all atoms. Single point energy calculations at the PBE-D3(BJ)/def2-TZVP level were carried out to compute the relative energy between the PBE-D3/def2-SVP and the PBE/def2-SVP optimized structures, ΔE. ΔE was decomposed into "dispersive" and "non-dispersive" contributions as:

$$\Delta E = \Delta E_{disp} + \Delta E_{no-disp} \quad (1)$$

ΔE_{disp} denotes the contribution to the relative energy from the D3 dispersion correction, whilst $\Delta E_{no-disp}$ represents the contribution from the PBE exchange-correlation functional. Note that previous studies[18,21] showed that ΔE_{disp} typically provides a lower bound for the coupled cluster dispersion computed using accurate techniques like the Local Energy Decomposition (LED) when the PBE exchange correlation functional is used.[21,22]

As ΔE_{disp} is computed by summing up a series of atom-pairwise terms, it can be easily decomposed into contributions from individual functional groups. Herein, we have used this method to quantify the contribution to ΔE_{disp} originating from the interaction between the key functional groups in **3a** and **3b.** In particular, we have used the following equation:

$$\Delta E_{disp} = \Delta E_{disp(TIPS)} + \Delta E_{disp(^t\mathrm{Bu})} + \Delta E_{disp(rest)} \quad (2)$$

in which $\Delta E_{disp(TIPS)}$, $\Delta E_{disp(^t\mathrm{Bu})}$ and $\Delta E_{disp(rest)}$ denote the contribution to ΔE_{disp} from dispersion forces between the TIPS groups, the ^{t}Bu groups (if present) and the rest of the metal-ligand catalyst, respectively.

Energetic Analysis

The decomposition of ΔE and ΔE_{disp} according to eq. 1 and eq. 2. is shown in **Table S1**. For **3b**, ΔE amounts to -11.6 kcal/mol. Hence, dispersion changes significantly the structure of the system. The pure dispersion contribution to the relative energy, ΔE_{disp}, amounts to -40.4 kcal/mol. Importantly, about 32% of ΔE_{disp} originates from the interaction between the TIPS groups, whilst the interaction between the ^{t}Bu groups contribute with an additional 12%. Similar results were found for **3a**.

Table S1: Decomposition of ΔE and ΔE_{disp} according to eq. 1 and eq. 2. All energies are in kcal/mol.

	**3a**	**3b**
ΔE	-9.9	-11.6
ΔE_{disp}	-28.3	-40.4
ΔE_{nodisp}	+18.4	+28.8
$\Delta E_{disp(TIPS)}$	-12.6	-12.7
$\Delta E_{disp(^{t}\mathrm{Bu})}$	(not present)	-4.8
$\Delta E_{disp(rest)}$	-15.7	-22.9

Optimized Structures in XYZ Format

3a at the PBE-D3 level

```
366
Coordinates from ORCA-job
  C   2.44574221536528      2.21582006442828      7.28644445874505
  C   2.50852304543049      2.76544372055893      5.99384477127055
  C   1.38560894440541      3.42986446243833      5.47243786063029
  C   0.17997886913238      3.53937437784057      6.19976611201558
  C   0.15810231331351      2.96535720686877      7.49327069080912
  C   1.27285244295571      2.31127247086700      8.06538958938494
  C   3.76295080163153      2.65474953430671      5.14660668121579
  N   4.02051618908023      1.31635650395124      4.62470703485901
  C   4.61193073321718      0.27379382594779      5.37398677328880
  C   4.95572349490502     -0.78905811590941      4.38619880096504
  C   4.58736955173454     -0.33647920874622      3.10610511795966
  C   4.03153450062907      1.04423946642292      3.24027279018546
  C   4.80814343677884     -1.11447617681953      1.96979573549511
  C   5.41775299869726     -2.37180314774312      2.15382435241192
  C   5.79933645899677     -2.81528445700671      3.43509879132160
  C   5.57525992082470     -2.02468689084705      4.58025443482316
  O   4.80736983521400      0.28938619000661      6.58012118237639
  O   3.68167837950952      1.82735897642310      2.37019784048601
  Si  -1.35322700168958      4.40907263845953      5.45883357493492
  C   -1.92563974598746      5.85181792852288      6.60905039606889
  C   -1.71520556544417      5.64167578853736      8.11551973534277
  Si  1.28751755691691      1.57140861389092      9.82023673713467
  C   -0.15633607635746      2.22280048680084     10.92755221191681
  C   0.06254667415297      3.67278284647252     11.39657258801677
  C   5.04424231849364      3.15919986786493      5.83133907418643
  O   6.12254618417123      2.70362926363016      5.32760984518293
  Rh  7.94495916842067      3.04759681718500      6.13097776155848
  O   8.09288617997044      4.74809935310586      5.07153622261842
  C   7.81787130486785      5.89762992972375      5.54878131523007
  C   7.81654057251786      7.01537580819666      4.49045340595893
  N   7.53410995314103      6.41153443039730      3.19230835875059
  C   8.49700160934949      6.32395807382409      2.16964237252866
```

C	7.90301286783833	5.42129216103320	1.13577591108130
C	6.66012237327200	4.96267504858957	1.61021415507203
C	6.41265628819635	5.58524624120481	2.94509808091948
C	5.89370628090569	4.04385967447965	0.89059363550169
C	6.41021582677200	3.60720948635644	-0.34634993014304
C	7.64648125536937	4.07697851842832	-0.83062901809239
C	8.41780188632062	4.99292287151154	-0.08772777172444
O	9.59076414729550	6.86973820820106	2.18613357717471
O	5.47351706491534	5.42388794741133	3.70840305224253
O	9.81799217330039	3.36255073861948	6.83255533246791
C	10.03685047426676	3.84780677471829	7.98923722342528
O	9.18157938323445	4.04060366493608	8.90426244033820
Bi	6.90555860414079	4.25285707564427	8.16154621898206
O	7.55654673111145	6.16997304021564	6.75708875736775
C	11.48799526632272	4.28511438875016	8.25677716330858
C	11.55460439571246	5.49883680217359	9.17338645856429
C	10.69000377694196	6.59020842629674	8.97858725694528
C	10.72923782723348	7.72213633153216	9.81645971167362
C	11.66244809748090	7.71611397599892	10.87469004239554
C	12.54568628779411	6.63610172287020	11.11414358928495
C	12.47542767803535	5.53120144919171	10.23541336447285
Si	9.55250594024681	9.15257512761041	9.38055565163977
C	9.87023108671133	9.56794535542427	7.52889003818959
C	9.12929010864930	10.83674315060232	7.08442425113818
Si	13.85978363500816	6.83732215755966	12.47549163705261
C	13.17245273755591	7.87702788983123	13.95616543543834
C	11.73346136066143	7.50455524609050	14.35279827617175
N	12.17562300028180	4.44305803369864	6.97856261093704
C	13.10070024299656	3.47817420188339	6.51884314906228
C	13.34565015703862	3.80148793582323	5.08047293886179
C	12.50609046487634	4.87033903698131	4.72220335596527
C	11.72454402569972	5.27707817047646	5.92583259945035
C	14.18255747159388	3.19102063096110	4.14563039674028
C	14.15146694511194	3.68747177098983	2.82715601559130
C	13.29891431470395	4.75041731564426	2.46901994743601
C	12.45363577417994	5.36180741837864	3.41644639365108
O	13.54989675834124	2.56344732984319	7.19236894458918
O	10.85429533495940	6.12885618566185	6.00853013559590
C	9.80158492665407	10.67469863369915	10.52361445470746
C	9.45814729788557	10.39134609970217	11.99675224043312
C	7.73028093121141	8.52170788760943	9.48819370546637
C	7.53518484460067	7.40311592773769	10.52102160833033
C	11.18308354626454	11.33524716078545	10.37921344784547
C	14.52211579746463	5.18481901198031	13.23260554035409
C	13.67380351391358	4.62639011801170	14.38794379064002
C	15.33621315183762	7.79783230355415	11.67780378000937
C	16.33371245527365	6.90193981816222	10.92849012019486
C	14.83797493018382	4.04898691806820	12.24294510302726
O	7.86400593950346	1.25537492923627	7.04700238605807
C	7.32551433712622	1.09830699227715	8.19029062755261
C	7.52806590511093	-0.29951005172304	8.80443555497750
C	7.45788252346546	-0.30342003281371	10.32061668705219
C	6.67237183877630	-1.26101211812127	10.98385266384459
C	6.55033088579974	-1.26468340335244	12.39083073248823
C	7.27055390309743	-0.27834065370729	13.10786405407731

C	8.07235674560427	0.69511409712026	12.47535924030814
C	8.14676762170372	0.66493115509501	11.06903359603639
Si	5.57054511952706	-2.55242488735500	13.39065450774872
C	4.73963341199666	-1.70340287103130	14.91629173219831
C	4.10292121678875	-0.35012110607025	14.55360193578515
Si	9.04978888514232	2.04803452871048	13.38442812558953
C	10.91466824577096	1.58524983106556	13.32997135081734
C	11.53308924539011	1.71086424895867	11.93013383911599
O	6.67106863778218	1.96118670428130	8.84577990879445
O	4.93713147997114	3.96053362193372	6.80554395765439
N	8.75432420283878	-0.86917190561723	8.25428911928149
C	8.74213579756658	-1.97436872429291	7.37784761341628
C	10.12909437233114	-2.06678678272489	6.82576648066317
C	10.87273608673946	-0.98148556173323	7.32252234904769
C	9.99398423005445	-0.18842942797545	8.22816338051121
C	12.19150150125360	-0.74781693415107	6.92968657285542
C	12.75990002071721	-1.66431580031502	6.02235512556336
C	12.02330181670506	-2.76115205569232	5.53336365111071
C	10.68674445217672	-2.97544514460847	5.92599390711600
O	7.76230695509307	-2.66012870653122	7.12729874964299
O	10.24674779595699	0.84953313048590	8.81870260127313
C	8.76088810226701	3.67237047420076	12.40505097542652
C	9.47566168368928	4.88078115943582	13.02717186309801
C	8.49174537307442	2.08688770533933	15.22353015522308
C	9.47954638398437	2.81918685473066	16.14720791962082
C	7.27937680406028	3.95447599441066	12.10916652430524
C	4.15759668509829	-3.47402122905654	12.43629122046043
C	4.43742753912697	-3.82831310105398	10.96460731729887
C	6.83992291567337	-3.88130559985002	13.98745508923141
C	7.13847726737591	-4.94946824220437	12.92348730969599
C	2.76385651102601	-2.83465024816657	12.54528468791897
C	7.06026093081881	2.61390023809512	15.41161527904389
C	11.13552207308240	0.18216550850599	13.91878585887833
C	8.15420660216521	-3.26201829593646	14.49096305353828
C	5.58666081287477	-1.58447459534214	16.19338124904694
C	6.90467005442820	8.19362767030364	4.77854845088489
C	5.67037820645070	8.02349788542833	5.42663662972138
C	4.78893461129991	9.10368943745193	5.63342809691835
C	5.18831197843064	10.36431079143202	5.12646957309878
C	6.41647254855918	10.57672953335457	4.45342171622438
C	7.26842567723417	9.46177620748528	4.29804833496599
Si	3.13402166335127	8.80153909700124	6.55132896302823
C	1.68155870551137	8.83799096145791	5.27853939891913
C	0.30760560895791	9.08900937705612	5.92281773263757
Si	6.75067626056092	12.26270966414751	3.62903009833676
C	5.92108885221717	12.18536473313278	1.88418035529945
C	4.53247663845991	11.52941293277828	1.91413114846321
C	2.96935050444738	10.12299930568073	7.94973048844491
C	1.97343046520917	9.73591907503455	9.05689494291275
C	3.23987406633797	7.02516407561472	7.28673433662751
C	1.89664992892356	6.39515575132161	7.68436511628020
C	2.68226978740760	11.54954000527001	7.45205585628538
C	5.96479927130831	13.68663935872057	4.66787660869335
C	4.47787064650933	13.98705623068114	4.42082800826695
C	8.60575712147127	12.74770289868740	3.32813019787562

C	9.57075560339389	11.60319762585075	2.97059617588967
C	6.26276958467951	13.53461718525150	6.17028322394581
C	6.70541427340487	9.65077460474689	9.68628292018398
C	11.34981772553595	9.59732052159497	7.11850238284363
C	14.85717932921257	8.93388283461598	10.75834951375115
C	13.32551046523762	9.40339810117931	13.85507067860928
C	9.25344179188871	13.65216724806016	4.39046072713124
C	6.79739687545692	11.49993082128008	0.82355545384055
C	4.26315241436100	6.91977440149544	8.42723648892617
C	1.91011006528020	9.79177198590299	4.09686796222083
C	-2.68585556506502	3.04527473599705	5.17407569243091
C	-3.87816624813389	3.48870545553865	4.30941257833617
C	-0.78695004086677	5.16468161773383	3.77570961747581
C	-0.61374748987533	4.10153598620957	2.67633852883354
C	-3.16830579785965	2.37976870275359	6.47321578832121
C	2.91336642279240	2.09501779177734	10.73982093491965
C	3.43508988075808	3.47352583497912	10.29672962177811
C	1.23404123196832	-0.35520118044804	9.70501692790246
C	2.18512786880731	-0.90686542522714	8.62974131688954
C	4.05572251930238	1.06824744981061	10.75286144006209
C	-0.16580175406633	-0.96165708314674	9.52821156255488
C	-1.59914072841915	2.06366893631095	10.41192427472052
C	-1.62129835757555	6.34902355127181	3.26188438516627
C	-3.36816001770926	6.31438052027798	6.33755108790546
H	13.79723515930981	-1.51805314301030	5.68462595016105
H	5.93823464339284	14.43546242315509	6.73515397532765
H	5.72926805800340	12.66499473777778	6.60522246584968
H	7.34020847736678	13.38135537555797	6.37565594954015
H	8.65292564677974	14.55606269337121	4.61257659356898
H	9.41753109494556	13.11360387000817	5.34529835162762
H	10.25101055418114	13.99925104122883	4.04301240719680
H	10.53048869520024	12.00950781722886	2.58404926735948
H	9.82419946519978	10.99939266650178	3.86635590402727
H	9.17203688114755	10.91209989212476	2.20309228619530
H	5.26973615476383	7.27283400400585	8.12490514946563
H	3.96006174497870	7.51315710241847	9.31476657714819
H	4.34920322784855	5.86614422393046	8.77280382607323
H	8.87181896694529	7.36231257621809	4.42150015095640
H	9.39297046543864	5.35599349969411	-0.44460697353591
H	4.94214746445347	3.65671193744282	1.28620080142246
H	5.83885793938400	2.87990733096037	-0.94262218626254
H	8.23336511337207	9.56339662128406	3.78182422959146
H	4.50393329696704	11.21684119899420	5.24094942380356
H	5.39726433527004	7.01580688298489	5.75995833654670
H	5.89979526757282	-2.35296975859973	5.57936908671077
H	6.28894693453397	-3.79493789430972	3.54472418516698
H	4.52221428849053	-0.74881317385039	0.97263450303386
H	11.76168434947385	6.17148165586636	3.13786229107430
H	13.28693359375434	5.10563583494729	1.42734847810908
H	14.83097390277525	2.35053042086108	4.43396875603056
H	10.09647295727725	-3.81803182688051	5.53672394975348
H	12.74713506673639	0.12917043541856	7.29663184713001
H	1.95752923113465	10.85144315568390	4.42712451600402
H	1.07796688091973	9.71976364397645	3.36270012623574
H	2.65745737730997	12.26524257013397	8.30289608225065

H	1.69862016827803	11.62019911057511	6.94339715037422
H	3.44828847694527	11.92084606393379	6.74512652063665
H	4.04345048666997	11.58869497967765	0.91750816571158
H	3.84612298826581	11.99271000290644	2.64843434704248
H	4.61209771426225	10.45490655208433	2.17933810400491
H	2.04027875951460	5.33863373310591	7.99479349856094
H	1.17899979602977	6.37847695676745	6.84166913161356
H	1.41131060390014	6.92660032200242	8.52828033513631
H	2.21016770473004	8.75766079920031	9.51852440177395
H	0.93292923107941	9.67915518708690	8.67773795981070
H	1.98047725174393	10.49384093496491	9.87066733300562
H	7.77129984335098	12.00710697457643	0.67502011474605
H	6.28267541910533	11.48239896577869	-0.16198577435402
H	7.00529828518333	10.44256336298828	1.09537949512772
H	2.85326451968448	9.57412025538569	3.56039182945972
H	-4.53158747266595	2.62165073451023	4.06887555813901
H	-3.89744082334627	1.56853062819638	6.25631847792229
H	-2.33674774604080	1.92709553884450	7.04832649018682
H	-0.10282127521150	-2.06896097567972	9.44455656185269
H	2.22509342015834	-2.01748698375264	8.67671768221017
H	-0.65653136228224	-0.59620215916349	8.60060476204954
H	-2.66003401837352	6.05007377324337	3.00968735732738
H	-4.51118880743029	4.23440668812126	4.82993373779184
H	1.83592805040936	-0.63419204767370	7.61217061195601
H	-3.68251869741253	3.10411339740056	7.13997041938754
H	-3.56257651582184	3.93855678118678	3.34718091724634
H	-0.83803181423520	-0.73664812105166	10.37911874284086
H	-1.17342070644346	6.76911072987330	2.33483763886989
H	-1.68034472678269	7.17477395033670	3.99863244866261
H	-1.79925803449111	1.08419156550753	9.94006836072185
H	-0.75917323247545	3.04259100782387	8.08970837679845
H	3.73199099409981	0.06699777508811	11.09856153426828
H	-3.57159535203207	6.50277485871119	5.26550785263095
H	-1.84929958205790	2.84521784063538	9.66626346607070
H	-4.10340838985601	5.56053808558234	6.68825677314019
H	-2.32530672511110	2.18916461990503	11.24410341043516
H	-3.58692380515042	7.25608190352985	6.88690059858765
H	-0.66541474318310	5.39960972703530	8.36629348928854
H	-2.35328864727682	4.82361163336431	8.51136270247456
H	0.04700342398380	4.37960699200635	10.53987400716385
H	-1.98972475306081	6.56066798408602	8.67815193618143
H	-0.74523086178905	3.98873583800693	12.09220288376976
H	1.02453046654250	3.81253327406229	11.92727294767216
H	2.65891489329011	4.26365498794259	10.33182002788272
H	3.22259091807554	-0.53444338598299	8.72446480406746
H	1.47409487022411	3.87347559875611	4.46873826677085
H	3.33724840134529	1.70834222108977	7.68015331851891
H	4.52190456081337	0.95019170527406	9.75517349417752
H	4.87233197170256	1.39187096048196	11.43345704164341
H	3.80324239515516	3.44086940375453	9.25081683047813
H	4.27943797790068	3.80242849320963	10.94226900468654
H	-1.59337967604252	3.68881004732431	2.35773218302074
H	0.01025714948618	3.24354649365482	2.99794161103440
H	-0.13603961347513	4.53700200973428	1.77195723864760
H	-0.05015624780665	1.56231806900780	11.82270687585373

H	1.61558803205350	-0.68250981589859	10.70022670982172
H	2.55720761719952	2.19284651026201	11.79316298400954
H	4.25906495586823	14.18726704570345	3.35315267741269
H	4.15953763652067	14.88281897305766	4.99747524107805
H	3.81809036324999	13.15436515350379	4.74180487947417
H	3.64501400708815	3.29194857007333	4.24250727703157
H	6.52933909554430	14.57689769539181	4.30327886869873
H	8.48623842706197	13.37360054912176	2.41055034233780
H	5.80138892198324	13.25572319353452	1.59052178420656
H	3.98750227855767	10.12730709198721	8.40300645779714
H	1.68109509534825	7.79812494833851	4.87232258698105
H	0.22148475225410	10.12866333152680	6.30047339695963
H	0.10073284026399	8.41204230718337	6.77471173443497
H	-0.50961404841225	8.94904544377041	5.18139224264202
H	3.62410659752494	6.41122800823299	6.43986245179736
H	-2.11990230060086	2.27769021787649	4.59610334082111
H	-1.24587702013032	6.67856870898906	6.29872963729924
H	0.22301942138521	5.57273767218351	4.01988711969463
H	6.77671290535550	10.43362246578477	8.90485898330395
H	5.66939846806924	9.25171688918481	9.65284201680480
H	6.82466119175429	10.15174974404366	10.66921638967558
H	8.22416304118643	6.55311459507722	10.34329999072342
H	7.72354740802929	7.74842509647373	11.55962220292735
H	6.49233746680720	7.01483015450329	10.49909843673213
H	11.45028061162659	9.73681747187740	6.01948479300611
H	13.02491902109757	9.88699337446303	14.81004394890193
H	9.24970422922700	11.00254496915790	5.99384221914144
H	11.86821213554310	8.65416329212138	7.38143503870538
H	12.67878979997656	9.83948613904827	13.06554251921320
H	11.90050693073440	10.42910544593593	7.60514102453236
H	9.51645352729871	11.74311228331710	7.59788450077265
H	8.03875920284110	10.78282451930647	7.27105079682890
H	11.43408434580059	8.02462890817243	15.28883613504708
H	11.71415554044921	8.59269709455699	11.53550859924236
H	11.00549850724107	7.79718471494914	13.56919360363511
H	11.60087536450068	6.41892319857624	14.51803598937308
H	10.15251339638732	9.65968289256368	12.45703143810293
H	8.43551694318700	9.98263084725614	12.11985812137424
H	14.15428929871895	3.72391778326102	14.82484510931148
H	9.51967349142906	11.31956872722802	12.60595632005062
H	14.16165856347665	9.63361088501523	11.26124151847938
H	15.71694867147716	9.53233132896282	10.38587472298212
H	14.36693089360198	9.71515083582639	13.64134081351442
H	14.32543051005017	8.53023487127636	9.87190069879196
H	17.15639852627161	7.50864375550735	10.49049849145804
H	9.97928581184567	6.55762925968970	8.14065346608776
H	15.84406560570062	6.36957682137192	10.08504663385973
H	13.14510246801249	4.67004826301629	10.37085237418029
H	16.79996812817544	6.13997713569826	11.58396906337567
H	15.38846324812699	4.38301388270701	11.34323590010908
H	13.90722173706206	3.55126280051452	11.90457475855039
H	12.66803204236763	4.31334358651534	14.03617766802698
H	15.45124073931323	3.26311478646546	12.73473261020617
H	13.52998891127036	5.35230393471781	15.21188448747782
H	11.99262708318667	3.42086250272166	8.74263776841917

H	7.55631680439740	8.07950313390797	8.48043511748892
H	9.39897821769684	8.70497947293143	7.00294537488831
H	13.84358072345393	7.54729504652968	14.78496073089898
H	15.87118109021756	8.25164892590078	12.54553723093631
H	15.49117925054284	5.52673470218658	13.67153384802003
H	8.83129897264700	-4.04591777048497	14.89472927039501
H	8.00150245532102	-2.51290170037953	15.29174733659913
H	6.00934145273282	-2.55727857701968	16.51472164385109
H	6.33559354612362	-4.38396083680914	14.84710648665617
H	4.97096195731247	-1.19678409883531	17.03427661850183
H	6.43310527272960	-0.87632400932964	16.07059685885697
H	6.70811937986037	2.46068913694036	16.45497225571241
H	6.33215378427523	2.11744918927799	14.73833291813284
H	8.49754998046692	1.01106201295072	15.52094843323231
H	3.91188775936087	-2.41317857368245	15.15280325254844
H	9.14471597594004	2.77189052353543	17.20651018759908
H	7.00551085010478	3.70342693035175	15.20837136544363
H	7.19882222620261	-0.26514655587314	14.20620347433922
H	3.50103598309804	0.04734270224390	15.39956091358810
H	4.87674613437942	0.40761429644694	14.31564273304753
H	10.76566403123047	0.09752324807382	14.96185951605182
H	10.49908266474266	2.38578211156876	16.10490208002242
H	11.42139407618997	2.32296173599830	13.99411481875019
H	4.11934879962438	-4.43297857744660	13.00828002521050
H	9.56820708286133	3.89345728967222	15.88278465636325
H	3.43774364378148	-0.41270535652301	13.67088907898779
H	2.44300672686408	-2.66542214505690	13.59165474192275
H	10.60601562711723	-0.58971021740859	13.32056493187922
H	6.70365137360982	4.18271224041308	13.02891839349250
H	2.72430152490334	-1.85767191117320	12.01996586837476
H	6.78639515274231	3.09197104954216	11.61632756798728
H	11.45373493675220	2.74141180074689	11.52719996638499
H	9.00405184694596	5.19354970789375	13.98267338140106
H	1.99818432208238	-3.48334631321468	12.06652301406871
H	10.54266191640395	4.66560529132782	13.23973158723977
H	11.04765068276216	1.03771553377565	11.19490758765109
H	7.17110107321417	4.83741195500273	11.44362904045322
H	9.46016184793436	5.75923449104642	12.34797449703364
H	8.69466126024773	-2.75151295747221	13.66677920628227
H	7.87163886804594	-5.69347948987205	13.30514595119999
H	7.58577241437537	-4.49608811659420	12.01273252350223
H	6.23404883592557	-5.50936587938329	12.61261431736049
H	6.13696067433728	-2.00345628456355	10.37641206234830
H	5.45114377442474	-4.23840525249769	10.79187066997842
H	4.31693603425445	-2.93734900371622	10.31447743489136
H	3.70715405114670	-4.58248368095456	10.59935651402647
H	12.21413803208376	-0.08773180114076	13.92698237457438
H	12.61261755903283	1.44482950620699	11.94925662751242
H	8.74389228379010	1.41317833693803	10.53048882701443
H	6.71176060295804	-0.93369760591122	8.39357240097842
H	11.27449386543491	12.21954170107606	11.04748241208723
H	12.00911727469880	10.64194967250498	10.64131043705957
H	11.37148015922186	11.68265460365826	9.34389350393063
H	9.04594649887762	11.40185582654653	10.14495251972231
H	9.25104393817367	3.47655592102211	11.42423401343263

```
  H   8.01910869917974      3.71542676553539     -1.80117198622303
  H   14.79517278912616      3.23101778288805      2.05973739868470
  H   5.60973099803337     -3.01478512774185      1.28126269227268
  H   12.49933937078538     -3.45704176960503      4.82567693316715
```

3a at the PBE level

```
366
Coordinates from ORCA-job
  C   2.08481151505900      2.51897667711003      7.40323048815041
  C   2.20778411416103      2.88957609396174      6.05273529297112
  C   1.05052357681639      3.23116204076428      5.32599402310387
  C   -0.23930337324910      3.18419786279076      5.90156157614291
  C   -0.31245564546650      2.81673159540096      7.26968791943662
  C   0.82549820061688      2.49363622706534      8.04724210795431
  C   3.53333389746178      2.83303393726307      5.30530165543625
  N   3.81724608698377      1.49576617542712      4.78542801554685
  C   4.35975634831465      0.44463302806626      5.55620677874933
  C   4.63888921606541     -0.66133847961260      4.58960085093572
  C   4.25219148746342     -0.23072791143947      3.30721619402198
  C   3.75472414077967      1.17658278870565      3.41370920556209
  C   4.39519792494817     -1.05329542341631      2.18898030288612
  C   4.94608508522428     -2.33457255325822      2.39208872824825
  C   5.34772788756767     -2.75803665071919      3.67413989438089
  C   5.20101805268813     -1.92249795940308      4.80032259640125
  O   4.55654740310881      0.48469036364287      6.76184500408952
  O   3.38248224407233      1.93507165143979      2.53053726280509
  Si  -1.81303260932157      3.51980817360142      4.84469475102669
  C   -2.81044776746511      5.01586271521944      5.57590766139543
  C   -2.76806669116679      5.16783418149643      7.10559649576326
  Si  0.75705400505161      1.93476945785284      9.87846514753037
  C   -0.79177829716273      2.55682135743576     10.87210587873516
  C   -0.67277211388019      3.98829347475749     11.42691256111748
  C   4.79081046326533      3.35244037826634      6.03384750729989
  O   5.88193638132718      2.93896540983912      5.51568835886058
  Rh  7.71872079036353      3.37045138329261      6.24903984613091
  O   7.76372790600092      5.04810169469004      5.11945506414244
  C   7.42739546888466      6.19849346487645      5.55644991355975
  C   7.42508308640441      7.30404706074101      4.47944818152439
  N   7.17889412180428      6.69667915340550      3.17360189717807
  C   8.11132116131694      6.73679666879016      2.11599735370221
  C   7.52694675621880      5.88126668494005      1.03498489359806
  C   6.32007935502894      5.33492770866844      1.50818928337564
  C   6.08188183738884      5.85451093506068      2.88921753846288
  C   5.56527622880309      4.44578476620628      0.73999842634372
  C   6.05295936990555      4.13157347955632     -0.54469729210605
  C   7.25266322171303      4.69013813347026     -1.02724719107095
  C   8.01338940587607      5.57508861162603     -0.23701313484735
  O   9.17085621137596      7.34519382165848      2.13523414303214
  O   5.15716956158588      5.62040858008127      3.65240315000035
  O   9.61813382278938      3.71492407856501      6.86204133729607
```

C	9.90372354857914	4.29024757831026	7.96437476148379
O	9.08702126678131	4.66897400027469	8.85509258768954
Bi	6.72779430373832	4.62198097894710	8.25535514469041
O	7.12683731327222	6.49710299367318	6.74972061136725
C	11.41759370364090	4.48770001689221	8.19748951004604
C	11.79832321312687	5.57439315442344	9.19414918966345
C	11.18264885990879	6.83727523546981	9.16656298878962
C	11.56785725843645	7.86583737130675	10.05400848038018
C	12.60733432345010	7.58496838987061	10.96906322993786
C	13.26800204673182	6.33098504121124	11.02315948609482
C	12.83515179023783	5.33476048659315	10.11739114340666
Si	10.69833160356868	9.55136678983900	9.83170396443523
C	11.25883396521668	10.17873604642948	8.08653480258025
C	10.79895398036796	11.60872410937746	7.75527505690554
Si	14.78732841389851	6.13343286146398	12.17133093609607
C	14.52500507799198	7.07651082407692	13.84952707537725
C	13.10702614407243	6.90297589978354	14.42486299636499
N	12.07718174124521	4.64625890554830	6.90290324140032
C	13.06091583271184	3.75206263746721	6.43019032840313
C	13.34996329396114	4.18093627283770	5.02548172082443
C	12.49186105553731	5.24826466282420	4.70493575005113
C	11.66196575164271	5.55971319106907	5.90794653256079
C	14.24855959562974	3.66551445769079	4.09017065345501
C	14.25988836523712	4.25249641409927	2.80894022701584
C	13.38579463414090	5.30839530973457	2.48435534831914
C	12.48169404281950	5.82594551618654	3.43376332223506
O	13.52944361212293	2.82073762857914	7.06711346979027
O	10.78855641238892	6.40313134032926	6.04209764732819
C	11.09971313005328	10.82644069273117	11.22205448639551
C	10.68455371470057	10.34427178781400	12.62469015713584
C	8.78501179748358	9.22660012773487	9.76115459786357
C	8.29437267270252	8.10033100280427	10.69032607106802
C	12.54133881414431	11.36664205094811	11.21911897218269
C	15.24972446911440	4.30805590474721	12.65721795533855
C	14.55596688633956	3.76470222741122	13.92027666868277
C	16.31745619334430	6.90012442783444	11.25327330853845
C	16.99832191071327	5.95059936847457	10.25263772990842
C	15.17933819879728	3.24290442007521	11.54526211601540
O	7.73638852233857	1.60338274209096	7.23788572695348
C	7.26313596886018	1.46506590594455	8.41421657192647
C	7.43876872572985	0.05098203601031	9.00484214858037
C	7.34942901999171	-0.04222356563719	10.52117919347061
C	6.64567114473762	-1.11561399942746	11.09717336119587
C	6.55547292193896	-1.27923576345033	12.49945394973163
C	7.20731156203537	-0.31232833730681	13.30317282704836
C	7.91672830514114	0.78680237205289	12.76350691826106
C	7.98202229605820	0.89491805386370	11.35789158371584
Si	5.74132135526107	-2.81985097701521	13.28699271230569
C	5.22045512597776	-2.51343628419554	15.13232304908687
C	4.52681154813738	-1.16080563858908	15.38677655833074
Si	8.82034984415905	2.08195081199744	13.84119783870934
C	10.66774156894709	1.53511851854834	13.97710953413717
C	11.39177467841080	1.56509663382908	12.62053251617235
O	6.66049415804191	2.34513117656404	9.09586253045015
O	4.67117945166965	4.14219913562460	7.01608265651578

N	8.65622557600565	-0.54465073671984	8.45944696309277
C	8.65220477873160	-1.72678010827202	7.68960029735913
C	10.05877908469083	-1.89397134918122	7.20512241371190
C	10.81171191002927	-0.79089477649107	7.64720316239669
C	9.91831481190579	0.08876008607661	8.46029686451808
C	12.15478237349468	-0.63095212540678	7.29925339959724
C	12.73592914608423	-1.63669422057716	6.50060672674102
C	11.98920315435925	-2.75142237200895	6.07163229742253
C	10.62956933766870	-2.89295885279883	6.41490103140464
O	7.67056647717505	-2.42170563710190	7.47383295074447
O	10.17606009407908	1.14634578074856	9.01536293451460
C	8.73047362171284	3.78212220178280	12.93075385632153
C	9.44647372566383	4.91322650513601	13.69120222756810
C	8.03731848478934	2.05739804072862	15.60492659667090
C	8.88720559723522	2.75950077141065	16.67899144413527
C	7.31430445618477	4.20865123556480	12.50280407906101
C	4.13952319072803	-3.45094879495109	12.38119400003597
C	4.17509534331622	-3.48102555306224	10.84119427212353
C	7.06313215333185	-4.24192099742963	13.25557003289297
C	7.11093108788267	-5.05539140413452	11.95119958134627
C	2.82678248811329	-2.78967692760615	12.83951995993232
C	6.57998587655996	2.55085754990218	15.63944924782063
C	10.79639731737912	0.15336053815436	14.64254795157915
C	8.47239381221918	-3.73121848838906	13.60668854922983
C	6.29453769484384	-2.77351839734025	16.20334463370934
C	6.51150010081493	8.49459576314290	4.74471075737253
C	5.25118838304645	8.34831347830386	5.35018321069935
C	4.38291686156342	9.44894714358782	5.51543943086380
C	4.82075568013370	10.70526115307685	5.03929165527491
C	6.08250593716569	10.90167386344892	4.42521058504578
C	6.91411259777208	9.76561922001002	4.29079180179115
Si	2.73759179776631	9.16872306936560	6.45197954736957
C	1.91921251351462	7.55374530210688	5.77828922019369
C	0.46403437455505	7.35703944488643	6.24378007268129
Si	6.52147690164045	12.58867400386634	3.63707510976397
C	6.11658403825394	12.44100638554710	1.74398763582535
C	4.81270618977782	11.66456641388580	1.48609879978282
C	1.65800187220120	10.75615932546670	6.25974199055384
C	0.48756445226261	10.86849786997495	7.25356903410085
C	3.30860911992287	8.89904324466575	8.28239315255273
C	2.31320101168116	8.20633768170256	9.22787580268117
C	1.17621734298260	10.97994626401172	4.81423022172775
C	5.46515748056474	14.04051203590458	4.37610846326142
C	4.07593864968587	14.26827925760472	3.75307331557777
C	8.38367420949598	13.12294252207365	3.81073258701345
C	9.42810951925563	12.00101039091890	3.66491576150702
C	5.37163535527964	14.04903339044031	5.91487857183047
C	7.94574657706814	10.50119405371121	9.96875125650163
C	12.76406115497592	10.00466305526380	7.81476268558388
C	15.99956103840561	8.24000353482847	10.56577241014429
C	14.95243049297004	8.55366454296907	13.90610405087075
C	8.71609712942252	13.96647209596707	5.05466440366883
C	7.25281766294238	11.85918567014673	0.88589879547748
C	3.86006545825560	10.19925502011895	8.89284829002838
C	2.01726807337593	7.38845547893457	4.25054824634977

C	-2.80303372286142	1.85549760364868	4.76098318367849
C	-3.92706091275809	1.83285681236090	3.70777657186824
C	-1.19193404033227	4.00927462974583	3.07432090801057
C	-0.65962782835562	2.81280889287057	2.26222581098918
C	-3.32776054633493	1.35149690639537	6.11693748855920
C	2.30613327988495	2.60128269493127	10.84283484196852
C	2.70452678504621	4.03693520735098	10.44774792295235
C	0.78544889097130	-0.00557878064263	9.93728979800488
C	1.73178818048475	-0.61286296698552	8.88668743188550
C	3.54532734054691	1.68900742367739	10.87808443478709
C	-0.58926042676536	-0.69079084774991	9.86453821481379
C	-2.17411157936916	2.38290989487002	10.21383505842563
C	-2.16894732477651	4.84530059789979	2.22762357810151
C	-4.26810634119516	5.09105412895298	5.08219715479522
H	13.79179279057562	-1.54768487009461	6.20263052320947
H	4.95868105073400	15.01541474903172	6.27874979070922
H	4.69763885623894	13.25187969083387	6.28950195650044
H	6.34995436556696	13.90021956112629	6.41111915252547
H	8.08069543433871	14.86886899300709	5.15086625322901
H	8.61172312925141	13.37762112619360	5.99034804835616
H	9.77171575127119	14.31465701966766	5.01454434830471
H	10.45190443329831	12.43011586587481	3.59610650142488
H	9.42320164809133	11.33272040934234	4.55071261143831
H	9.27797889267061	11.37053397993589	2.76685413412419
H	4.62546555192424	10.67409389435672	8.24445905203112
H	3.05698987522727	10.94764050880210	9.06297427999237
H	4.33397928406280	10.00911779705875	9.88103636897128
H	8.47824710945826	7.66070423996185	4.42468856103951
H	8.95773433361576	6.00843189508616	-0.59877700502668
H	4.64116033944482	3.99581267327713	1.13476210931360
H	5.48782097328614	3.43478288269772	-1.18232566156156
H	7.89953395879206	9.85064483087794	3.80851467367713
H	4.14801829530830	11.56967837258738	5.14553936726300
H	4.94402303991428	7.34853008067941	5.69022814711492
H	5.53799721558998	-2.23794498414930	5.79976086559265
H	5.78793398845738	-3.75934215969252	3.79827903126134
H	4.08778037861232	-0.70536604678274	1.19148155761887
H	11.77732996889680	6.63380752954212	3.18257108478898
H	13.40791104368627	5.73443951442564	1.46963327223883
H	14.91470457503123	2.82934303834999	4.34970268430120
H	10.03248519264993	-3.75142961753352	6.07289251804755
H	12.72327724033550	0.25686969582699	7.61684412298868
H	1.46059958838744	8.18441303739971	3.71329950349627
H	1.57699467463942	6.41571581919842	3.93711593944945
H	0.69769465250703	11.97791922515815	4.70178225380296
H	0.41726532005657	10.22498796244692	4.52006398551525
H	2.00029183839553	10.91951862105910	4.07384674567067
H	4.53995219384854	11.69259600750620	0.40824254427391
H	3.94969199464242	12.06291840505114	2.05553736168033
H	4.92342496153783	10.59738608308323	1.77061002649987
H	2.76496644949974	8.05823196073173	10.23397274069604
H	2.00905591290428	7.20576012303785	8.85933172721031
H	1.38934237221323	8.80364118414863	9.37781213310399
H	0.82073827122018	10.80866784419368	8.30910873947258
H	-0.26573995772775	10.06901096535433	7.09960754761784

H	-0.04188925325137	11.83925737430094	7.13168152055027
H	8.18959264915877	12.44771092328810	0.95528317740426
H	6.96059357717794	11.83510757005645	-0.18720853451640
H	7.48985724704924	10.81231870934636	1.17363710256106
H	3.06261335470847	7.40289366519903	3.88720958278945
H	-4.34982167771130	0.80836550134910	3.61191198321056
H	-3.83372265345882	0.36749803243251	6.00174385077851
H	-2.51806904867924	1.21497722325093	6.86057456428997
H	-0.47793902386250	-1.79516593263346	9.93976481852068
H	1.82380229507534	-1.71222416895177	9.02899261125619
H	-1.10038630329538	-0.48781670747591	8.89879499656227
H	-3.08679894288619	4.27998253213752	1.96199260129297
H	-4.76734894711396	2.50169373306889	3.98290457564490
H	1.34499988282419	-0.44553674017560	7.85962126003907
H	-4.07518731162038	2.04563667801157	6.55681978534509
H	-3.57882115168912	2.13765083539475	2.70076508927680
H	-1.27127967576018	-0.37680063655099	10.67962523145358
H	-1.69196646295213	5.14725656004707	1.26933766451690
H	-2.48644476962602	5.77613755685219	2.73880755330116
H	-2.31191535784331	1.40552570608562	9.71339103223207
H	-1.29795100616285	2.78025532369956	7.75270203986323
H	3.31133059156374	0.65821412458728	11.21245385126130
H	-4.36109790878090	5.02351677340914	3.98062210524996
H	-2.35720897316560	3.17447221596672	9.45892059806476
H	-4.88419880378178	4.27686309788154	5.51811837275066
H	-2.98196175008715	2.48369614959089	10.97142582258303
H	-4.73847203364568	6.04941375716689	5.39452758724153
H	-1.73457902306412	5.21270234796400	7.50124461461017
H	-3.28994982704265	4.33112851125761	7.61694438673438
H	-0.64198769700634	4.74122938335755	10.61073851439554
H	-3.28466908637107	6.10212379652744	7.41769457946386
H	-1.55427104937543	4.23626582915239	12.05842599639765
H	0.22830277835333	4.13626039975407	12.05404971592896
H	1.85158215777095	4.74411812048612	10.46717553906193
H	2.75491165702739	-0.19010739296592	8.91564627659224
H	1.17956532550830	3.52253493738820	4.27177598095372
H	2.99519069950360	2.23799404761029	7.95066519463437
H	4.04885233821283	1.61516211163137	9.89232658176534
H	4.30636265221612	2.09080609838356	11.58251316131708
H	3.12775554151184	4.07425910826579	9.42272358130781
H	3.47838522891129	4.43406381026598	11.14193276543179
H	-1.47730134274962	2.11958550892862	1.97408798160222
H	0.09755604200949	2.21664196989249	2.81000868198819
H	-0.18047481519243	3.15602609749362	1.31937806244326
H	-0.75523512404044	1.86374498119565	11.74828479068664
H	1.20780314567385	-0.22332155548497	10.94752458735992
H	1.91222725585898	2.64088420110415	11.88715946634883
H	4.11476422027922	14.37796154760032	2.65087203563188
H	3.61602956883347	15.19655572068204	4.15821497952614
H	3.37149174805736	13.44022293800540	3.97985681919960
H	3.45702233022367	3.47234038253258	4.39686402558334
H	6.08779096801143	14.91967502110352	4.08169778498531
H	8.49210652142544	13.79670094681754	2.92579204587544
H	5.96203976983630	13.49863423172053	1.42132890255526
H	2.37115968352955	11.57824468886872	6.50768359693036

H	2.52768814187395	6.74004167508786	6.24103075936518
H	-0.22022448970194	8.08083213767380	5.75239967730585
H	0.33420176441094	7.47009976498036	7.33829474477841
H	0.10774301630726	6.33880824663520	5.97604797929284
H	4.17360477158747	8.20658790161773	8.14825420893463
H	-2.01876145081140	1.13755604955026	4.42314649290490
H	-2.26541919686149	5.89153205282688	5.14947219709991
H	-0.32567717731125	4.67709640111960	3.29699932262364
H	8.23341296322987	11.32431736275654	9.28403296422445
H	6.86679260862871	10.29750052316773	9.79560133545549
H	8.03788536975164	10.88886664095685	11.00555304418889
H	8.81418863989090	7.13965565274345	10.50304308661131
H	8.44166035382724	8.34890306172164	11.76264617719987
H	7.20292858502382	7.92838918182442	10.55158416460564
H	13.00507244512576	10.26207734458996	6.75990906480242
H	14.87988081276497	8.93841719765469	14.94733334910129
H	11.08480645001546	11.88956144756268	6.71772911627844
H	13.10156619139420	8.96337859780845	7.98959391536150
H	14.30206853410900	9.20607728738954	13.28618809794510
H	13.38408015649851	10.66448862546469	8.45675425954654
H	11.26362733447106	12.35954389410969	8.42966489757955
H	9.69979227689590	11.73228882868447	7.83217410155149
H	13.04698721840543	7.30419571950973	15.46040617655648
H	12.92848275808630	8.37965352765118	11.66001347553915
H	12.35310193473729	7.44766951826387	13.81987317046431
H	12.78309390704432	5.84437101459075	14.46032663906495
H	11.27020791522055	9.46062425751201	12.95372727488070
H	9.61451448552777	10.05908636977439	12.67363546006766
H	14.96456021468685	2.76504008175372	14.18654192555416
H	10.84763343236330	11.14121299322303	13.38337809420381
H	15.55440597516926	8.98658648177771	11.25228100376971
H	16.92166107663365	8.69332675951306	10.14022781823576
H	15.99751755021497	8.71067847097322	13.57199439845495
H	15.28549363136049	8.10127404012330	9.72721671190173
H	17.87511647533297	6.44447739370457	9.77871595058682
H	10.39450121402136	7.01618099147742	8.41989434702644
H	16.30963228425038	5.66327703768454	9.42919083745241
H	13.31870652656846	4.34723799763100	10.10697318152022
H	17.36620349888062	5.01846652445592	10.72538887401672
H	15.60245579334712	3.57509246961085	10.57778797760142
H	14.13193196572004	2.92679965351270	11.36236037213109
H	13.46507972117606	3.63021117282398	13.76310644775690
H	15.73128654931473	2.32698220727290	11.85013837517930
H	14.69161932894963	4.41625696744898	14.80588707992984
H	11.77837651870578	3.50517231565305	8.57637669702719
H	8.61392185503402	8.88359705669105	8.71335966266724
H	10.72416127501492	9.48226869236456	7.39784064391592
H	15.22595107381245	6.52468507734313	14.52065255188683
H	17.04487952814578	7.09780712947199	12.07735303148565
H	16.32882630210094	4.44371976991315	12.91564882222261
H	9.18965124504983	-4.57648030767823	13.69576473270466
H	8.50648871738611	-3.16770174791143	14.55988525517900
H	6.74544245449643	-3.78278113169144	16.12139772129492
H	6.73137429613764	-4.93249314600571	14.06771424983762
H	5.85456755959869	-2.69257868228817	17.22168410617170

H	7.12211109714316	-2.03425986198616	16.15572494381830
H	6.10739274626258	2.33738433963341	16.62328885660704
H	5.95134684476053	2.07734872111895	14.85749905167140
H	8.02522235037360	0.97105029668400	15.86088736085774
H	4.44760700391415	-3.30877219946841	15.26408348813943
H	8.43649554316492	2.63266245066869	17.68787372962245
H	6.52158355348570	3.64888761542455	15.48766525944778
H	7.16370274479055	-0.42270648027197	14.39816137686064
H	4.04783867044144	-1.14740299240534	16.39043884494325
H	5.24903683284387	-0.31992682181440	15.36563091736318
H	10.35537484649100	0.12779353444632	15.66055944573999
H	9.92149617708320	2.36262019652210	16.72995279214159
H	11.15612284107008	2.28459543418590	14.64338087563333
H	4.10930238050803	-4.51145441074846	12.73349726358284
H	8.96299880839250	3.85126889471758	16.49526444498450
H	3.74138107554765	-0.92672989629789	14.64224756251510
H	2.66472820160661	-2.85507559014899	13.93310813763778
H	10.28840040921589	-0.63235158955852	14.04335132729317
H	6.66894273180629	4.44123738218656	13.37476175806881
H	2.78990899018279	-1.71580374744179	12.55643731756382
H	6.80031866729900	3.42547564379111	11.90854710243498
H	11.40415119865925	2.57777325087940	12.16783909897168
H	8.89744097916992	5.19705222228167	14.61379456737151
H	1.95534284147673	-3.27875433659648	12.35131440591782
H	10.47916903317307	4.63992428130734	13.99143188714747
H	10.91570170956380	0.88656169765184	11.88229801788843
H	7.35829621404308	5.13281092244466	11.88337153681875
H	9.52260055181226	5.82781195226391	13.06358027653844
H	8.85797034217425	-3.05427895766904	12.81583115563635
H	7.87641324727481	-5.85976078740289	12.01964113965986
H	7.39250469315722	-4.42227512946070	11.08238243099796
H	6.14481645405555	-5.54401758092638	11.71404143486069
H	6.16488661297582	-1.83881971776514	10.42199774682045
H	5.09696076321962	-3.93156146957987	10.42514236953234
H	4.07769150730684	-2.45974628682091	10.41857278923855
H	3.31659131950834	-4.06716009076444	10.44546037649416
H	11.86251421148226	-0.14787362915309	14.73958951598977
H	12.44988786937555	1.24008677959309	12.72787327741040
H	8.53411838458275	1.72276943220639	10.89091287616259
H	6.61244217733815	-0.54702413279795	8.55931807889342
H	12.67853173871223	12.12731708103043	12.01913283927280
H	13.29333413983500	10.57022062586582	11.39986026262978
H	12.80738435209119	11.85050548070742	10.25842481441740
H	10.43439847262202	11.68314069177063	10.96146015614117
H	9.30916344524795	3.60490118155656	11.99404296134079
H	7.60334541107441	4.42491359825937	-2.03640812671401
H	14.95771107180918	3.87617886114922	2.04524473133825
H	5.07319428473915	-3.01355908094122	1.53475621869037
H	12.47541827916766	-3.51976785843503	5.45106898483997

3b at the PBE-D3 level

```
414
Coordinates from ORCA-job
  C   12.15664560747696      4.79103845222574      3.44686819291944
  C   12.30372244491790      4.45642213460947      4.79385560290803
  C   12.96595136800422      3.28015822614356      5.17382968753684
  C   13.49337878689873      2.39674289941237      4.23387756823379
  C   13.33833958570609      2.69037529496421      2.85790935304840
  C   12.67819158944696      3.89234207064444      2.49821284645146
  C   12.86382905834476      3.14538244897166      6.65571817209392
  N   12.16098852792385      4.28756171346695      7.10753969182016
  C   11.74185407385065      5.09716792851509      6.01563790064842
  C   11.44784747395457      4.23539501677354      8.38255297635079
  C   11.53130271531823      5.48546289992160      9.24606343352940
  C   10.66851002716227      6.57332418306181      9.02593852159062
  C   10.71902902914857      7.73227968114835      9.82594799743744
  C   11.66341438648313      7.75713101030249     10.87430231795825
  C   12.54327815649417      6.68077184034202     11.14065727774334
  C   12.46275294441483      5.55016401511978     10.29698819128337
  Si   9.55471393128409      9.16270879744199      9.35136337623126
  C    9.81992555129384     10.71639193002844     10.44787486627650
  C   11.20094292125150     11.37105238207001     10.27751899233127
  Si  13.86049841889900      6.91233919555761     12.49281554264263
  C   14.50341593782836      5.28122792183771     13.31230701839107
  C   14.79053799349256      4.10109983307961     12.36679236742248
  O   11.01931706560735      6.07706406842991      6.09360170642698
  O   13.23993121852561      2.22995813759624      7.37274075508472
  C   13.77454883805060      1.68345461444826      1.77893312520526
  C   14.28474214840397      2.40170282549460      0.51203358715813
  C    9.98727708575652      3.83374204975913      8.09640162328819
  O    9.77413101620798      3.34779176454531      6.93842128985108
  Rh   7.91477810628207      3.07143063638160      6.19410033287214
  O    7.79001874157897      1.26885150008935      7.09094537684028
  C    7.24596958702874      1.10969658429264      8.23164668406528
  O    6.58892570461383      1.97463523013895      8.88303846691497
  Bi   6.85357296031429      4.26960661035920      8.21659280579594
  O    4.88979658809714      3.98657959778597      6.83250058643481
  C    5.01439131564645      3.20238025480403      5.84740152582192
  C    3.75026926165084      2.69395445138819      5.12525048423494
  N    4.09527382062572      1.42254109281103      4.48686052879349
  C    4.73492803305948      0.36213584566418      5.18472996547367
  C    5.47753211960965     -0.41657742333742      4.15425574081676
  C    5.33659673100874      0.23912438644505      2.91986337499084
  C    4.44502649742513      1.41709998924948      3.11624194610593
  C    6.06646782794326     -0.14220350768664      1.79839910417737
  C    6.98240240226169     -1.21844913686944      1.91067568712669
  C    7.07350733837859     -1.89529999020226      3.15011087694581
  C    6.33080642112843     -1.51123820289077      4.28463912812961
  O    4.72103018702186      0.20711527199463      6.39576837558710
  O    4.10219005233842      2.26890627835302      2.30979734910582
  C    7.91152064581513     -1.54439412807882      0.72892267046068
  C    8.64630745740038     -2.88330031251246      0.91497766177049
```

C	7.44468496231285	-0.28723339325098	8.85303863347150
N	8.63230958572656	-0.89183247750607	8.25656264610794
C	8.53914066079112	-1.97218556274361	7.35682836904188
C	9.87393926322612	-2.06699964808987	6.69339263517308
C	10.67607017906869	-1.01892937340053	7.16762225235471
C	9.88818200817498	-0.23803658092823	8.16355994470317
C	11.96143352372340	-0.81385026033638	6.66075990499706
C	12.40926917762921	-1.70458782318869	5.66894170212491
C	11.62095556069664	-2.77992772836667	5.18340390526149
C	10.32246279077828	-2.95429168113823	5.71557704535142
O	7.53093137858307	-2.63624817968395	7.16103762071812
O	10.20409815689203	0.77308795022845	8.76826377722344
H	13.41766872204376	-1.55597575619423	5.25301792668716
C	7.42925760079146	-0.26606561422381	10.37199338216253
C	6.65069932605608	-1.19915388414371	11.07777125455882
C	6.56837841746621	-1.17040591067500	12.48737239740055
C	7.31764355502639	-0.17578126773448	13.16199700626197
C	8.11265659521006	0.77435353991956	12.48719691792552
C	8.15057184259910	0.70934491191123	11.08014720493180
Si	9.11183358197548	2.14478796941463	13.34738775537185
C	8.79716625920374	3.74578623399149	12.34294098317613
C	7.30972150969174	4.01823967116282	12.06948684154786
Si	5.59708303240333	-2.41458636617934	13.54944235130131
C	4.15386056865799	-3.35471923600395	12.65979121990220
C	2.76490229914266	-2.70893112777780	12.79333549726113
C	8.59938134683189	2.23409029264072	15.19800142347315
C	7.17834547949035	2.78472908413018	15.40121711914585
C	10.97492846071827	1.68265465158818	13.24955712759322
C	11.21387699449392	0.28855510484034	13.85146621520981
C	9.61663801769815	2.99043596218548	16.06951034179474
C	6.86797994131539	-3.73779947626676	14.15546768559906
C	8.19586229181832	-3.12165641148612	14.62647583943940
C	4.80893769697941	-1.50548569324709	15.06429980133939
C	5.68648222406846	-1.34142050740470	16.31586241435767
C	7.14211796821065	-4.82641758475941	13.10520279494818
C	11.55285884652022	1.78845646987837	11.83078763416805
C	9.52721377630292	4.97419813929585	12.90598803826479
C	4.17045252568153	-0.16240617466645	14.66684385048903
C	4.38953003596227	-3.74905482734528	11.19043555417384
O	9.11934791146041	4.04958557284030	8.99395811087686
O	7.56359241317405	6.18916833971234	6.83994953497030
C	7.84759939187327	5.92908566826634	5.63348728988688
C	7.88429451609088	7.06519769625234	4.59345520220224
C	6.91569856638154	8.20900168875358	4.84196476605042
C	5.67486983907819	8.00509266013629	5.46767184158698
C	4.75985652214953	9.06077944249536	5.65599517285826
C	5.12977249438780	10.32897939123511	5.14753741176135
C	6.36051662791154	10.57425012452528	4.49124653412287
C	7.24882947497567	9.48540023114081	4.35891423189928
Si	3.10385817312689	8.72990714219866	6.56141318114704
C	2.89727031986010	10.05569934902341	7.95119624507112
C	2.57649781786969	11.47156516637059	7.44437698343984
Si	6.65449002660194	12.27975969460656	3.69136891727890
C	5.85291130058912	13.66834513104686	4.76926420251385
C	6.14683654574626	13.47779212266291	6.26825630234421

C	7.72233992022961	8.56296351574814	9.47558517302114
C	6.71141474807545	9.70980800130313	9.64189964990436
C	9.88040929828024	9.52472276610910	7.49082849770250
C	11.35993521175456	9.51581841097119	7.08033773619332
C	7.51608363342117	7.47665690581981	10.53985816196521
C	15.35131217375475	7.82021998112179	11.66128746524373
C	14.89106883077175	8.92853331802172	10.69937713388099
C	13.18094666981684	8.01352098420432	13.93140854969397
C	13.33749351665567	9.53422643505581	13.76659291128130
C	16.33567061542179	6.88206141976540	10.94680387828047
O	8.11478414073750	4.77939531971841	5.15198302773859
O	6.10648848246906	2.76442114705675	5.35458145975042
N	7.72391751106340	6.47880769649286	3.26823124911854
C	8.78499938606151	6.38910606490131	2.35098681117319
C	8.32530766306317	5.43772166174266	1.29480216754912
C	7.04391231060517	4.97472884256906	1.64355936663191
C	6.64302923625598	5.62456899933370	2.92602281564848
C	6.39868085345849	4.01598684790850	0.86515868189842
C	7.07782094498732	3.53624736200671	-0.27418279787015
C	8.36350199109645	3.99190645613268	-0.64688579829427
C	8.99086880552681	4.97061669702395	0.16671360590760
O	9.85888458061443	6.96576396473826	2.45939883855256
O	5.63773793911770	5.46830343429731	3.59875778121807
C	9.09898108365856	3.46793151356032	-1.89230299630010
C	10.45549337028782	2.86124694797873	-1.46480579012710
C	8.29828820689564	2.38434927380770	-2.63524434972156
C	9.34270386305520	4.64935270779177	-2.85999125601725
C	8.49552521907376	12.80926265687624	3.38679317743557
C	9.13868166751364	13.68015433927514	4.47961803670296
C	5.80728093100364	12.22202095102296	1.95337952639706
C	6.68224066133483	11.56794674018470	0.87213224219130
C	9.47621578191476	11.69900394920289	2.96788259539430
C	3.23931381044046	6.96385752618437	7.31388268079196
C	4.24539712495600	6.89235494820122	8.47218150079136
C	1.66558815009908	8.72603104334426	5.27366074349318
C	1.88817142513436	9.67833554590558	4.08972903574685
C	1.90547717738197	6.30208287049325	7.69148074320600
C	4.42953033117506	11.54383015960721	1.98581603734610
C	4.36451720894220	13.96340607350011	4.52445081650463
C	0.27927594959441	8.95031853213449	5.90001190106603
C	2.49184315784872	2.71076053640167	5.97116517802595
C	1.35129622409669	3.35707963421002	5.46419768430245
C	0.14642257757651	3.42945530983936	6.19564423689579
C	0.14293341332425	2.83802215552968	7.48131805107349
C	1.27406976277523	2.20152565618163	8.04116948794140
C	2.44594077930974	2.14092160005752	7.25589905268031
Si	-1.40013360176521	4.29761712207957	5.48344329496751
C	-2.73185553524883	2.93350565028468	5.19771433446469
C	-3.20173549387163	2.25413788127777	6.49428781303014
Si	1.29556863836857	1.46360868841738	9.79850364431436
C	2.88647391497836	2.04302869326316	10.74713535329239
C	4.04519324787971	1.03871874784028	10.81364815161402
C	1.30792457119459	-0.46298522827259	9.68942470465021
C	-0.06876809666873	-1.11543303576393	9.49666162986652
C	-0.17990108341573	2.07661443765830	10.88779452450401

C	-1.61520076354557	1.85968559066492	10.37371128415166
C	2.29250793624491	-0.98022272891118	8.62767880177554
C	8.96235640549755	-0.40978551078766	0.64062613446722
C	7.11139561243108	-1.60979385030061	-0.58988458394525
C	12.53186813960259	0.83203348814804	1.42157612718203
C	14.89203703468058	0.75255487150390	2.28792731203372
C	9.16536781469236	10.79769123770239	7.01853034416367
C	9.48134081956433	10.47883126276524	11.92995407147525
C	11.74161597147796	7.65901589558839	14.34500651289091
C	13.66302493926311	4.78341448243440	14.50051622376615
C	-0.01357031614449	3.53970002665024	11.33785613312873
C	3.39352944573046	3.42083376015004	10.28741718222199
C	-0.86135344014056	5.07940259177198	3.80362636336540
C	-1.71740843640771	6.25792289159287	3.31248105466081
C	-1.95981288305428	5.72570618545710	6.65747939938464
C	-3.41325233995414	6.17400742112945	6.42475029134257
C	-0.68607000528311	4.03028547042141	2.69132947544515
C	-1.70737043169073	5.50909239474825	8.15637290932304
C	-3.93233545033431	3.38387799414308	4.34784066445092
C	1.90251003043713	9.65064612339125	9.05334478922926
H	5.81005550445789	14.35888350919145	6.85679021022522
H	5.62177419762546	12.59024668940854	6.67658003196764
H	7.22541620306116	13.33163685243943	6.47364019380973
H	8.52637792485149	14.56480493271511	4.74305882508843
H	9.31779025138103	13.10600737174285	5.41031670119844
H	10.12806618884554	14.05635038664190	4.13956557224954
H	10.41543690387908	12.13902171640939	2.56831314794657
H	9.76933464556206	11.07402326260926	3.83640157455783
H	9.07246551781813	11.02155702792295	2.19121582920451
H	5.25218173200604	7.25391384152794	8.18024719320235
H	3.92321895242077	7.49584705753096	9.34600386570303
H	4.33828995192817	5.84571132823716	8.83717360852026
H	8.92809793249904	7.45193749455425	4.60766872759479
H	9.99358140359391	5.35969570592497	-0.06547135853493
H	5.41141123033509	3.62119230754331	1.14827424564057
H	6.57853196618956	2.77352618232569	-0.88714855003699
H	8.22054980966564	9.61612024873372	3.86172911288382
H	4.41966242091807	11.16158790258400	5.24930932725650
H	5.42061725951022	6.99229032047911	5.80154866638892
H	6.45615741708575	-2.03123365938010	5.24641343187267
H	7.77361758302688	-2.73487289851210	3.25683486423994
H	5.95615875847983	0.42770053773230	0.86434993126372
H	6.62033559364599	-0.64669714146188	-0.83209119083670
H	6.32523860762332	-2.39002091770973	-0.54333495730160
H	7.78898522545331	-1.85466620778420	-1.43303803697044
H	11.61984310805563	5.70122760059342	3.13700881396253
H	12.54320317377554	4.13159988952190	1.43357178110044
H	13.98427856304964	1.47658692352800	4.57764556926534
H	15.77509361168116	1.32668846813206	2.63368139906979
H	15.22002944683063	0.07751119801351	1.47252009809193
H	14.55296513117111	0.10951691919128	3.12402424899160
H	9.31901258556898	-2.86933713477746	1.79518801000858
H	9.27639676773988	-3.09146049510239	0.02728315813540
H	7.93855085350171	-3.72837101470088	1.03397037292234
H	9.56060175491596	-0.34963934057690	1.57224315440327

H	8.48265908374908	0.57855528027446	0.48976637929697
H	9.65856049503481	-0.58970390323840	-0.20517238526695
H	15.12562724599477	3.08539146281928	0.74616393957276
H	13.49140486930323	2.99209875625742	0.01268888244255
H	14.64402252851844	1.65581366771354	-0.22532211745318
H	12.15200633771750	0.29323469949007	2.31265428647522
H	12.78064770444757	0.08061030682511	0.64306145896396
H	11.71034111291265	1.46918396736402	1.03857258221155
H	8.86989688016327	2.04036779109299	-3.52017934416870
H	8.10965842653116	1.49756579144366	-1.99676138473580
H	7.32285635217952	2.76582779060037	-2.99792250174784
H	9.88011187115201	4.30008249184058	-3.76567665843291
H	8.38330740270967	5.10289518978890	-3.18087705026649
H	9.95317936006288	5.44678709694316	-2.39159926724725
H	11.10082763103498	3.60406778822232	-0.95481994318259
H	11.00914688225655	2.49084140040935	-2.35188377344558
H	9.65743848108022	-3.75931723917413	5.37630642450322
C	12.18951969700499	-3.70906296342440	4.09675929648078
H	12.58236127955383	0.02614737361134	7.01092819239326
H	10.30773693288791	2.00839731867951	-0.77269357125854
H	1.91209760615060	10.74036516092579	4.41548627046197
H	1.06539429557426	9.58767525554631	3.34691461066615
H	2.53315033283927	12.19177461486609	8.29055516006585
H	1.59182196650148	11.51415191080221	6.93471694822391
H	3.33334246846104	11.85704975022022	6.73546364726755
H	3.93030594581251	11.60938512030641	0.99459621184896
H	3.74273967162044	11.98564074936183	2.73283952269670
H	4.52895597248279	10.46690728313577	2.23345114271361
H	2.07175134091978	5.24998721207562	8.00516424102462
H	1.20120339879450	6.26474372453470	6.83795832525529
H	1.39452042671350	6.82298980361422	8.52673183250004
H	2.15741022243625	8.68063279972258	9.52258241359800
H	0.86670254694460	9.56782466002195	8.66609884933347
H	1.88555872599666	10.41326780284574	9.86265094565076
H	7.64298226524489	12.09737997823173	0.71695844437243
H	6.15383508762985	11.55199534600884	-0.10612588866520
H	6.91487497665448	10.51167971443080	1.12765263195064
H	2.84094154216396	9.47491758571006	3.56462519103660
H	-4.58903449991608	2.51911417303618	4.10754746815775
H	-3.93157502720718	1.44404919254172	6.27544673351381
H	-2.36486298736049	1.79627561900138	7.05723651398194
H	0.03216784067229	-2.22005418169743	9.41582007754879
H	2.36684044373205	-2.08901865103060	8.67016029898723
H	-0.56093516813444	-0.76759248657277	8.56322502281392
H	-2.75288959778972	5.94602927176526	3.06294067939801
H	-4.55922882887867	4.12686329782177	4.87971346159587
H	1.95691420190508	-0.71215785608092	7.60477112112103
H	-3.71060741604193	2.97099728734544	7.17317073308975
H	-3.62552019136754	3.84013694351277	3.38570814498355
H	-0.75790786926099	-0.91140246567914	10.33949325307304
H	-1.28262683473710	6.69943389656841	2.38914867322905
H	-1.78560870496705	7.07152172987280	4.06180308884934
H	-1.78220921909204	0.86449223877520	9.92267966399602
H	-0.77250171847534	2.88949739718272	8.08356589091989
H	3.73001204574172	0.03992539769522	11.17355411851408

H	-3.64206161064579	6.37461351626652	5.35998099076289
H	-1.89022984172747	2.61600501024218	9.61126060261188
H	-4.13132334473231	5.40571091625507	6.77971931136712
H	-2.34641409925634	1.97987826456865	11.20218680340248
H	-3.63147514492284	7.10518484789223	6.99204260240636
H	-0.64941471931673	5.27191092543148	8.37613790139377
H	-2.32911117496048	4.68523405798011	8.56615693566269
H	-0.04449951320941	4.23322846504565	10.47121228669744
H	-1.97135337962336	6.42366268149769	8.73137859374623
H	-0.83800737366131	3.83913168776002	12.02121700348443
H	0.93820294988667	3.71887011328695	11.87501820152644
H	2.60193525002687	4.19618147091811	10.29104162728286
H	3.31597089295167	-0.57611565239686	8.74286801487954
H	1.42741521418680	3.82283839102879	4.46934043190050
H	3.34898228505369	1.64642582314148	7.63892017936376
H	4.53693859105078	0.90680395601958	9.83078462056925
H	4.83937378654302	1.39280064177471	11.50548050798353
H	3.78452554698971	3.37492832513295	9.25034050212782
H	4.21734988835792	3.77793011162924	10.94475791029692
H	-1.66488501412769	3.61688014273265	2.37093690191182
H	-0.05845900663964	3.17126923836162	3.00292463392227
H	-0.21209143907755	4.47720942479522	1.79063436739641
H	-0.05233645684764	1.43188254301838	11.79163013897194
H	1.68669180496255	-0.77701987889594	10.68965483642245
H	2.50145562920306	2.15831967913410	11.78835401697543
H	4.14864024291806	14.19092568384473	3.46159636202506
H	4.03579512082597	14.84018515596840	5.12414970713246
H	3.71042101371144	13.11683611615537	4.81942185770266
H	3.60979317220154	3.39410585419453	4.27138643206458
H	6.41041984426638	14.57379273907196	4.43226644219945
H	8.35154455355787	13.47060819983138	2.49778272712904
H	5.66771259051471	13.29518863325866	1.67929219376570
H	3.91227959650011	10.08821240903258	8.41101805260899
H	1.69433841934911	7.68426481533165	4.87329163088738
H	0.16654889435099	9.98952071380577	6.27164287617991
H	0.07708327860270	8.27246027140570	6.75218501642640
H	-0.52466992476487	8.78941070335420	5.14869478124220
H	3.65325673543908	6.35257072108094	6.47972254680383
H	-2.16974283500935	2.17291423150863	4.60684765085834
H	-1.29632398431108	6.56107357533282	6.33515997720761
H	0.14564120587625	5.49862558731224	4.04115579051354
H	6.78855719466646	10.46890414586223	8.83783212346049
H	5.67010518309356	9.32245892756629	9.62266807469916
H	6.83867084143356	10.23821914495949	10.60934031538257
H	8.18864046514322	6.61099945830580	10.37724096104046
H	7.72239441090030	7.84419435390795	11.56744195112886
H	6.46676925240423	7.10534978708301	10.53851398659202
H	11.46252603977688	9.63046574469841	5.97891377016591
H	13.04106073420774	10.05884011532640	14.70097519282316
H	9.30027165630260	10.94187305960875	5.92686685206611
H	11.85781785891301	8.56717964690636	7.36038694070583
H	12.69050089591228	9.93963474724372	12.96106315652120
H	11.92657107042147	10.34668607828352	7.54957257138119
H	9.56532704843958	11.70575837837830	7.51879836425669
H	8.07230711880922	10.76700719025629	7.19599237424235

H	11.44808870594969	8.20820349441098	15.26602407056594
H	11.72607705436335	8.65469379631087	11.50561401230589
H	11.01181054824402	7.93083243490636	13.55588007486696
H	11.60422653100225	6.57932200047856	14.54303344527423
H	10.18314575615604	9.76861763093611	12.41204150195937
H	8.46253716372278	10.06522703621477	12.06847221366847
H	14.13785909730403	3.89386868662003	14.96896796507366
H	9.53581894774494	11.42699713625086	12.50835530366460
H	14.20911643611265	9.65971648750692	11.17530352485225
H	15.76160289471152	9.49619944712431	10.30468888967939
H	14.37926719443388	9.83358140646620	13.53704527595742
H	14.35116678755582	8.50173940810538	9.82880295433743
H	17.16898266173949	7.45989420383619	10.49041854429291
H	9.94937841381158	6.51902707938945	8.19663443710035
H	15.84011981833103	6.32848065631031	10.12070851078219
H	13.13191549442954	4.69259766186112	10.45358175360835
H	16.78814639309842	6.13629110585508	11.62987887031681
H	15.34446867634274	4.38861760713232	11.45301815533688
H	13.84882827496592	3.60993640135735	12.05032891255264
H	12.64847050514953	4.46974269287803	14.17567089590674
H	15.38942559139936	3.32221642292915	12.88688817806629
H	13.54039815871358	5.54550862593133	15.29448207498727
H	11.90475498533039	3.37748274855814	8.92392650747192
H	7.54147019055277	8.09345192095767	8.48187907446698
H	9.39078419620111	8.65946669329976	6.98592538842050
H	13.85340920421282	7.71709467651037	14.77167251919225
H	15.89264936591816	8.29711256220002	12.51257377902164
H	15.48314692241573	5.62583156653749	13.72463456665640
H	8.87333991042344	-3.90554892406722	15.03003490881997
H	8.06419274420790	-2.35953270584865	15.41857446331802
H	6.11359287546030	-2.30264532717648	16.66489441121843
H	6.37424251191578	-4.22162455308473	15.03181855943222
H	5.09252492693441	-0.91873750265116	17.15576137931471
H	6.53173104672361	-0.64134163850047	16.14642274146682
H	6.84913816397138	2.67371837085654	16.45747869980213
H	6.42809195009888	2.27400003783293	14.76362170967248
H	8.60159948836852	1.16937044291599	15.53261769019573
H	3.98365855256827	-2.20051801956883	15.34819193057153
H	9.30185122903291	2.99706097589795	17.13594075613895
H	7.13114080819250	3.86699637648092	15.16010037613730
H	7.27384033373735	-0.13605692505416	14.26111749974828
H	3.59044352596024	0.26979948510489	15.51130300058907
H	4.94311814778805	0.58121230181830	14.38430445732103
H	10.86878701678244	0.21738684812663	14.90388904617154
H	10.62823757537535	2.53884158497526	16.02731449170519
H	11.50014281176200	2.43045310421998	13.88741570172599
H	4.12905979239830	-4.29820340809769	13.25764005894809
H	9.71635830585293	4.05031102856698	15.75516314352043
H	3.48479839336246	-0.25170925907208	13.80218079276396
H	2.47690559076911	-2.50920218641726	13.84397825265376
H	10.67270055965394	-0.49220152186219	13.27581440407113
H	6.75430631767056	4.29492661829998	12.98829094521800
H	2.71085190338008	-1.74658935053388	12.24250629876774
H	6.80319226965899	3.13424074622274	11.63162045073354
H	11.46609524992441	2.81588365795119	11.42193186339210

```
  H   9.08116715708780      5.31841118569065     13.86294361471703
  H   1.98351246753880     -3.36863505387096     12.35636965779484
  H   10.60116725653420      4.77072034280652     13.09491108806674
  H   11.04519306170098      1.10976375829071     11.11587845257134
  H   7.18842705805655      4.86798192531330     11.36378887747800
  H   9.48969227938441      5.82810284693352     12.19738344947488
  H   8.72664351728390     -2.62833448419255     13.78571644388578
  H   7.87884805047179     -5.56742106466789     13.48612887315373
  H   7.57448555144354     -4.39099608033024     12.17859255808108
  H   6.22999494752459     -5.38726854856810     12.81979784001376
  H   6.08310113409695     -1.94302721735901     10.50189340141686
  H   5.39595576067265     -4.16901352085296     11.00034710841339
  H   4.25552995143821     -2.87572896287610     10.51933244739750
  H   3.64600210955078     -4.50908955501061     10.86605413089820
  H   12.29321615336178      0.02213174367897     13.83786444487580
  H   12.63120361750454      1.51760089423407     11.82126430380899
  H   8.74018368216422      1.43957135862010     10.50935765026470
  H   6.59957052768435     -0.91068454267731      8.48429804814076
  H   11.29496025172280     12.27589900781883     10.91729090784171
  H   12.02840037469324     10.68649863680114     10.55715128145791
  H   11.38461445615400     11.68575988566795      9.23112067057277
  H   9.06408908835042     11.43312409414786     10.05018668131172
  H   9.26153125664007      3.51940137604734     11.35728472139853
  C   11.20711135914727     -4.83430826981557      3.72712245071548
  C   12.48682414389546     -2.87770042099411      2.82752828796533
  C   13.49630088838785     -4.35444167063018      4.61190719786760
  H   12.89282815090219     -3.52826102305894      2.02561102716863
  H   11.56648293811799     -2.39278574035702      2.44328941210200
  H   13.23022495723451     -2.07934169795189      3.02076256808049
  H   11.65514575585730     -5.48299028434046      2.94814284844751
  H   10.96870037206494     -5.47533462592522      4.59937164177405
  H   10.25488897206184     -4.43861902680490      3.31988321472093
  H   13.92126281630436     -5.02924417914937      3.84076780951839
  H   14.26544196151300     -3.59511142295699      4.85479614354915
  H   13.30934777302227     -4.95119327833889      5.52716583545050
```

3b at the PBE level

```
414
Coordinates from ORCA-job
  C   12.53094423648720      5.85278267441272      3.43025844721211
  C   12.53977635595956      5.29402386598636      4.70693775909854
  C   13.41207476231544      4.23725743842940      5.02251678819656
  C   14.30874699506555      3.72654028951820      4.08909422746461
  C   14.34056873990823      4.28576096849510      2.78437775163642
  C   13.43973402674307      5.33481862293507      2.48439366918367
  C   13.13505656611053      3.80429427214685      6.42819973539210
  N   12.14119975396690      4.68526038105405      6.90418857219013
  C   11.70981772486661      5.59435984116746      5.90931930979820
```

C	11.48243900006685	4.51283499185620	8.19702203682879
C	11.84354273995826	5.59902317966058	9.20114612146086
C	11.20897025198296	6.85271535495708	9.18195810106784
C	11.57424442382603	7.87874223035283	10.08076584118628
C	12.61327626919924	7.60547231155118	10.99868438583359
C	13.29161357557848	6.36080149113489	11.04491573685675
C	12.87915720599598	5.36687766471157	10.12738084051960
Si	10.67980539539771	9.55262015318329	9.87001480152846
C	11.08602642645932	10.82954408483145	11.25707907524216
C	12.52783629938345	11.36963127949060	11.24381804475495
Si	14.80438990050780	6.16714021610534	12.20075235819738
C	15.27804087485651	4.34114295916870	12.67368414077360
C	15.22939753548651	3.28409638642488	11.55274021198452
O	10.82311371991231	6.42338704744530	6.05048691727580
O	13.61888095318730	2.87929373907506	7.06388377670228
C	15.33827138430608	3.72840589870713	1.74987571207982
C	15.23540154485254	4.44248176472002	0.38916932687054
C	9.97131103885148	4.29545041706896	7.96080596769255
O	9.69799108871723	3.71019451965267	6.86067652265938
Rh	7.80641311225195	3.34521223237984	6.23636937555748
O	7.82746304855473	1.58410815024487	7.23542070287695
C	7.35236632983471	1.45168666537374	8.41159102155676
O	6.74498178906479	2.33435450409958	9.08558691828179
Bi	6.79166873604636	4.60231025552314	8.22608606031003
O	4.75122727450302	4.10554524210446	6.96695101372220
C	4.88385030541148	3.30796786201970	5.99252331622626
C	3.63424387445938	2.77389753850341	5.25990749349909
N	3.92562217054245	1.42905204527039	4.76441100860114
C	4.47343008464976	0.39433908808998	5.55694999040227
C	4.76374222956577	-0.72327366652026	4.61210863887720
C	4.37986512976248	-0.32050605983690	3.32051228701207
C	3.87238182886977	1.08519501850227	3.39810618430102
C	4.53294904472657	-1.15553166805910	2.21822678322441
C	5.09624603739809	-2.44602932488258	2.40246098234334
C	5.49569374908878	-2.82090887341786	3.70699963367785
C	5.33837942307622	-1.97528677882828	4.82490699492466
O	4.66368581149735	0.46038253657237	6.76299025197644
O	3.49975596658915	1.82522307854262	2.49916307758878
C	5.25654540216427	-3.37881931526830	1.18569867641174
C	5.88964722622523	-4.73202555926155	1.56001031538051
C	7.53353529796943	0.04260995890140	9.01396187198530
N	8.76174114673499	-0.54567353662795	8.48704701529991
C	8.77694871752709	-1.73197701046049	7.72417887407633
C	10.18858322114166	-1.88438383693330	7.24983139007180
C	10.92367118941905	-0.77371801588902	7.69197558590834
C	10.01829701003101	0.10214710752965	8.49135128839582
C	12.26640857545546	-0.61952607304012	7.33971273080543
C	12.84730715173808	-1.62789813322316	6.55056854003779
C	12.12973358605359	-2.76844407507860	6.10530440937924
C	10.76598607938159	-2.88487288971508	6.46681992022234
O	7.80487622152569	-2.44044468800052	7.50617240509529
O	10.25869024410680	1.16668992096101	9.04164693585587
H	13.90410129249352	-1.51582460928033	6.26366402061458
C	7.42496468123749	-0.03905185531929	10.53012852393386
C	6.71730752249011	-1.10935465069416	11.10719750853153

C	6.60181707357254	-1.25537839431473	12.50986048334198
C	7.23422445479010	-0.27520733428354	13.31287490010857
C	7.95208358485137	0.81780146567169	12.77220398143633
C	8.04013501579673	0.90995972221328	11.36656964087458
Si	8.84535421844434	2.12263522189606	13.84589435022371
C	8.73661376601283	3.81787487762875	12.92968261618882
C	7.31541027802434	4.22484039615063	12.49865711551687
Si	5.78977100114897	-2.79688033801685	13.29703528602959
C	4.17957697797896	-3.42046033600818	12.39935083389444
C	2.86662257014938	-2.78019514734515	12.88607164985544
C	8.07099423617250	2.09755926976857	15.61386020321920
C	6.61403081011105	2.59176854475052	15.65660126074987
C	10.69884512484559	1.59521331000178	13.97807929018007
C	10.84666566458476	0.22591320610879	14.66470122636146
C	8.92618726821211	2.79941415623709	16.68415310936591
C	7.10722521740550	-4.22281230511294	13.23677305686150
C	8.52350603162988	-3.72154032429606	13.57358615668736
C	5.29542116704717	-2.50639027499587	15.15222282946841
C	6.39868244617512	-2.73387391576527	16.20135924671430
C	7.13470636184434	-5.02547001301975	11.92484499656382
C	11.41676073673365	1.61006496440723	12.61797173800011
C	9.43945497528356	4.95925245067891	13.68651816345677
C	4.56715460563336	-1.17561164578258	15.42550352720607
C	4.20018118146175	-3.41283808572578	10.85928619522807
O	9.14565795902293	4.67115586292575	8.84481807673258
O	7.18903609354180	6.46872953725236	6.71285950615338
C	7.50894177056623	6.16827886334021	5.52501687958338
C	7.52273218214126	7.27442761142233	4.44800062504725
C	6.59670810547386	8.45998174723273	4.69665623369016
C	5.32187238850855	8.30254157992983	5.26816609799696
C	4.44540460876724	9.39788026779002	5.42477688491469
C	4.88887771935867	10.66153209930418	4.97449938680889
C	6.16374711692622	10.86910431966573	4.39221583320299
C	7.00346278846666	9.73791166089229	4.26545043944278
Si	2.77875502556035	9.09525921132316	6.31329059259391
C	1.70159305084419	10.68489091925668	6.12698261722694
C	1.25136724839444	10.93614213645705	4.67564440185931
Si	6.60746860474135	12.56591234959754	3.62915714619344
C	5.54482995573639	14.00720887476415	4.38129308878774
C	5.45316031490647	13.99713538476931	5.92012382168407
C	8.76961865316919	9.20778777056488	9.82139674367041
C	7.91723606205431	10.47004784487180	10.04922402169362
C	11.20726029371646	10.19202130109112	8.11823544556714
C	12.70815345561670	10.02858278279512	7.81811644341159
C	8.30512791724194	8.06763873803845	10.74692627411339
C	16.33486315151323	6.95576295973569	11.30210547319701
C	16.00973684271343	8.30054147464154	10.62789380328063
C	14.52275712979365	7.08913415139187	13.88738079776761
C	14.94608506271662	8.56610907343337	13.96658817168673
C	17.02790157836802	6.02159977831788	10.29515492768889
O	7.85552802348199	5.01775453617628	5.09654122655654
O	5.98136455538020	2.89476608013895	5.48834210564742
N	7.30942639172004	6.66415179556371	3.13875857467781
C	8.26809861553212	6.70218992640752	2.10587338654816
C	7.71970902667396	5.82873336841940	1.02120424291264

C	6.50889127097560	5.27348781405510	1.47158440915257
C	6.22746562569506	5.80448562358195	2.83659529900732
C	5.80705980589213	4.36224114426128	0.68478442616810
C	6.34492051700720	4.03997621616969	-0.57860627704099
C	7.55080604912481	4.60134625669386	-1.06150001956511
C	8.24945978922783	5.51194675481758	-0.22557432054347
O	9.32226017223688	7.32017197295616	2.14607853084901
O	5.28889333253944	5.56822748620343	3.58232733601646
C	8.13024044571803	4.25540883624644	-2.44728452236962
C	9.54202510030426	3.64426741017669	-2.27248654407308
C	7.25659871499539	3.24599546132559	-3.21513723224073
C	8.22940656144388	5.55191503159470	-3.28730952229312
C	8.46939153833078	13.10097135397352	3.81695600507693
C	8.79381616353660	13.94637897556888	5.06187644539132
C	6.22226335518758	12.44046375326127	1.72972376533712
C	7.36027480870483	11.83704230646963	0.88813064947510
C	9.51557318131734	11.97932110100937	3.68044431492479
C	3.30561503051119	8.78390494173343	8.15037181597289
C	3.83558830162803	10.07058634776081	8.80694041247082
C	1.97698902559200	7.49444634307764	5.58635338258339
C	2.11755542586078	7.35751765185683	4.05913893516250
C	2.29171845510056	8.06150677023357	9.05291027292822
C	4.90712585786659	11.69352354170710	1.44271779808084
C	4.15283885830755	14.23670720854608	3.76429432624500
C	0.50894565153685	7.29456476360288	6.00892941300372
C	2.30394284679688	2.83753475594368	5.99822003861747
C	1.15039675231457	3.18583069388747	5.26941883617345
C	-0.14341624553200	3.12374899826800	5.83601204873682
C	-0.22380829760493	2.74378521625807	7.20081055988360
C	0.91314520853402	2.43147086696403	7.98532150925644
C	2.17446412700462	2.46279920781455	7.34676352526478
Si	-1.70729353701532	3.45059077667768	4.76090882207742
C	-2.64759553858798	1.76298458239692	4.61004263344083
C	-3.20032926332198	1.22166919130299	5.94032720348219
Si	0.85222994401630	1.87611661702901	9.81852378453481
C	2.43739690281123	2.48374246363845	10.76434710109453
C	3.64512836776400	1.52957436046950	10.77328670569050
C	0.81075013662451	-0.06412536073267	9.86911006010476
C	-0.59016108191291	-0.69382171901649	9.79220205425742
C	-0.65624180655974	2.55515302958884	10.83760512484668
C	-2.05193153069677	2.43798780322984	10.19629726418416
C	1.72885436887589	-0.69617930372893	8.80780227274952
C	6.16115080279044	-2.69172234702869	0.13387514963473
C	3.86156436010030	-3.64721287887036	0.56989032467527
C	15.05579055638474	2.22186437133855	1.53224261581587
C	16.77817145705836	3.91102429671201	2.28870817566296
C	10.73259440472184	11.62117519599764	7.80379379846534
C	10.68070775767841	10.34656067931438	12.66221943671960
C	13.09934796825265	6.90407836853283	14.44579492987186
C	14.57501058300802	3.78132908605580	13.92419280304155
C	-0.47373492689574	3.98112439529330	11.38994994063343
C	2.88165918535442	3.91082503050631	10.38608988599699
C	-1.07329629886914	4.01624606835090	3.01652625675340
C	-2.06858225672667	4.84701876348498	2.18540017196979
C	-2.76707239436435	4.89087318958885	5.51884094187585

C	-4.21873219085756	4.93399898398539	5.00407808586266
C	-0.48926222309675	2.87112254219509	2.16604610726911
C	-2.75693771123192	4.99676905515354	7.05313912511421
C	-3.73667300848033	1.73361531156082	3.52123425192095
C	0.50907526110718	10.77690125039875	7.09642171289900
H	5.02211313075621	14.95182189778127	6.29411155347373
H	4.79563475055798	13.18286659633917	6.28739697998423
H	6.43524077254007	13.86275370769629	6.41295772188188
H	8.15897406665296	14.84984575902416	5.15193390706729
H	8.68262971915558	13.35948974093611	5.99782199493800
H	9.85022096124708	14.29327693171894	5.02845615768373
H	10.53992680077234	12.40900932874929	3.62608568673066
H	9.49992983512340	11.30735167519831	4.56319234997021
H	9.37717436144261	11.35198828891763	2.77840414729641
H	4.61367301139311	10.56611912640066	8.18968073447597
H	3.02440051955789	10.81021746190942	8.97693839040658
H	4.28703440138471	9.85746909792521	9.80108994437210
H	8.57482648856081	7.63764903169846	4.41785962824106
H	9.20090252678841	5.96861784288579	-0.53726325021414
H	4.88214790761829	3.88894514116703	1.04992648097297
H	5.79938559533860	3.31739867950134	-1.20042711423866
H	7.99927978722545	9.83430657826782	3.80771455439490
H	4.21080436279309	11.52238461011846	5.07560321424694
H	5.00915877761974	7.29746332391584	5.58747134498539
H	5.68359191729014	-2.28517111818514	5.82327765591652
H	5.95219507484116	-3.80659692757869	3.87006947421364
H	4.21850322371102	-0.79901697362636	1.22566364758369
H	3.36643954935419	-2.71265299142225	0.23874044171923
H	3.19111227897248	-4.14352076711967	1.30052617727330
H	3.95359721774616	-4.30966999079433	-0.31565386983564
H	11.82185920621963	6.65111384488479	3.16179325104289
H	13.43418056027982	5.76906239694974	1.47564167069492
H	14.97167896583461	2.89668760531344	4.37694854091915
H	17.01103225553823	4.98289677122430	2.45178033001485
H	17.51439396917754	3.50737531758248	1.56308696265253
H	16.93195525992525	3.38218666157897	3.25036185376981
H	6.90790016677678	-4.61232560189363	1.98268376637652
H	5.97953443022750	-5.36452704312062	0.65381496949829
H	5.27379947639531	-5.29074984754932	2.29356426574561
H	7.16899898586553	-2.48572443700364	0.54835200526288
H	5.73627810430643	-1.72926446529295	-0.21478000339188
H	6.28172041202969	-3.34601642106415	-0.75447642348489
H	15.45322902018461	5.52674944267346	0.47067887027405
H	14.23185630426422	4.32400730256566	-0.06778630942011
H	15.97317794277076	4.00926409667326	-0.31602007774232
H	15.15764409392731	1.64001959051943	2.46998760832922
H	15.77169858942556	1.79999598040947	0.79662036367177
H	14.02932495524072	2.06046612636151	1.14469500376317
H	7.71218776257385	3.03562971170013	-4.20378058026783
H	7.16742645484329	2.27933803006040	-2.67885372990776
H	6.23485947916674	3.63585208020669	-3.39857266951399
H	8.65225687468721	5.33065823277053	-4.28901344418387
H	7.23035622189546	6.01060280655796	-3.43146850887514
H	8.88212319636312	6.30809249770154	-2.80776203977033
H	10.23789650699229	4.34132365298538	-1.76411578120896

H	9.97773749535596	3.39993804292072	-3.26350342048909
H	10.14959862437913	-3.73392976219434	6.14212039587328
C	12.84673775483273	-3.83111085698289	5.24858093897097
H	12.84004388092075	0.26809873361255	7.64836751325482
H	9.50287537466506	2.71092729007784	-1.67458148905252
H	1.57707617062249	8.16433437561718	3.52133832473073
H	1.68604343700695	6.39148031155756	3.71421507094496
H	0.77388435147222	11.93572266952228	4.57202529977853
H	0.50004408656589	10.18635353101236	4.35018692770781
H	2.09146947148263	10.89074344547348	3.95233406855328
H	4.65681155131279	11.73297391156579	0.35959298479100
H	4.04168136100240	12.10855429267422	1.99604463641912
H	4.98844388101890	10.62245748288023	1.72302622504620
H	2.71918700663530	7.89039571979310	10.06594961620155
H	2.00471229525319	7.06870344726112	8.65121833269049
H	1.36026892188266	8.64891748977556	9.19454702955033
H	0.81853917625416	10.69261499635202	8.15761684449875
H	-0.24061838958956	9.98150835558550	6.90713848596031
H	-0.01731839871725	11.75074710577599	6.98435429046105
H	8.30762410464981	12.40636664893984	0.97260655246143
H	7.08480331383271	11.81949561020381	-0.18966803791977
H	7.57168470907681	10.78532152356699	1.17883978120262
H	3.17289931468796	7.37704102526239	3.72608600832987
H	-4.14021387673039	0.70410921056332	3.39995549749837
H	-3.65992264113047	0.21857362254686	5.79763261630012
H	-2.41406371024632	1.11181056483974	6.71341525160518
H	-0.52354931923136	-1.80305676800360	9.84845120751674
H	1.78461391958185	-1.79916173880370	8.94133228874444
H	-1.09529273983875	-0.45485632419379	8.83127449893011
H	-2.96224811784124	4.25883443969330	1.88856893585359
H	-4.59588355542799	2.38681208651967	3.77429399022842
H	1.34008309029406	-0.50832948829583	7.78495080859854
H	-3.99172590684939	1.88044332124653	6.35715614049000
H	-3.35885526762155	2.05460762058134	2.53020922094802
H	-1.25633787862642	-0.36535246058803	10.61480791601184
H	-1.59107041680745	5.19798917647613	1.24413993477333
H	-2.42582597566959	5.74829620146365	2.72263033386896
H	-2.23946257242946	1.46238574308717	9.70878919644541
H	-1.21294991566270	2.68842942205759	7.67553142975772
H	3.38122311530917	0.50580930376365	11.10705017177350
H	-4.29309663481847	4.90505066043539	3.89949145788203
H	-2.20533868385531	3.22888797301427	9.43451449994648
H	-4.81331912056323	4.08402093326675	5.39967623113642
H	-2.84600233514649	2.58348970677130	10.96114294197018
H	-4.72619591111685	5.86355255689270	5.34440211389244
H	-1.73299327000128	5.07388332348618	7.46836657833913
H	-3.25047179564953	4.12296965626723	7.52947528140203
H	-0.43091599474019	4.73279385880953	10.57300431985185
H	-3.31977807981636	5.89723732416761	7.38483693155271
H	-1.33303736536092	4.26076207866023	12.03858661610472
H	0.44375546492303	4.09600023626443	11.99981471269983
H	2.05655970266930	4.64904523950604	10.43110716332647
H	2.76568910948730	-0.30768876452065	8.83231534884575
H	1.28418610561573	3.49198548839129	4.22014248843325
H	3.08236135057784	2.17986494290639	7.89653296186080

H	4.12755528780587	1.44413387270262	9.77809169642087
H	4.43262421909365	1.90095776719881	11.46489008695205
H	3.29089273873965	3.95138063820783	9.35569332630599
H	3.68017905511448	4.26554906516136	11.07557431185318
H	-1.27666149937588	2.15768693652264	1.84485981383138
H	0.28904116570756	2.28441129150655	2.69380293145147
H	-0.01929619819836	3.26933161360711	1.24018293024799
H	-0.63458703878058	1.86108078955248	11.71364059285546
H	1.22821154247242	-0.30724390484030	10.87556214905012
H	2.05797985051083	2.52309871726979	11.81397736326556
H	4.18870411335591	14.36285947086156	2.66374435881423
H	3.68940204053578	15.15676066617860	4.18448350237042
H	3.45215968222863	13.40248385001823	3.98045902445978
H	3.56336563708546	3.39879309915877	4.34123907862292
H	6.16265824520991	14.89290940310751	4.09611159867472
H	8.58486077132705	13.77375575313798	2.93200408687851
H	6.09650060739831	13.50341251698958	1.41110904025609
H	2.40810278044181	11.50276576415930	6.40588667702001
H	2.57070269454581	6.67096964986268	6.05083823765524
H	-0.16038559712165	8.02735120019362	5.51002674380069
H	0.35059099055167	7.39139240329137	7.10137010750631
H	0.15676664001797	6.28099467908590	5.71819711823888
H	4.17708629178120	8.09908083118980	8.01896804072461
H	-1.83474494788163	1.07197735186472	4.28383919203302
H	-2.24548570475946	5.79723041538406	5.12917725709688
H	-0.23307257684114	4.70432747842976	3.27464445904743
H	8.18540348308794	11.30133079056998	9.36668710687590
H	6.83884659543273	10.25357193701307	9.88839705032937
H	8.01741576085170	10.85198464992767	11.08723629623727
H	8.83130523249311	7.11403793315300	10.54137171796311
H	8.46811680353183	8.30675298321914	11.81930067737675
H	7.21311762859975	7.88581950913638	10.62504034614010
H	12.92835695321198	10.29364444209927	6.76068817773093
H	14.86124080488792	8.93767490578707	15.01157695948393
H	10.99667658964480	11.90881627360481	6.76233133921666
H	13.05392441440508	8.98810971255940	7.98046649661288
H	14.30178730826962	9.22461263935032	13.34704671955252
H	13.33597546490587	10.68799064630566	8.45280357750997
H	11.20612827764593	12.37143358272321	8.47276903654691
H	9.63430643777712	11.73799645847059	7.90284337314274
H	13.02749358020158	7.29011247482315	15.48641586413415
H	12.91916078914370	8.39887563283650	11.69784699688525
H	12.35038561208767	7.45538840359022	13.84070396213558
H	12.77739586938428	5.84429977041994	14.46252283560079
H	11.26899657704548	9.46312890464318	12.98718713737848
H	9.61127365111742	10.06036694085182	12.71763349229610
H	14.98844039367574	2.78238550111049	14.18509053846513
H	10.84798725459814	11.14318138570674	13.42030281899454
H	15.55720267648576	9.03599062738900	11.32160747162765
H	16.92950433034991	8.76583011545920	10.21001267907062
H	15.99458482641064	8.72858291038950	13.64622862027214
H	15.29873941661526	8.16593492950536	9.78603056158668
H	17.90290117844051	6.52631703948894	9.82890129375119
H	10.42197857899244	7.02622554629273	8.43276777872068
H	16.34445230493909	5.73677442507939	9.46641964049397

H	13.37844202458616	4.38717706666310	10.10941439065061
H	17.40086456106748	5.08747225531263	10.76034086355643
H	15.65369426193345	3.62962309298298	10.59056786337980
H	14.18786741833352	2.95444170556871	11.36067027112663
H	13.48694512257510	3.64066014407437	13.75401948179582
H	15.79254510353407	2.37409950006811	11.85459754650772
H	14.69666932360971	4.42558654075562	14.81704471823493
H	11.85874940485591	3.53489087645214	8.57248621921623
H	8.59084360380771	8.87157273438613	8.77280846199960
H	10.66413180369626	9.49682827607492	7.43474204928857
H	15.21796019973622	6.53068814704829	14.55895819031175
H	17.05639802212458	7.15004153343586	12.13205634736169
H	16.35315557446584	4.48364801914353	12.94512979888121
H	9.23763357025695	-4.57101008330282	13.64915085121967
H	8.57242374825574	-3.16355592842609	14.52941551602018
H	6.88040942243910	-3.72763662605778	16.10905253019760
H	6.78277368833090	-4.91880738379479	14.04721879426766
H	5.97696301478663	-2.66829361932286	17.22849224105702
H	7.20108200166070	-1.96843015036828	16.13895958970727
H	6.14803647352650	2.38122783010371	16.64435785456132
H	5.97986346841353	2.11673578398683	14.88002123000451
H	8.06020527563718	1.01112181597250	15.86994503678914
H	4.54965106555148	-3.32505996939304	15.29821155040205
H	8.47698538689698	2.67622291746193	17.69418024878565
H	6.55525274249800	3.68952324523555	15.50236324134361
H	7.17000384488583	-0.37038607218368	14.40803250066166
H	4.11791188747711	-1.17668653528418	16.44298843248432
H	5.26083391195090	-0.31172869221298	15.38324035900802
H	10.41355927045091	0.21212136892322	15.68624236811413
H	9.95913702798611	2.39884128014741	16.73367127120740
H	11.18186648771107	2.36099157029422	14.62966449415421
H	4.15966499115787	-4.48888598940718	12.72682619168647
H	9.00560378372365	3.89058573570628	16.49863663118944
H	3.75279442373246	-0.96832407722622	14.70504791872801
H	2.71277432941388	-2.87811908191077	13.97861555296635
H	10.34272637910477	-0.57453031342647	14.08164840994558
H	6.66659249329705	4.45459136131836	13.36890871899729
H	2.82089905543298	-1.69904243730466	12.63359588604195
H	6.81191992830513	3.43170575053093	11.90847934471943
H	11.41196862349847	2.61334863642032	12.14464875101012
H	8.89186248957223	5.23470299563077	14.61242224383106
H	1.99483532059720	-3.26055608620794	12.38975070661175
H	10.47767329292475	4.70094598960838	13.98048019653993
H	10.94858626935436	0.90979524415395	11.89532615988651
H	7.34773712972568	5.14538647588205	11.87330109126932
H	9.49894550087726	5.87448565471220	13.05828849960320
H	8.90292236160634	-3.04171150871718	12.78216414961359
H	7.89423355194449	-5.83671814931953	11.97775330730199
H	7.41210323551761	-4.38717910108879	11.05845829105562
H	6.16242577472575	-5.50441021765598	11.69300539331200
H	6.25416021352471	-1.84449819141308	10.43249552178366
H	5.11126187634346	-3.86753157740286	10.42455738865839
H	4.11579070314602	-2.38035526485599	10.46227771069855
H	3.32915363177856	-3.97608371824157	10.45796900413245
H	11.91670386158541	-0.06253683913653	14.75845813774279

H	12.48013260173831	1.30290073532617	12.72718463236766
H	8.59792816542516	1.73336062981876	10.89822467098195
H	6.71782793867590	-0.56678024547958	8.56403608055344
H	12.67071550512262	12.13224412366886	12.04110351698474
H	13.28014545394667	10.57276160969906	11.42154193264369
H	12.78836360073276	11.85046646878362	10.27997221034501
H	10.41837472893254	11.68590811599523	11.00126177218930
H	9.31768864897393	3.64389529864203	11.99387913299658
C	11.90522149321759	-4.98081618559285	4.84379687690469
C	13.38844608523391	-3.16830612311983	3.95908433772446
C	14.02370257333964	-4.42593794670387	6.05958866054313
H	13.91029604875449	-3.91968593949060	3.33099927450278
H	12.56485654930049	-2.73165732884581	3.35834198689771
H	14.11126243424335	-2.35770574875247	4.17944422713900
H	12.45868444655248	-5.71987802783620	4.23016112915457
H	11.50302524508952	-5.51828629167936	5.72610107783457
H	11.04858744422359	-4.62168021036973	4.23815074895092
H	14.55801412660646	-5.19040824396042	5.45797909210641
H	14.76142345199117	-3.65132685388326	6.34965070197904
H	13.66198724928718	-4.91197876194398	6.98837331191168

Spectra

S1: ^{1}H NMR (400 MHz, $CDCl_3$):

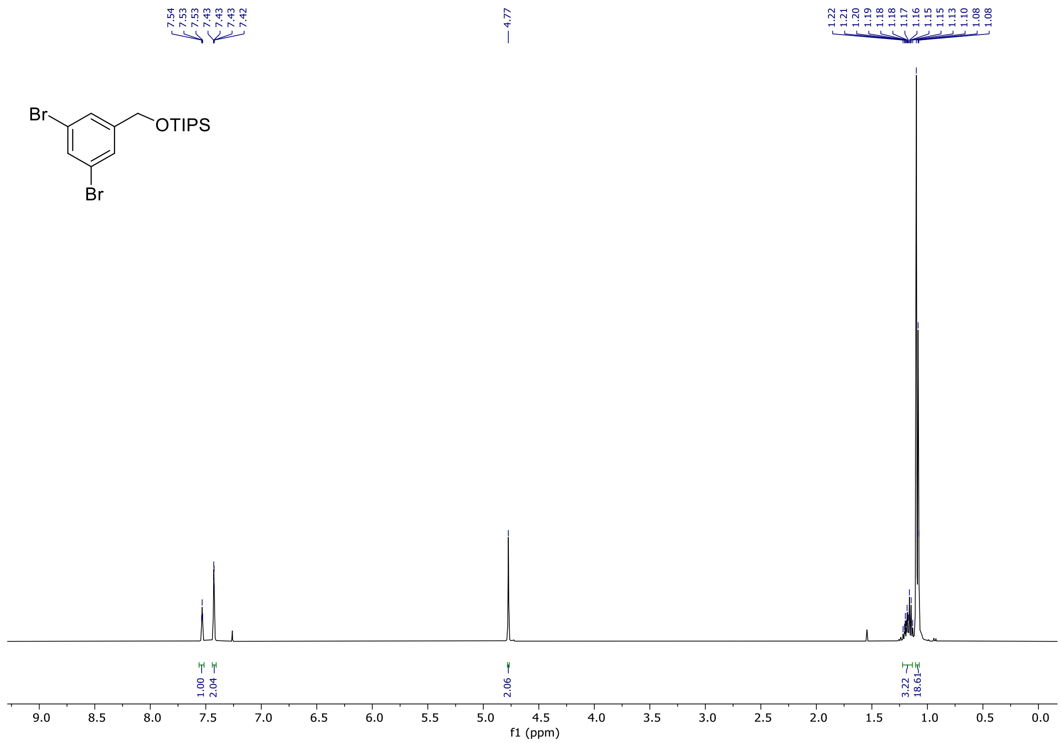

S1: ^{13}C NMR (101 MHz, $CDCl_3$):

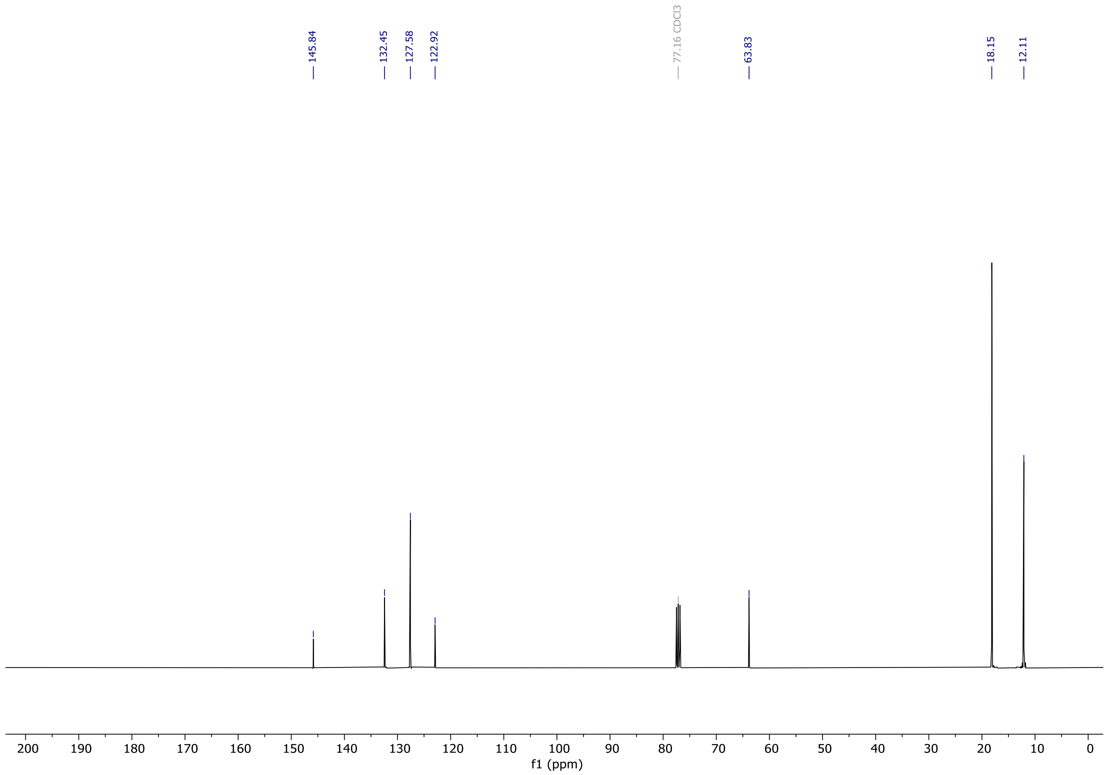

7: ^1H NMR (400 MHz, $CDCl_3$):

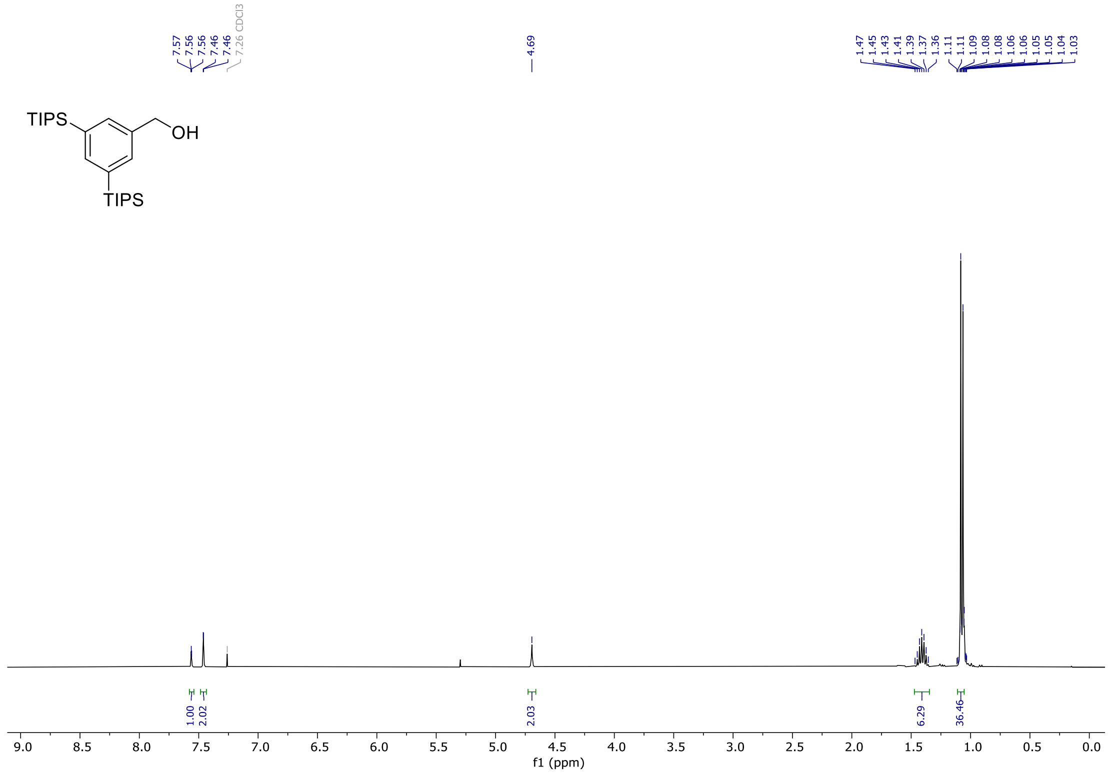

7: ^{13}C NMR (101 MHz, $CDCl_3$):

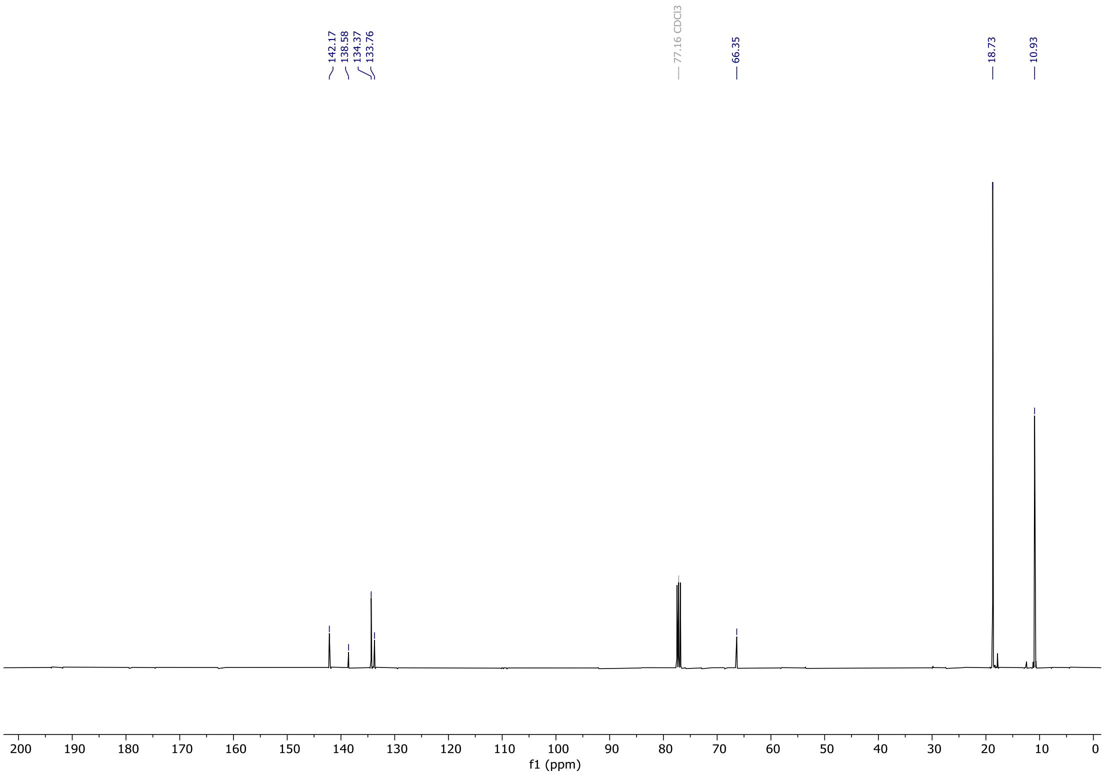

S2: ^{1}H NMR (400 MHz, $CDCl_3$):

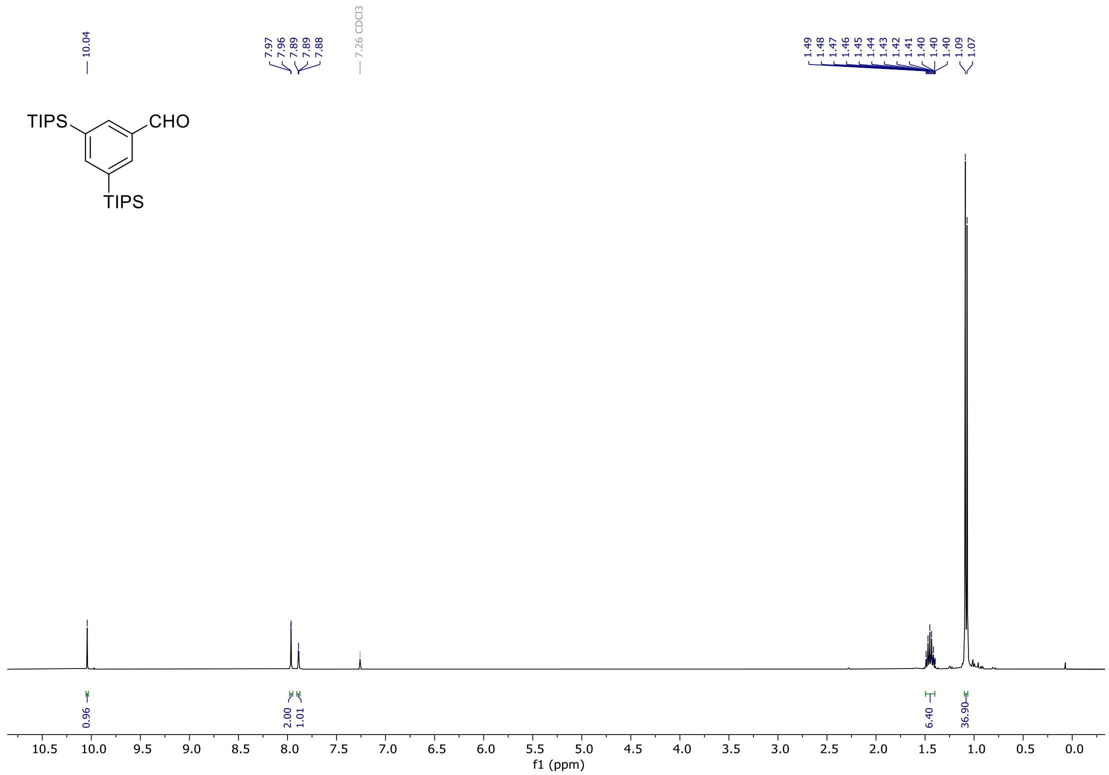

S2: ^{13}C NMR (101 MHz, $CDCl_3$):

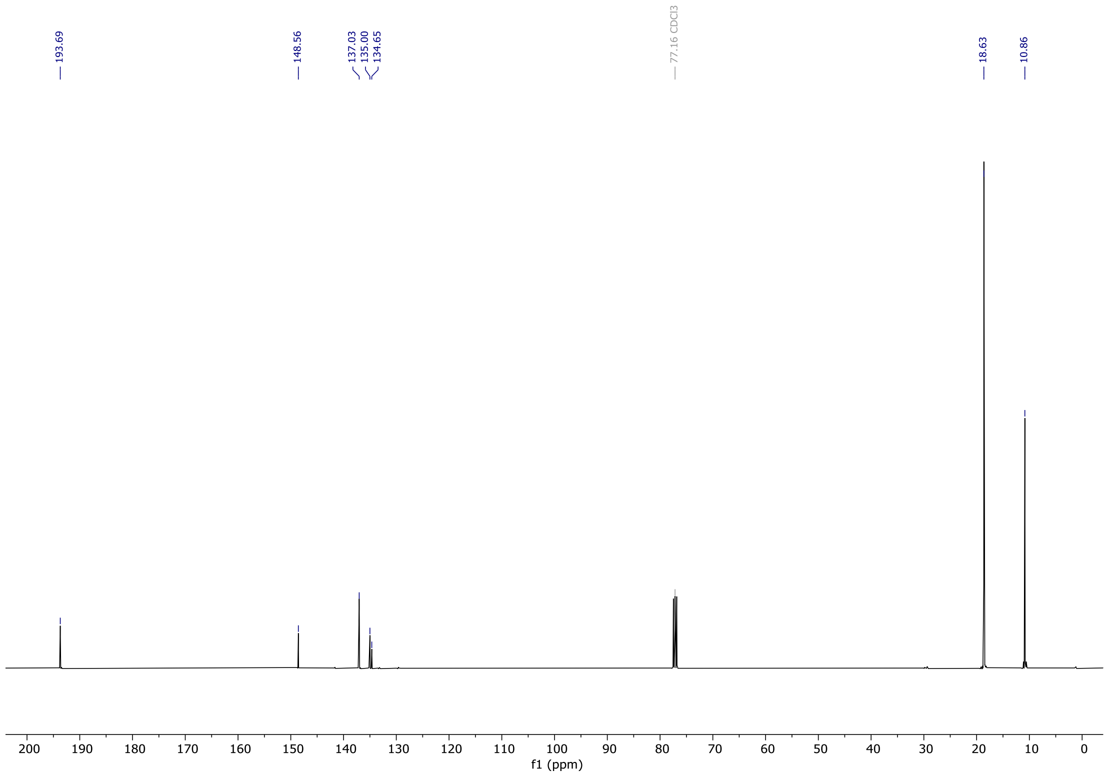

8: ^{1}H NMR (400 MHz, CDCl$_3$):

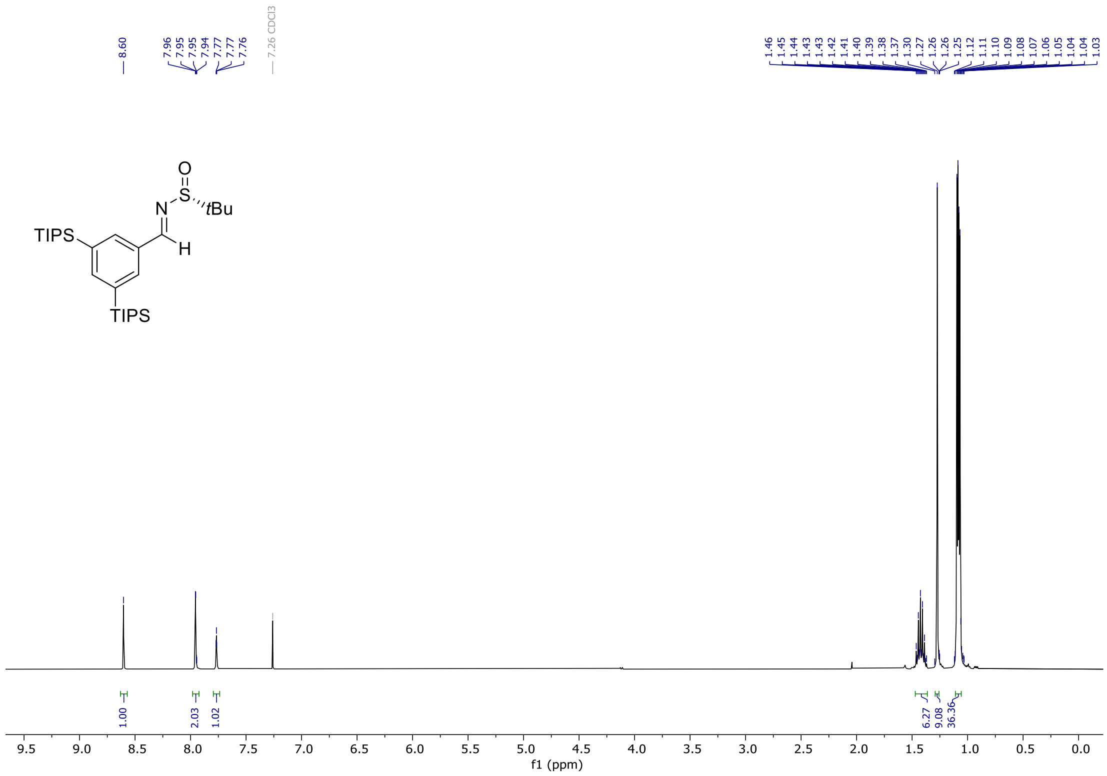

8: ^{13}C NMR (101 MHz, CDCl$_3$):

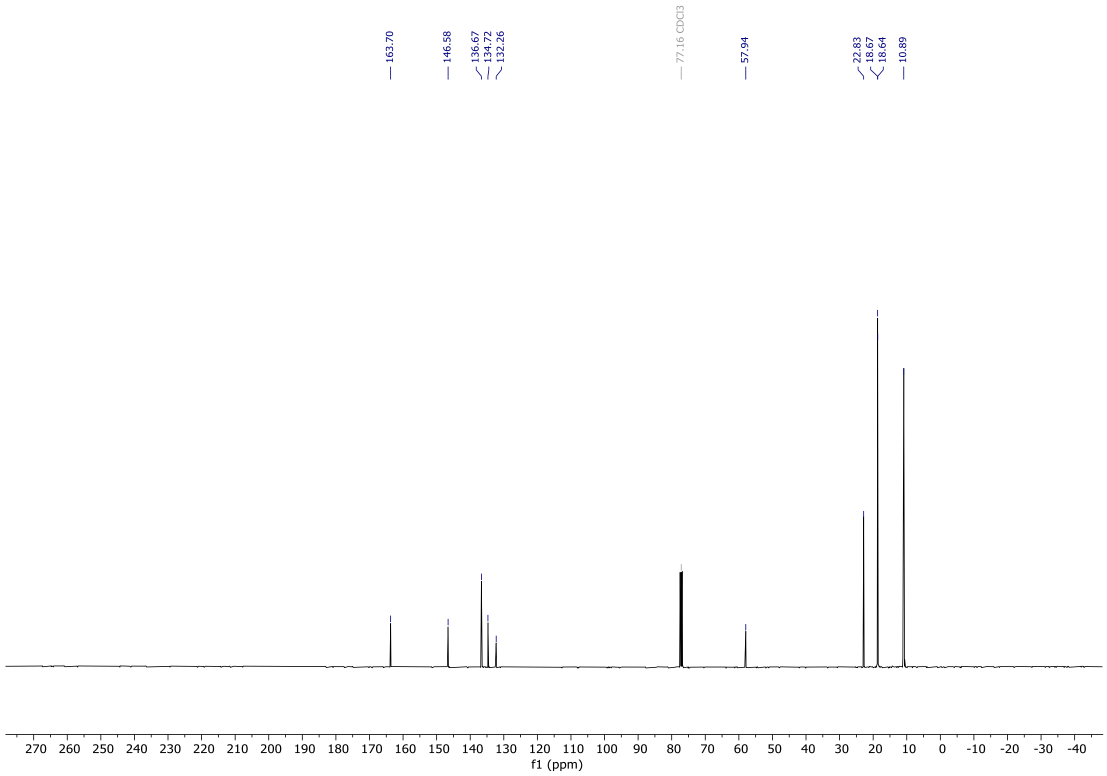

9: ^{1}H NMR (400 MHz, $CDCl_3$):

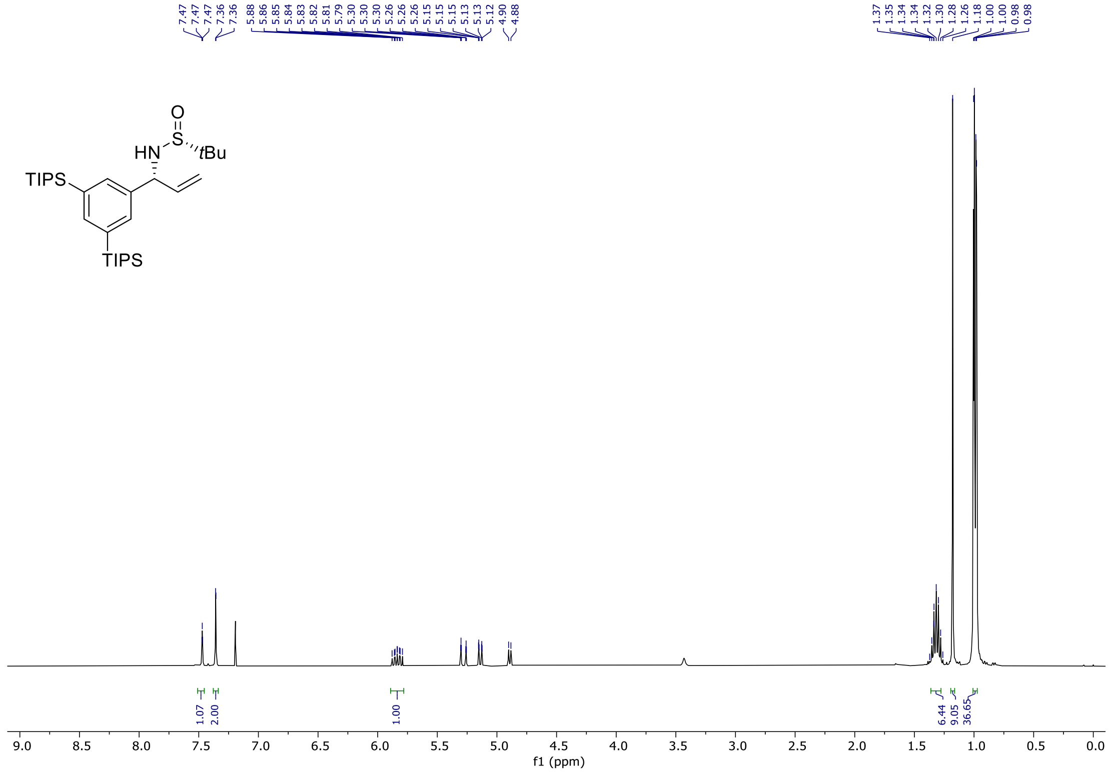

9: ^{13}C NMR (101 MHz, $CDCl_3$):

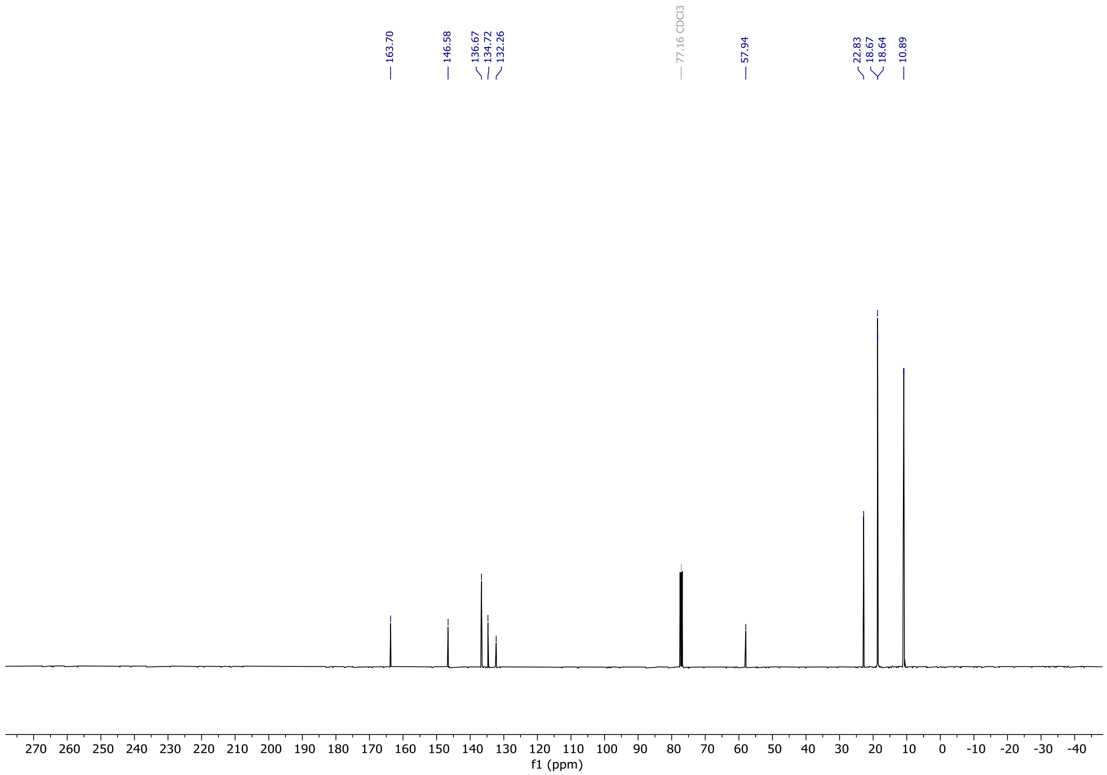

10a: ^{1}H NMR (400 MHz, $CDCl_3$):

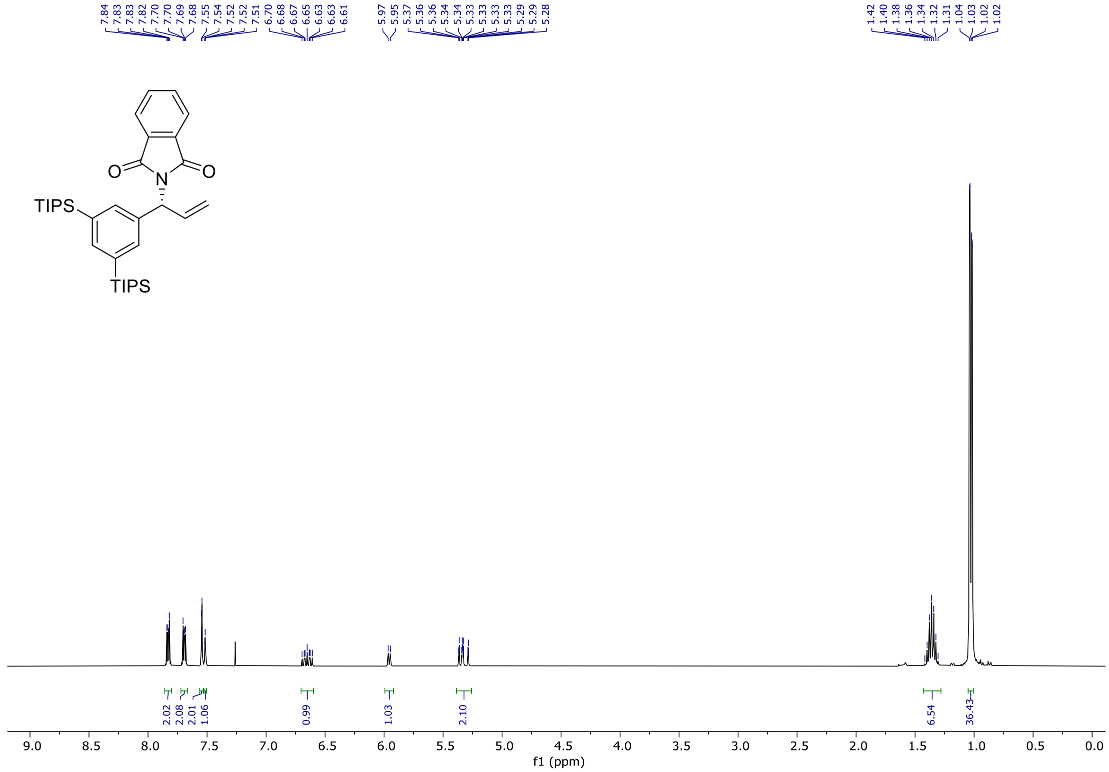

10a: ^{13}C NMR (101 MHz, $CDCl_3$):

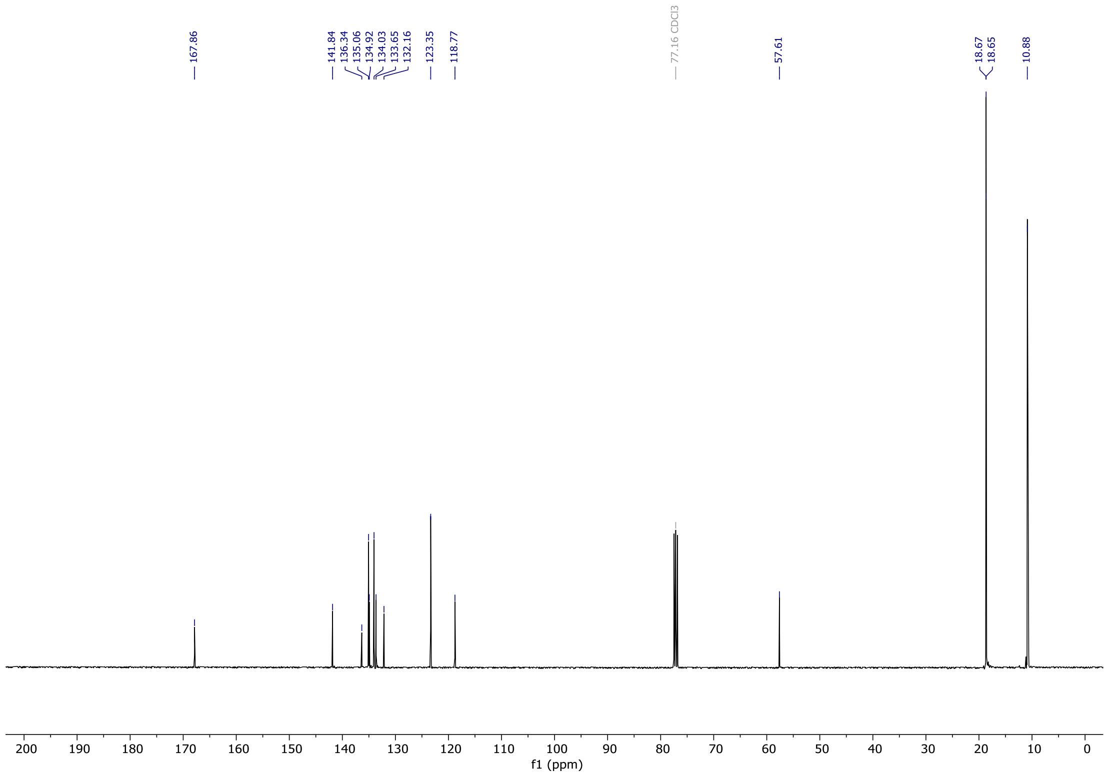

11a: ^{1}H NMR (400 MHz, $CDCl_3$):

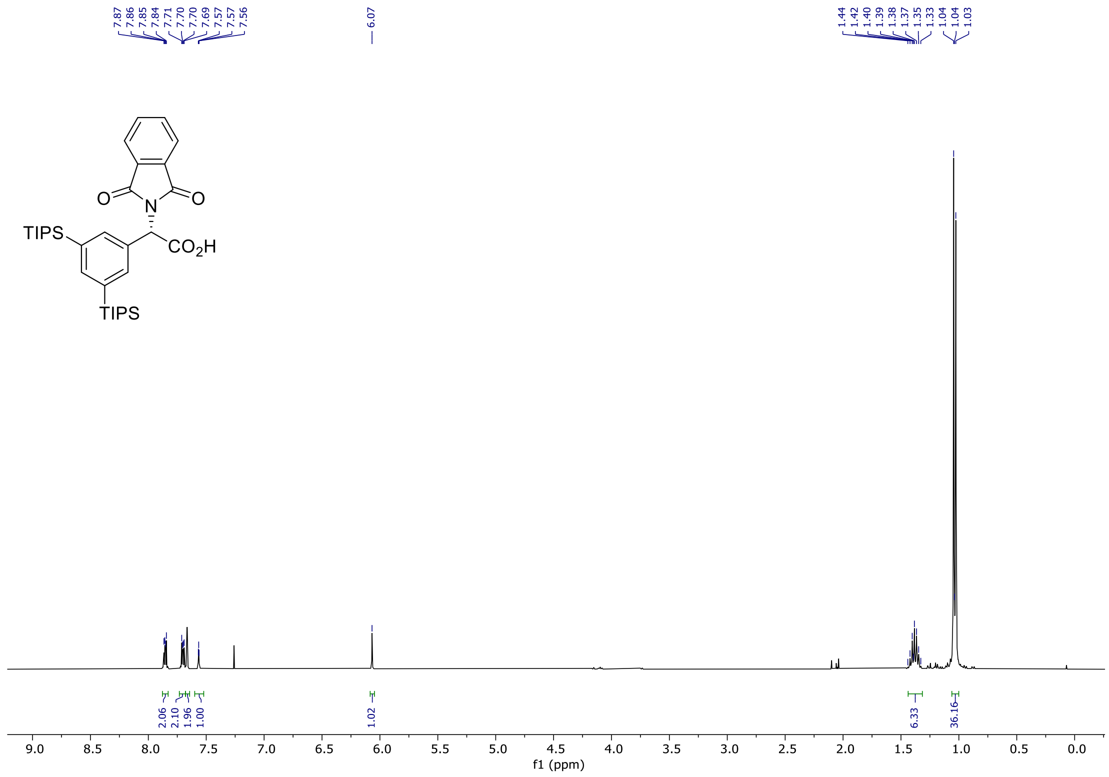

11a: ^{13}C NMR (101 MHz, $CDCl_3$):

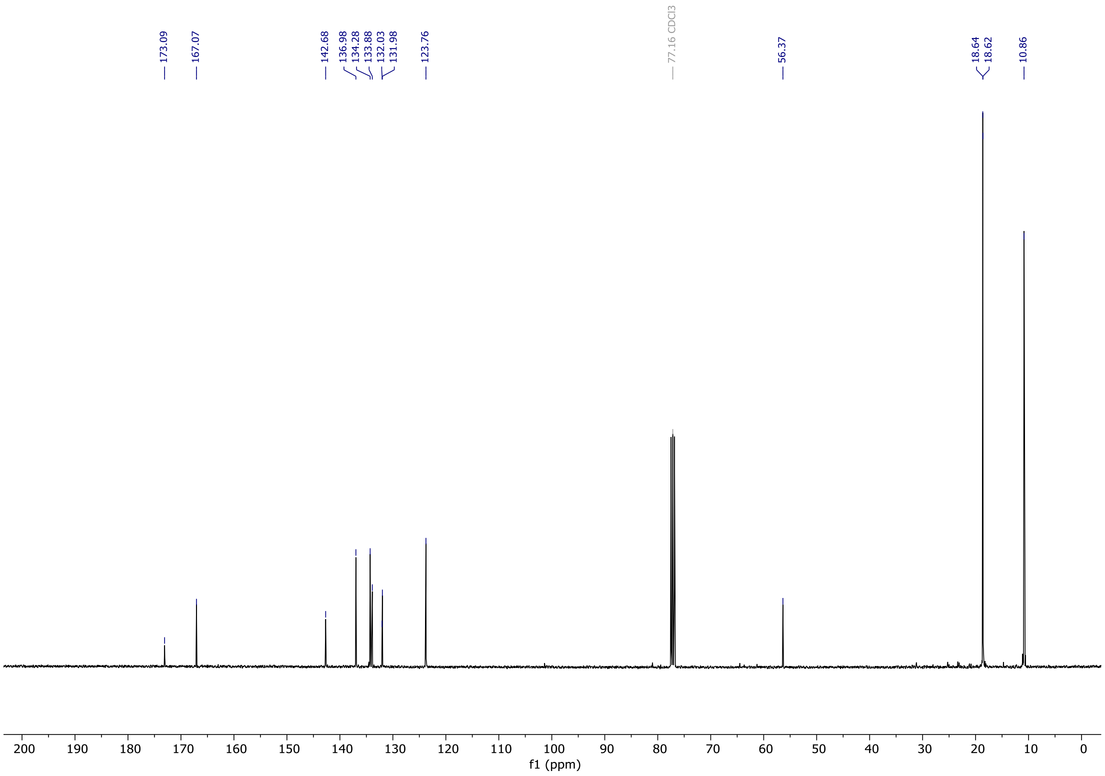

3a: ^{1}H NMR (400 MHz, $CDCl_3$):

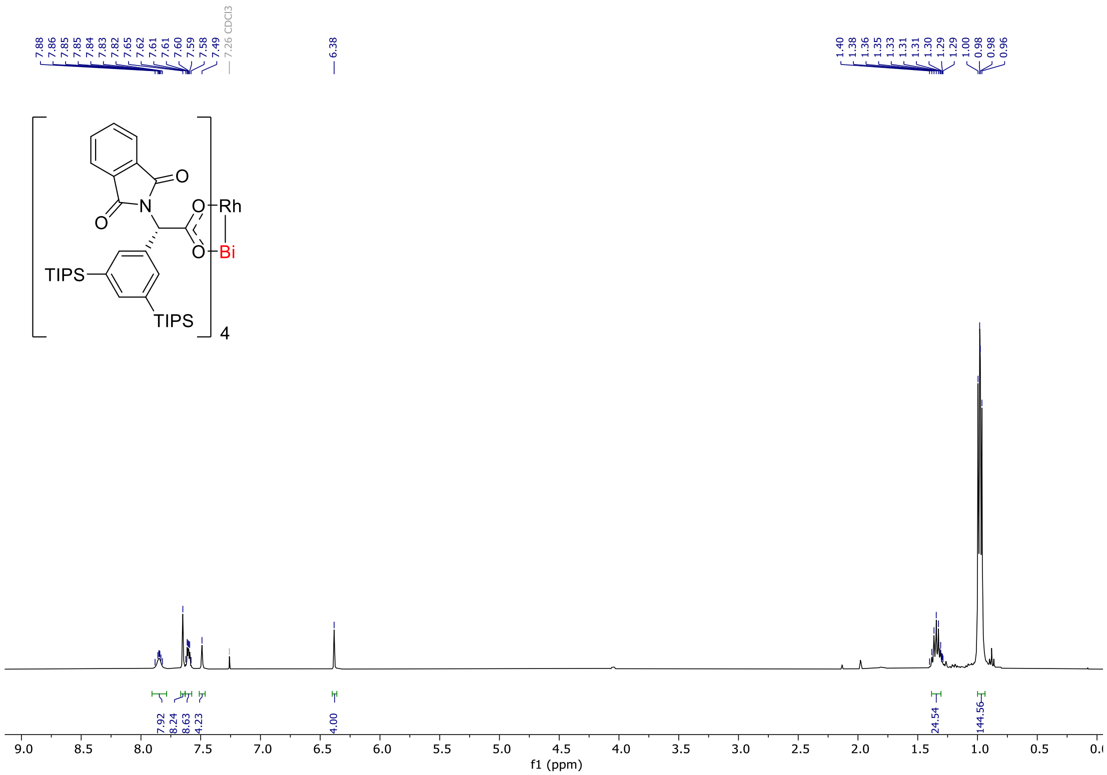

3a: ^{13}C NMR (101 MHz, $CDCl_3$):

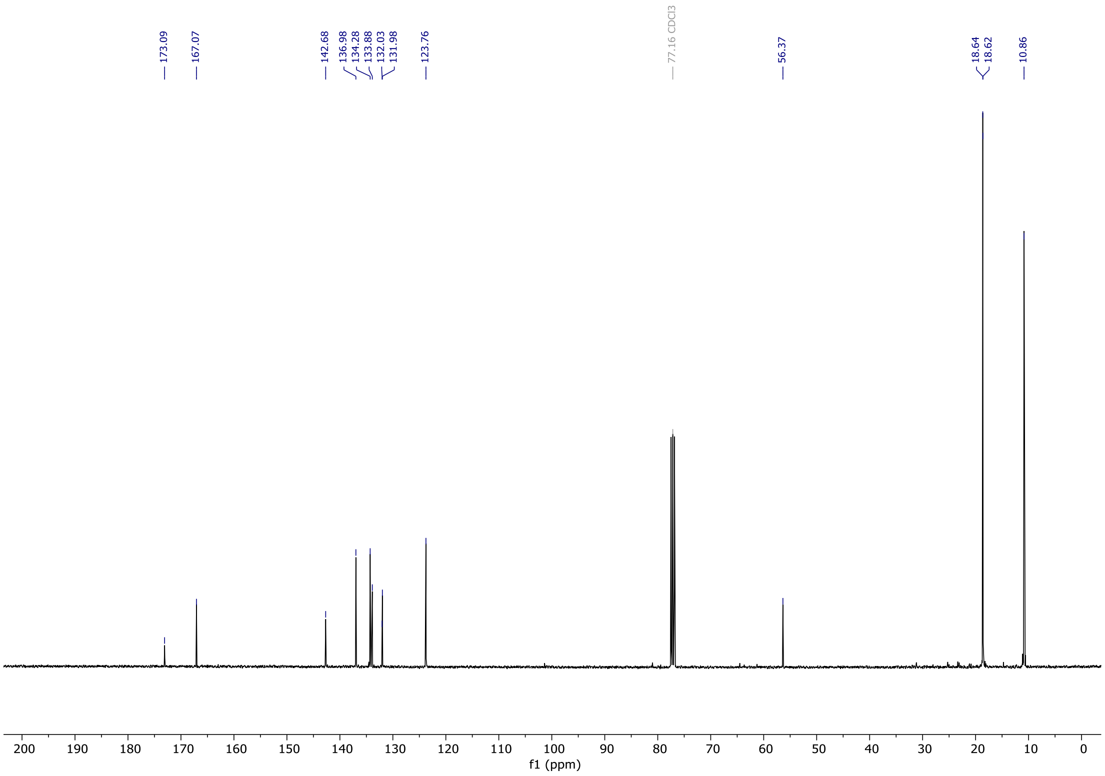

10b: ^{1}H NMR (400 MHz, $CDCl_3$):

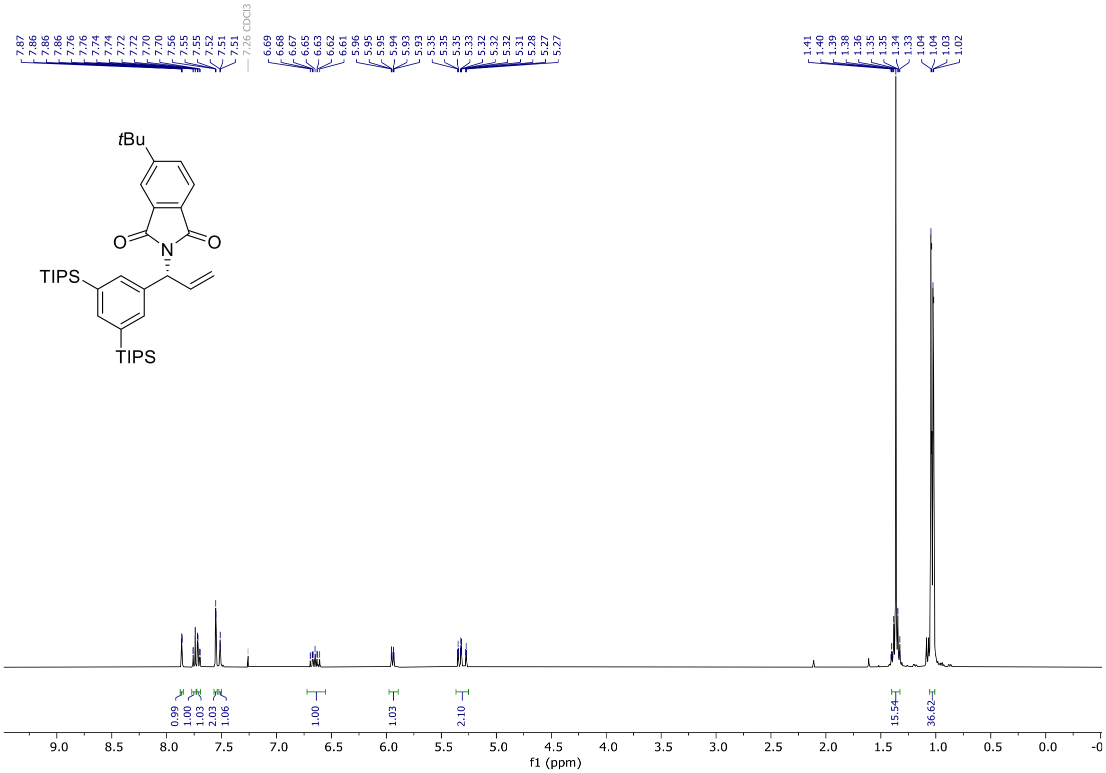

10b: ^{13}C NMR (101 MHz, $CDCl_3$):

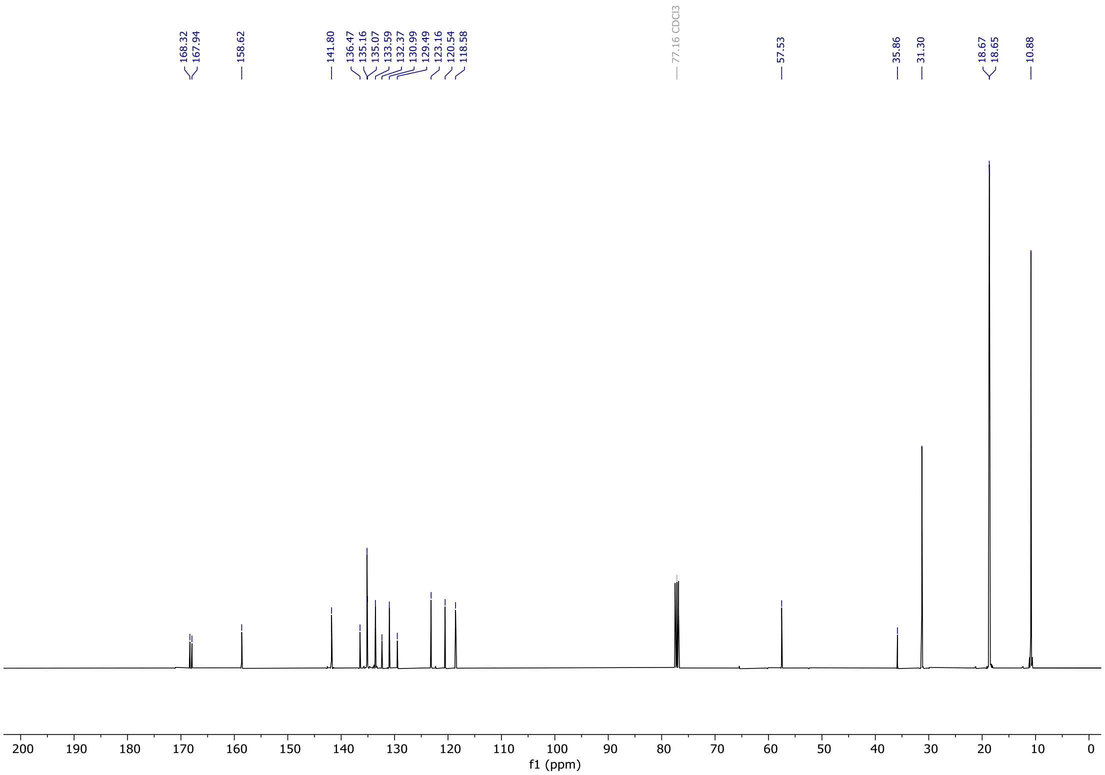

11b: ^{1}H NMR (400 MHz, $CDCl_3$):

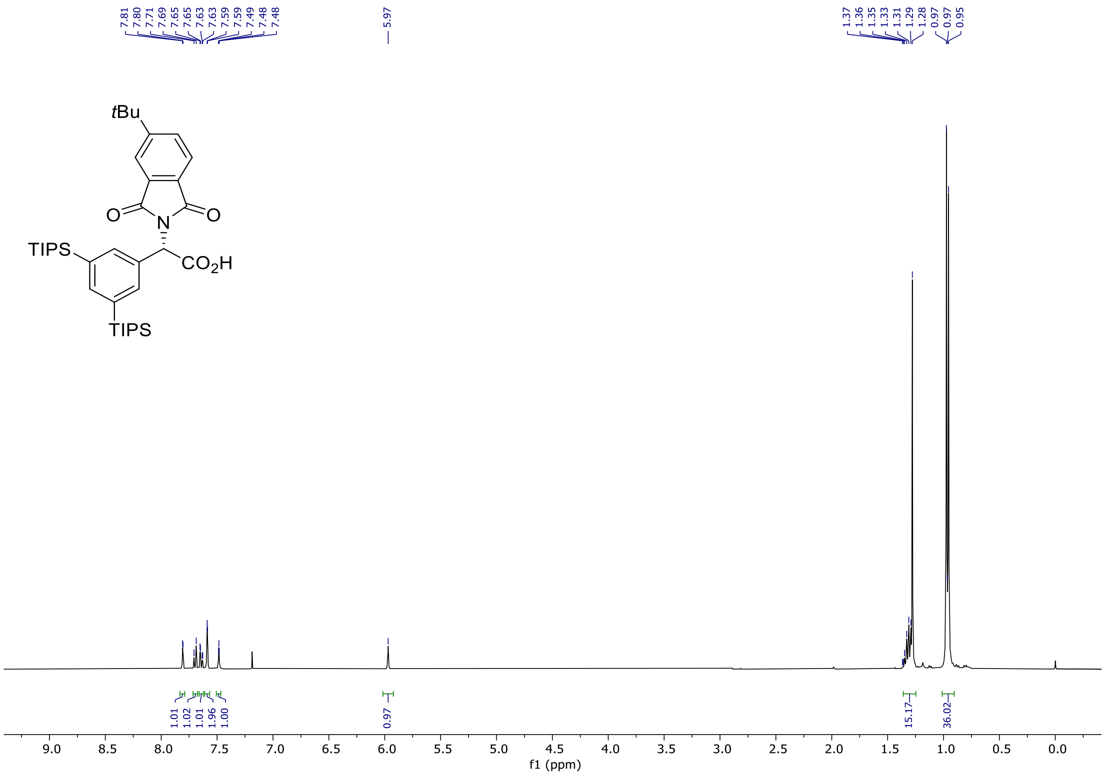

11b: ^{13}C NMR (101 MHz, $CDCl_3$):

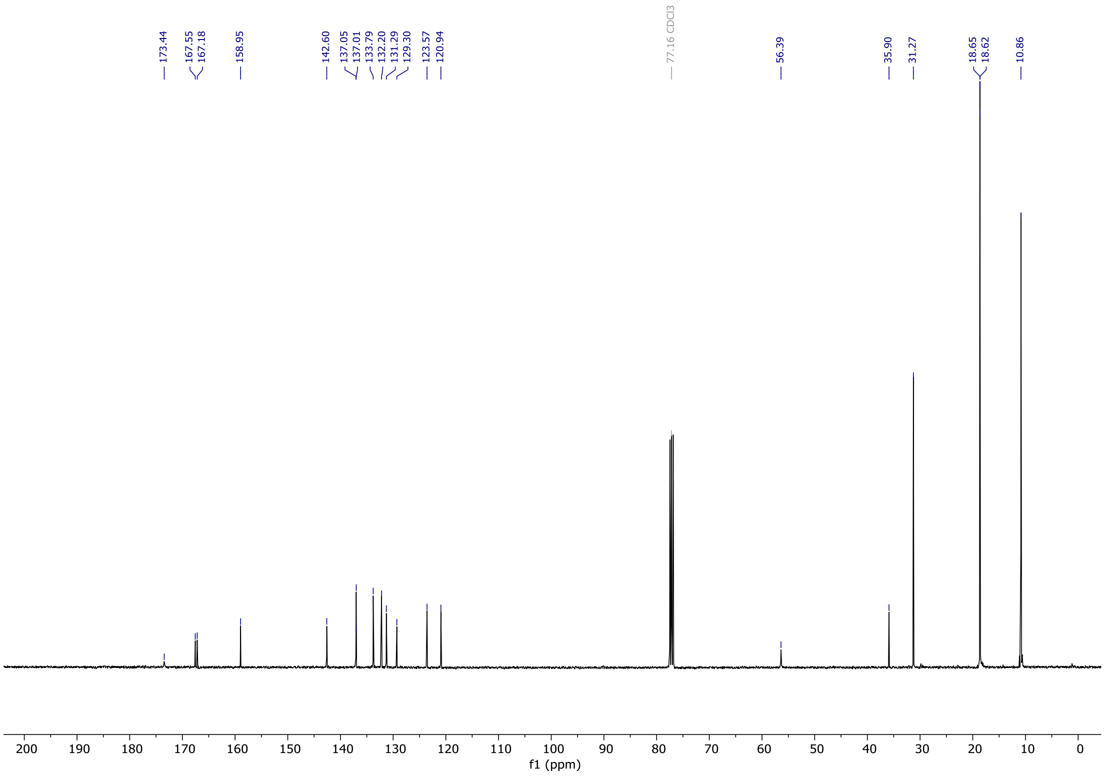

3b: ^{1}H NMR (600 MHz, $CDCl_3$, 353 K):

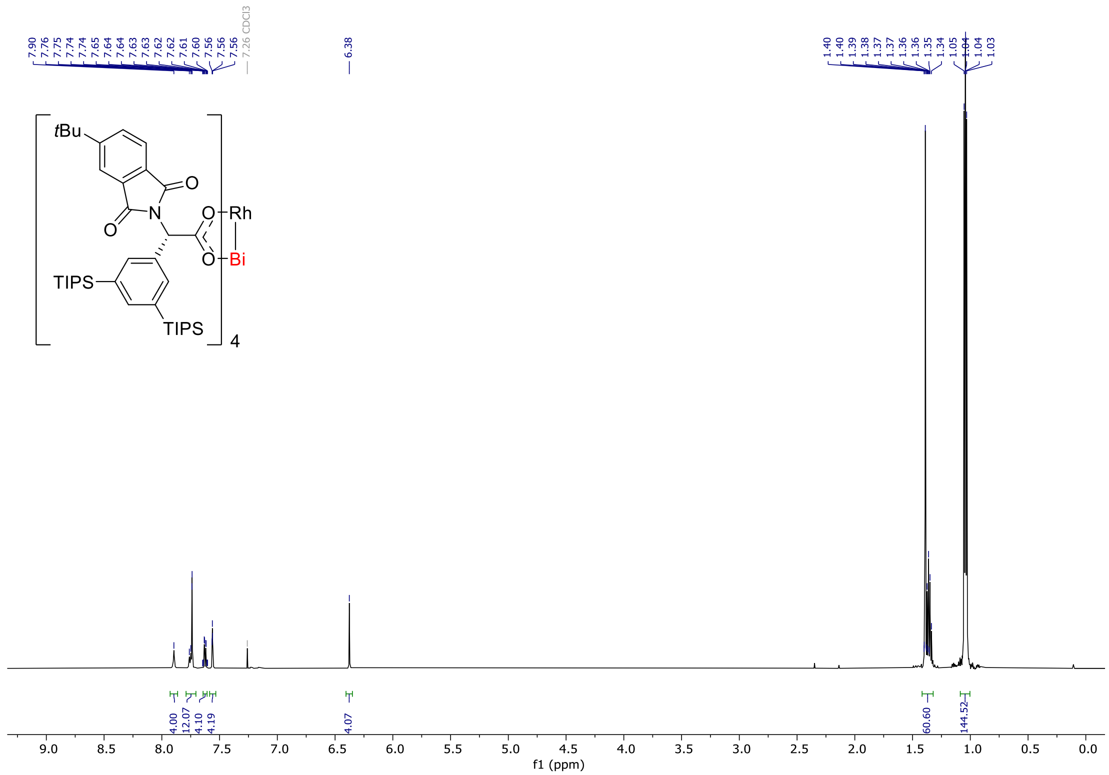

3b: ^{13}C NMR (151 MHz, $CDCl_3$, 353 K):

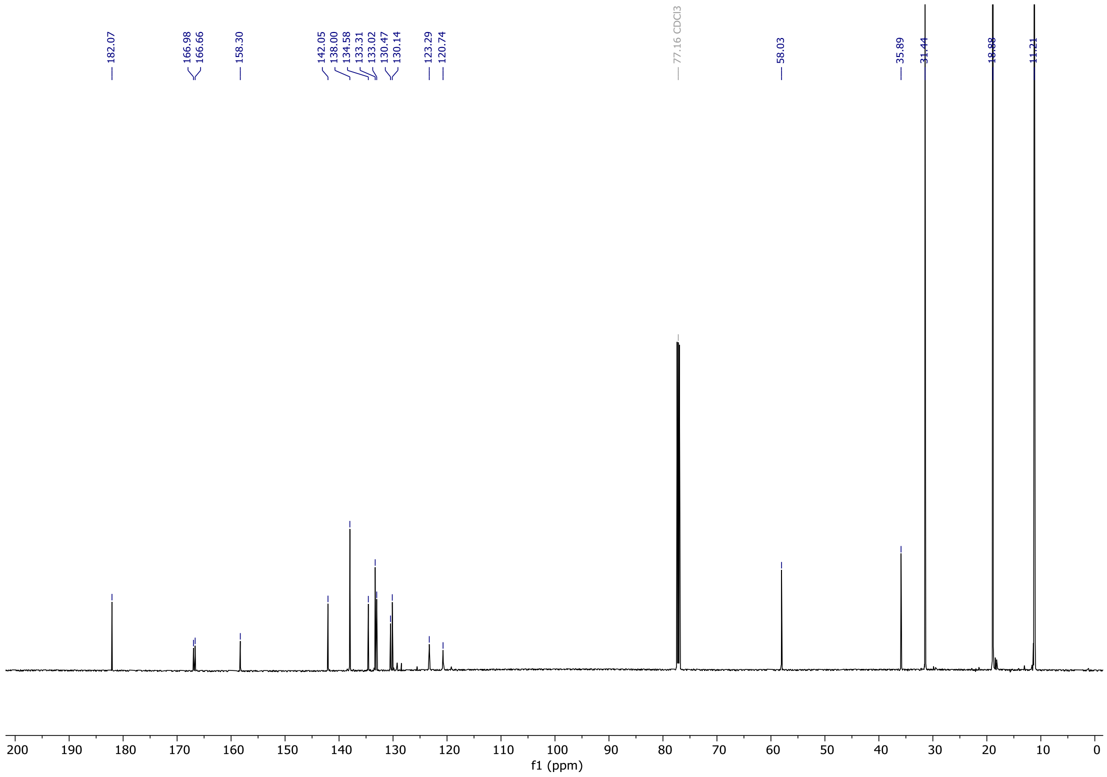

BiRh(*S*-PTPG)$_4$ (S3): ^{1}H NMR (400 MHz, $CDCl_3$):

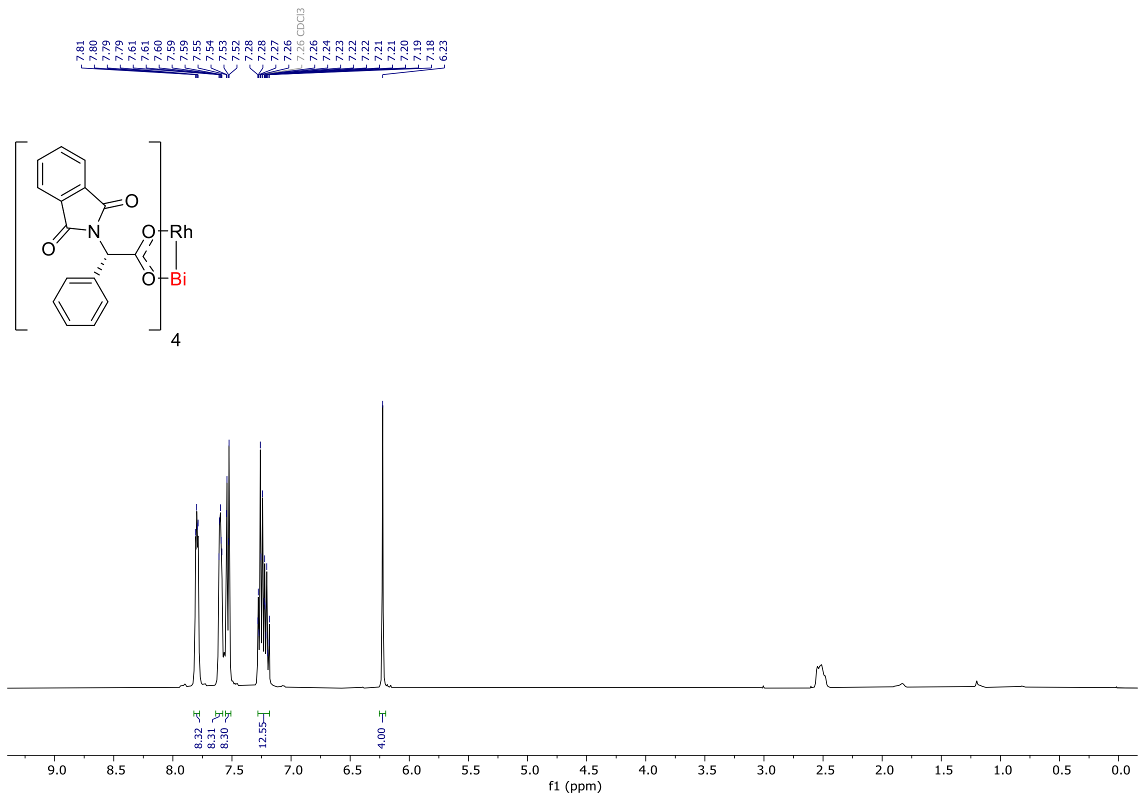

BiRh(*S*-PTPG)$_4$ (S3): ^{13}C NMR (101 MHz, $CDCl_3$):

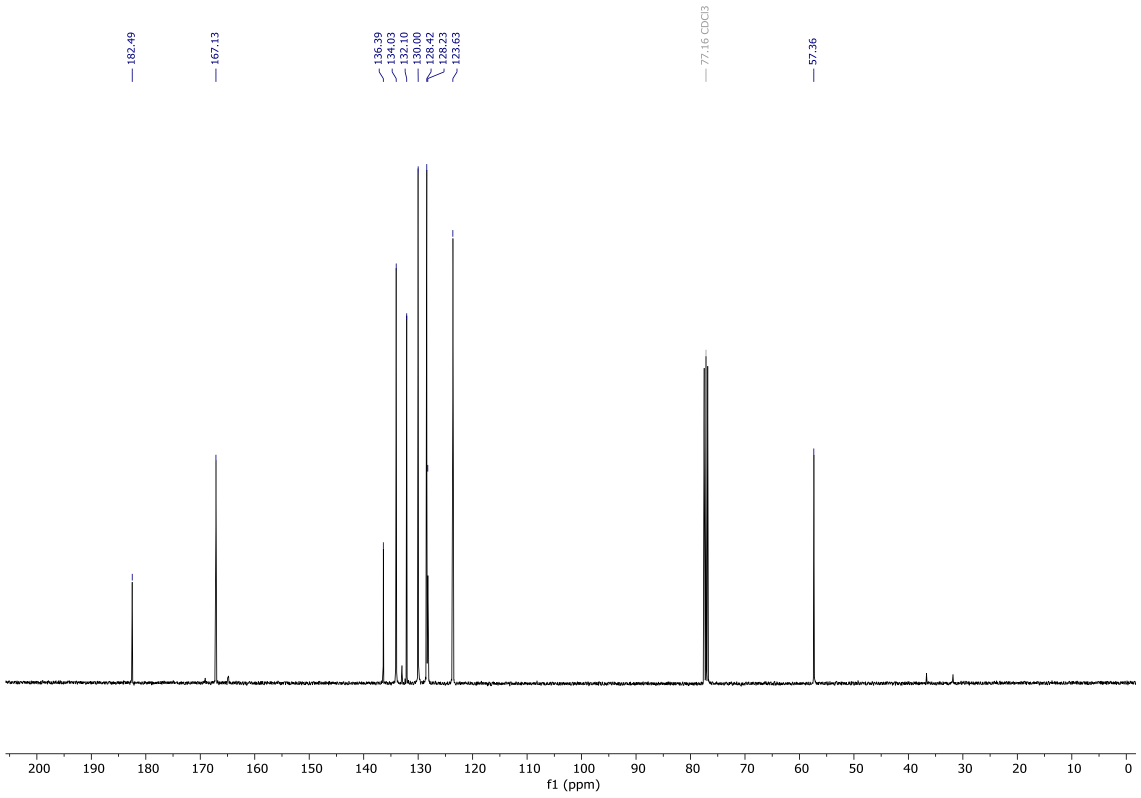

4e: ^{1}H NMR (400 MHz, $CDCl_3$):

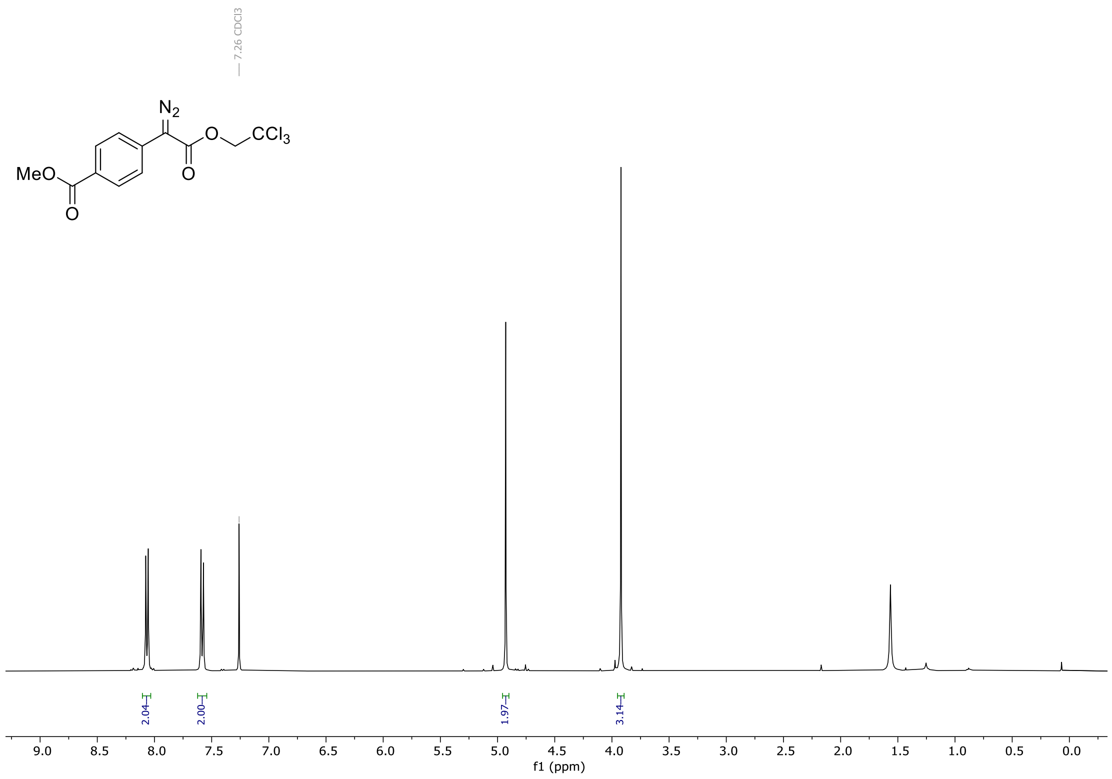

4e: ^{13}C NMR (101 MHz, $CDCl_3$):

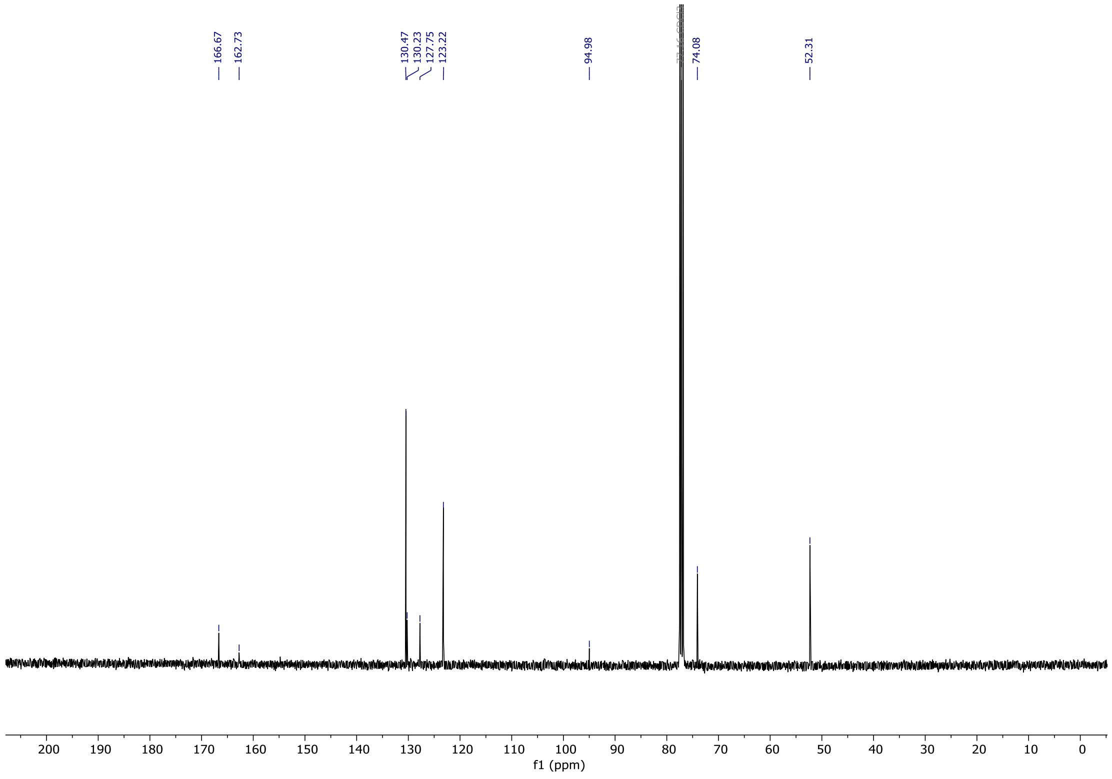

4f: ^{1}H NMR (400 MHz, $CDCl_3$):

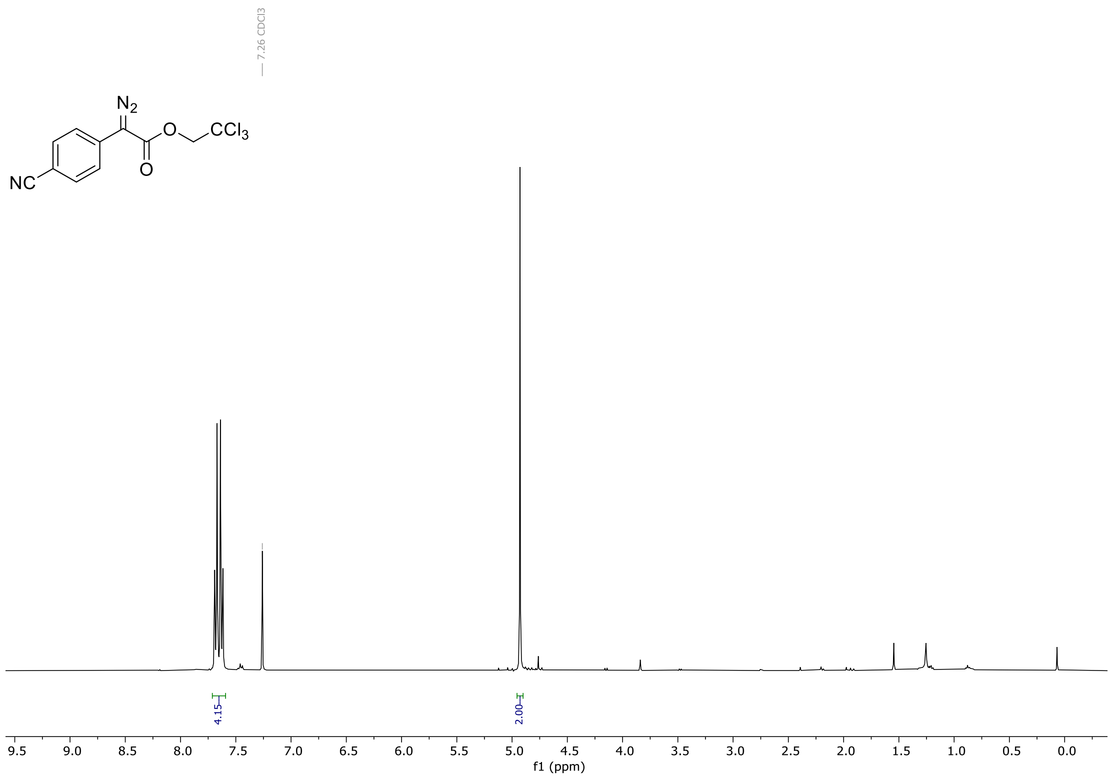

4f: ^{13}C NMR (101 MHz, $CDCl_3$):

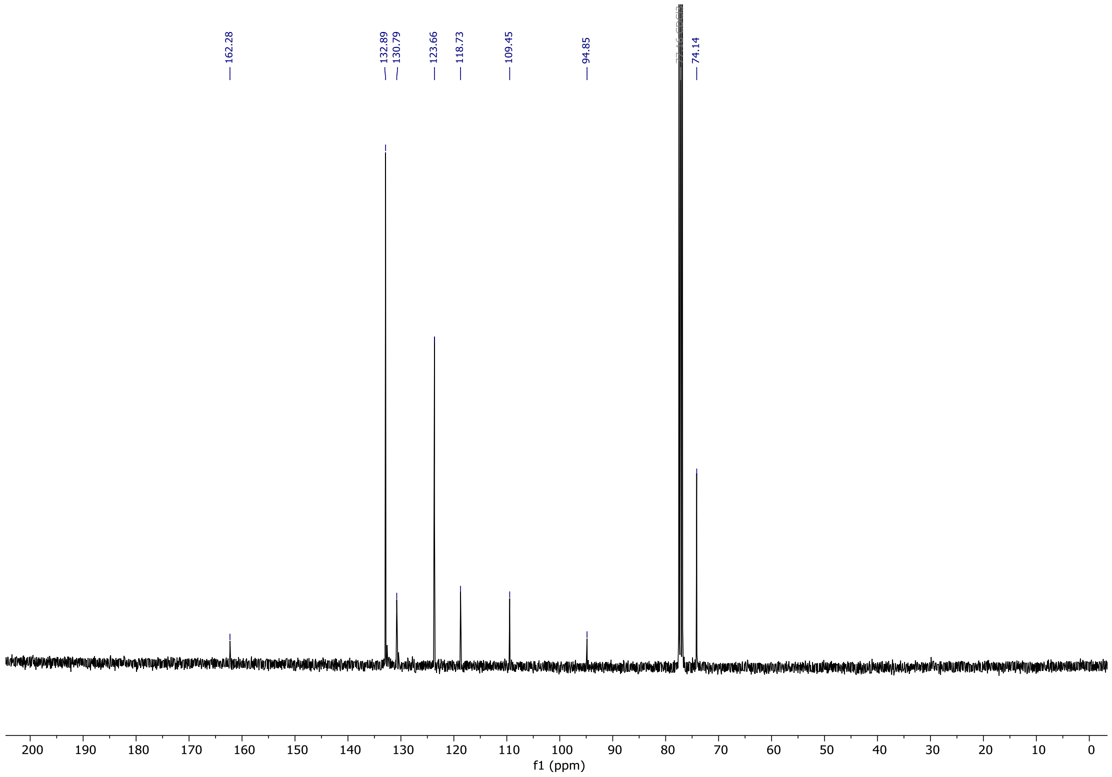

4h: ^{1}H NMR (400 MHz, $CDCl_3$):

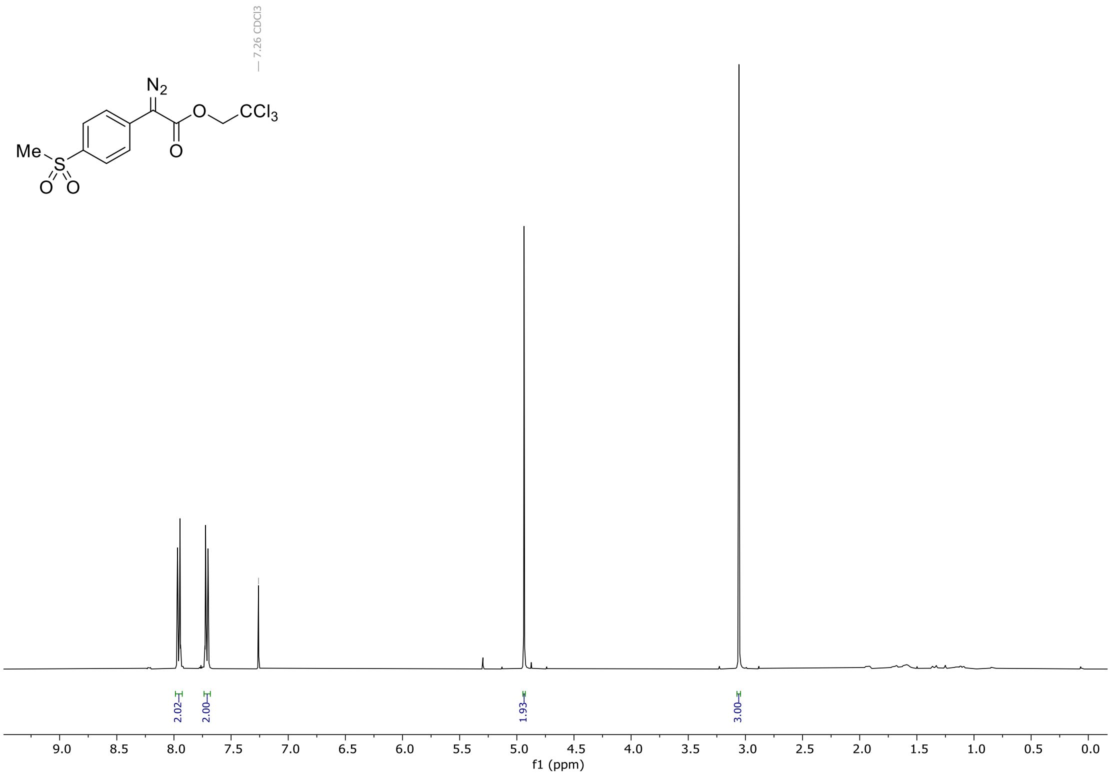

4h: ^{13}C NMR (101 MHz, $CDCl_3$):

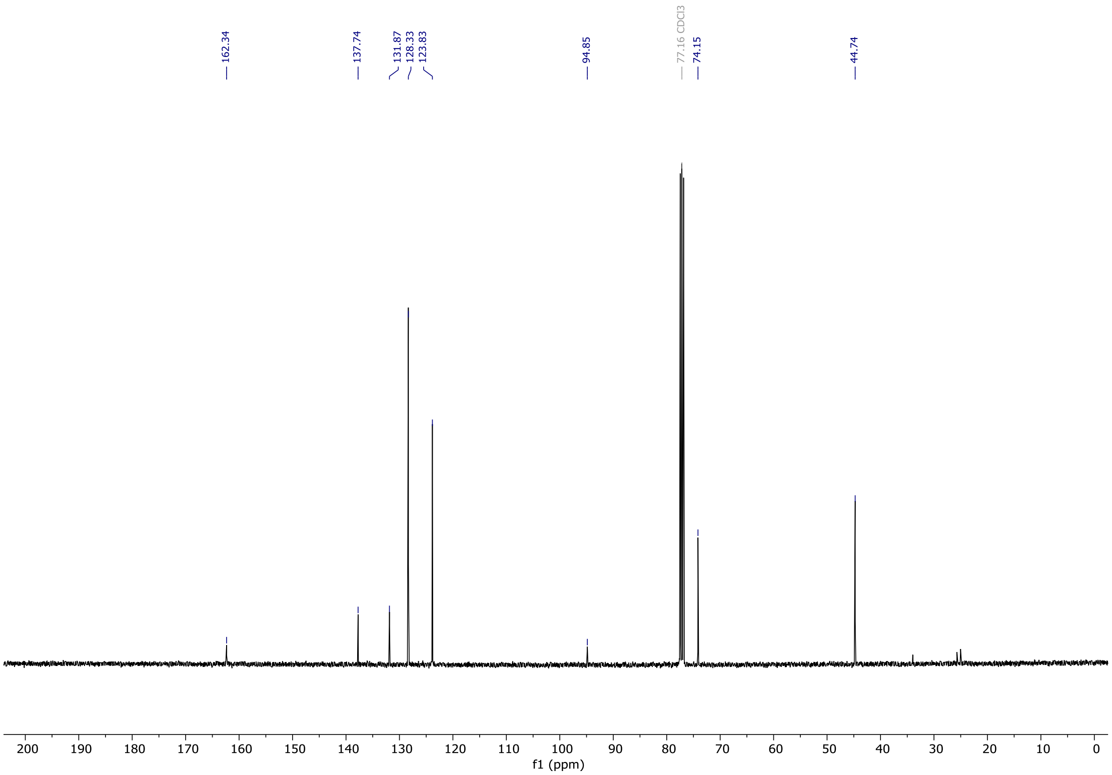

4k: ^{1}H NMR (400 MHz, $CDCl_3$):

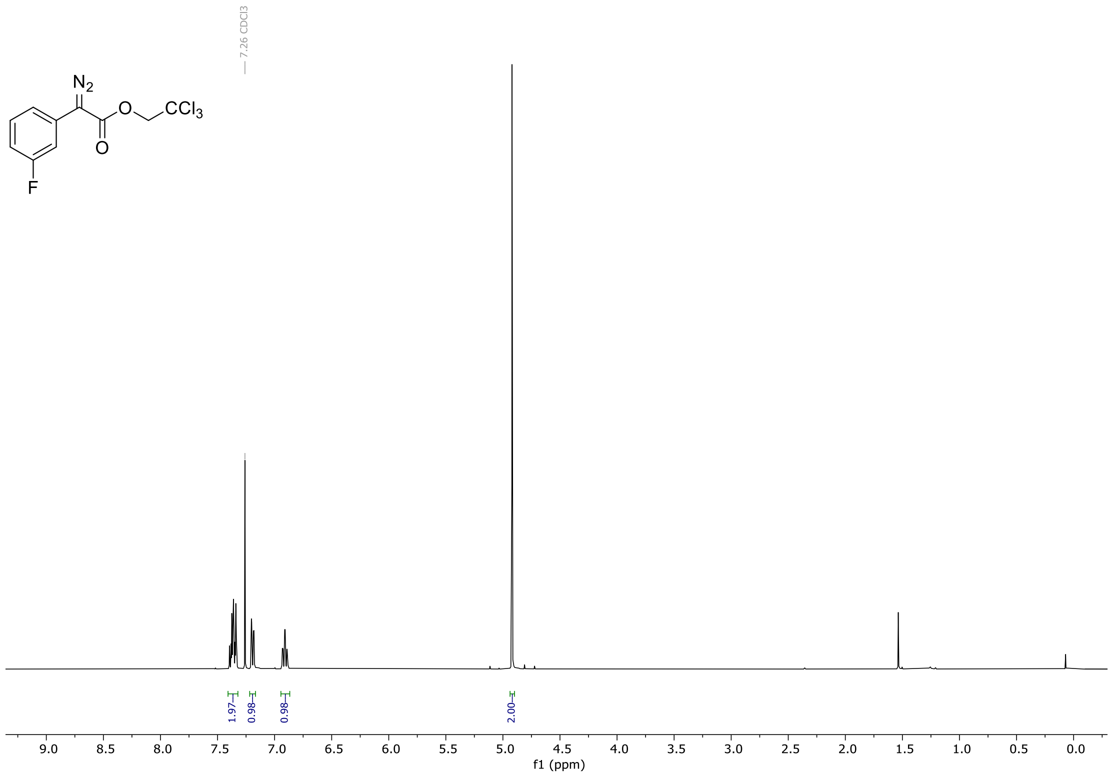

4k: ^{13}C NMR (101 MHz, $CDCl_3$):

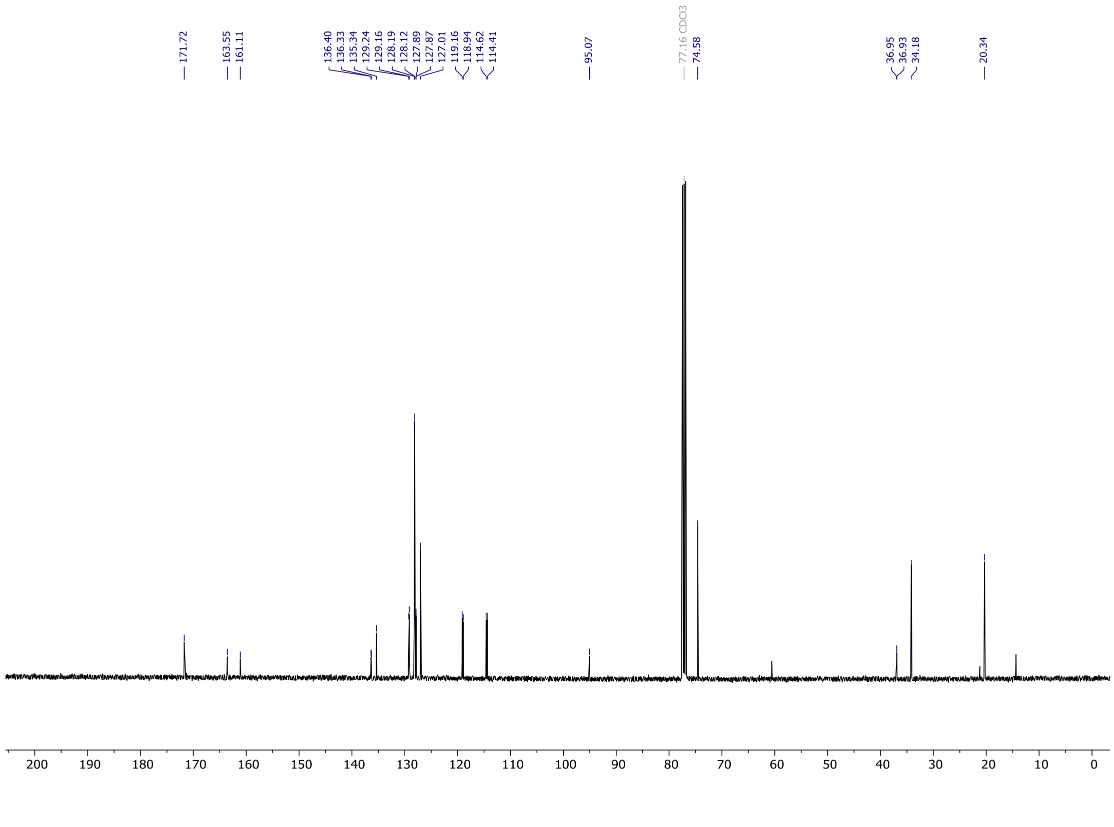

4k: ^{19}F NMR (470 MHz, $CDCl_3$):

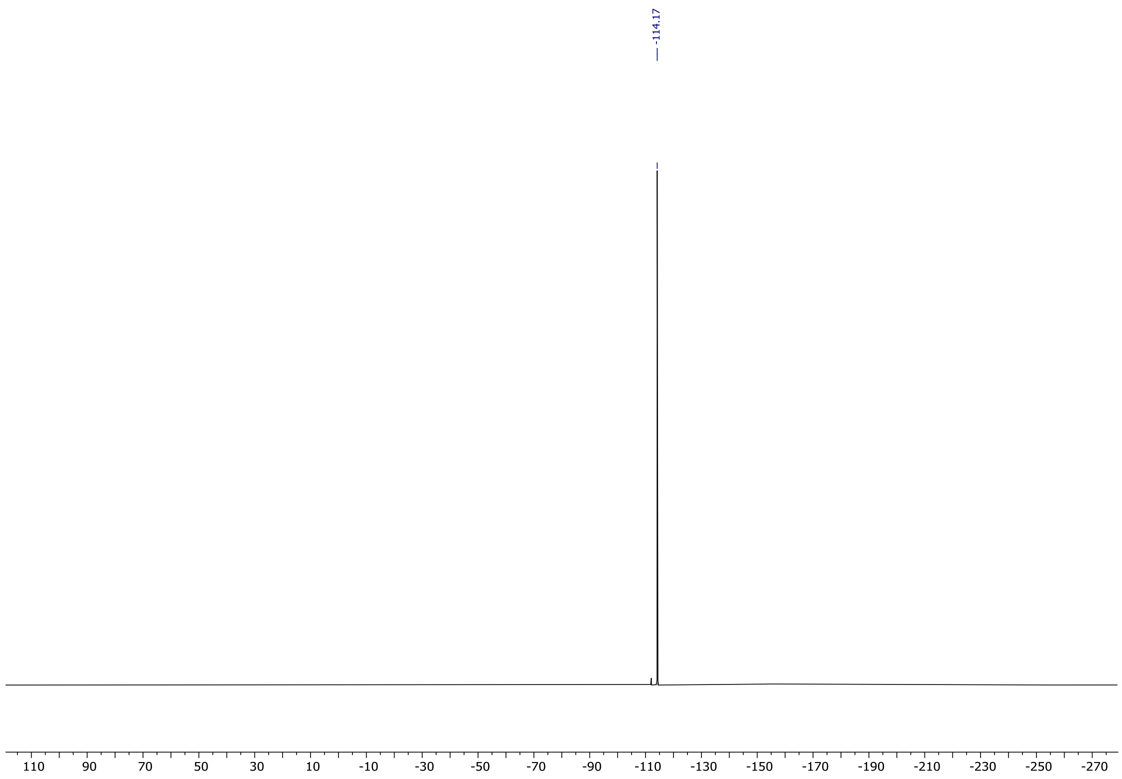

4l: ^{1}H NMR (400 MHz, $CDCl_3$):

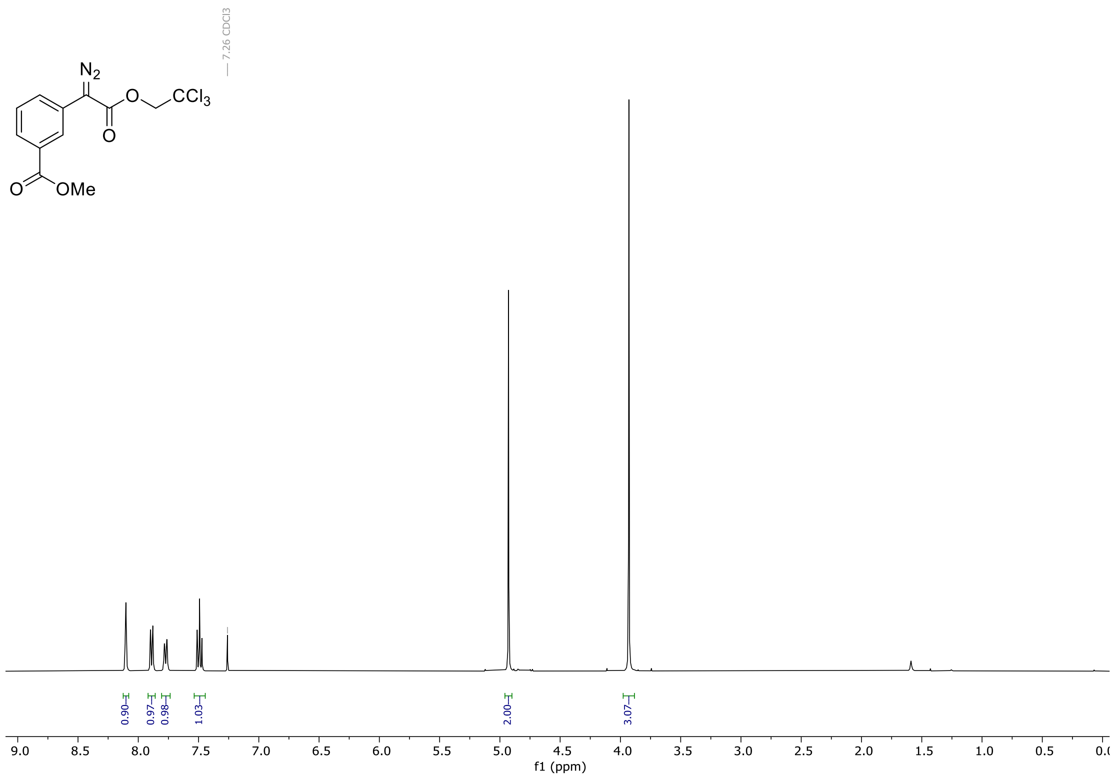

4l: ^{13}C NMR (400 MHz, $CDCl_3$):

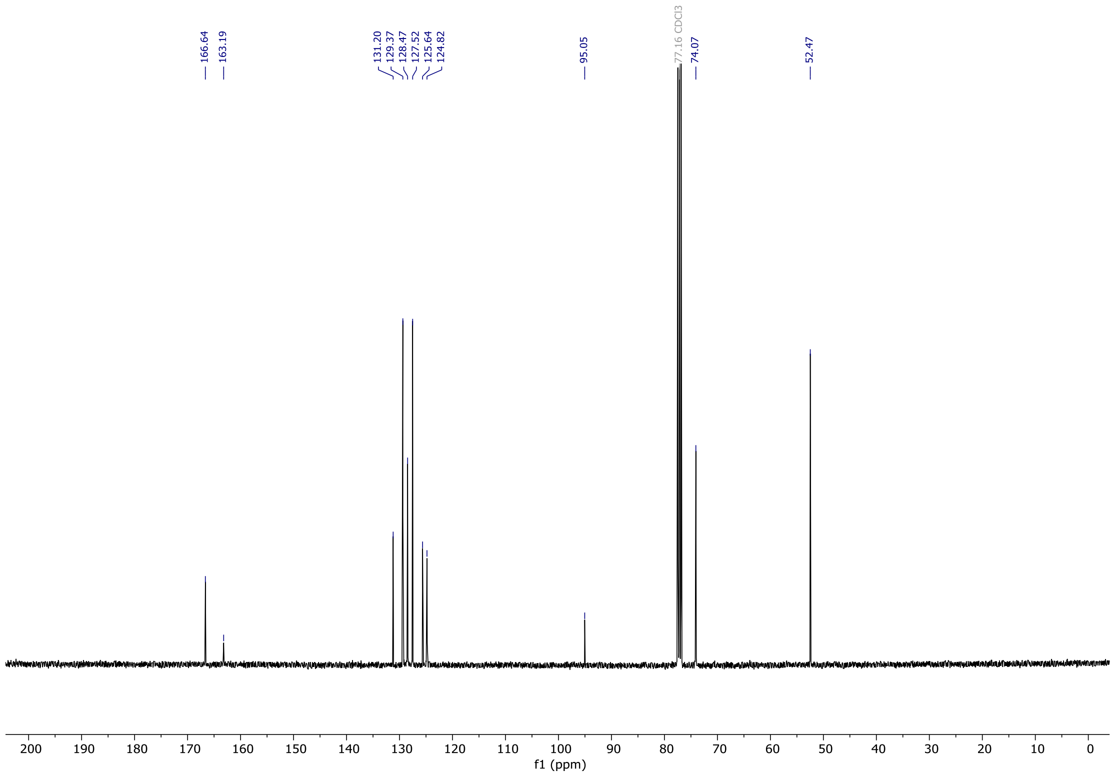

5a': ^{1}H NMR (400 MHz, $CDCl_3$):

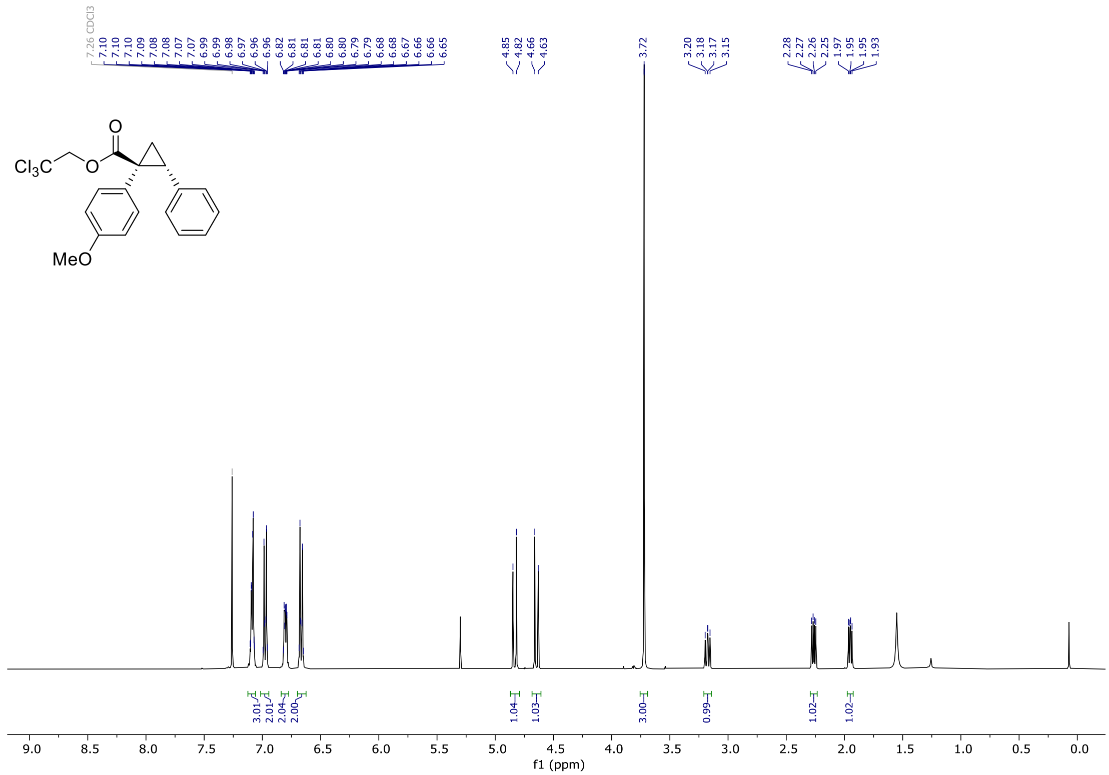

5a': ^{13}C NMR (101 MHz, $CDCl_3$):

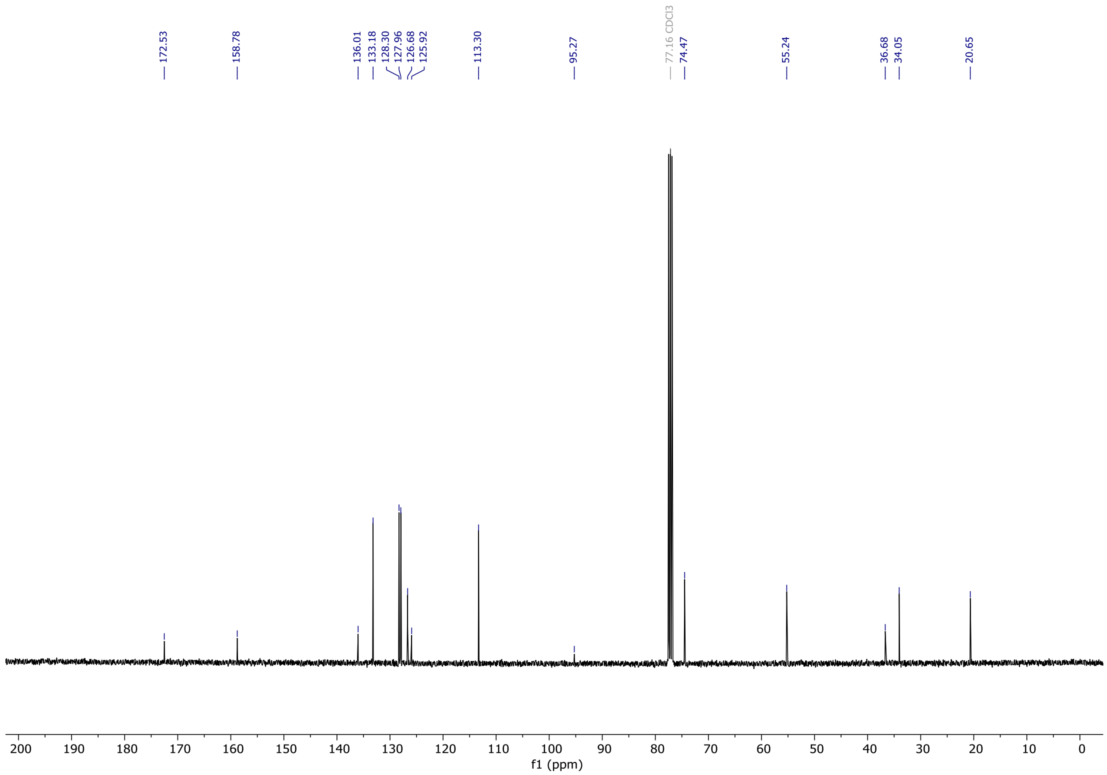

5b': ^{1}H NMR (400 MHz, $CDCl_3$):

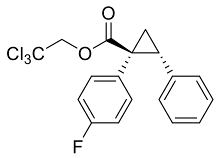

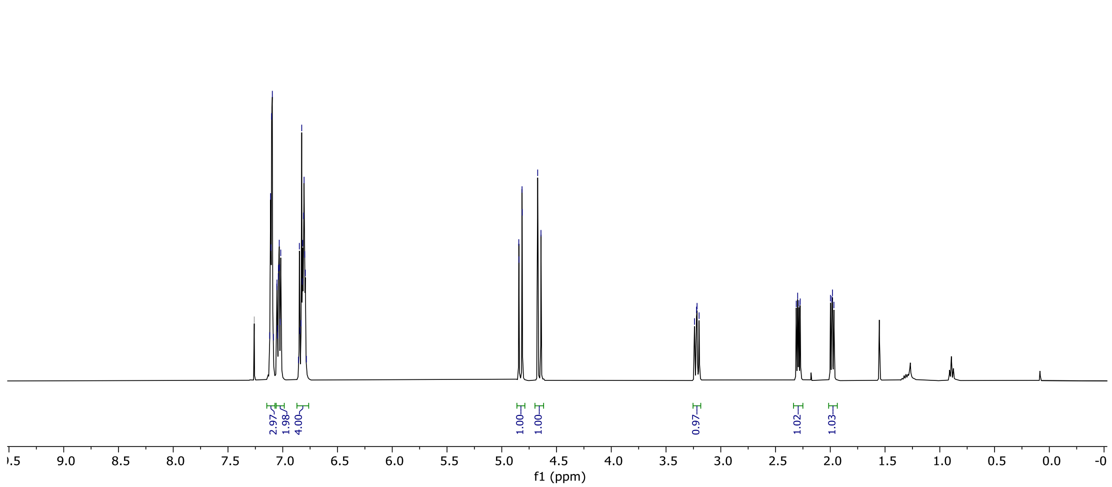

5b': ^{13}C NMR (101 MHz, $CDCl_3$):

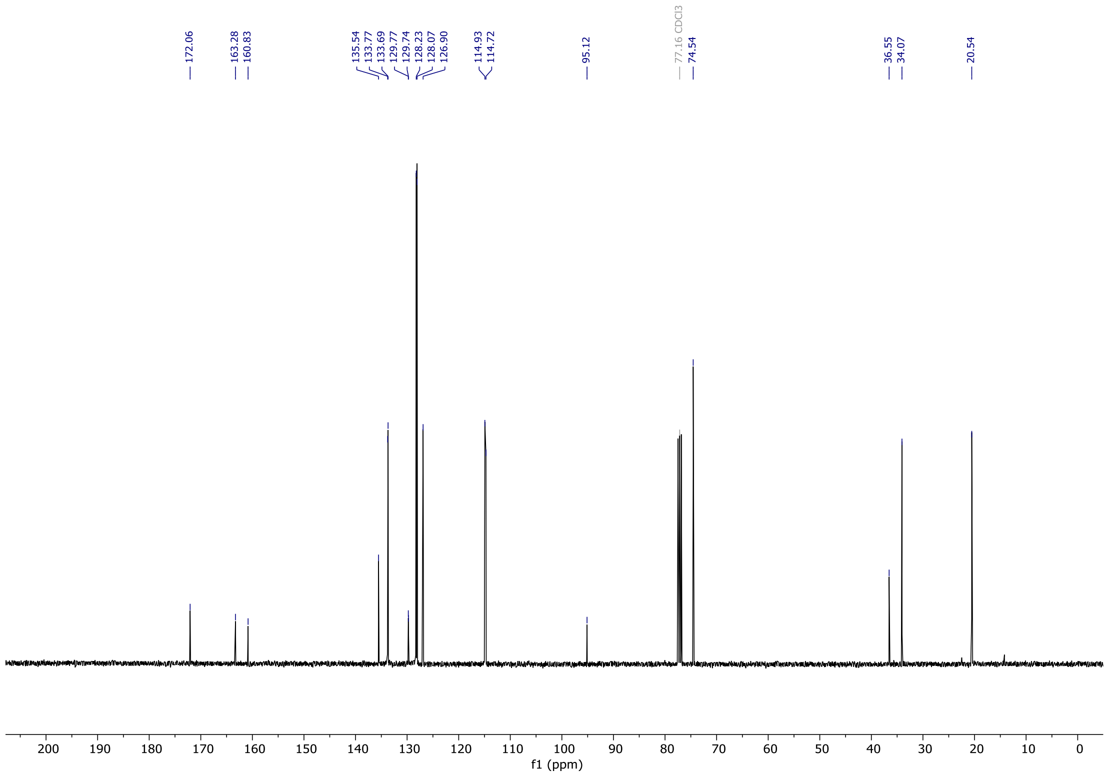

5b': ^{19}F NMR (282 MHz, $CDCl_3$):

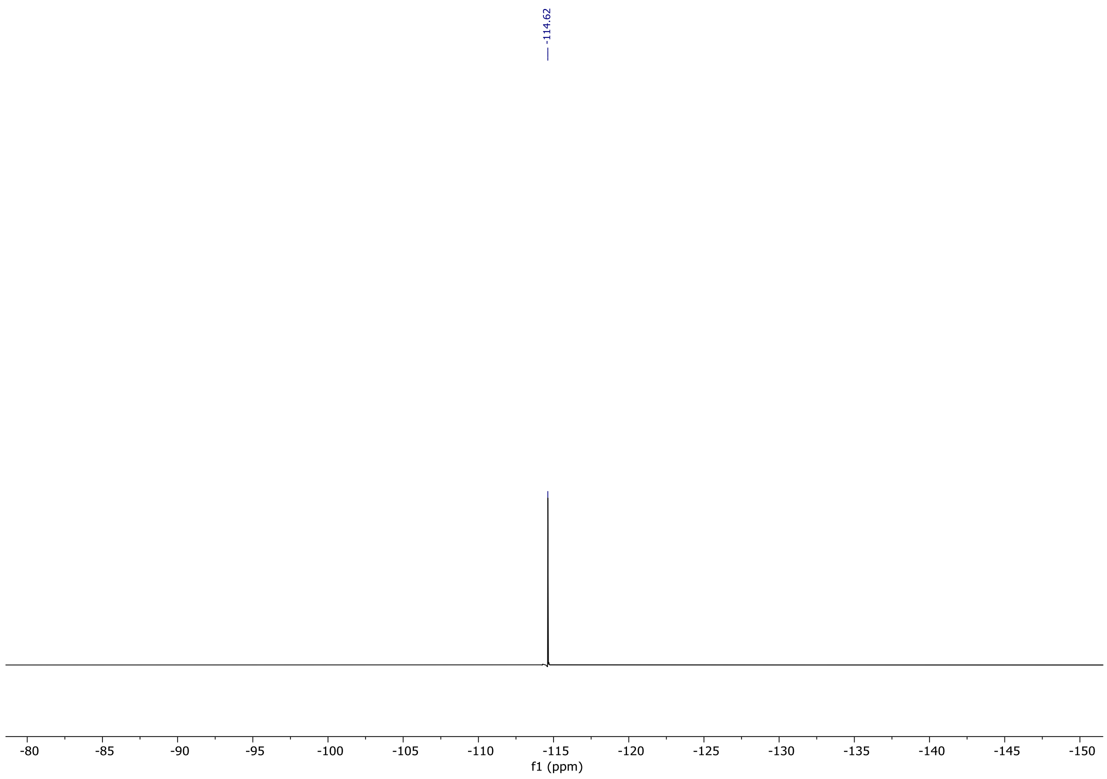

S4: ^{1}H NMR (400 MHz, $CDCl_3$):

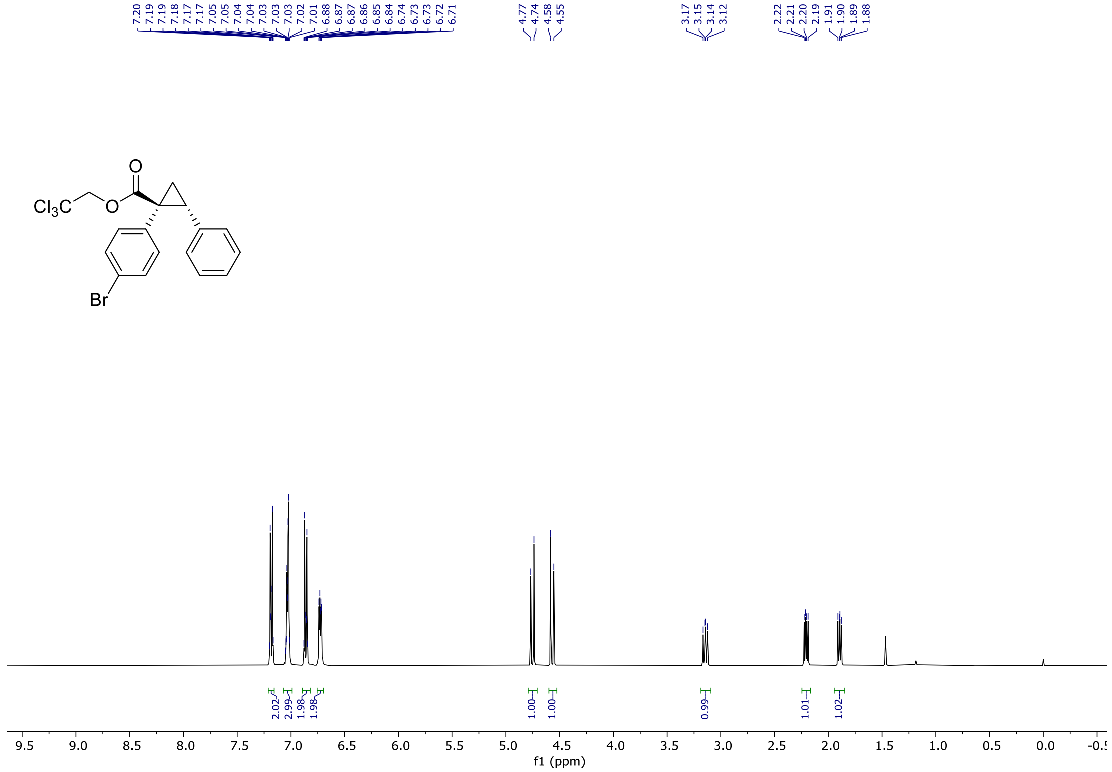

S4: ^{13}C NMR (101 MHz, $CDCl_3$):

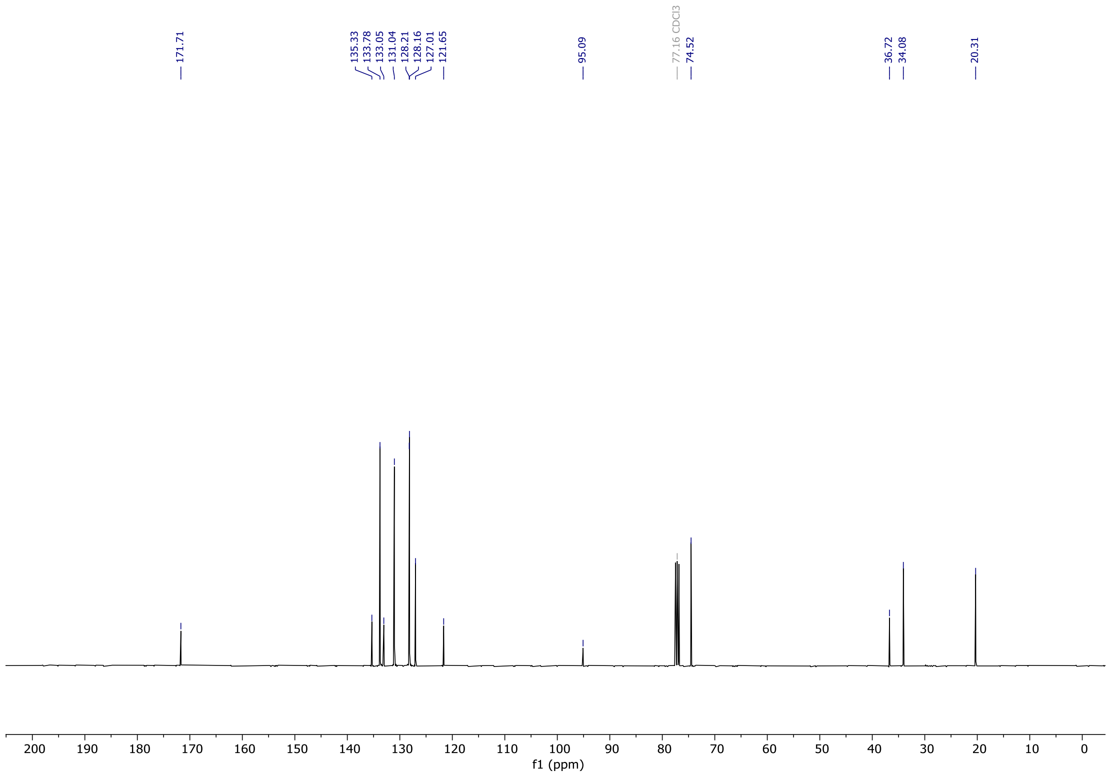

S5: ^{1}H NMR (400 MHz, $CDCl_3$):

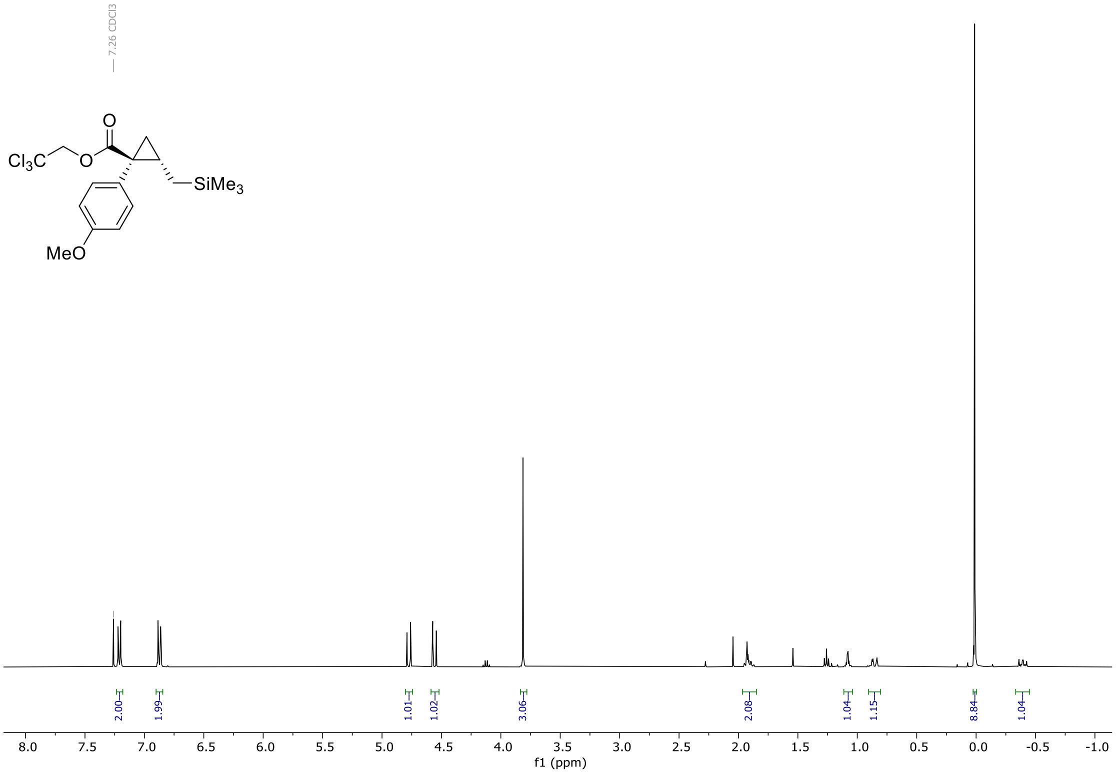

S5: ^{13}C NMR (101 MHz, $CDCl_3$):

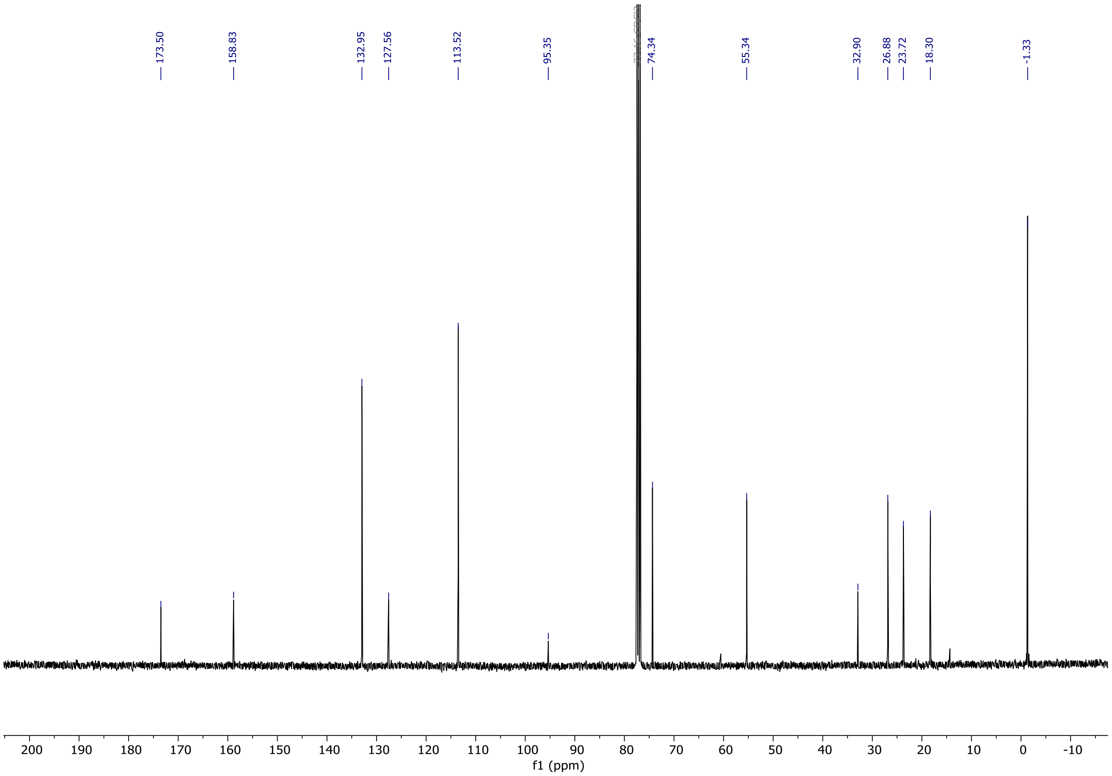

S5: ^{29}Si NMR (99 MHz, $CDCl_3$):

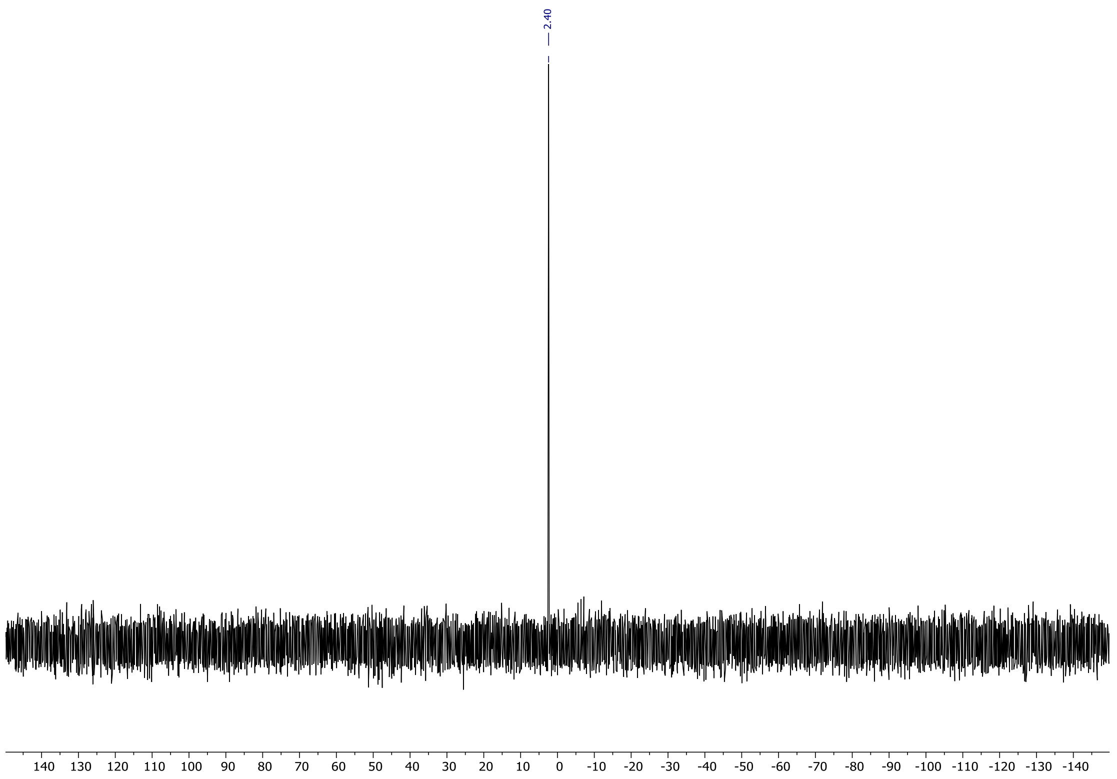

S5: HSQC NMR (400 MHz, 101 MHz, $CDCl_3$):

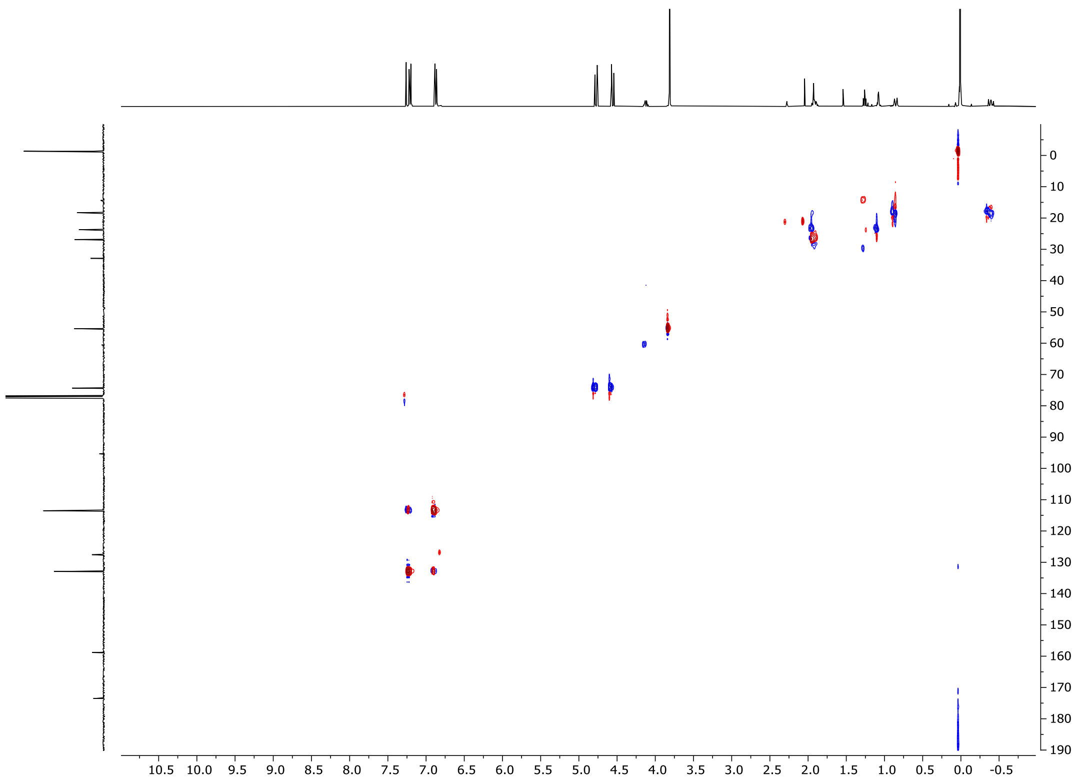

S5: HMBC NMR (400 MHz, 101 MHz, $CDCl_3$):

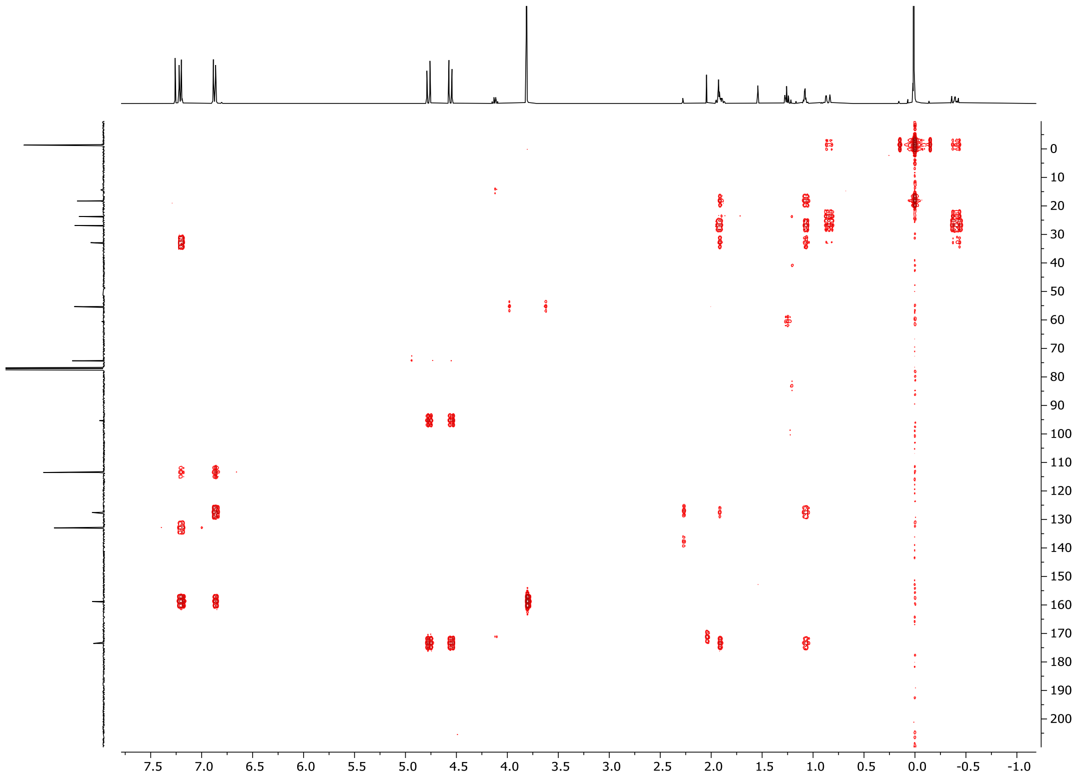

S5: NOESY NMR (400 MHz, $CDCl_3$):

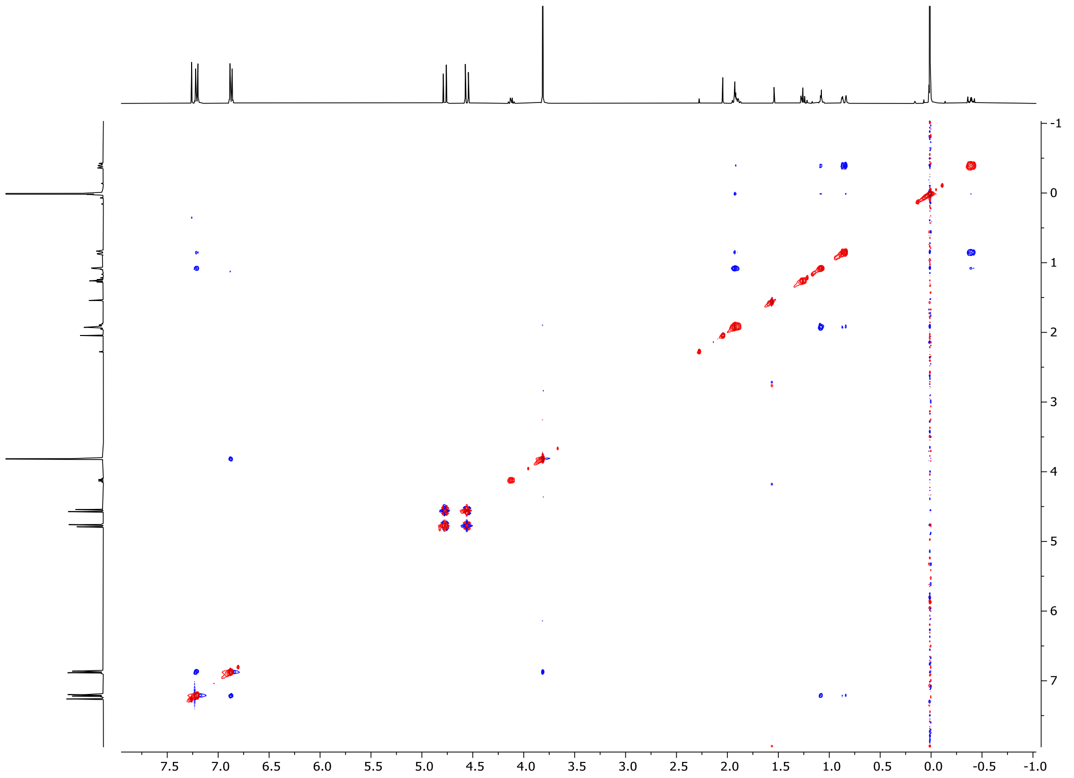

S6: ^{1}H NMR (400 MHz, $CDCl_3$):

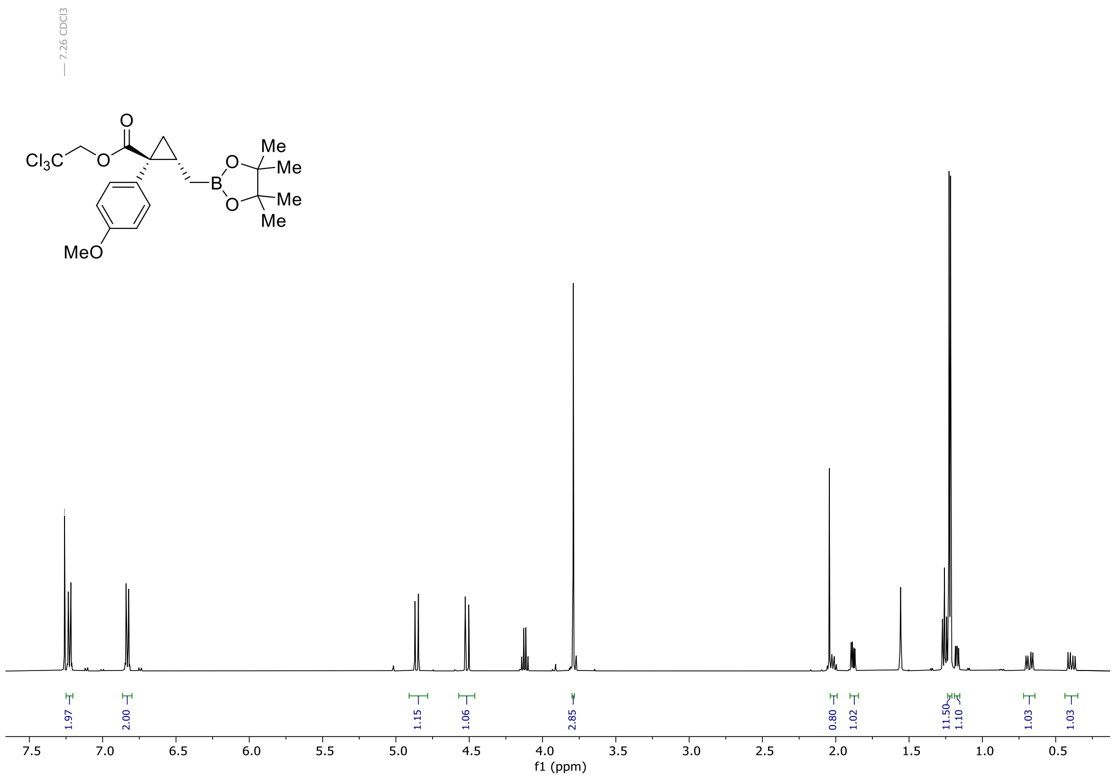

S6: ^{13}C NMR (101 MHz, $CDCl_3$):

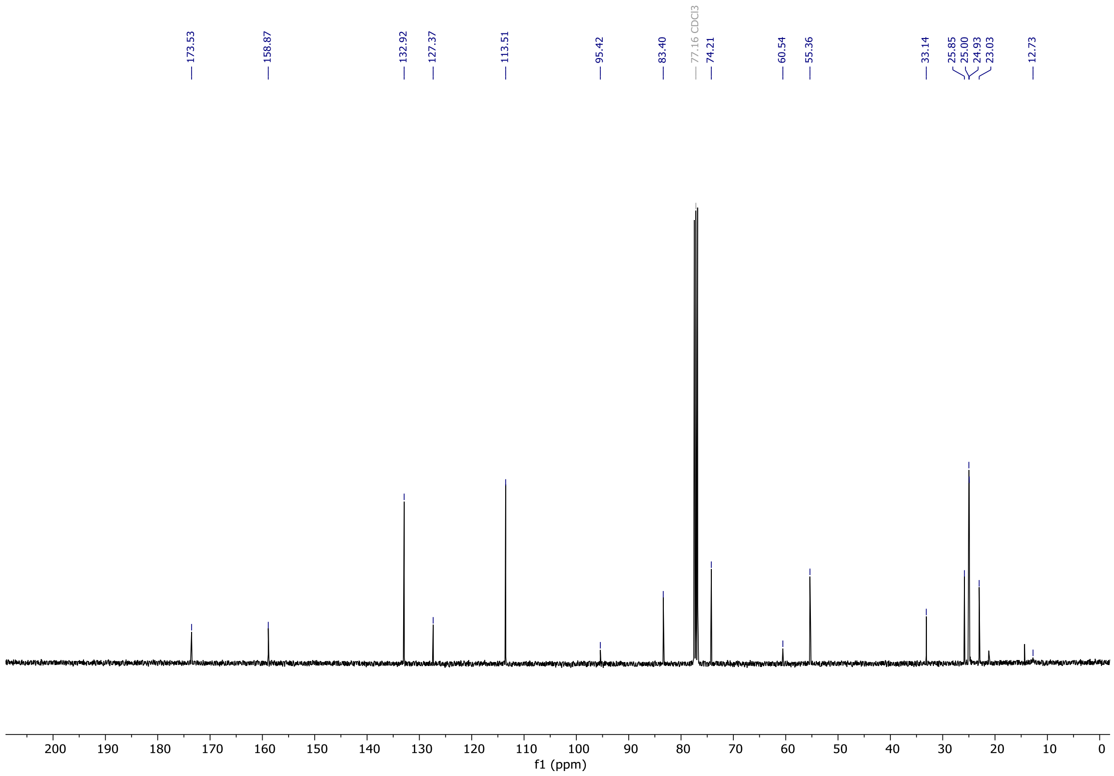

S6: ^{11}B NMR (128 MHz, $CDCl_3$):

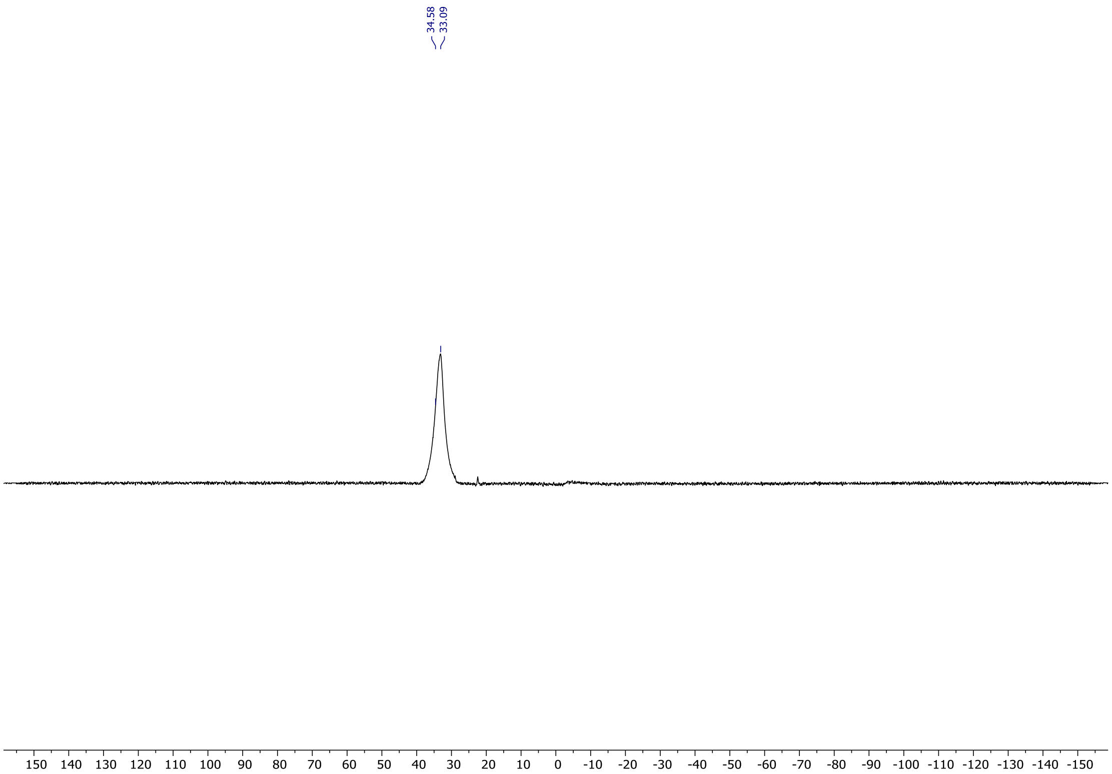

S6: HSQC NMR (400 MHz, 101 MHz, $CDCl_3$):

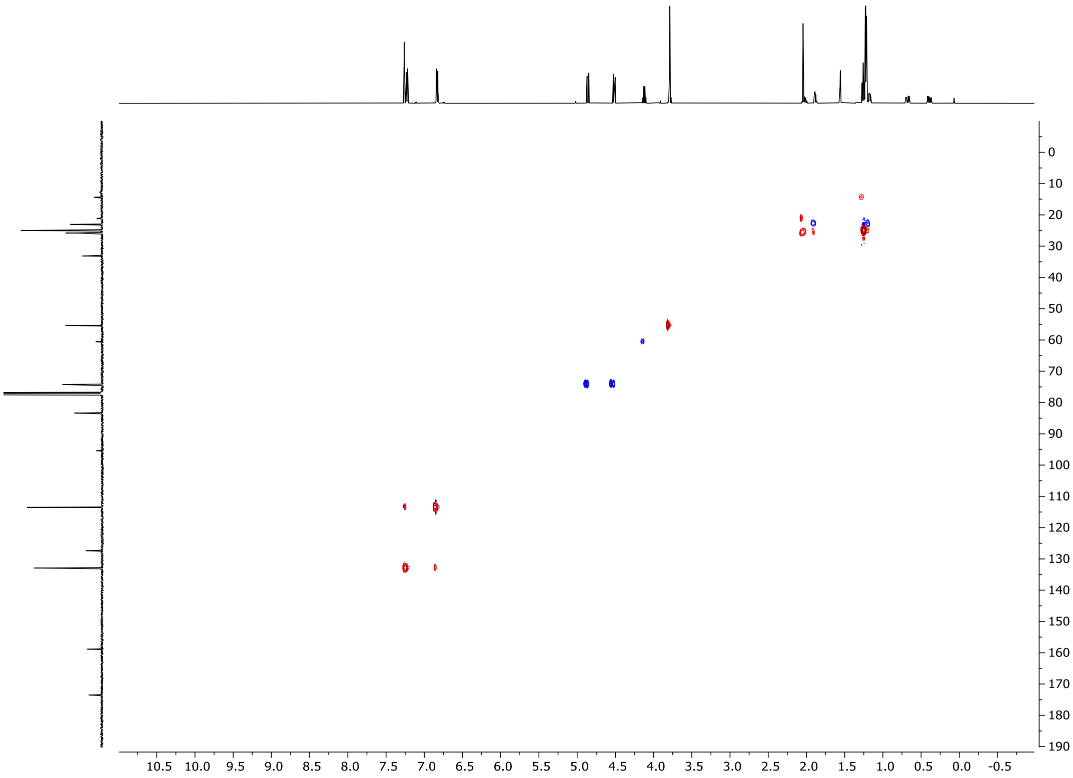

S6: HMBC NMR (400 MHz, 101 MHz, $CDCl_3$):

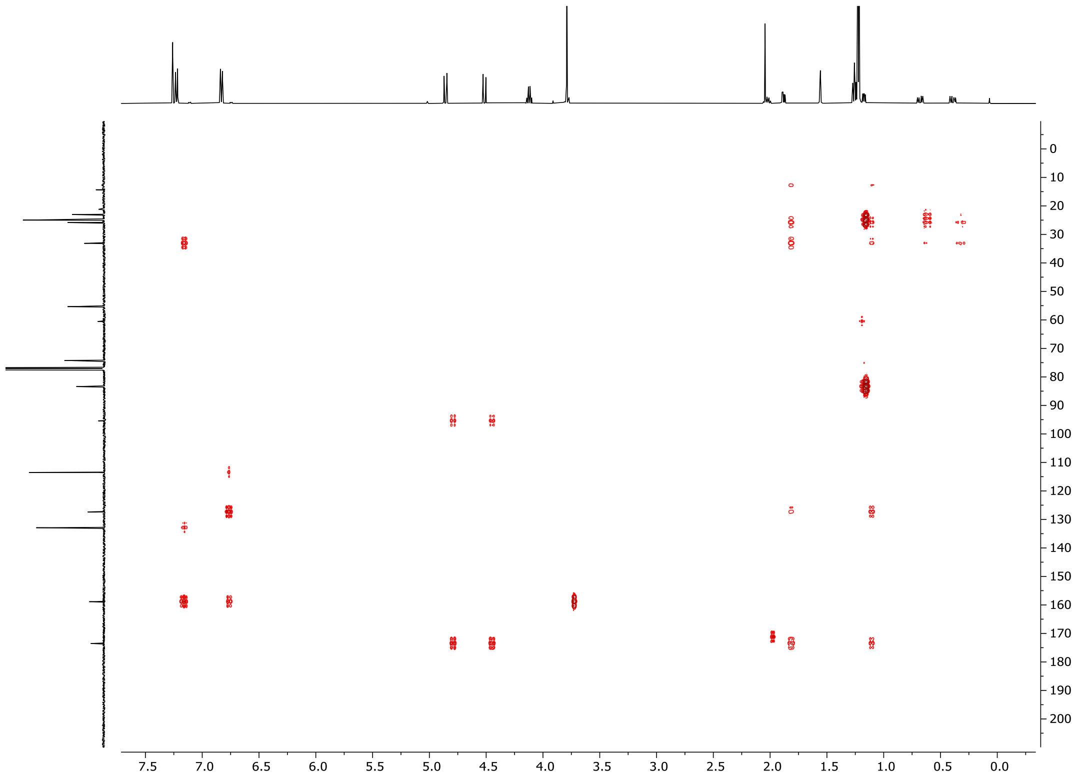

S6: NOESY NMR (400 MHz, $CDCl_3$):

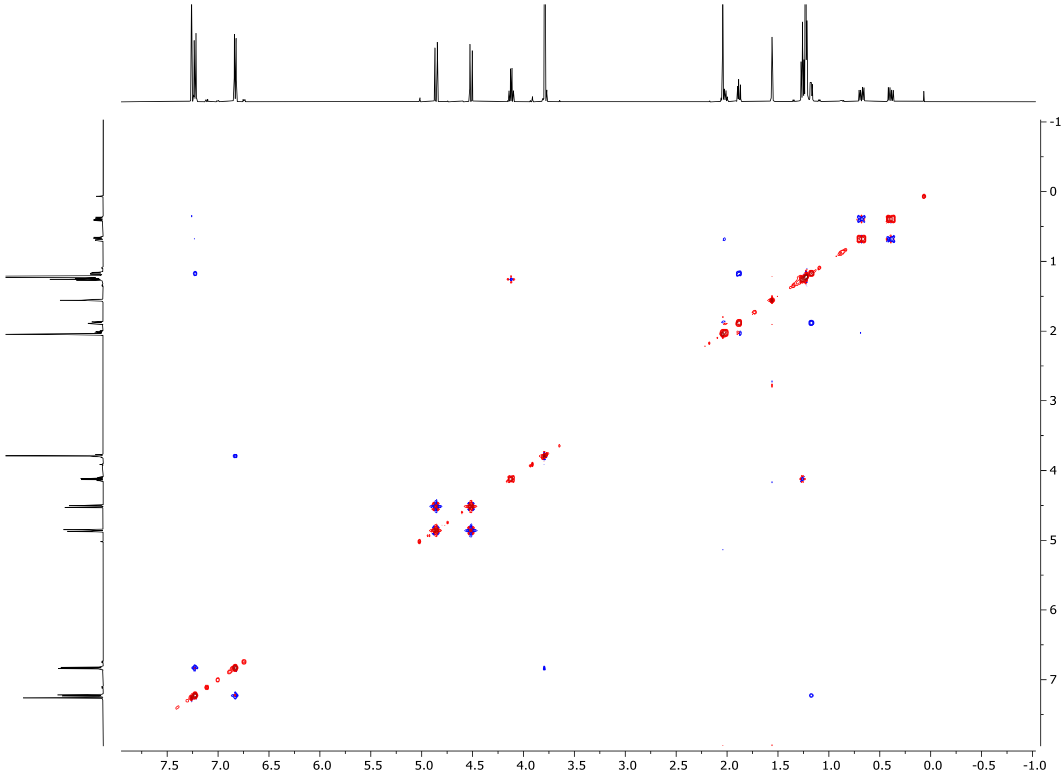

S7: ^{1}H NMR (400 MHz, $CDCl_3$):

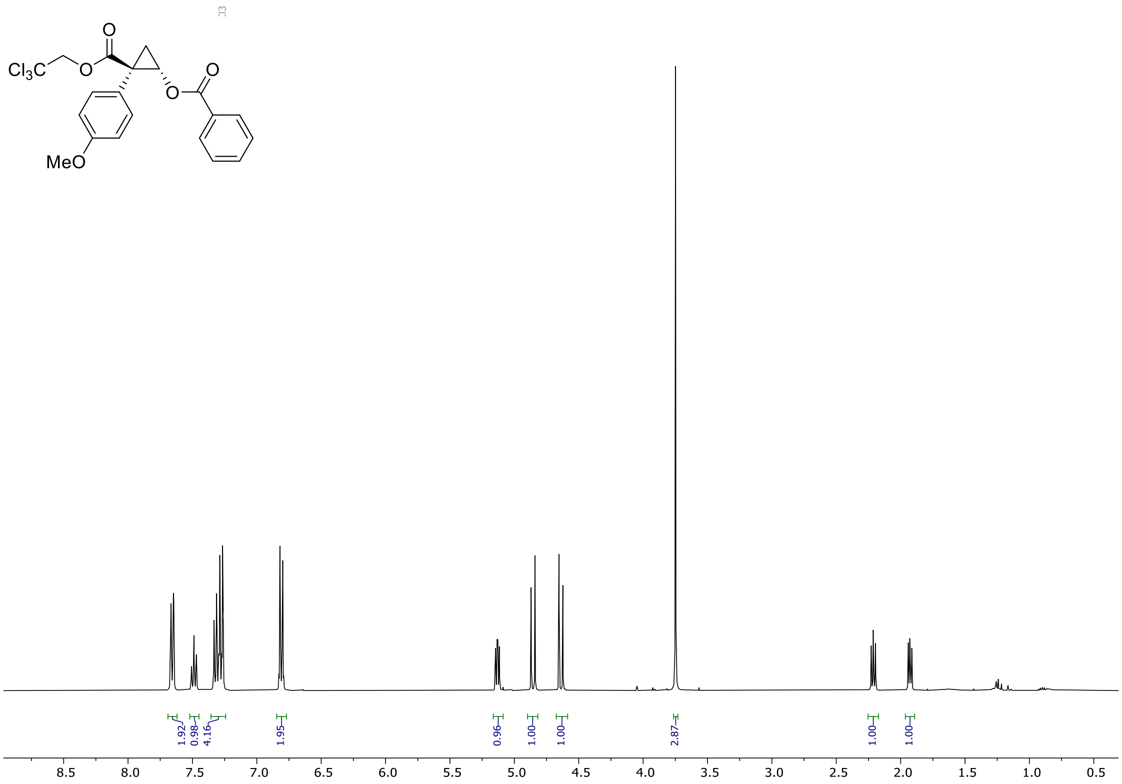

S7: ^{13}C NMR (101 MHz, $CDCl_3$)

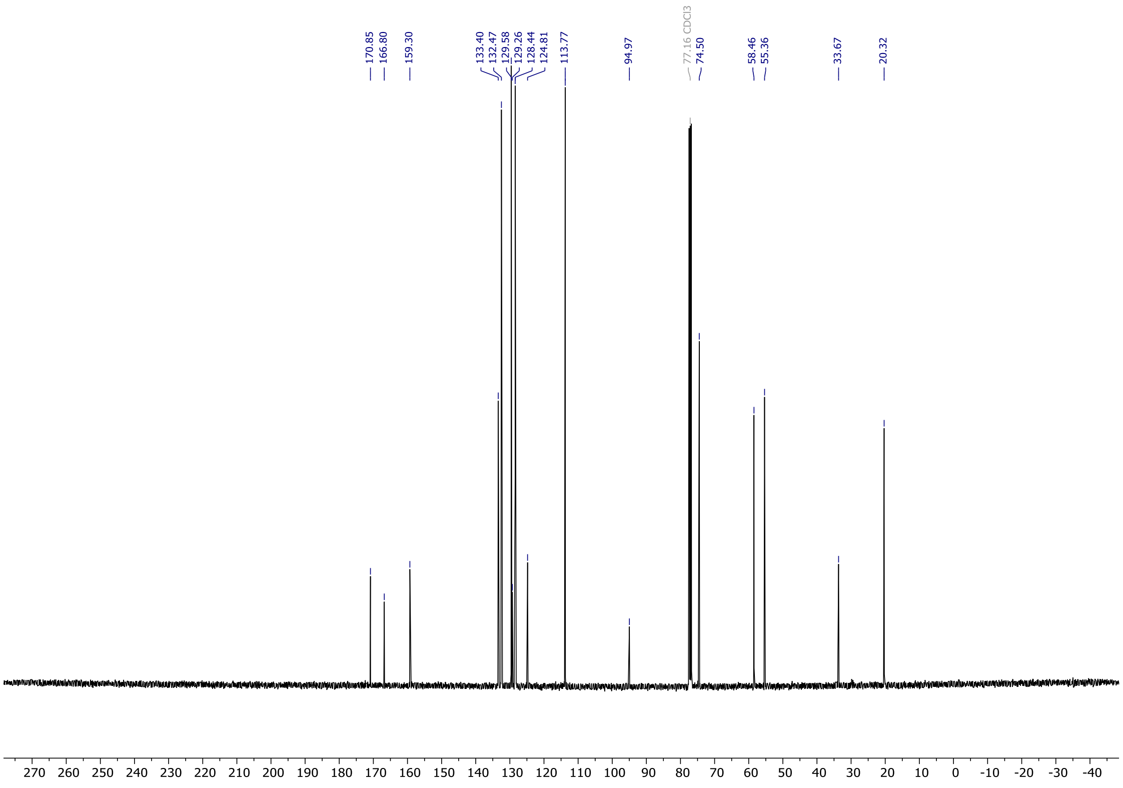

S7: HSQC NMR (400 MHz, 101 MHz, $CDCl_3$):

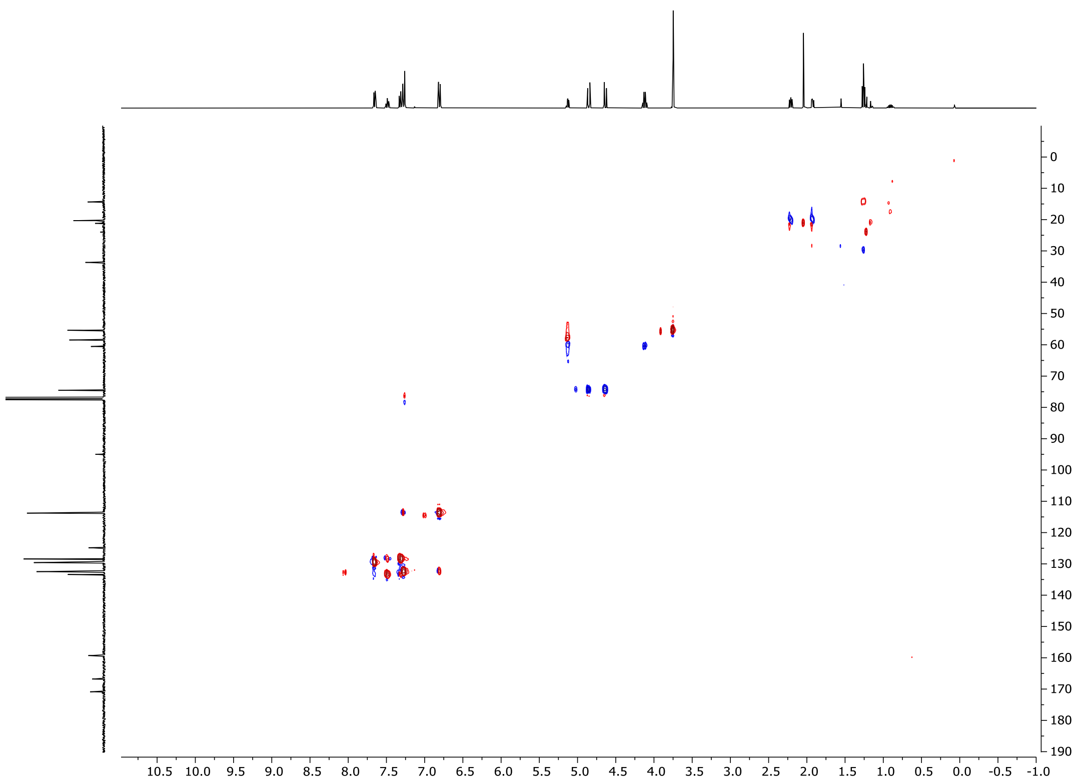

S7: HMBC NMR (400 MHz, 101 MHz, CDCl3):

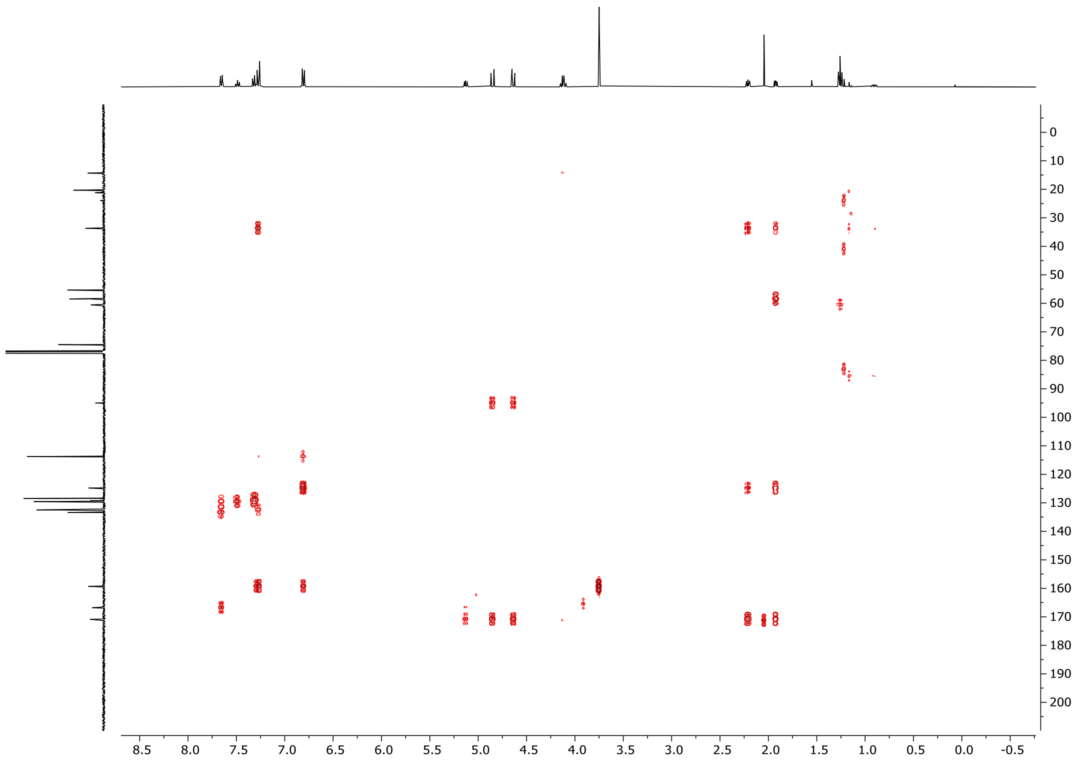

S7: NOESY NMR (500 MHz, $CDCl_3$):

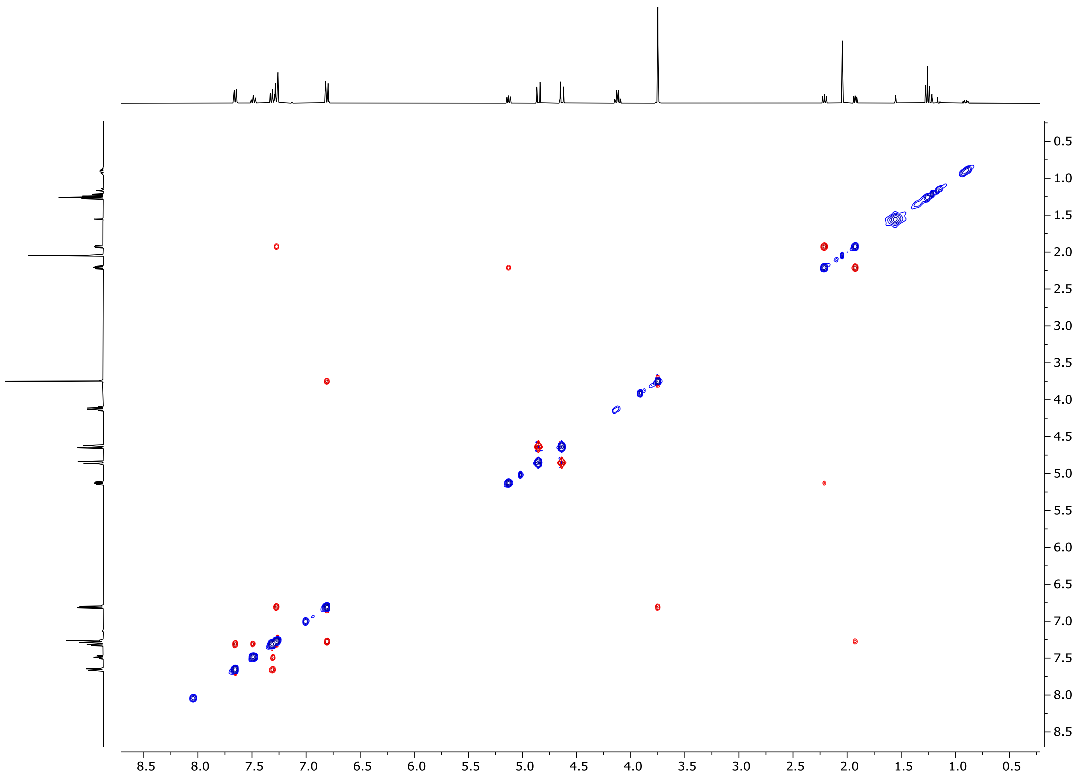

S8: ^{1}H NMR (400 MHz, $CDCl_3$):

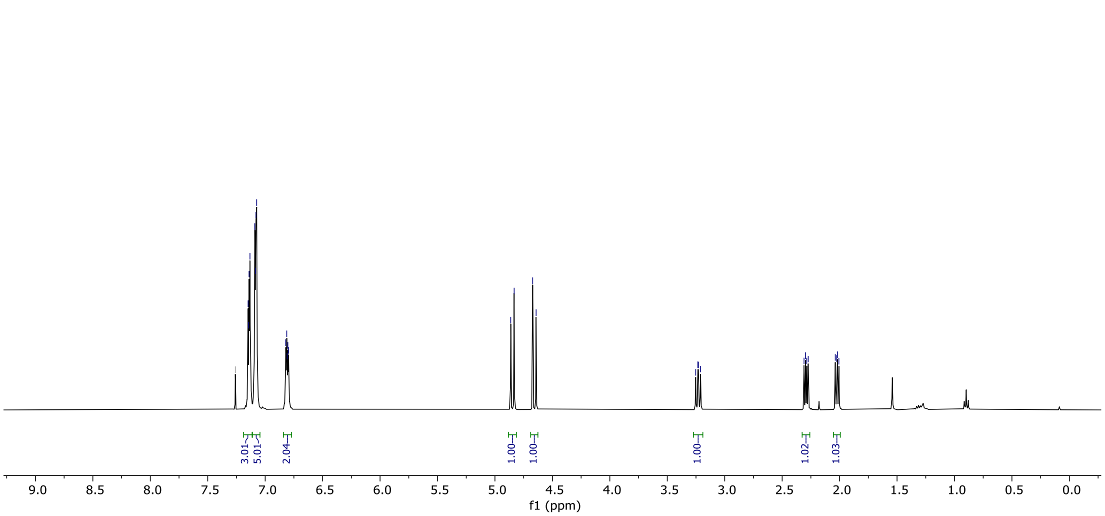

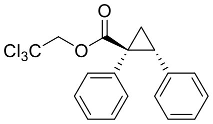

S8: ^{13}C NMR (101 MHz, $CDCl_3$):

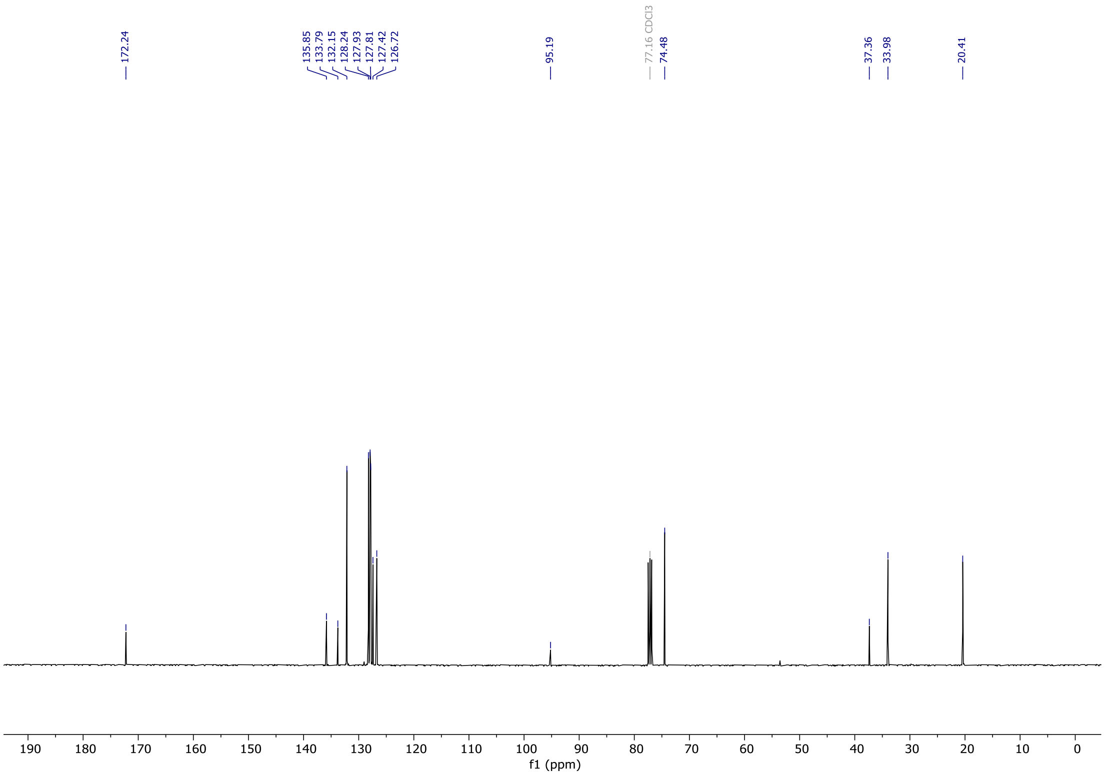

S9: ^{1}H NMR (400 MHz, $CDCl_3$):

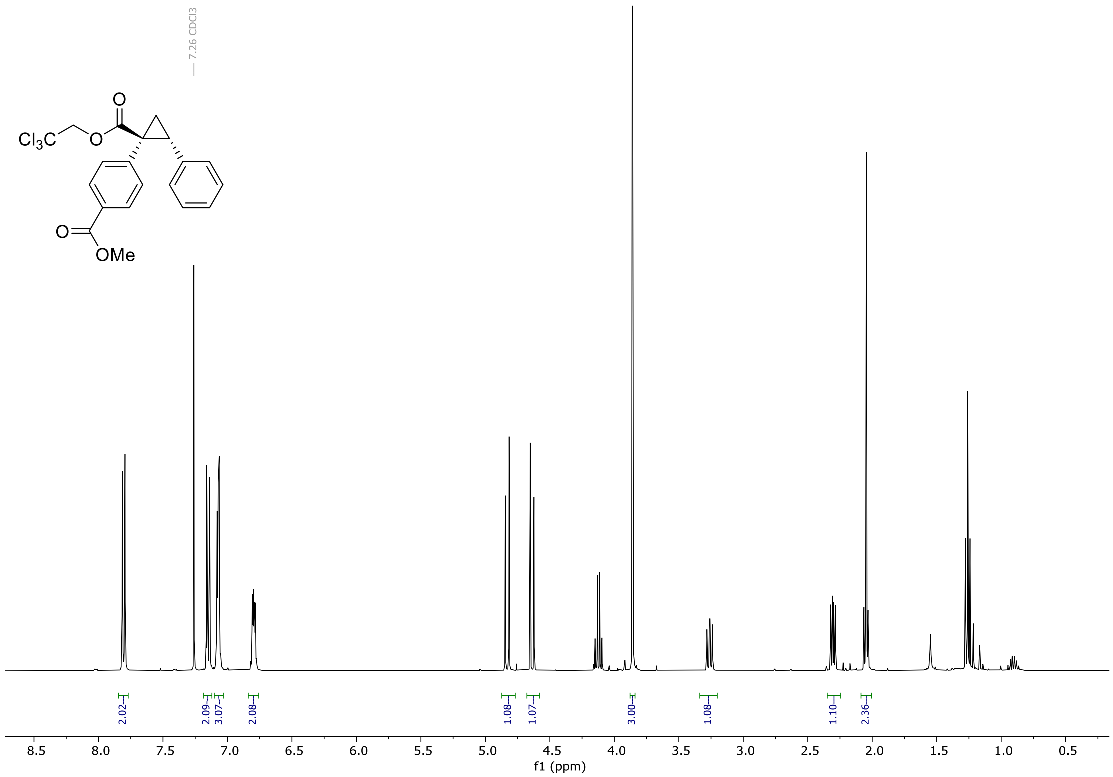

S9: ^{13}C NMR (101 MHz, $CDCl_3$):

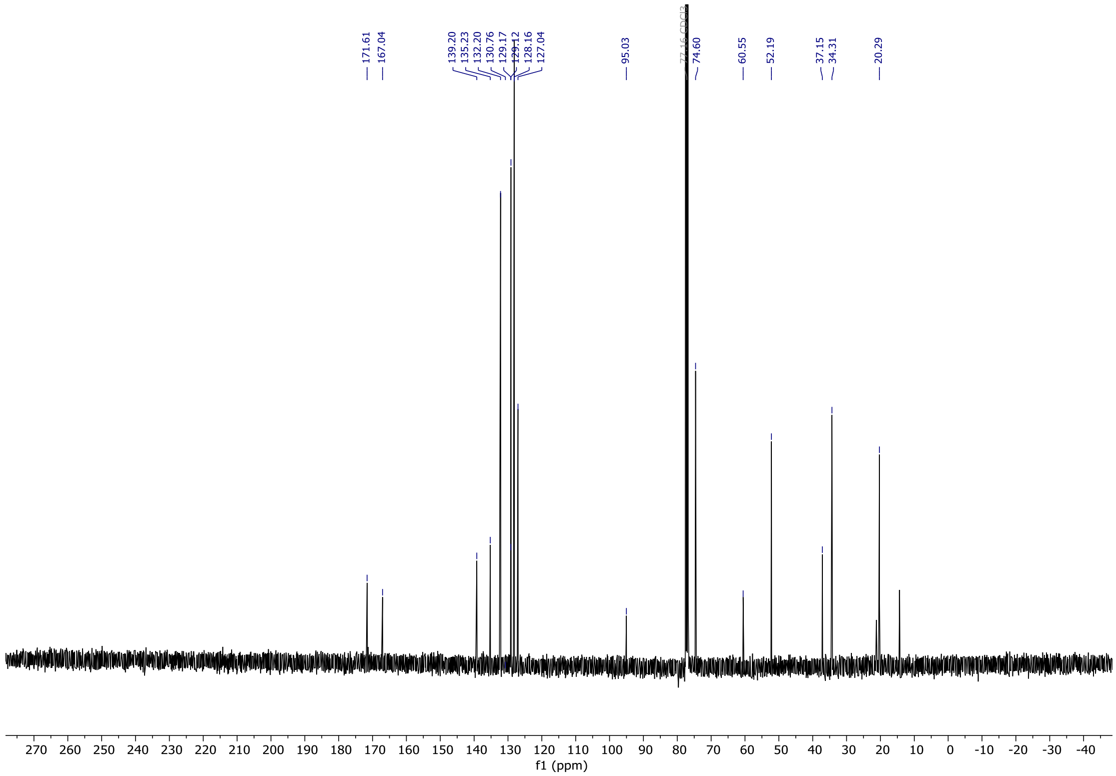

S9: HSQC NMR (400 MHz, 101 MHz, $CDCl_3$):

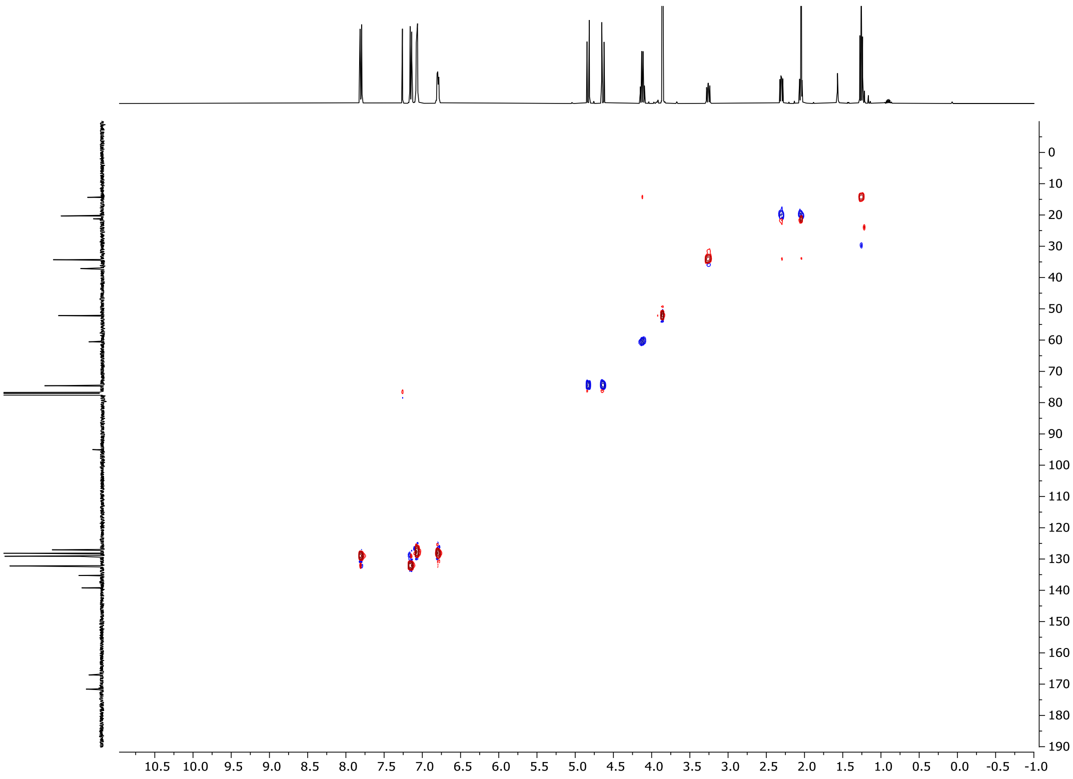

S9: HMBC NMR (400 MHz, 101 MHz, $CDCl_3$):

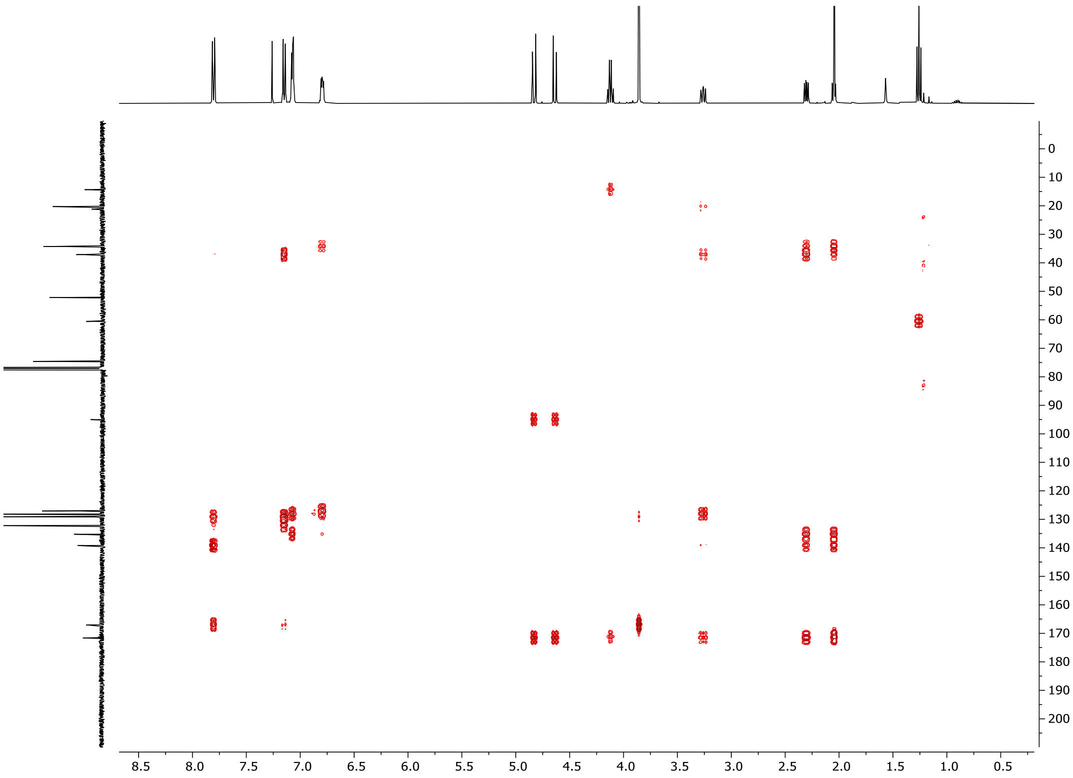

S9: NOESY NMR (400 MHz, $CDCl_3$):

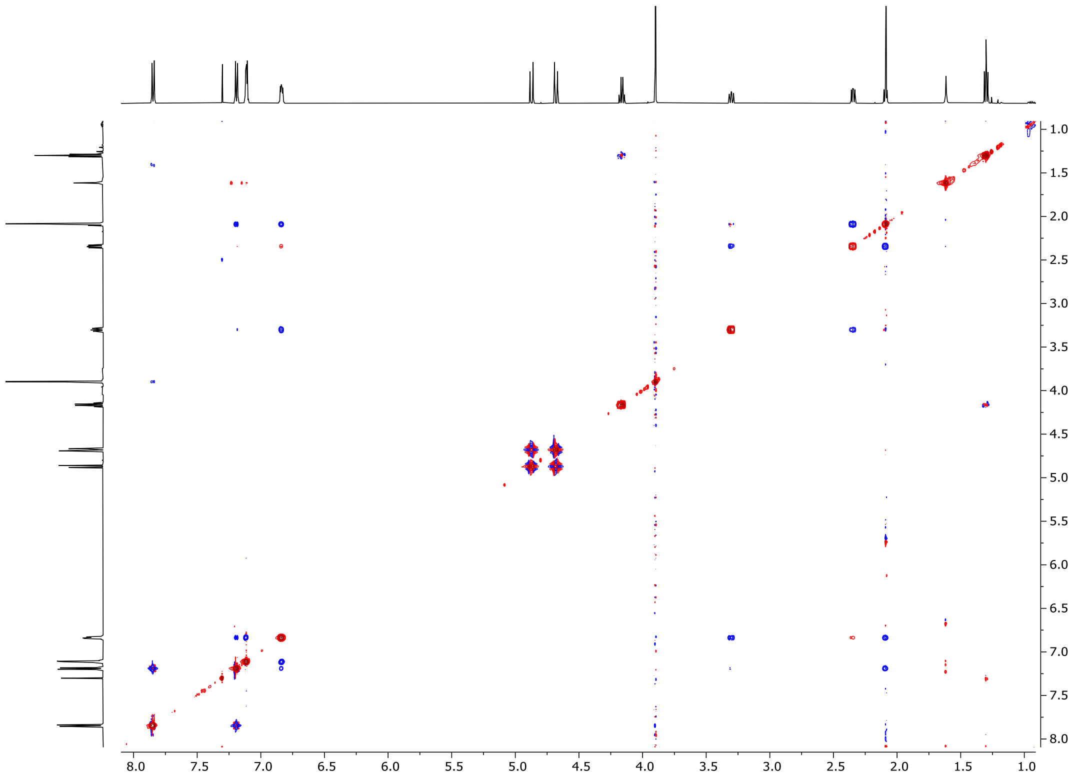

S10: ^{1}H NMR (400 MHz, $CDCl_3$):

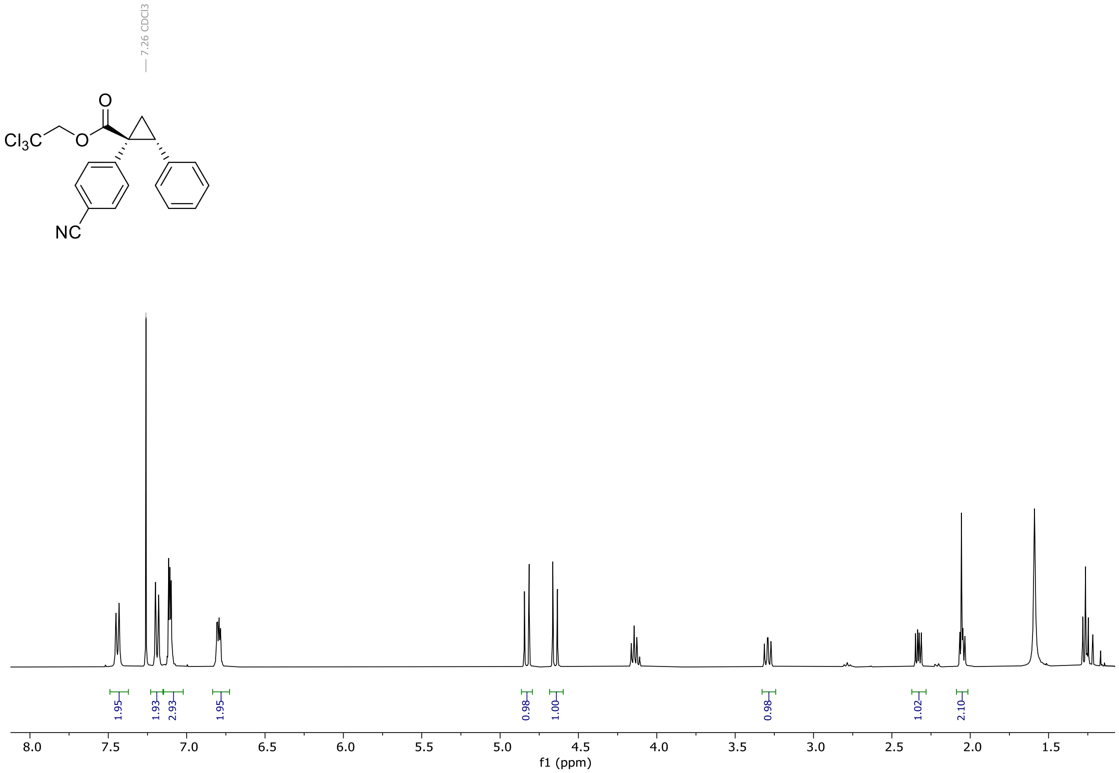

S10: ^{13}C NMR (101 MHz, $CDCl_3$):

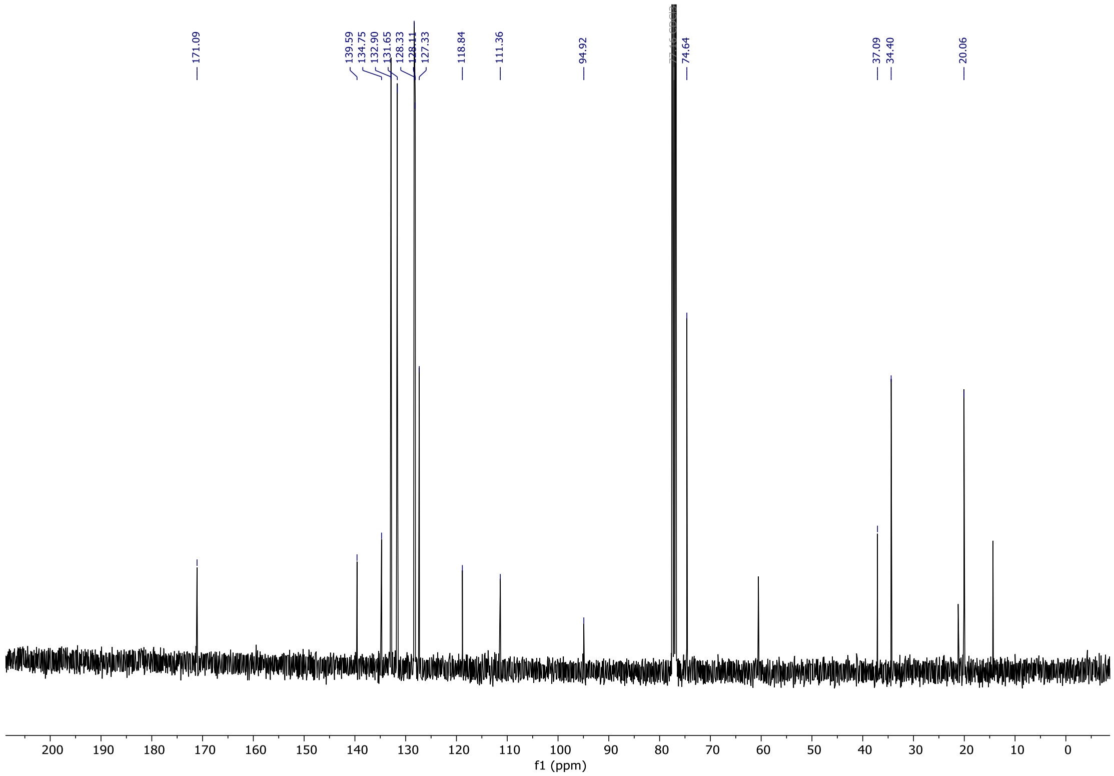

S10: HSQC NMR (400 MHz, 101 MHz, $CDCl_3$):

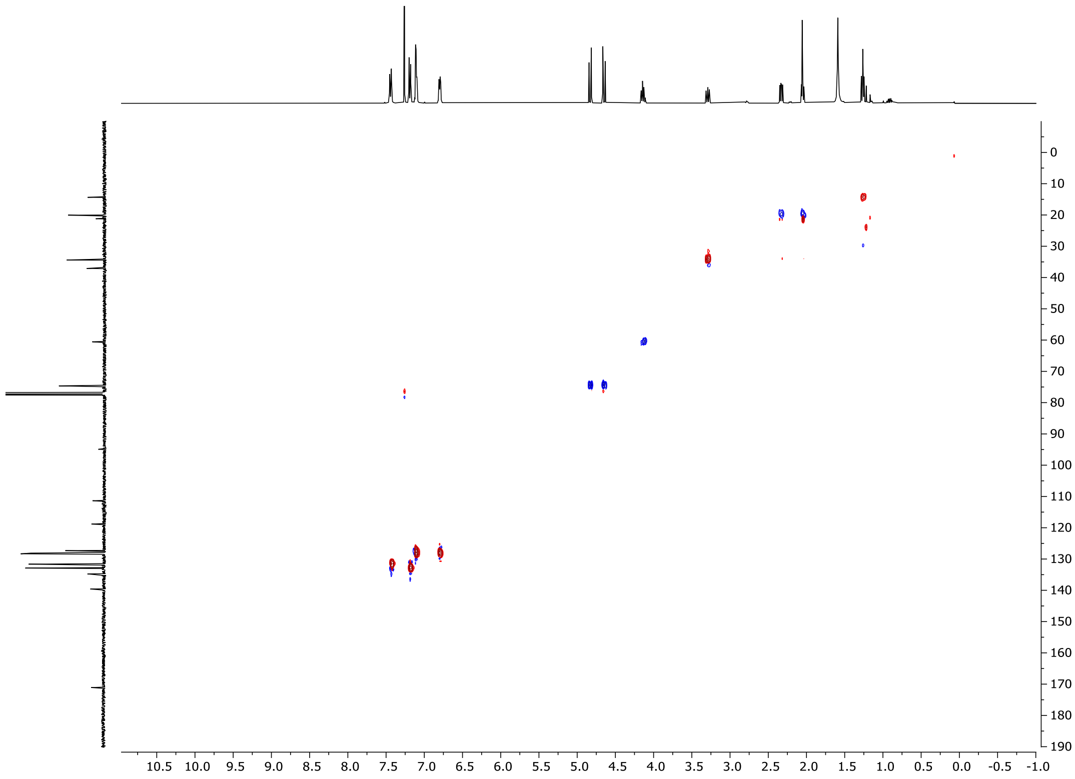

S10: HMBC NMR (400 MHz, 101 MHz, $CDCl_3$):

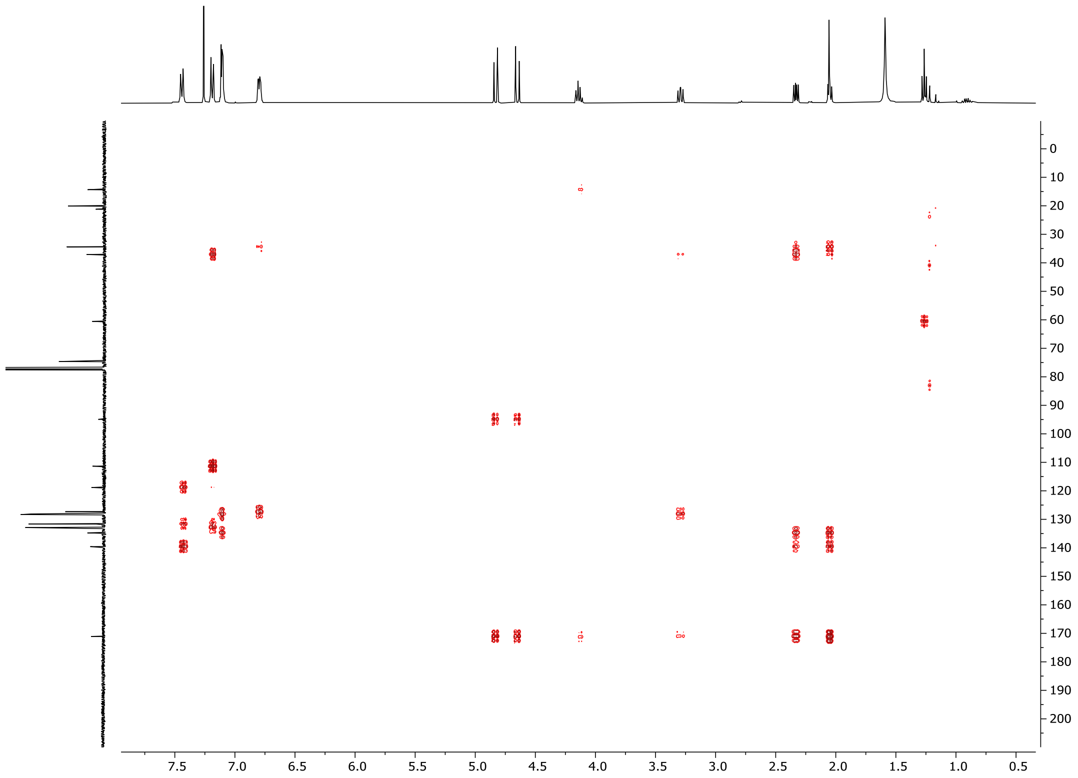

S10: NOESY NMR (500 MHz, $CDCl_3$):

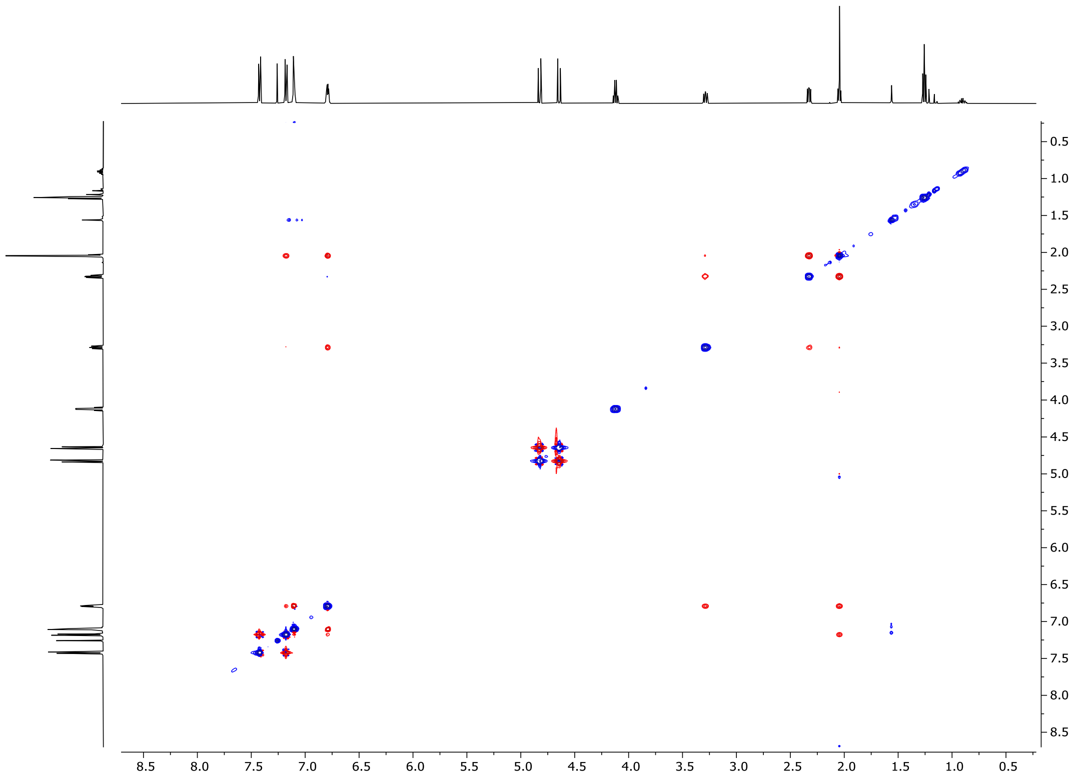

S11: ^{1}H NMR (400 MHz, $CDCl_3$):

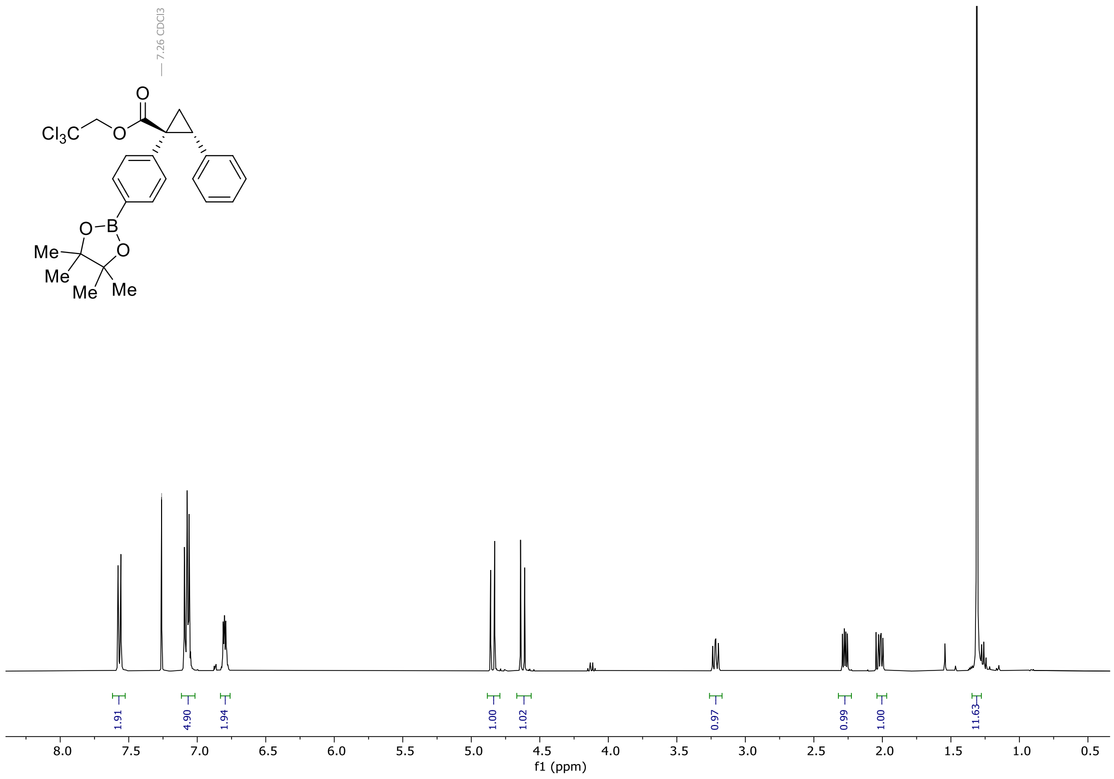

S11: ^{13}C NMR (101 MHz, $CDCl_3$):

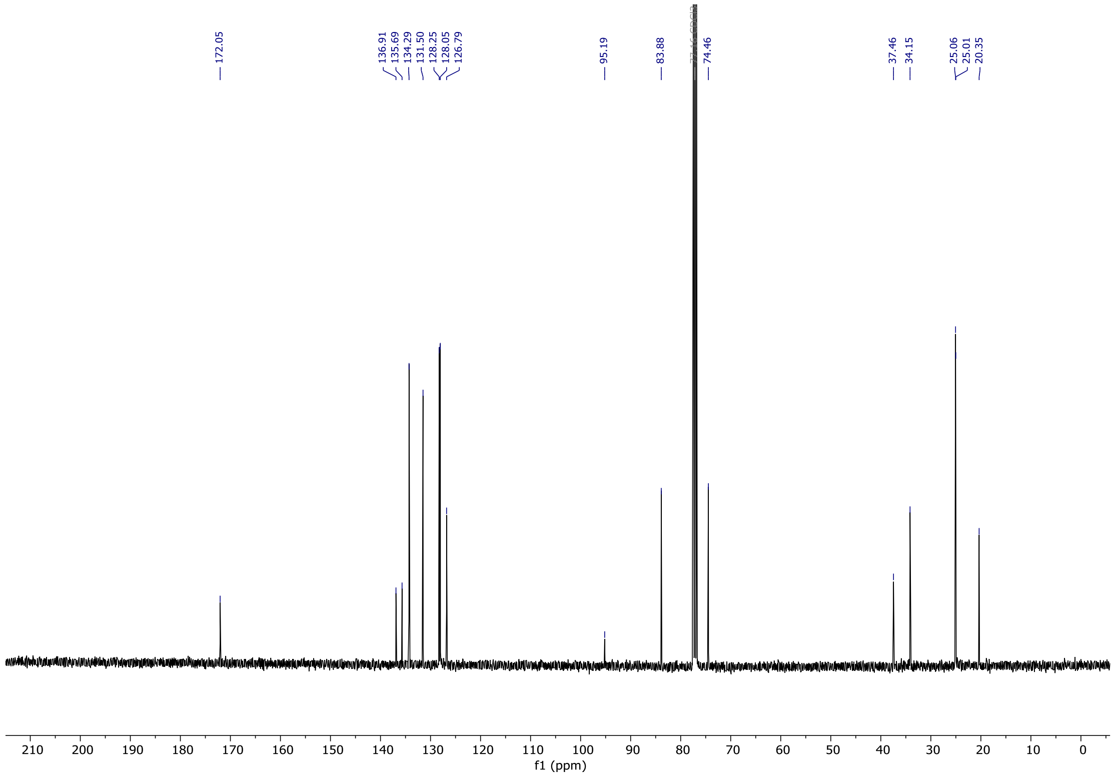

S11: ^{11}B NMR (128 MHz, $CDCl_3$):

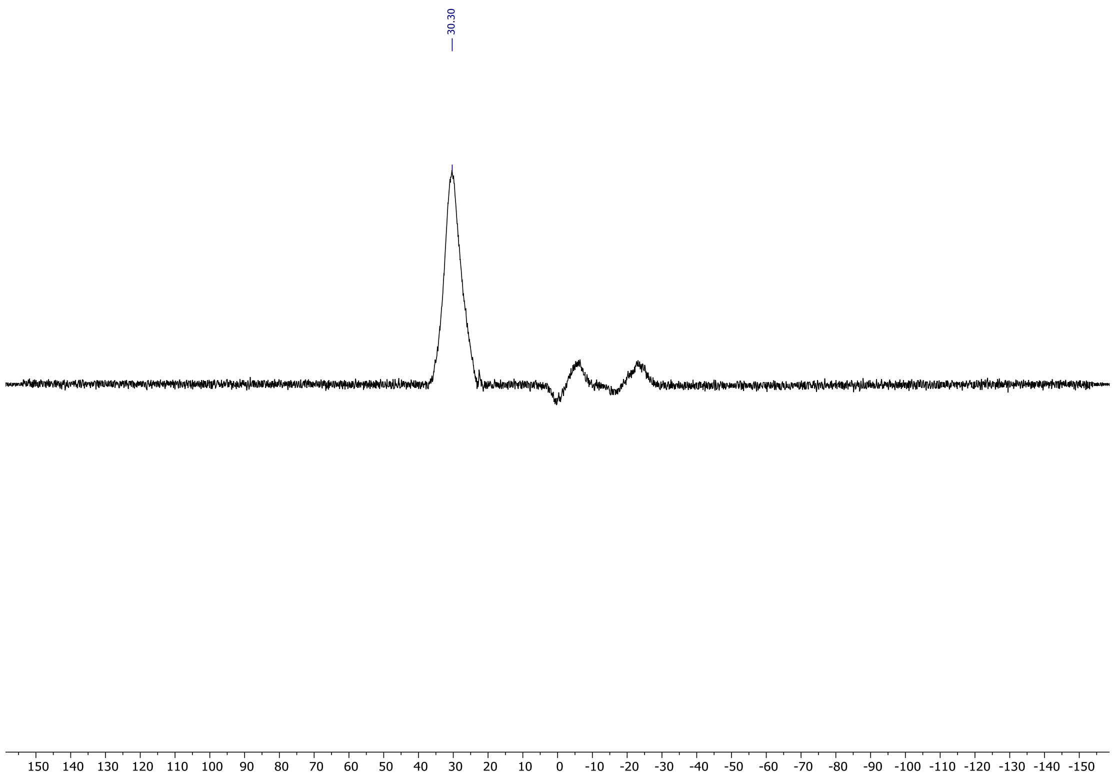

S11: HSQC NMR (400 MHz, 101 MHz, $CDCl_3$):

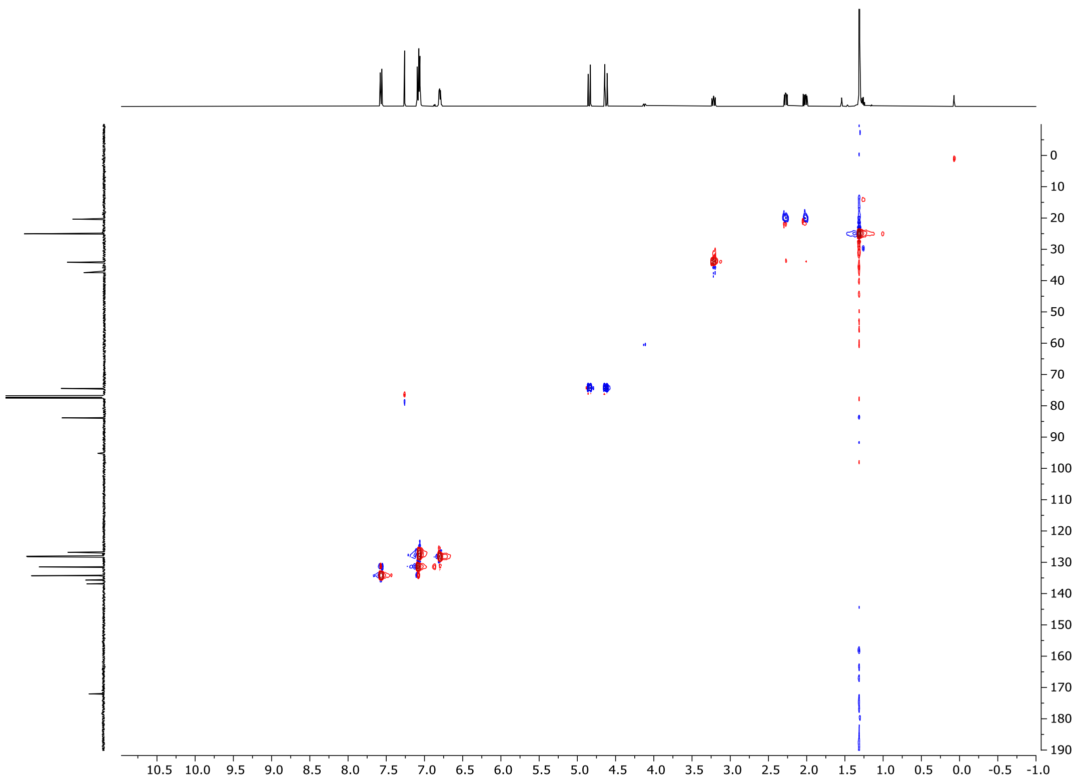

S11: HMBC NMR (400 MHz, 101 MHz, $CDCl_3$):

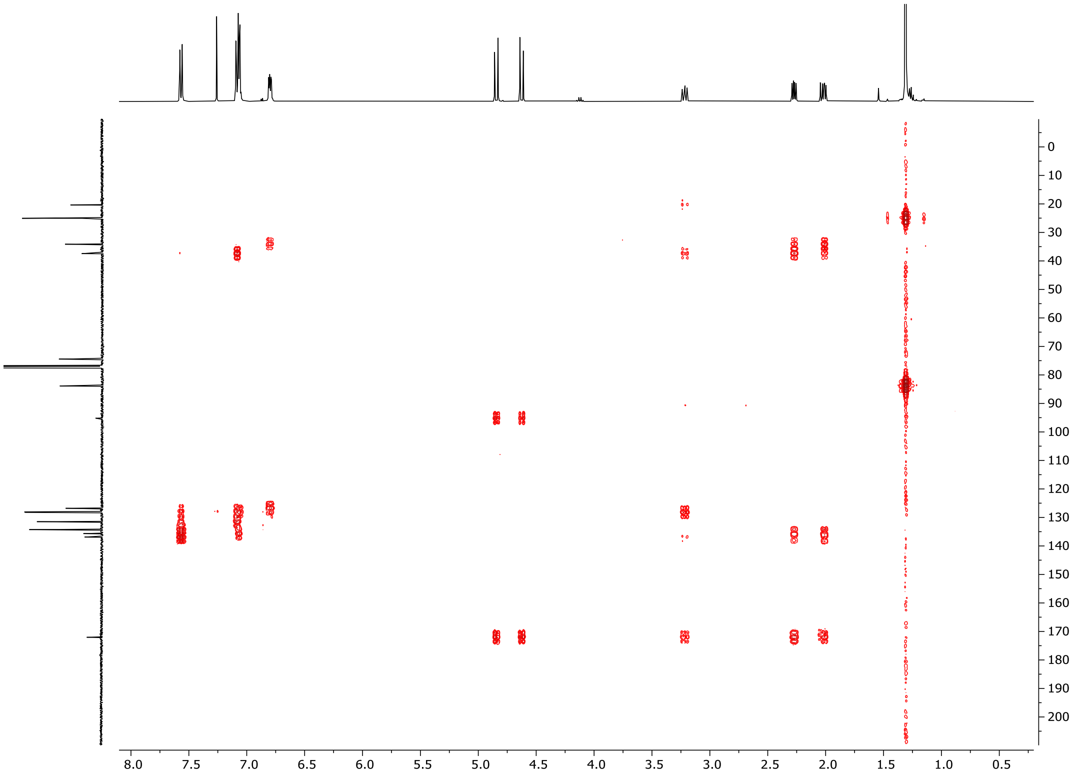

S11: NOESY NMR (400 MHz, $CDCl_3$):

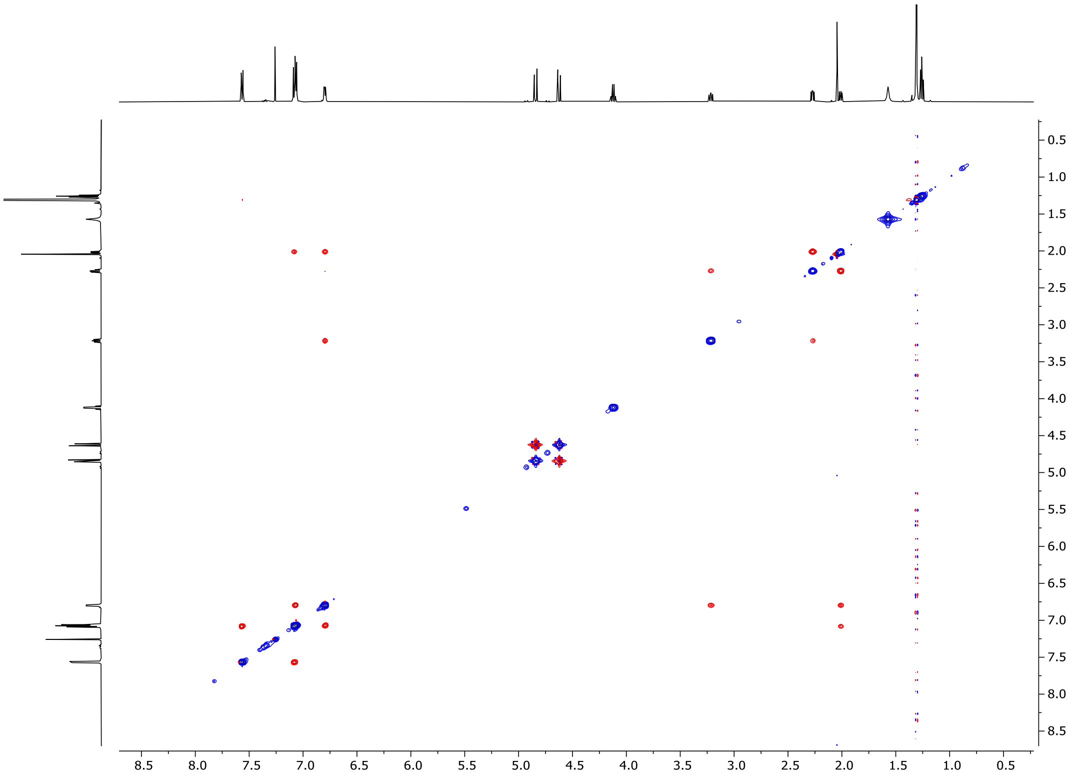

S12: ^{1}H NMR (400 MHz, CDCl$_3$):

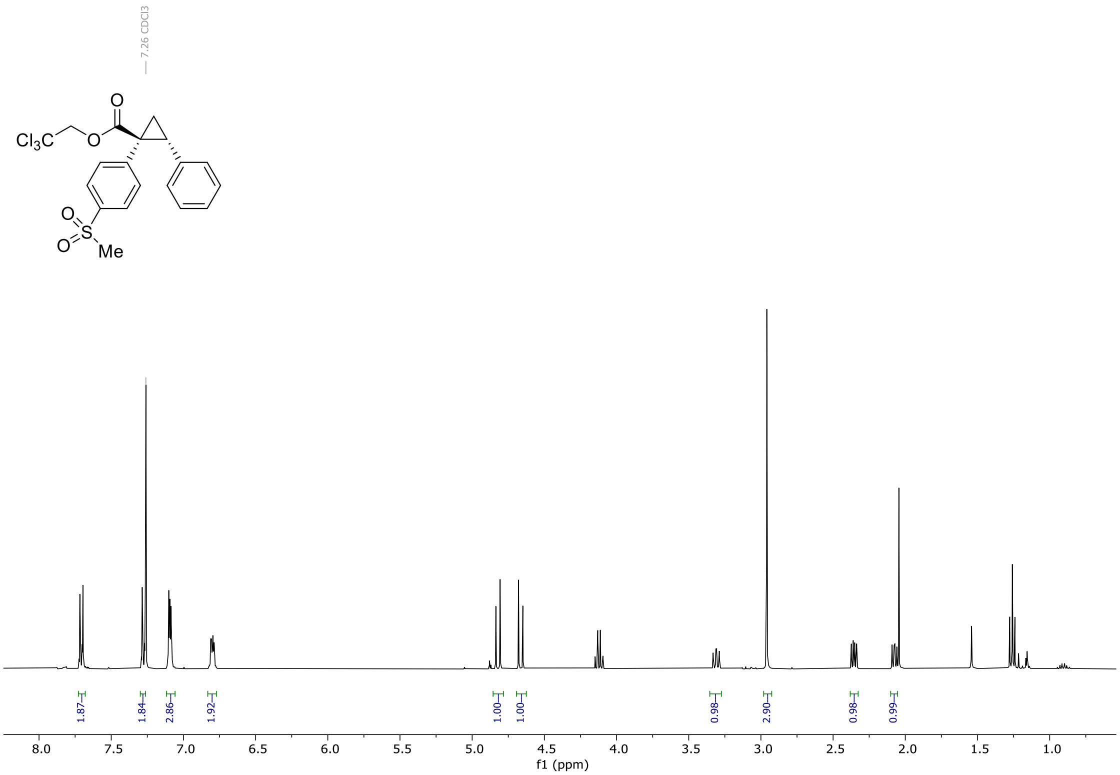

S12: ^{13}C NMR (101 MHz, CDCl$_3$):

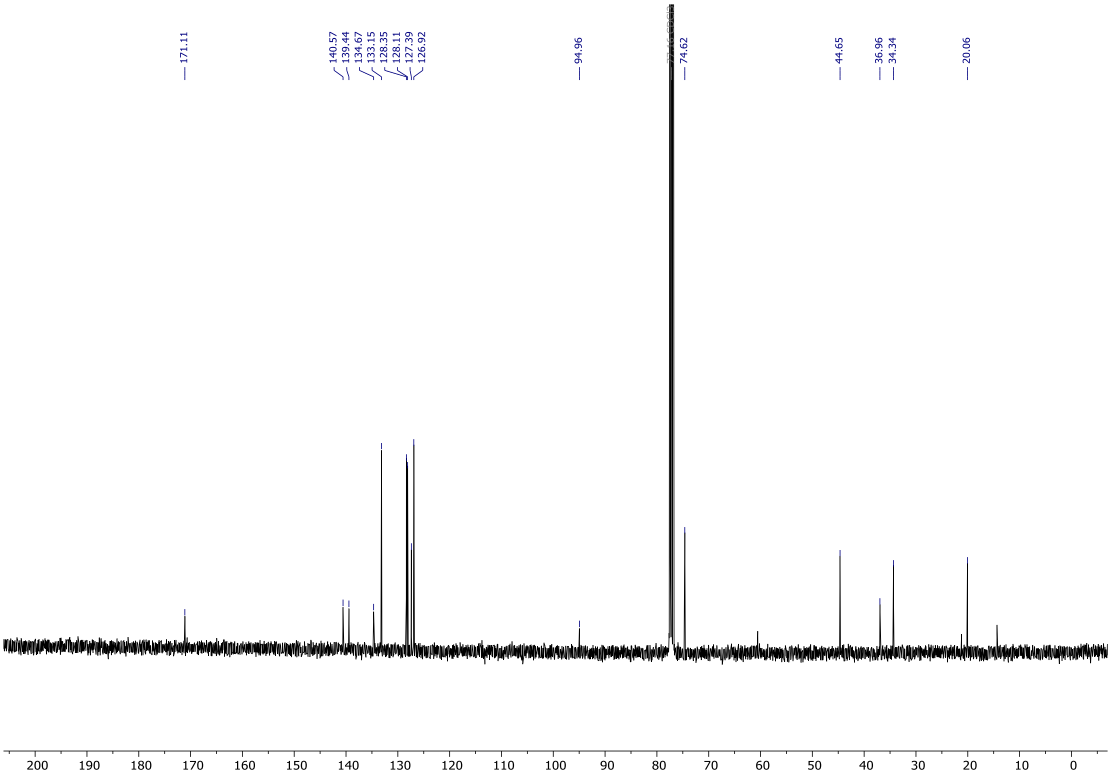

S13: ^{1}H NMR (400 MHz, $CDCl_3$):

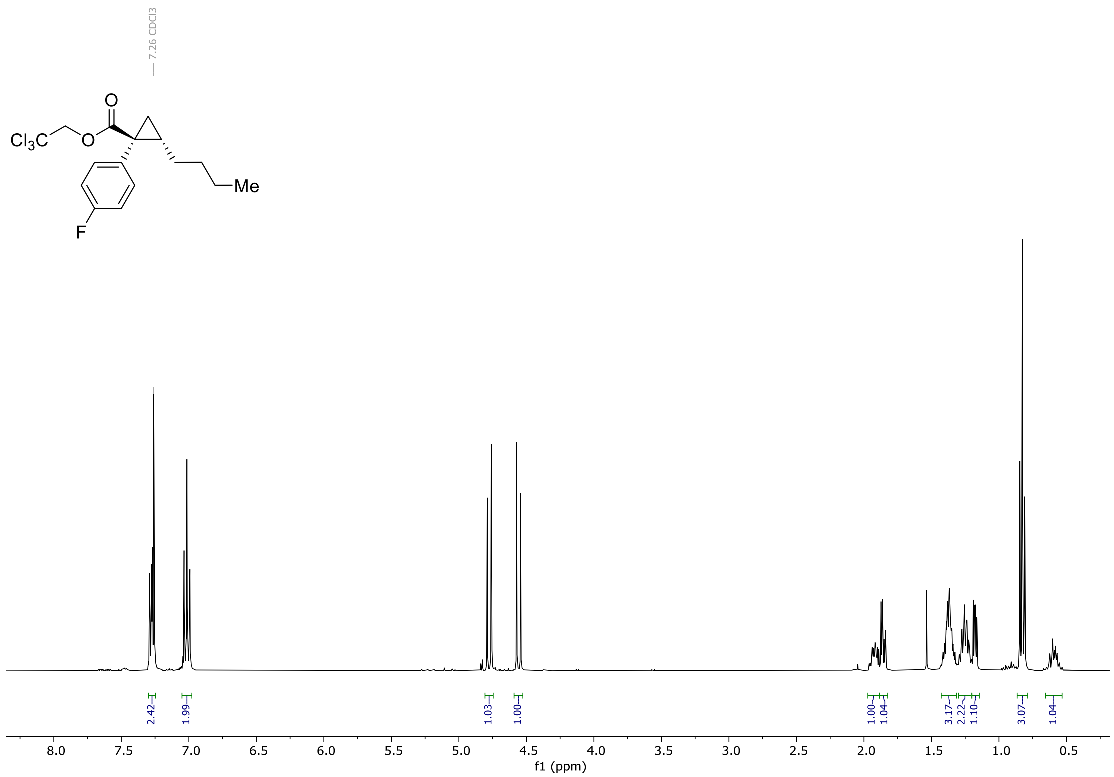

S13: ^{13}C NMR (101 MHz, $CDCl_3$):

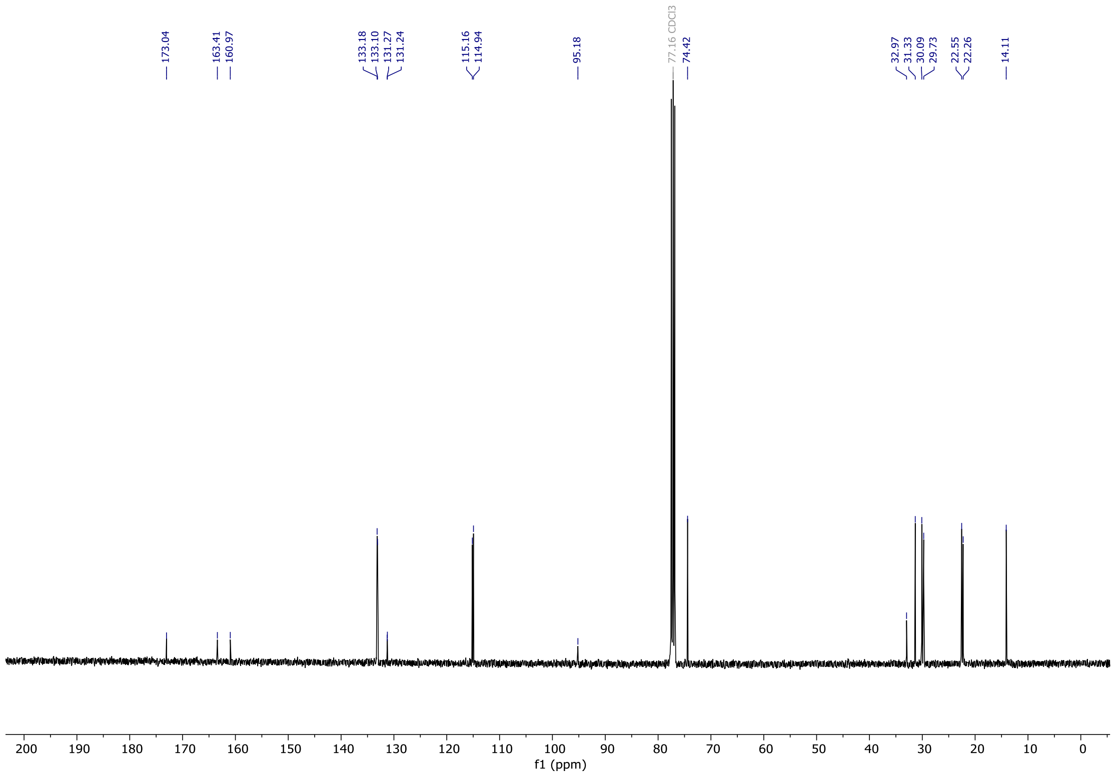

S13: ^{19}F NMR (282 MHz, $CDCl_3$):

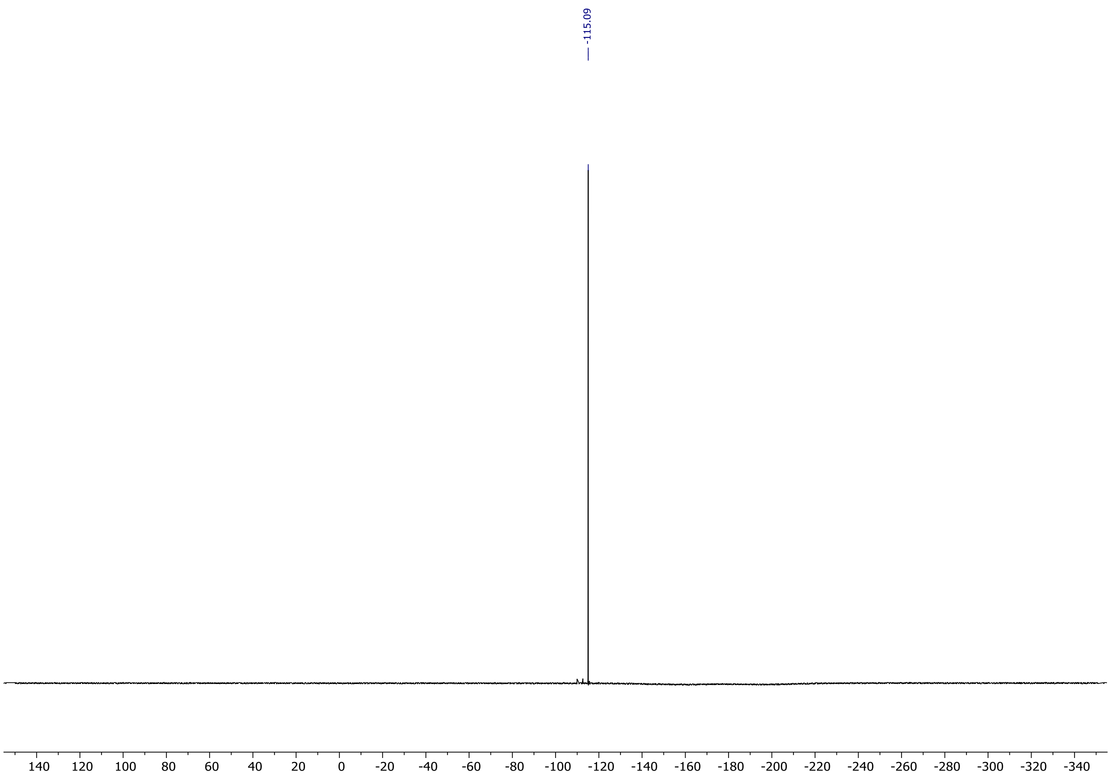

S14: ^{1}H NMR (400 MHz, $CDCl_3$):

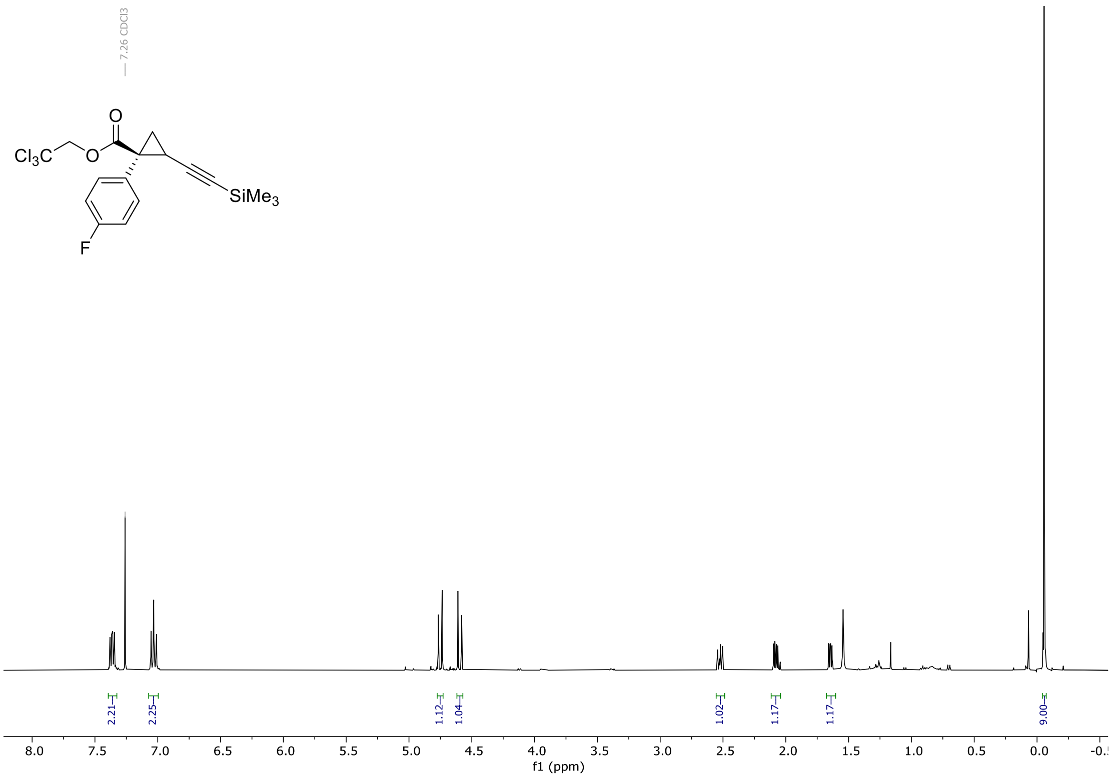

S14: ^{13}C NMR (101 MHz, $CDCl_3$):

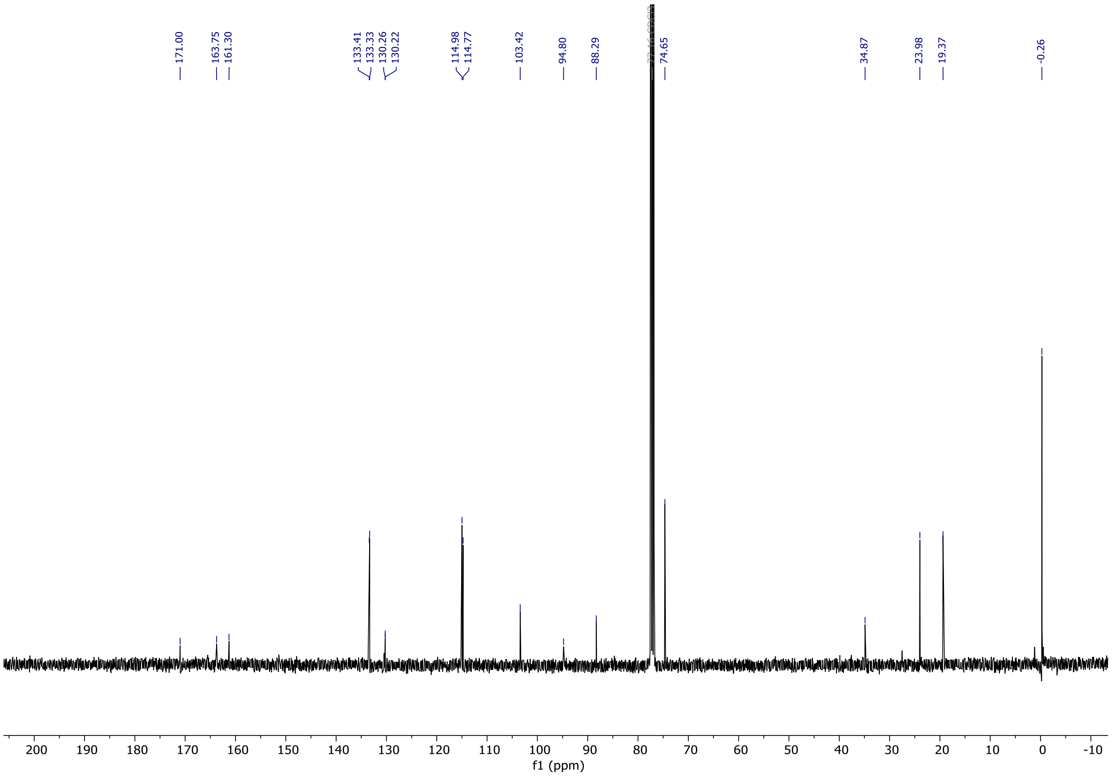

S14: ^{19}F NMR (282 MHz, $CDCl_3$):

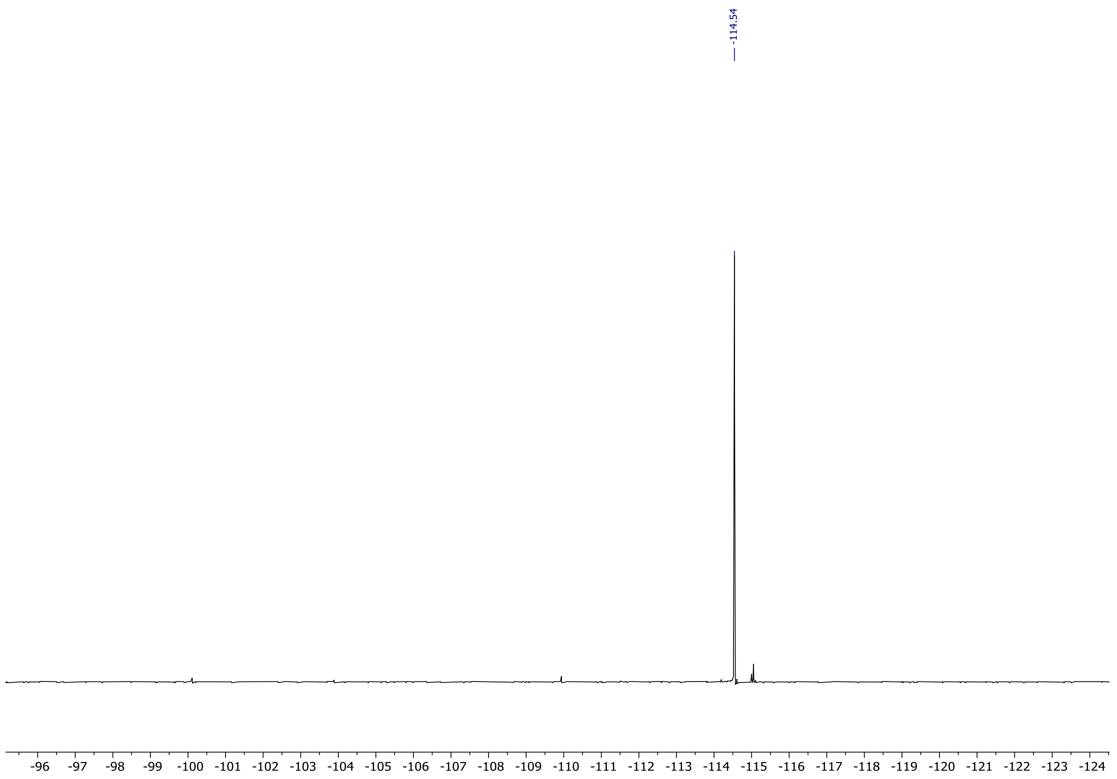

S14: ^{29}Si NMR (60 MHz, $CDCl_3$):

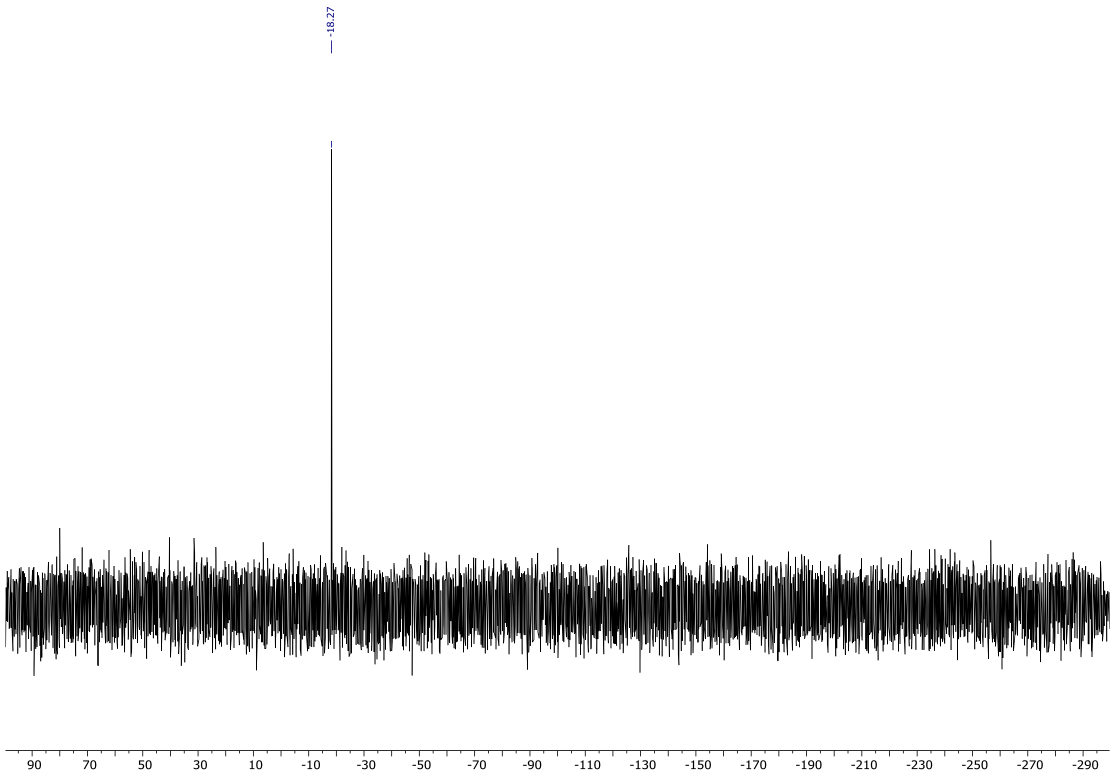

S15: ^{1}H NMR (400 MHz, $CDCl_3$):

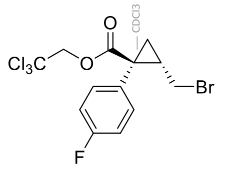

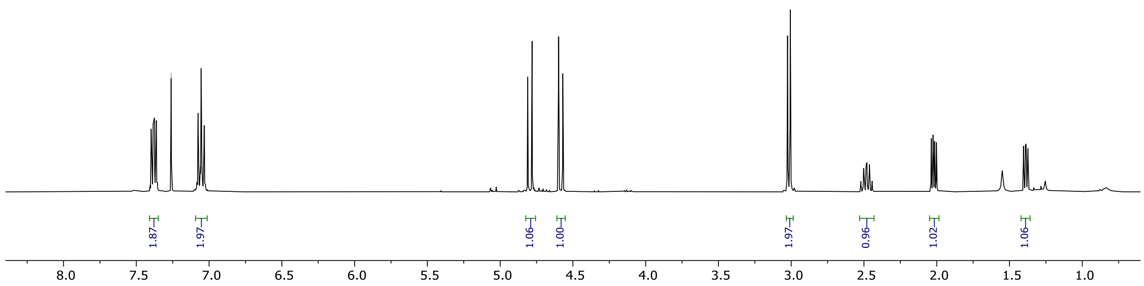

S15: ^{13}C NMR (101 MHz, $CDCl_3$):

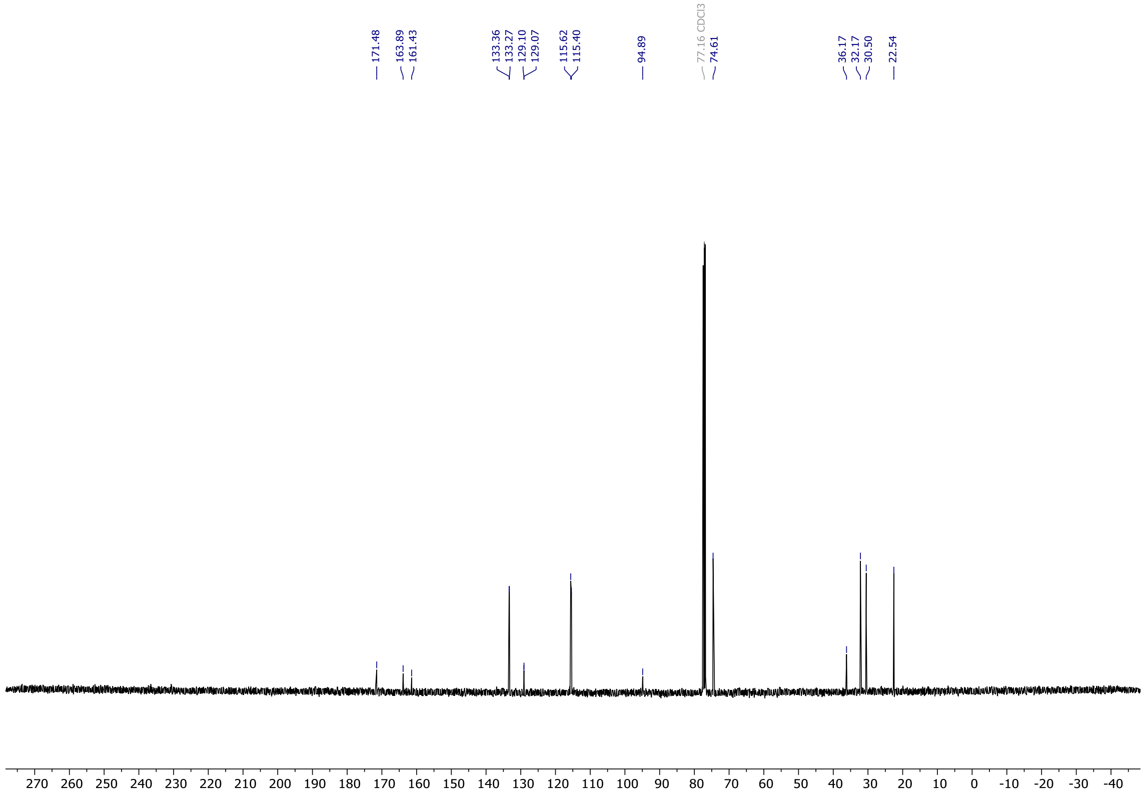

S15: ^{19}F NMR (282 MHz, $CDCl_3$):

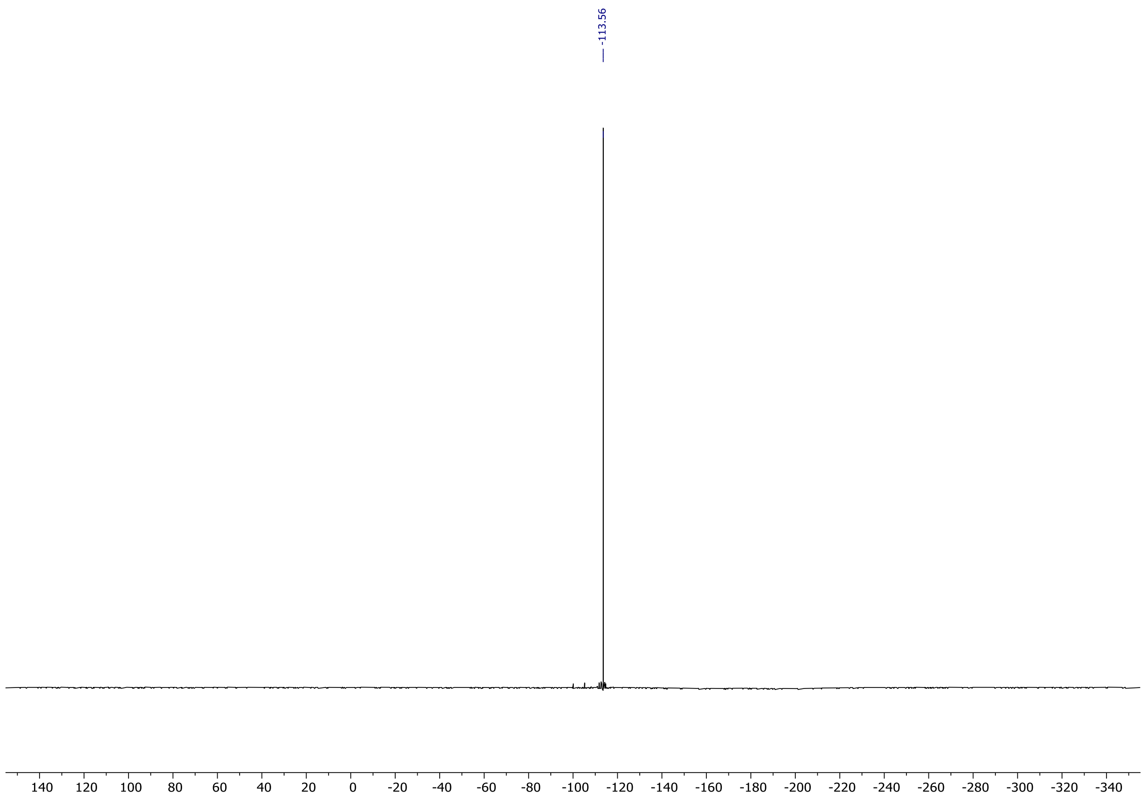

S16: ^{1}H NMR (400 MHz, $CDCl_3$):

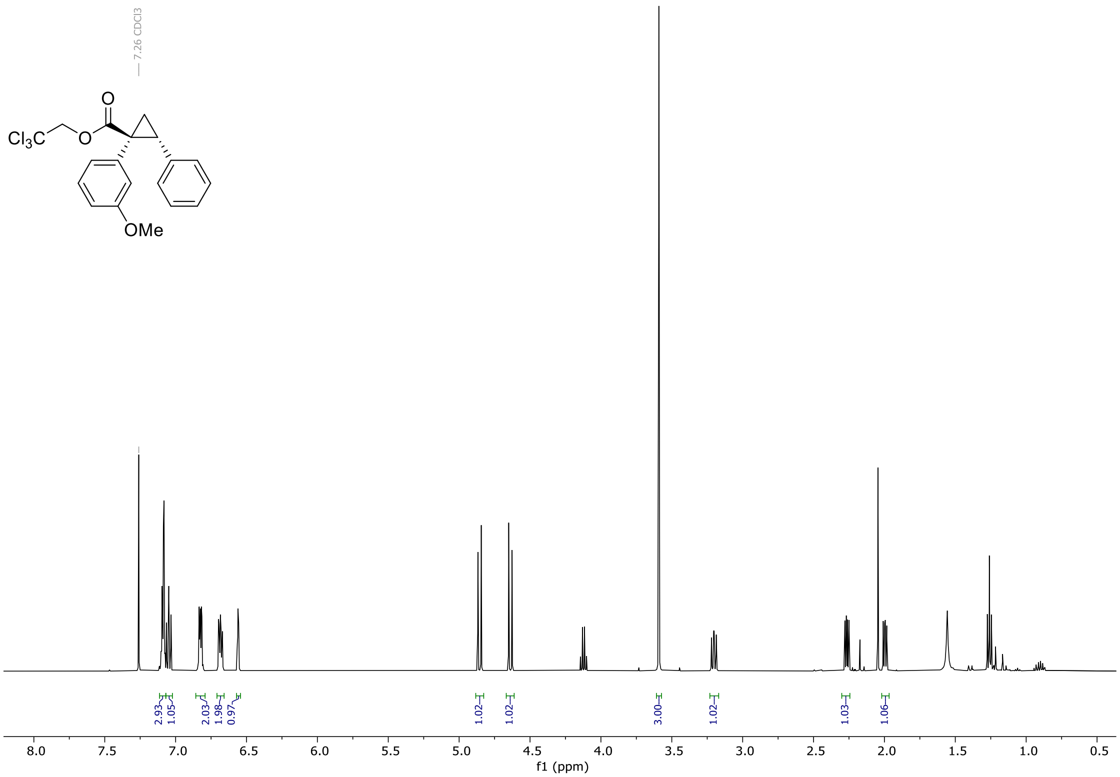

S16: ^{13}C NMR (101 MHz, $CDCl_3$):

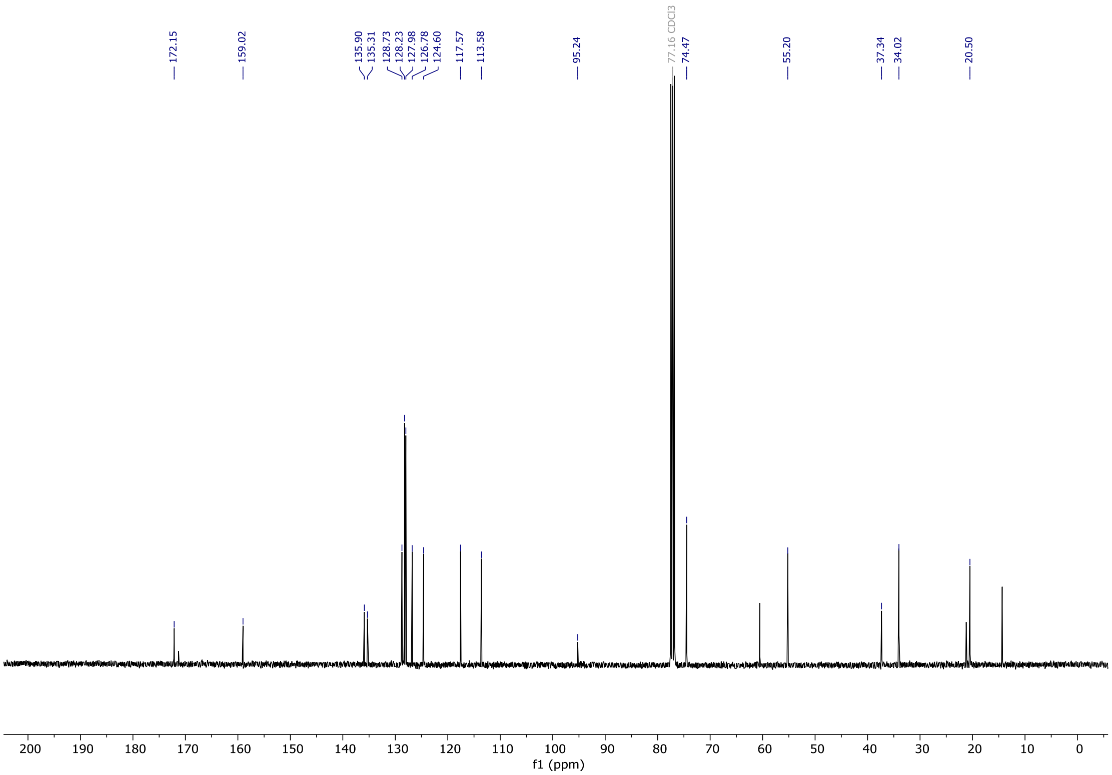

S16: HSQC NMR (400 MHz, 101 MHz, $CDCl_3$):

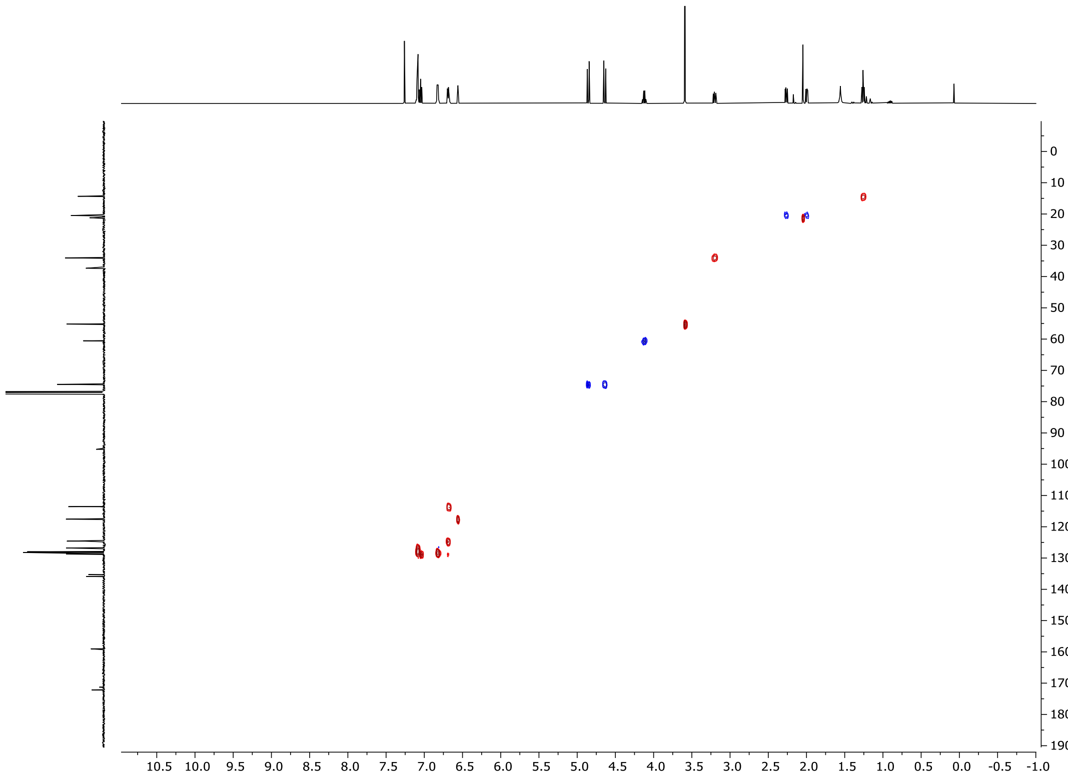

S16: HMBC NMR (400MHz, 101 MHz, $CDCl_3$):

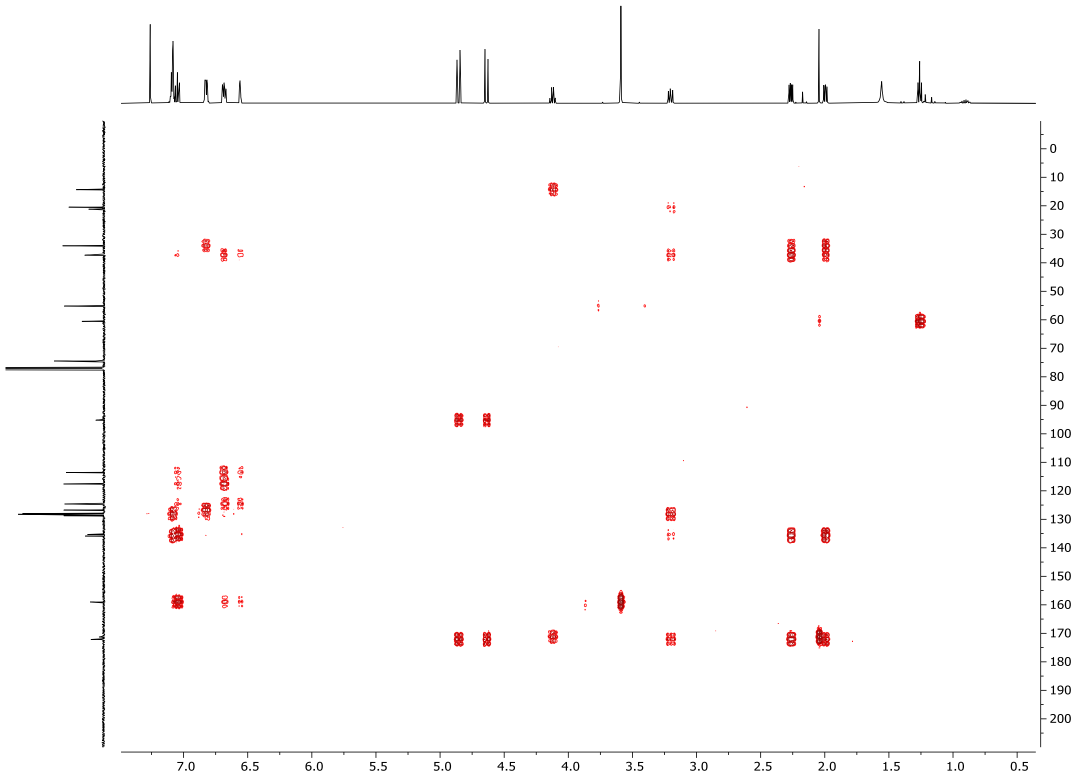

S16: NOESY NMR (500 MHz, $CDCl_3$):

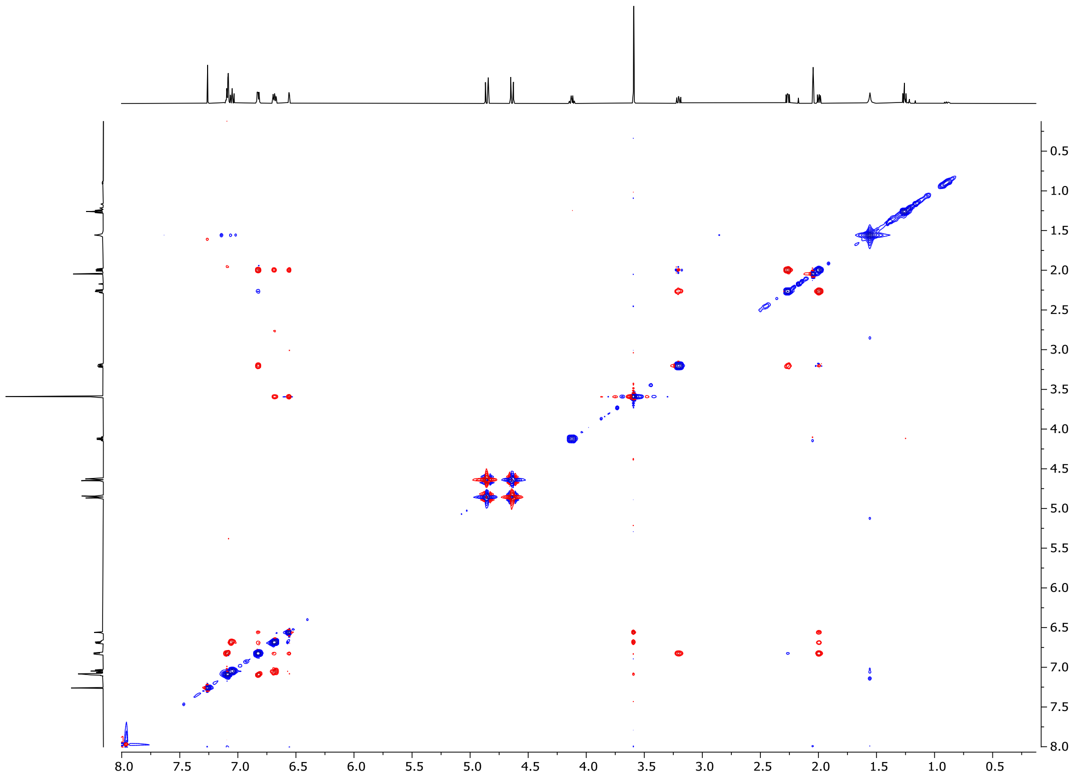

S17: ^{1}H NMR (400 MHz, $CDCl_3$):

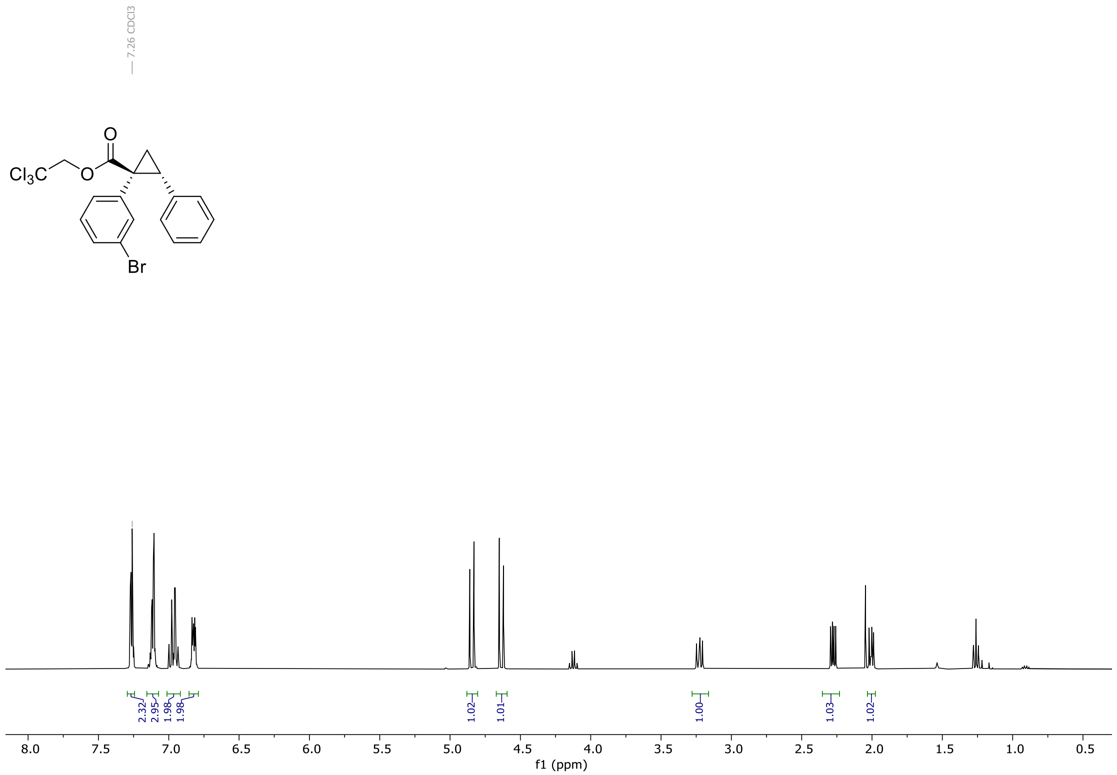

S17: ^{13}C NMR (101 MHz, $CDCl_3$):

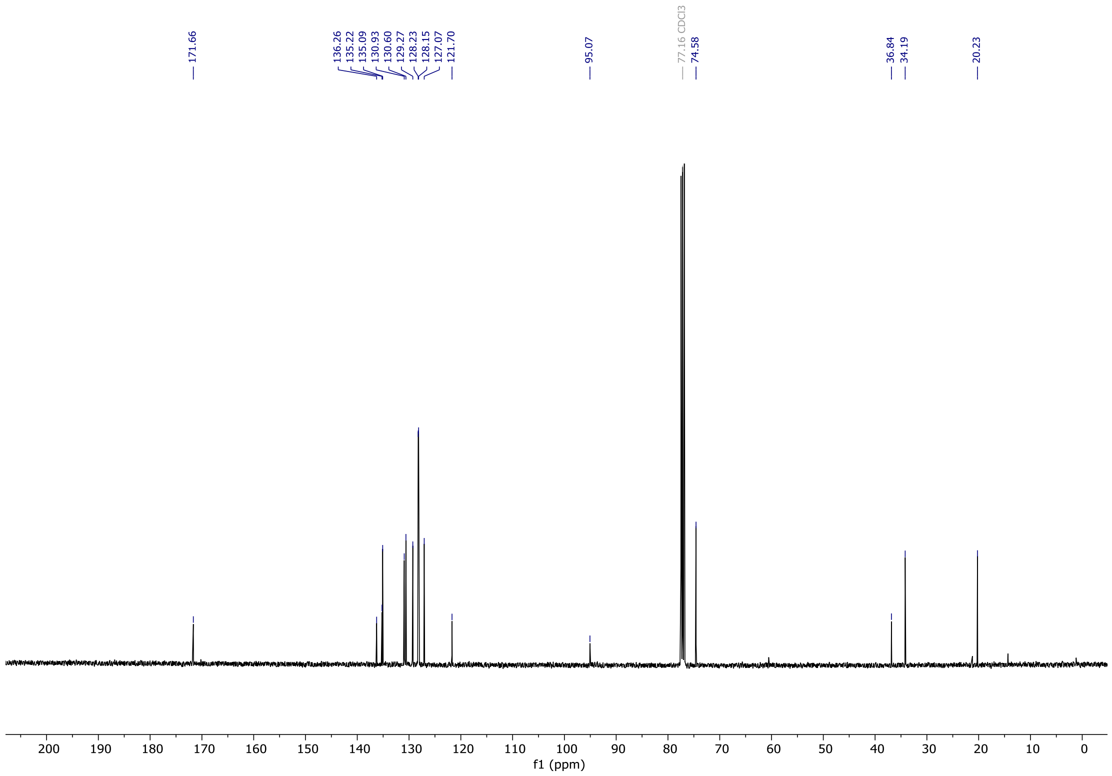

S18: ^{1}H NMR (400 MHz, $CDCl_3$):

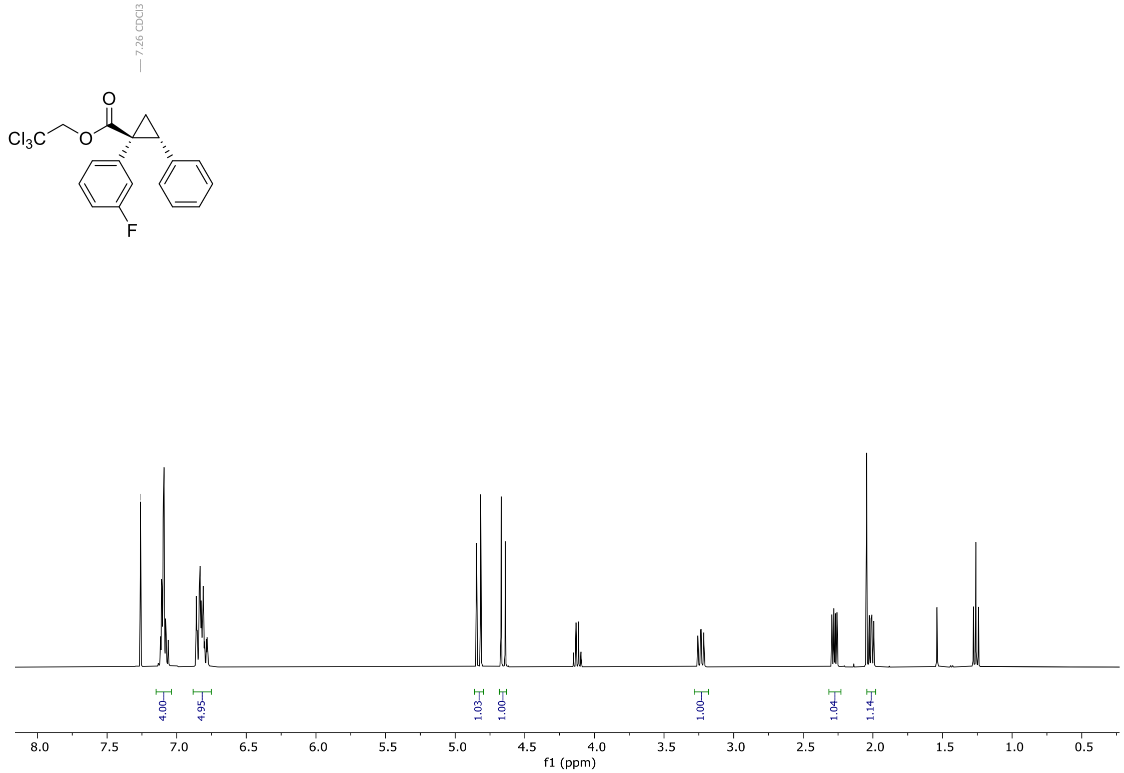

S18: ^{13}C NMR (101 MHz, $CDCl_3$):

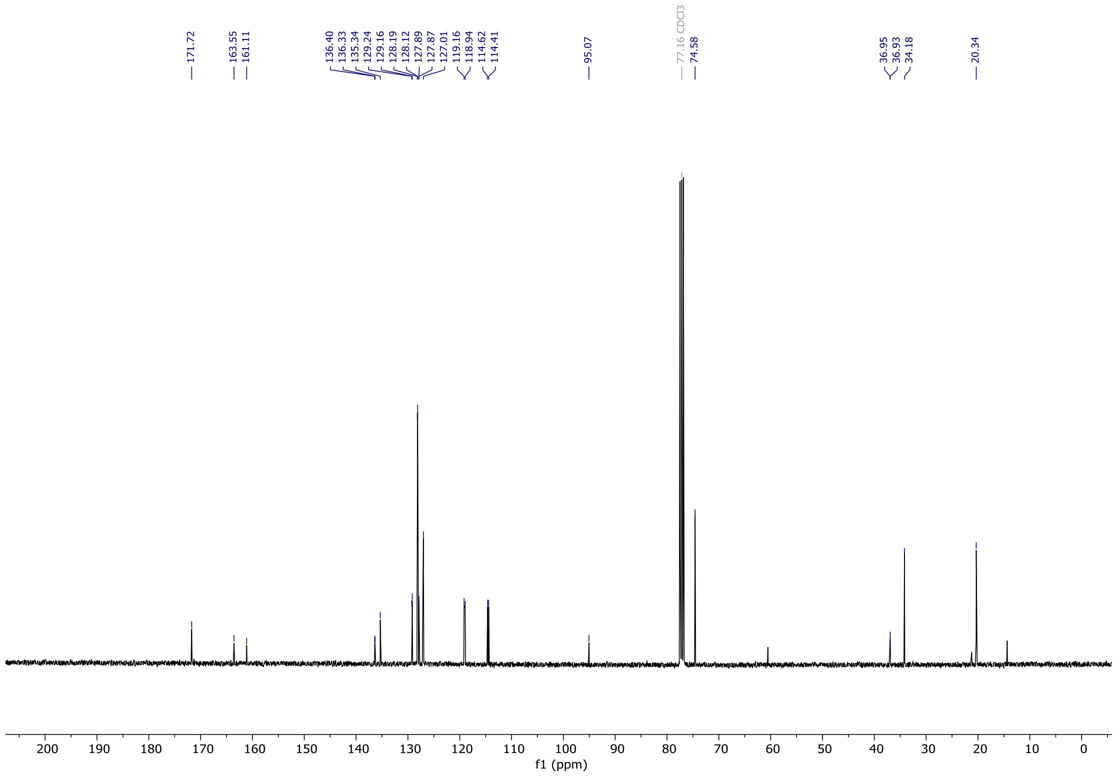

S18: ^{19}F NMR (470 MHz, $CDCl_3$):

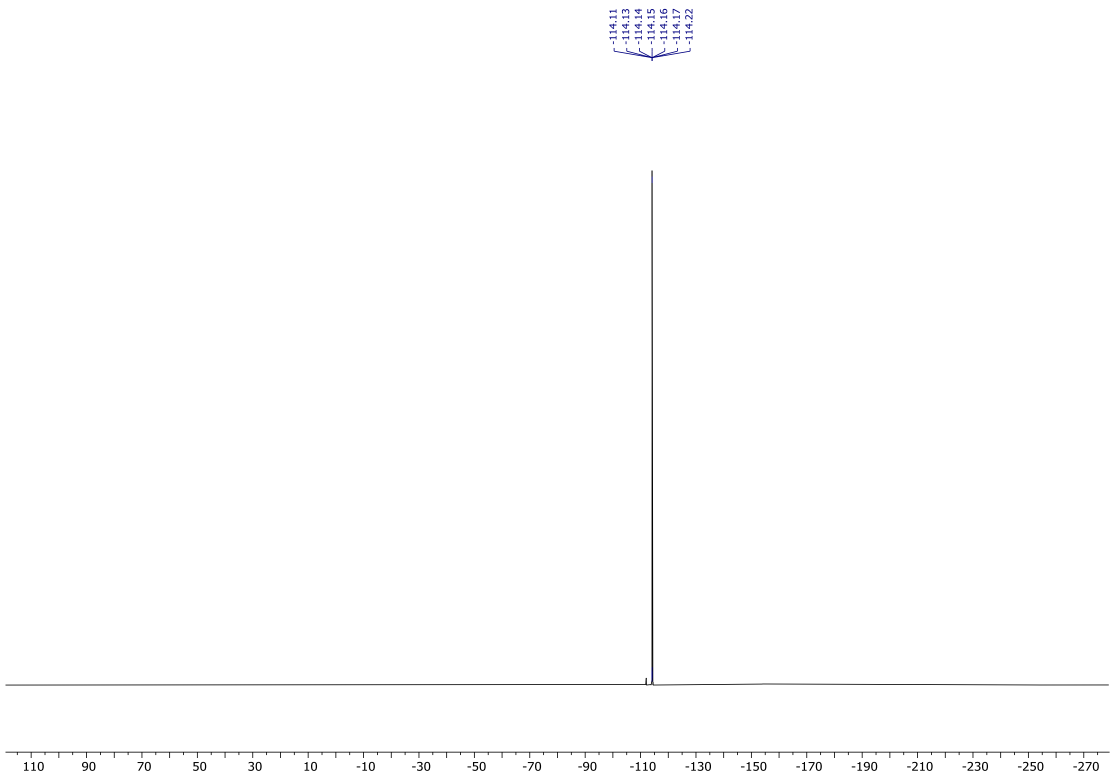

S18: HSQC NMR (400 MHz, 101 MHz, $CDCl_3$):

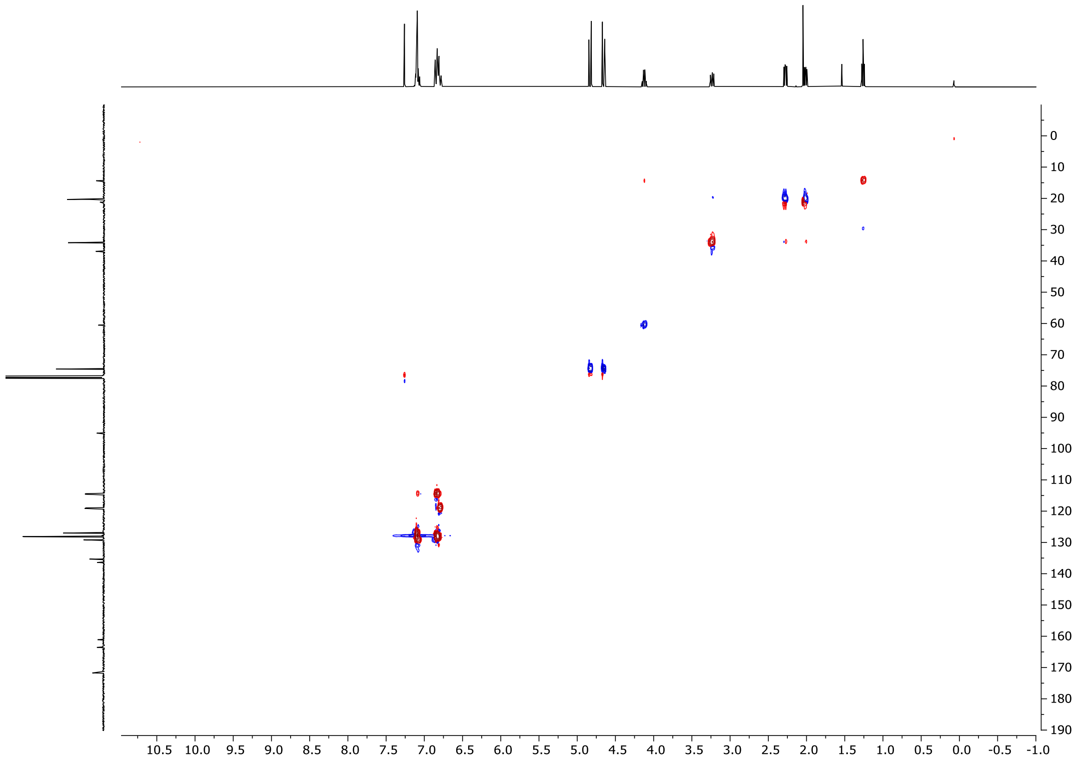

S18: HMBC NMR (400 MHz, 101 MHz, CDCl$_3$):

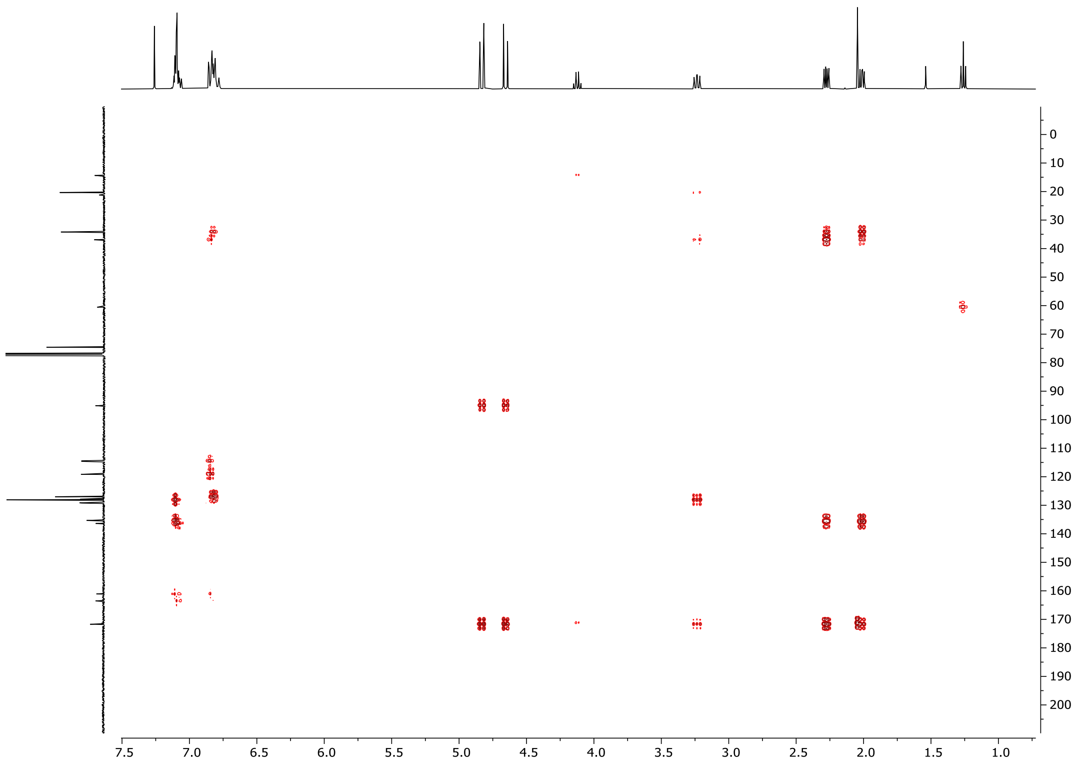

S18: NOESY NMR (500 MHz, CDCl$_3$):

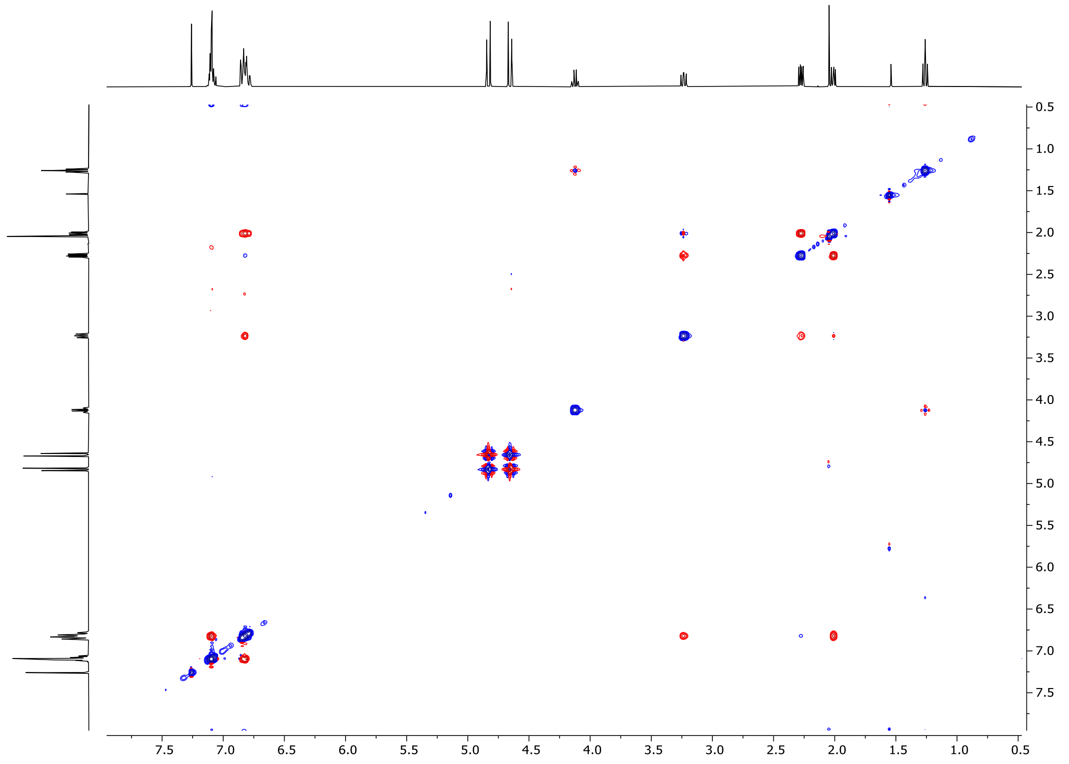

S19: ^{1}H NMR (400 MHz, $CDCl_3$):

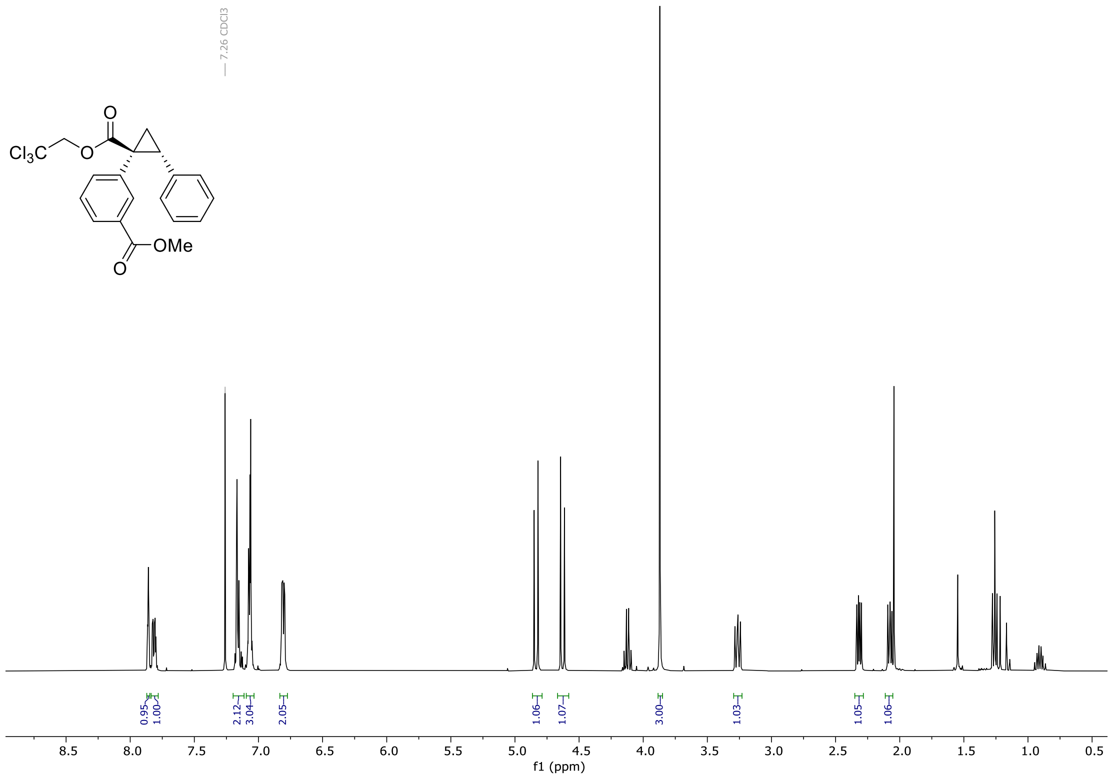

S19: ^{13}C NMR (101 MHz, $CDCl_3$):

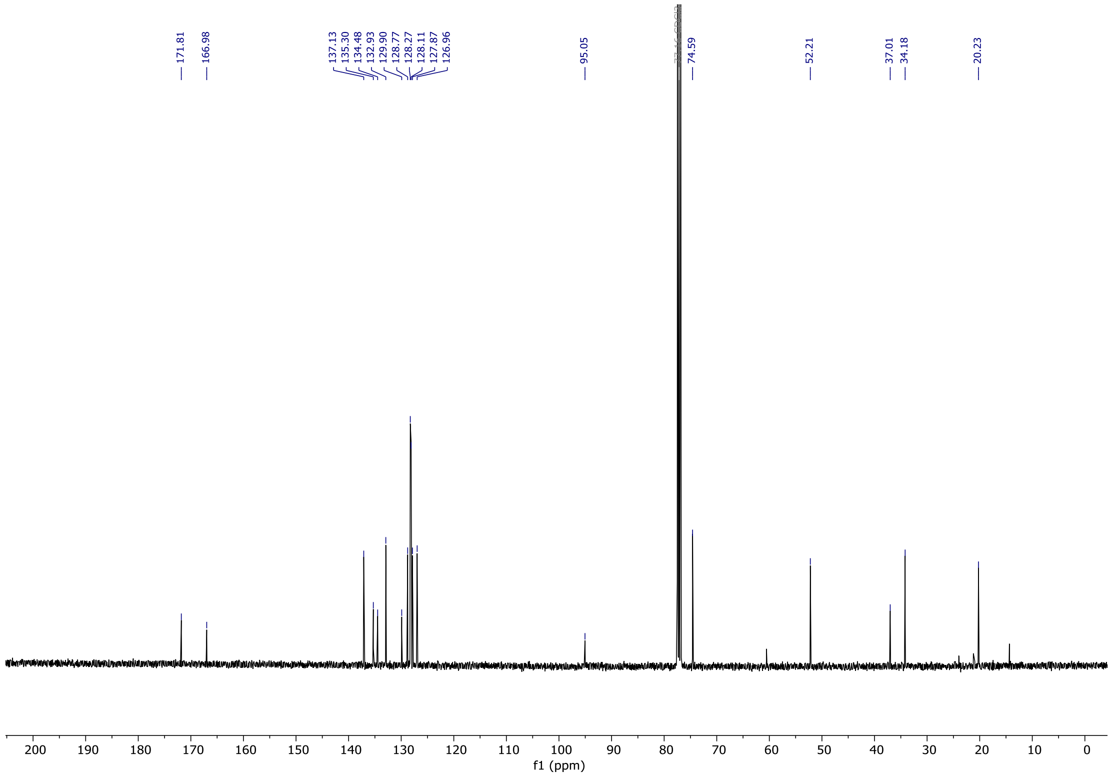

S20: ^{1}H NMR (400 MHz, $CDCl_3$):

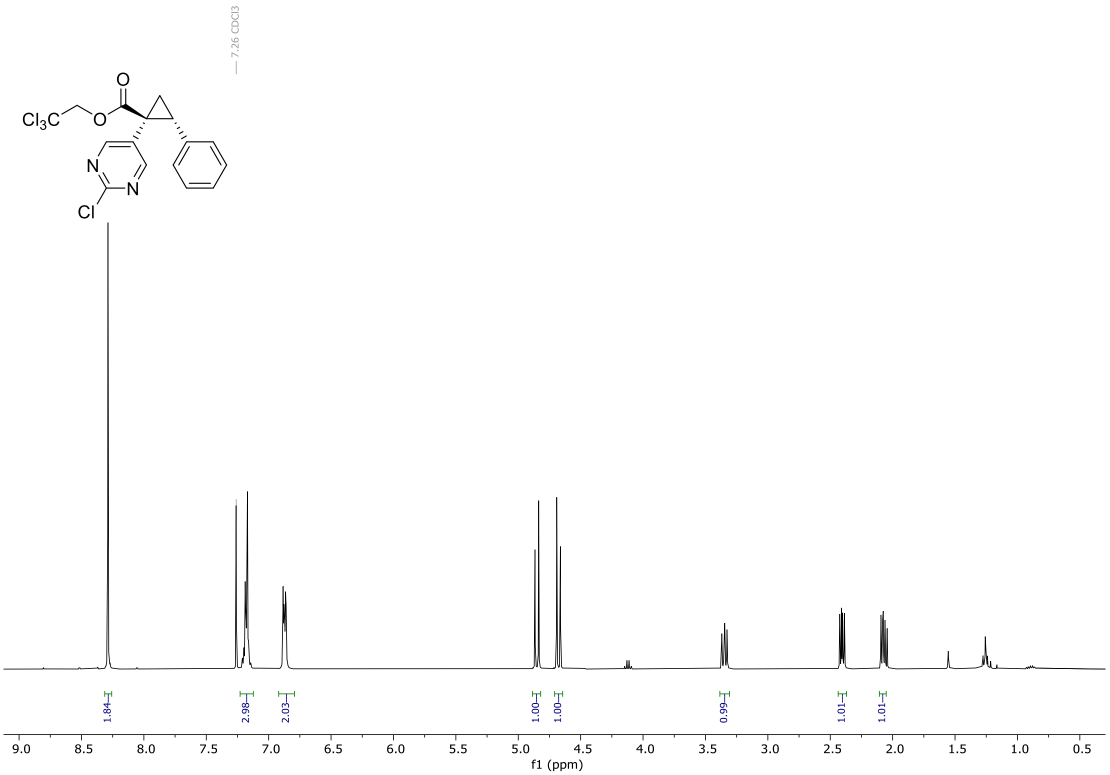

S20: ^{13}C NMR (101 MHz, $CDCl_3$):

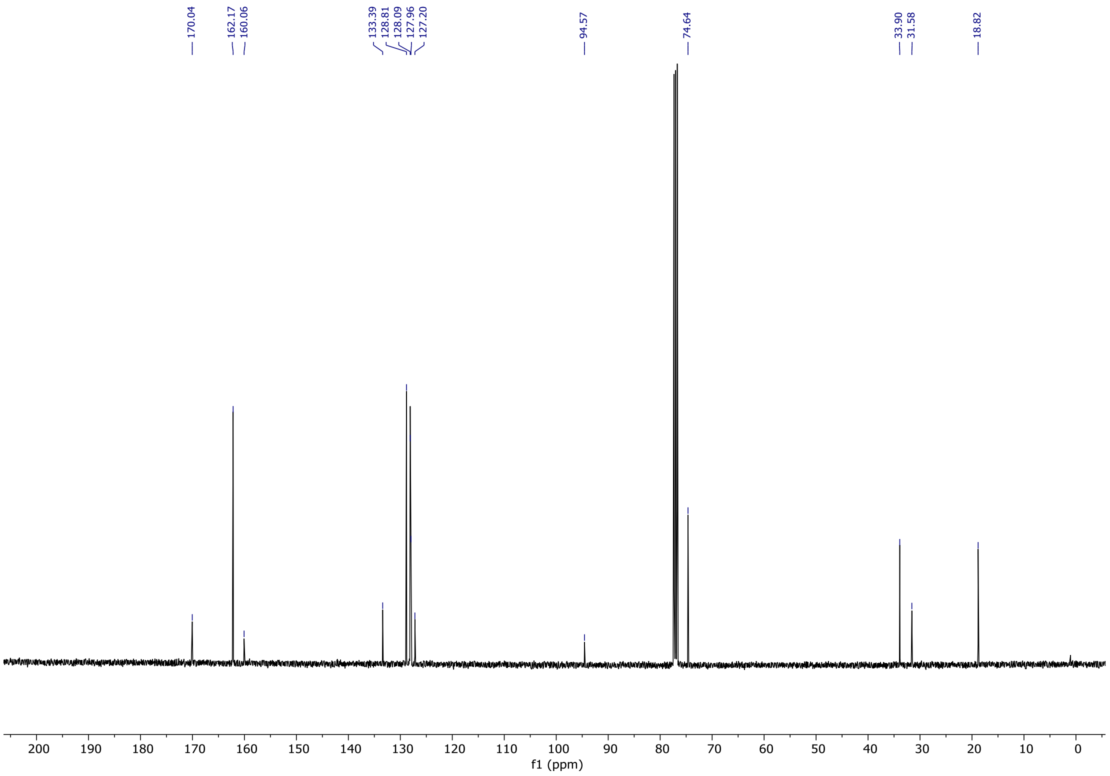

S21: ^{1}H NMR (400 MHz, $CDCl_3$):

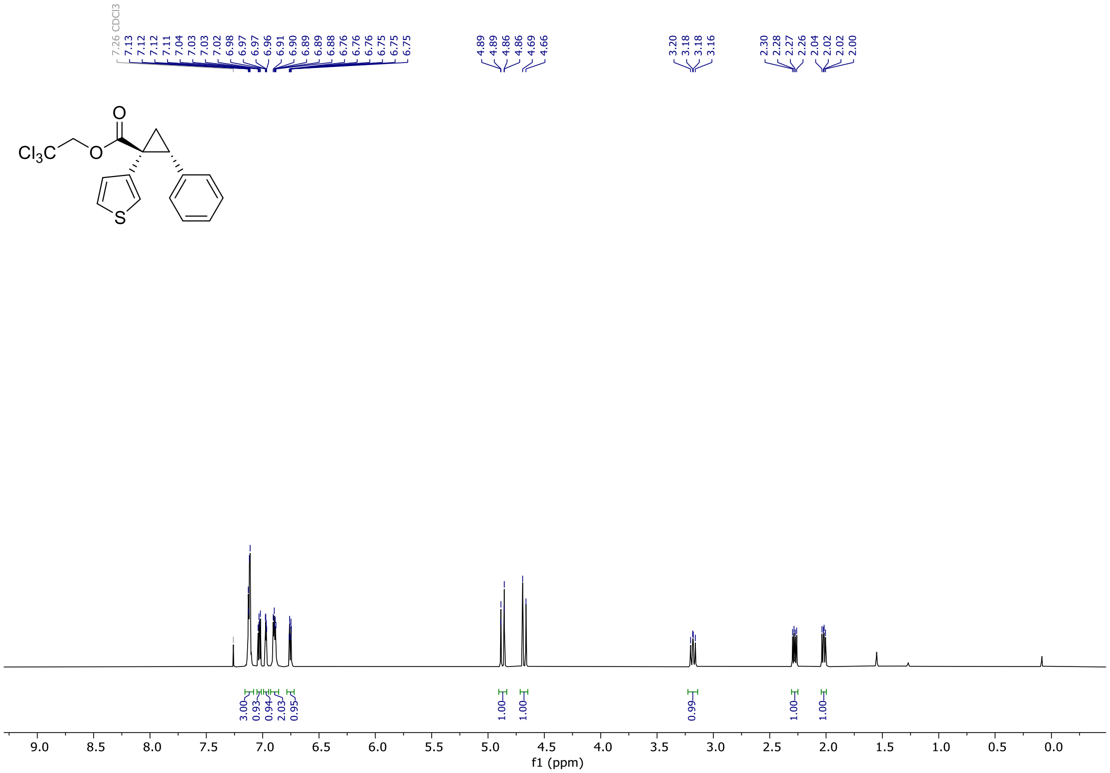

S21: ^{13}C NMR (101 MHz, $CDCl_3$):

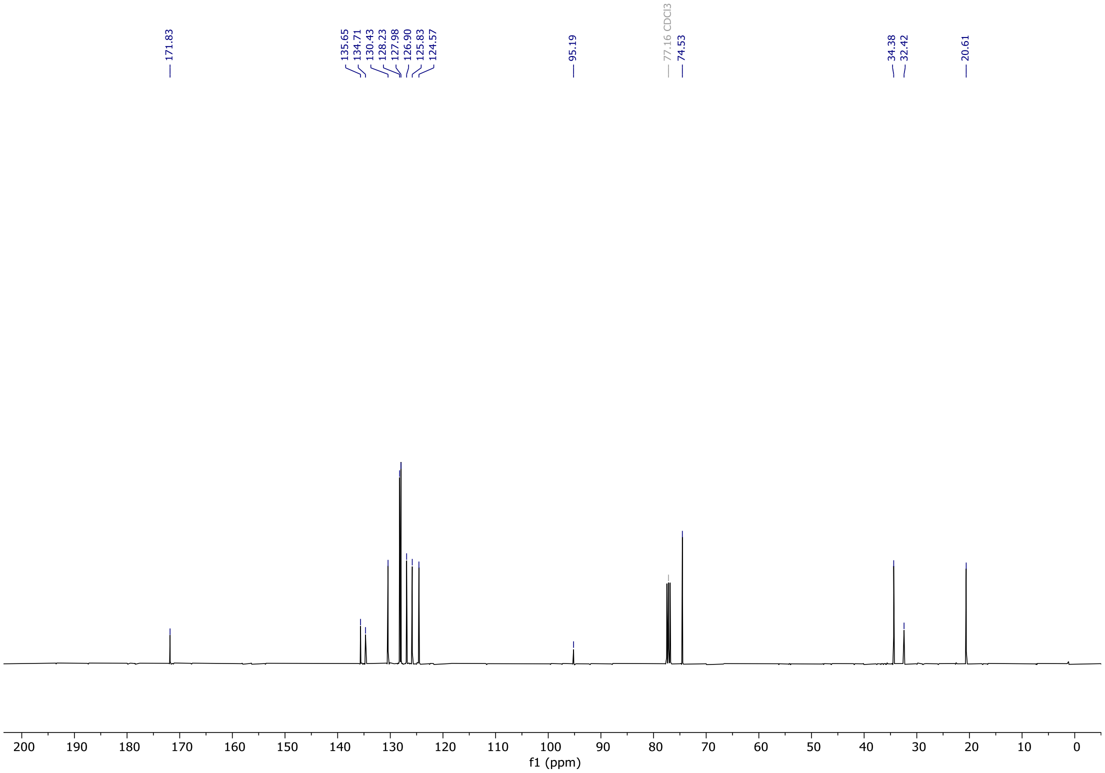

S22: ^{1}H NMR (400 MHz, CDCl$_3$):

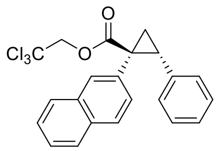

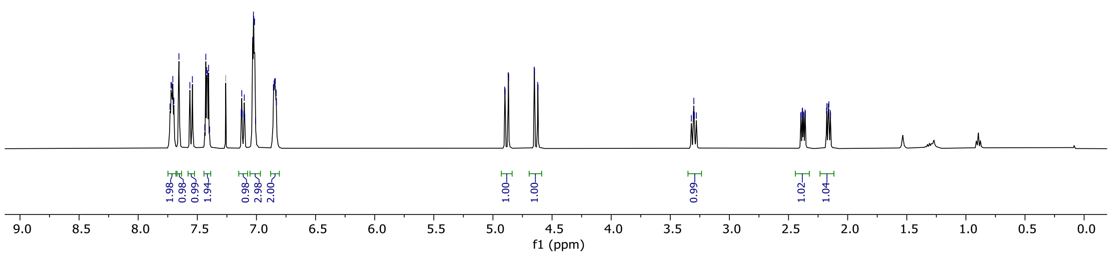

S22: ^{13}C NMR (101 MHz, CDCl$_3$):

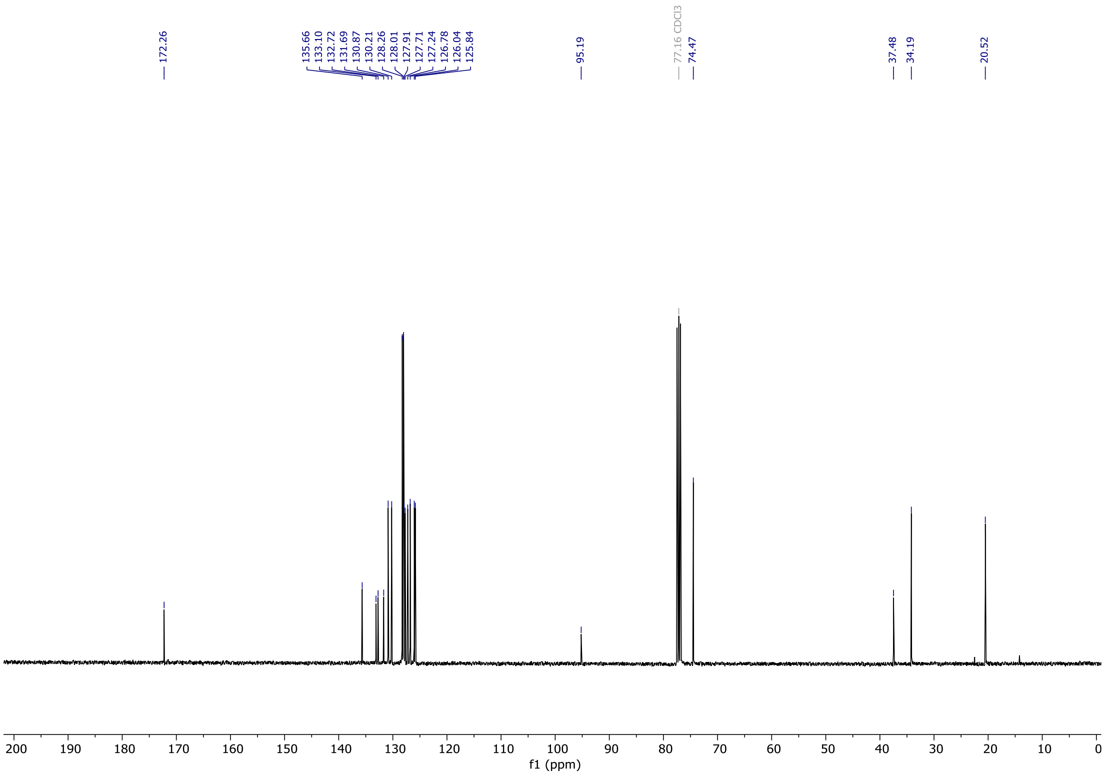

S23: ^{1}H NMR (400 MHz, $CDCl_3$):

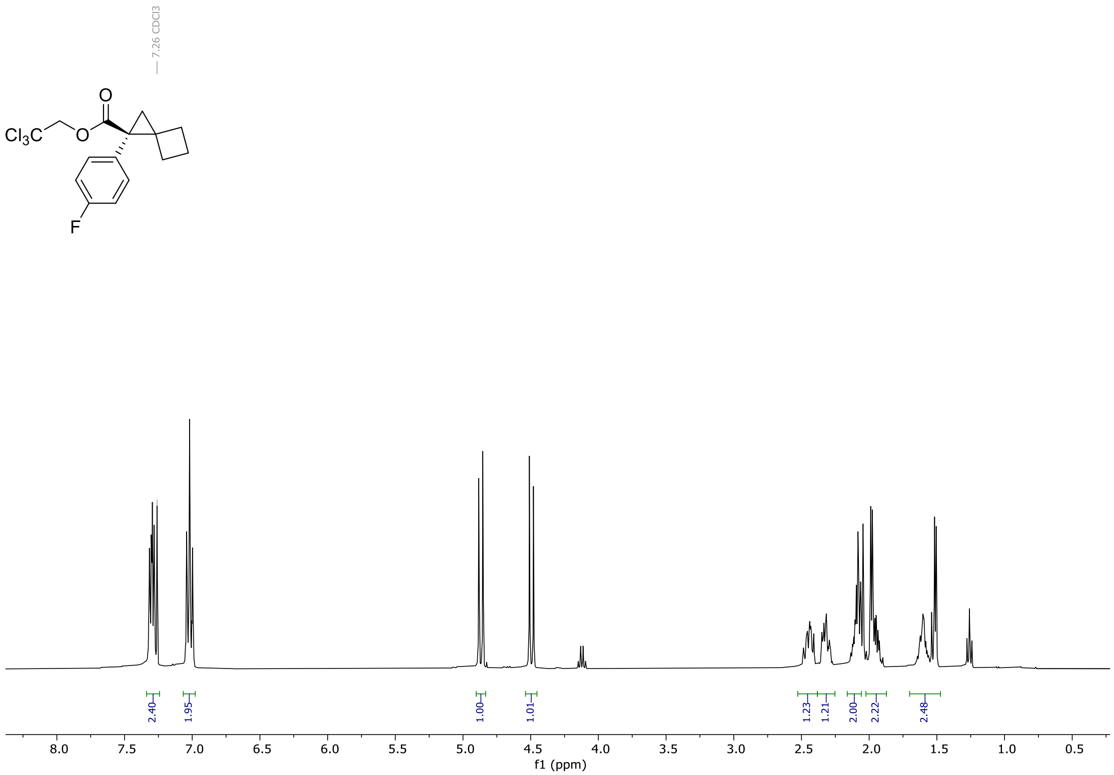

S23: ^{13}C NMR (101 MHz, $CDCl_3$):

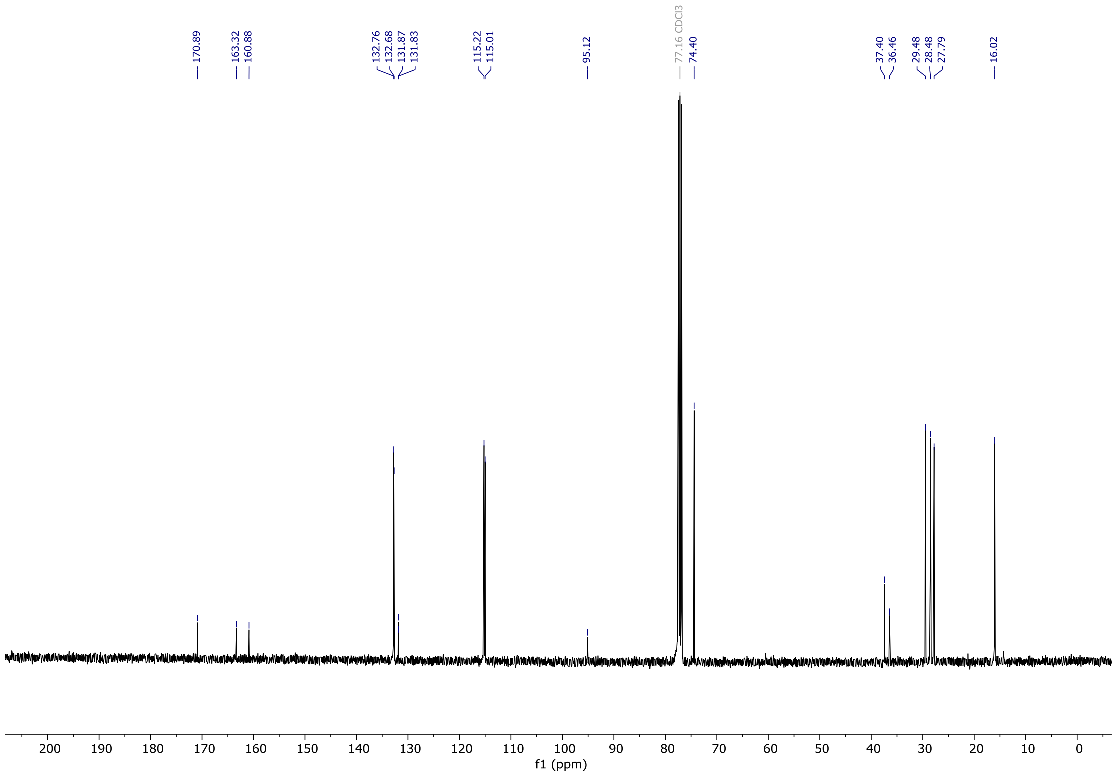

S23: ^{19}F NMR (282 MHz, $CDCl_3$):

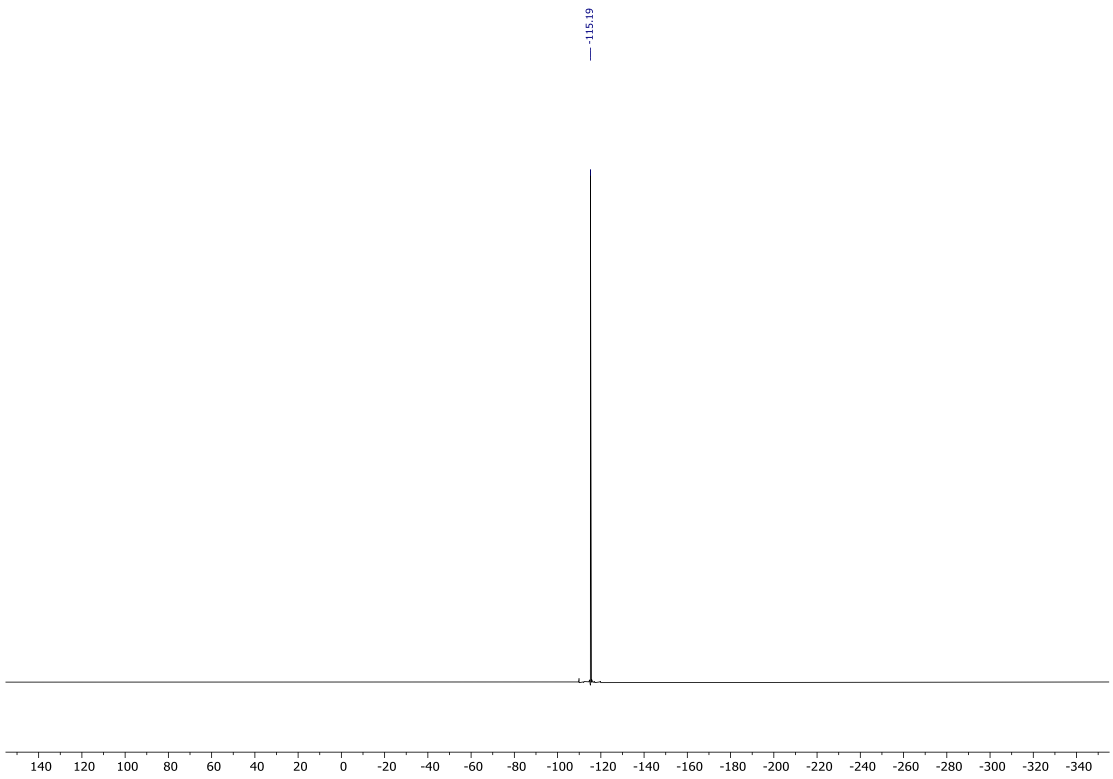

S24: ^{1}H NMR (400 MHz, CDCl3):

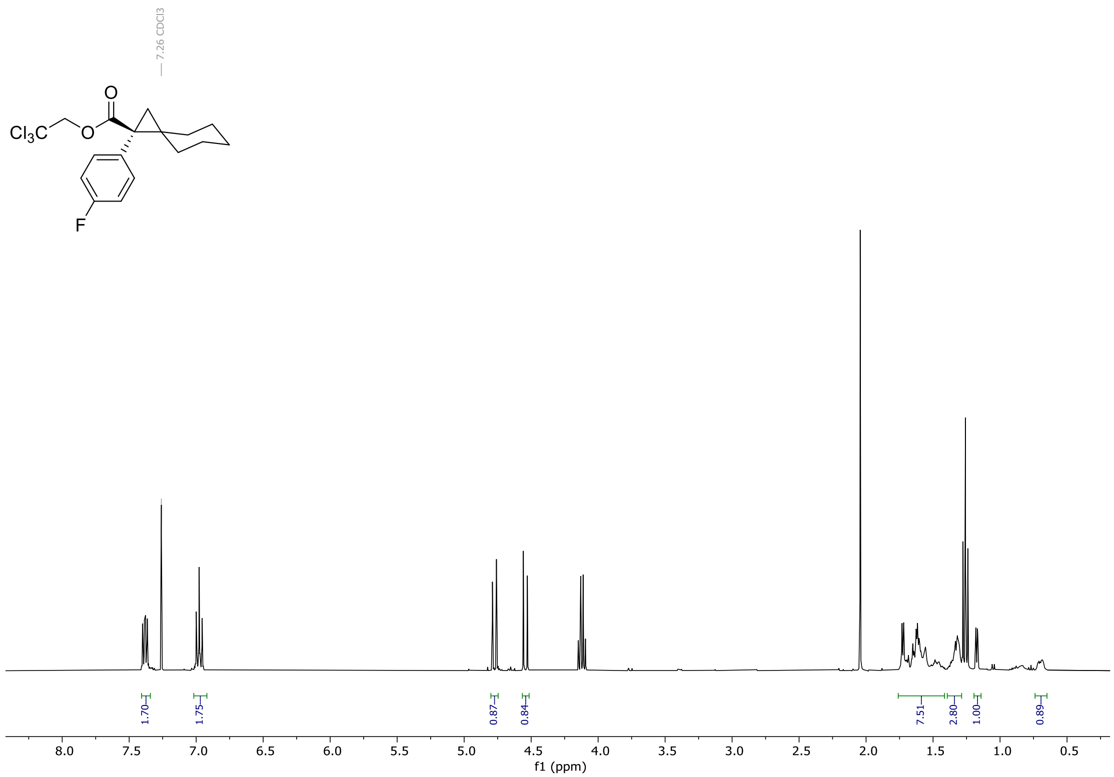

S24: ^{13}C NMR (400 MHz, $CDCl_3$):

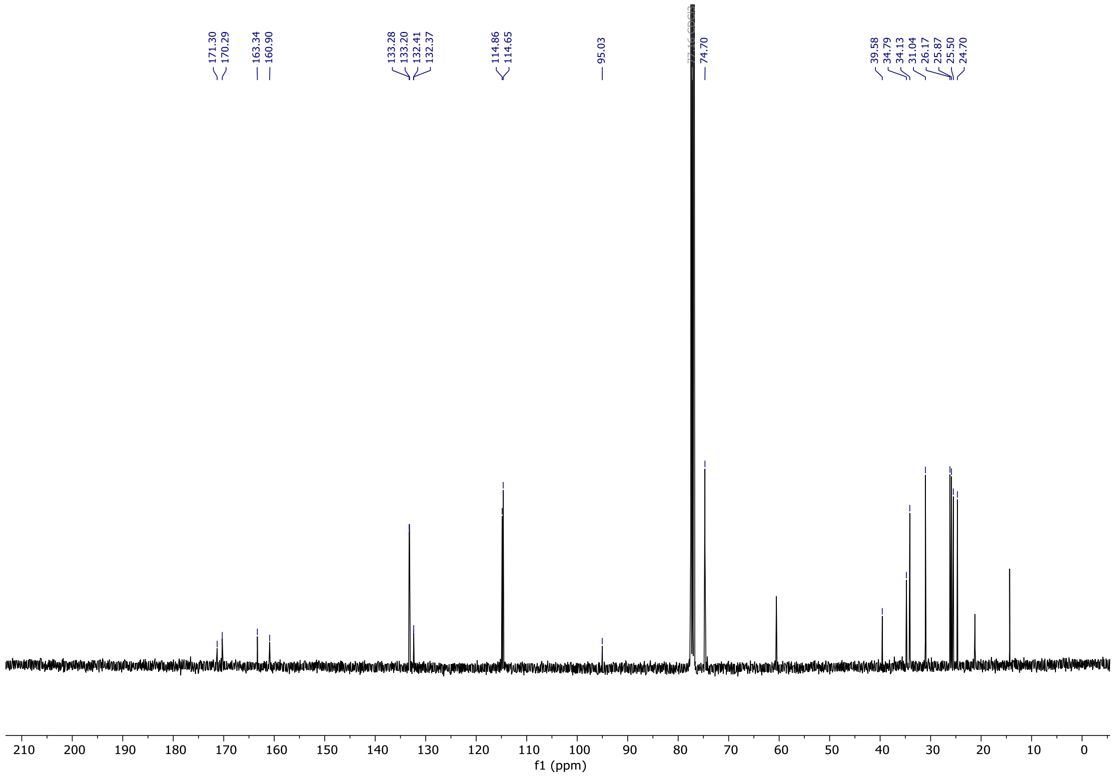

S24: ^{19}F NMR (282 MHz, $CDCl_3$):

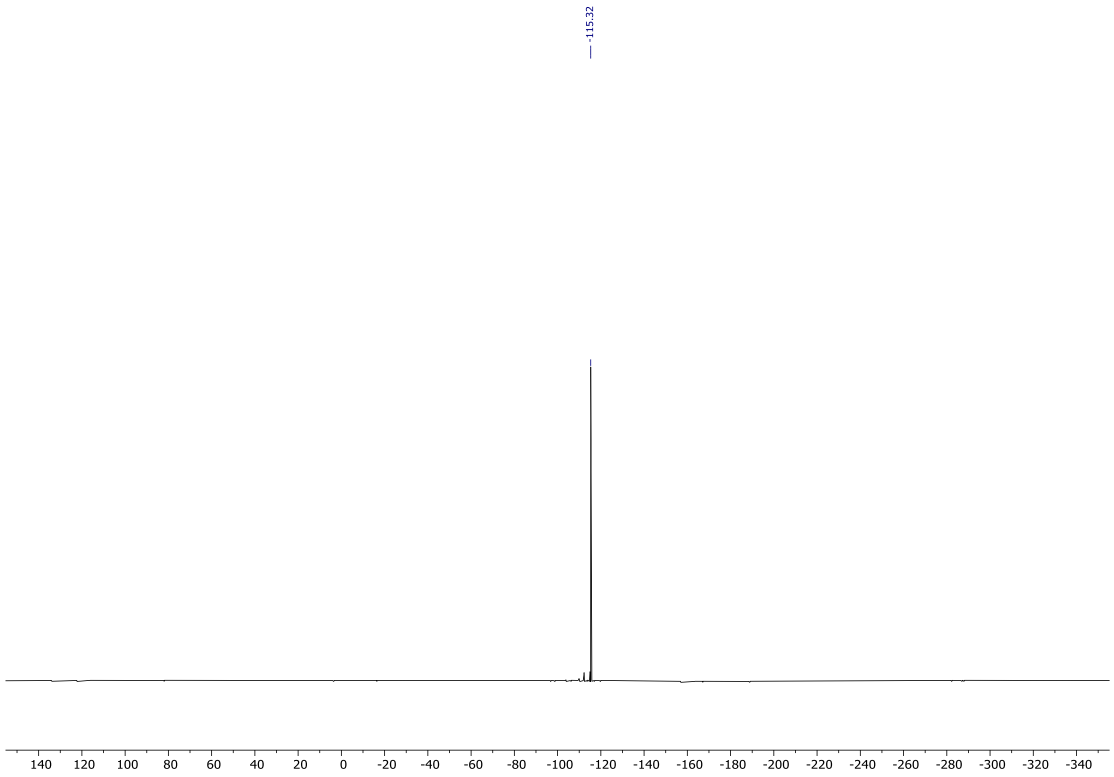

S25: ^{1}H NMR (400 MHz, $CDCl_3$):

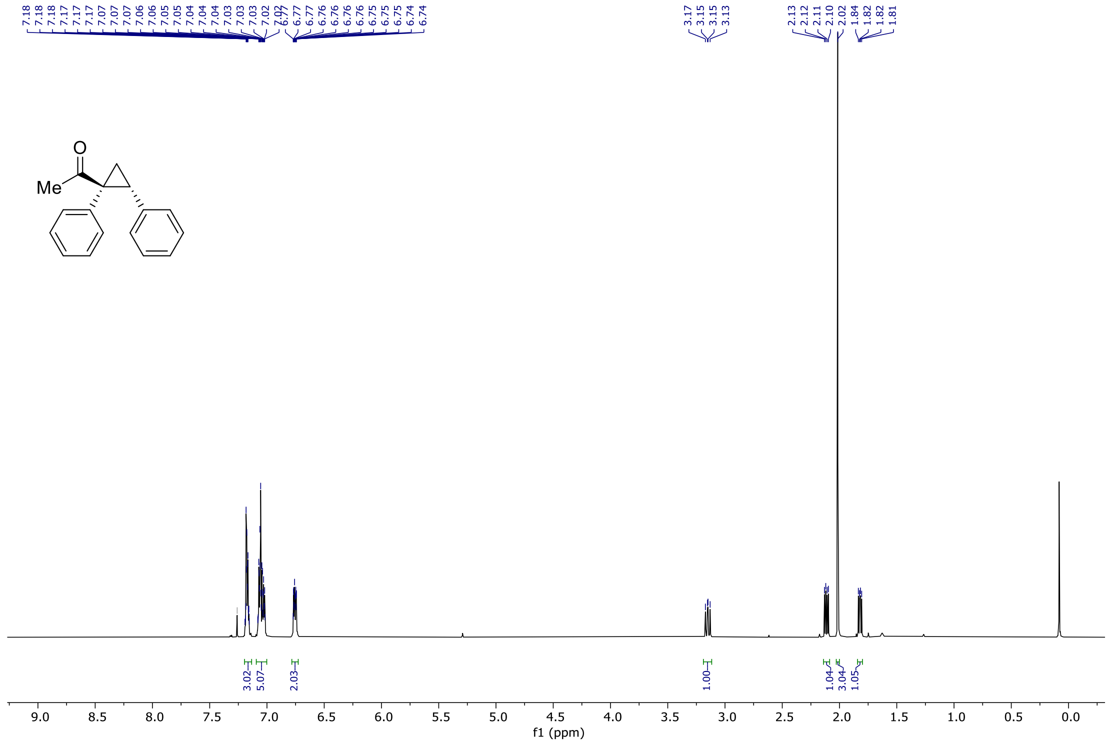

S25: ^{13}C NMR (101 MHz, $CDCl_3$):

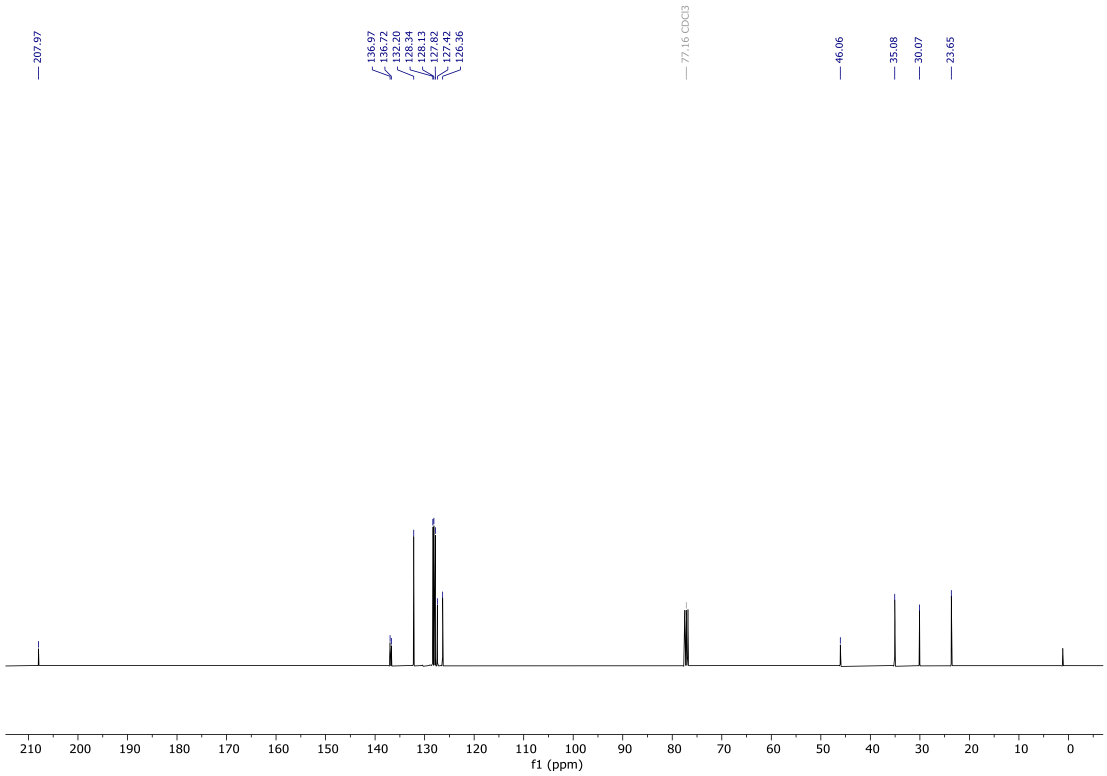

S26: ^{1}H NMR (400 MHz, $CDCl_3$):

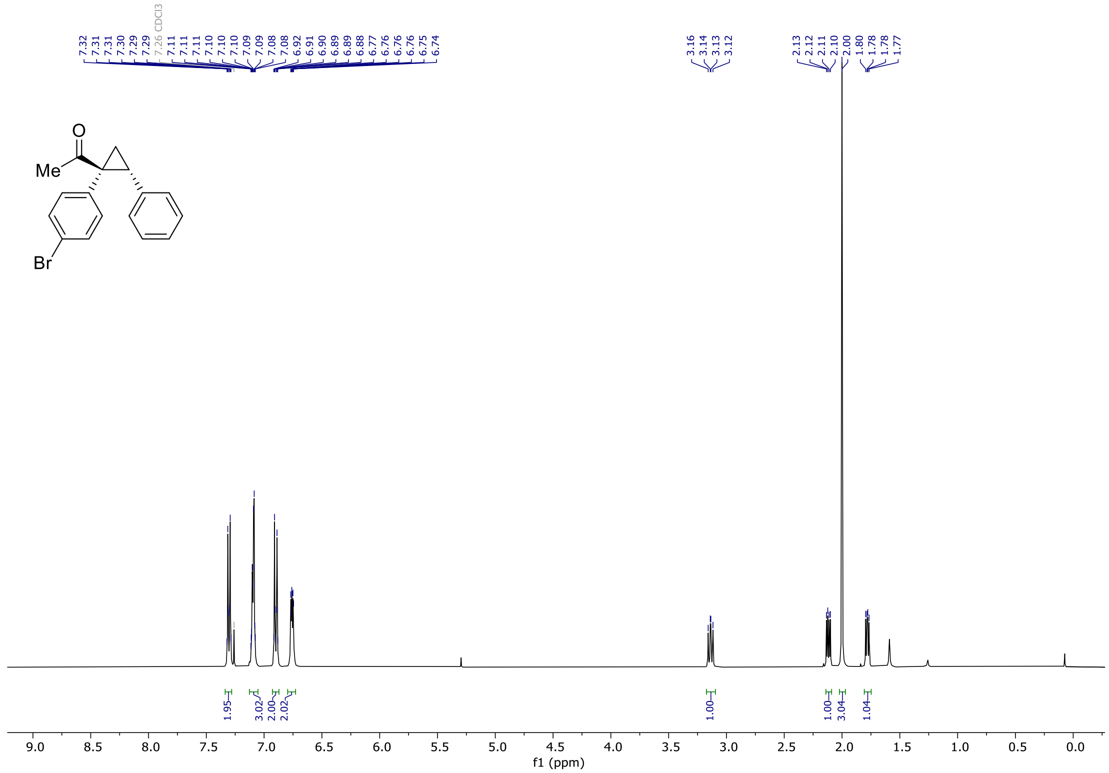

S26: ^{13}C NMR (101 MHz, $CDCl_3$):

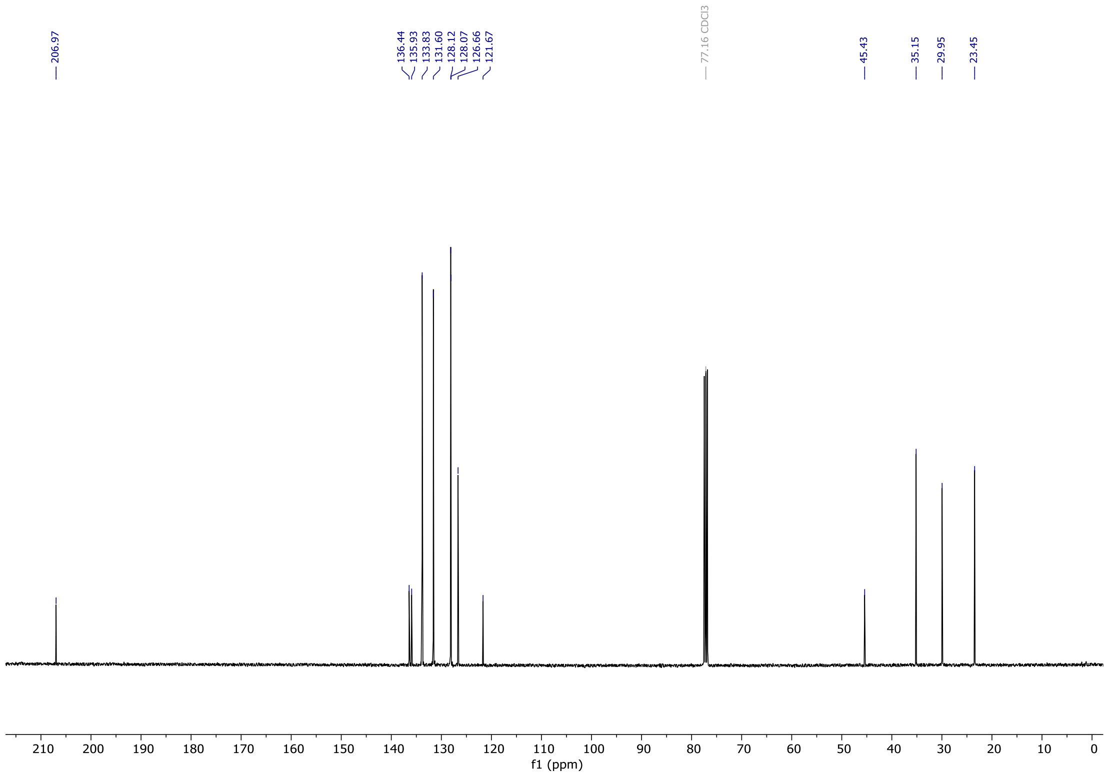

S27: ^{1}H NMR (400MHz, $CDCl_3$):

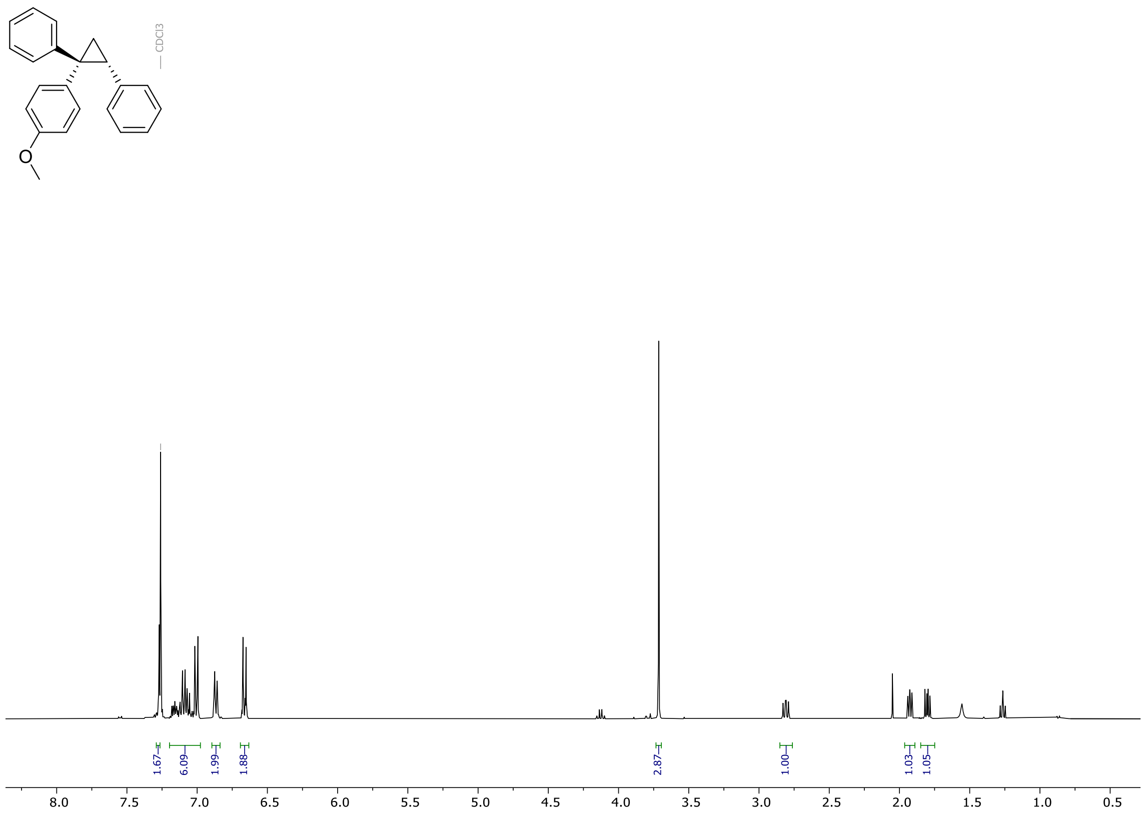

S27: ^{13}C NMR (101 MHz, $CDCl_3$):

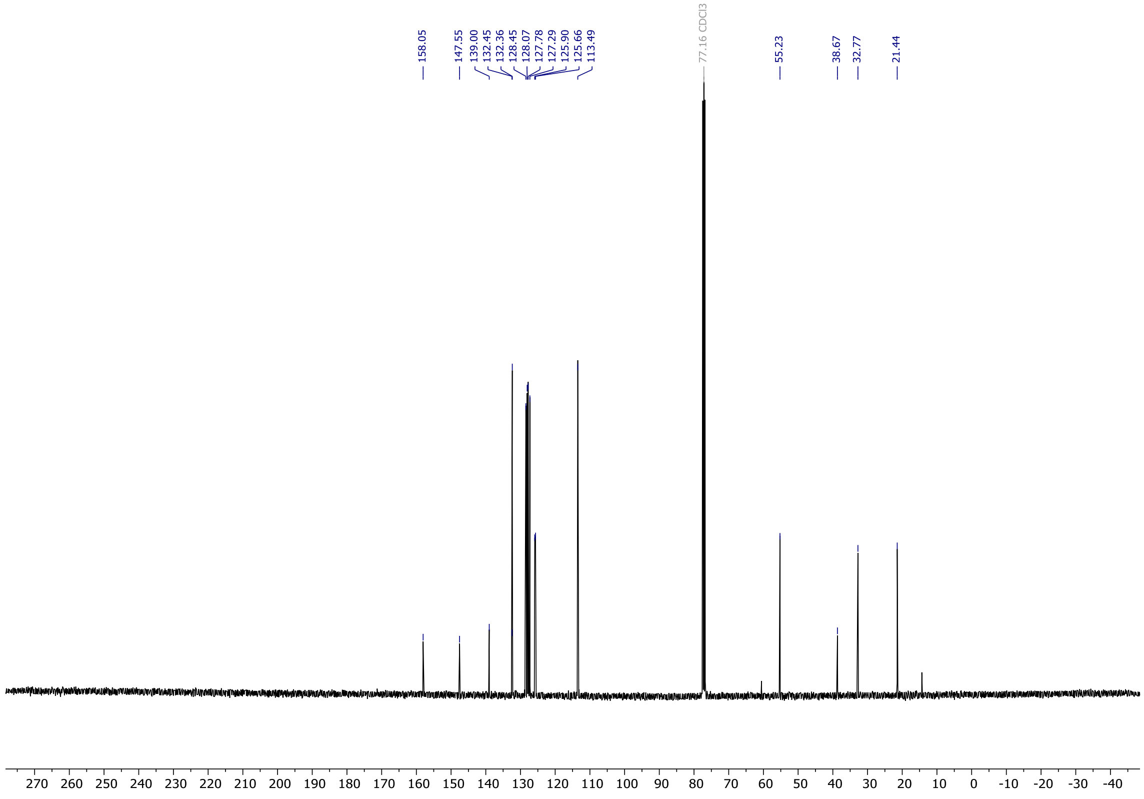

S27: HSQC NMR (400 MHz, 101 MHz, $CDCl_3$):

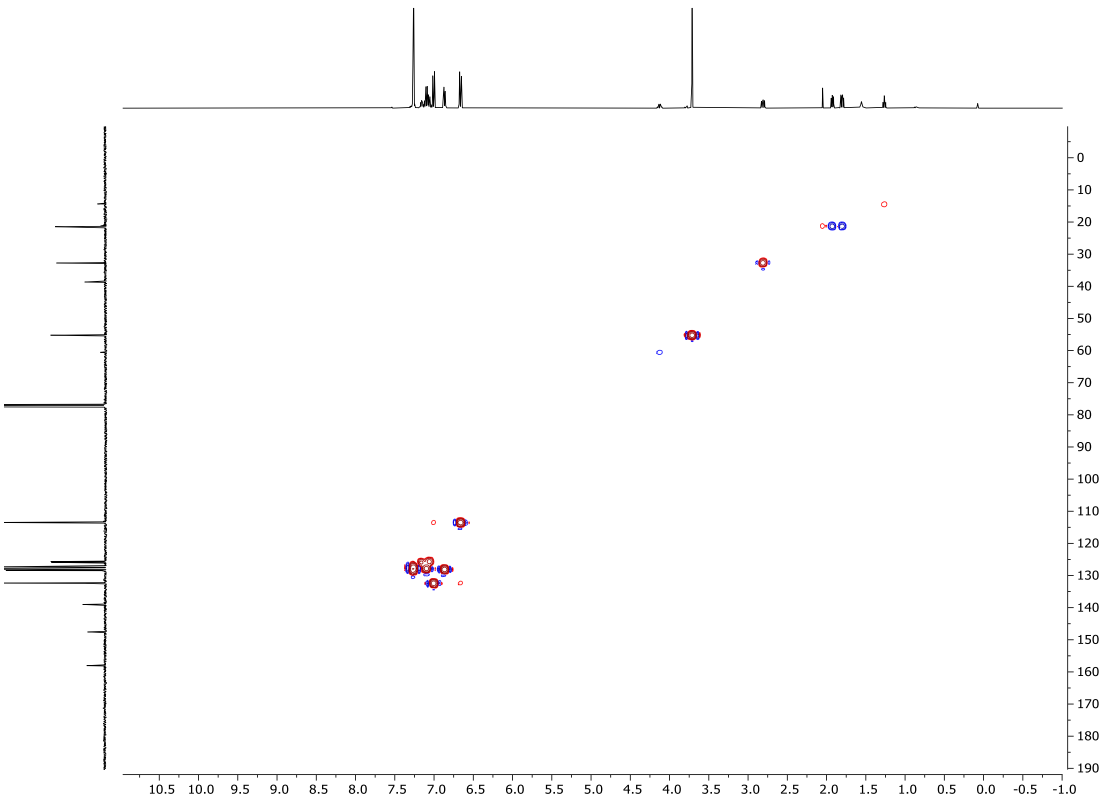

S27: HMBC NMR (400 MHz, 101 MHz, $CDCl_3$):

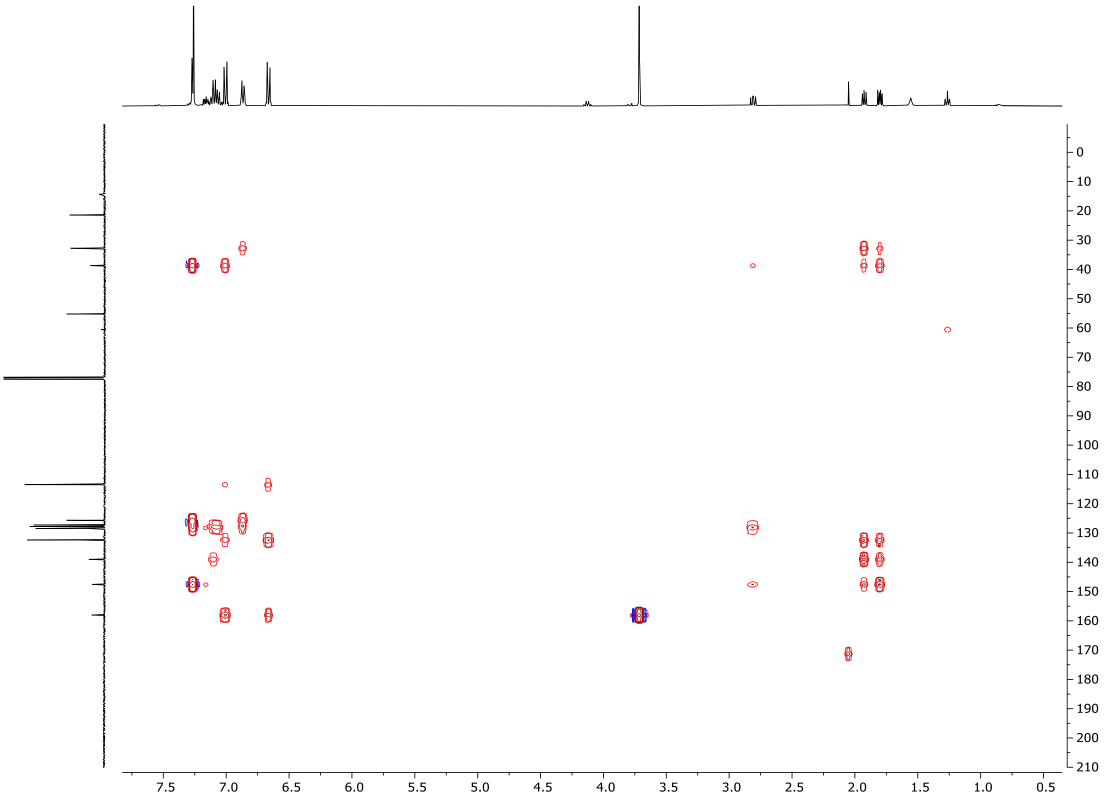

S27: NOESY NMR (400 MHz, $CDCl_3$):

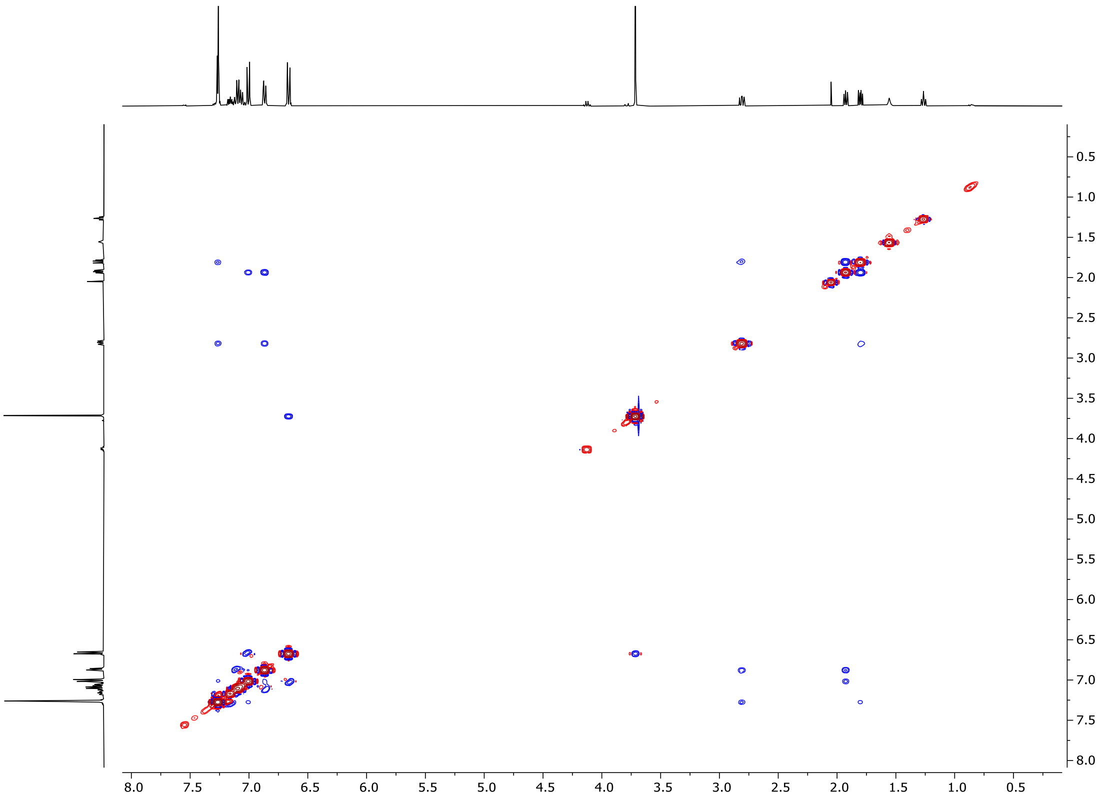

S28: ^{1}H NMR (400 MHz, $CDCl_3$):

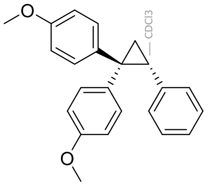

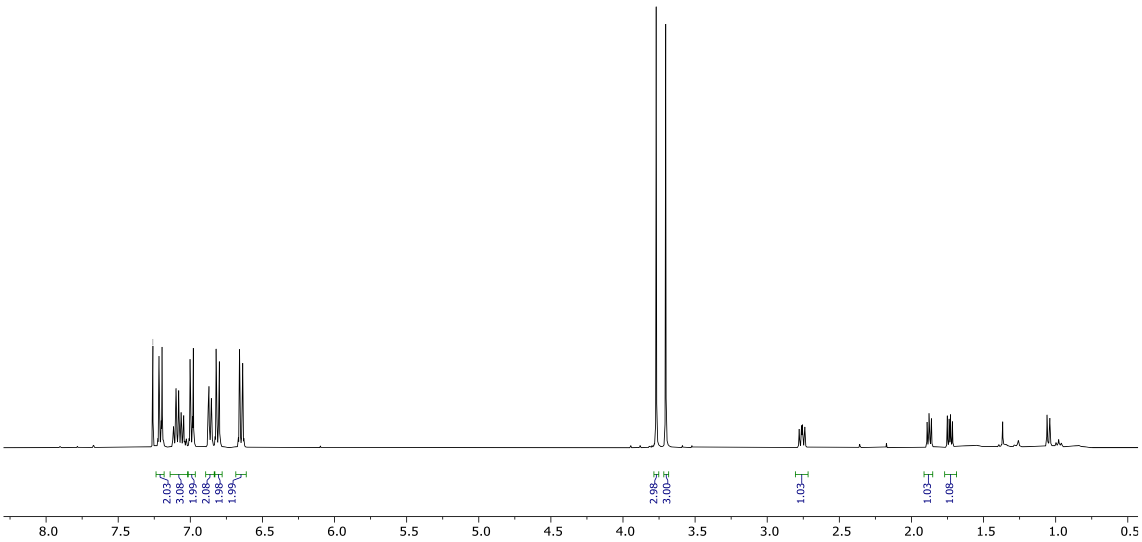

S28: ^{13}C NMR (101 MHz, $CDCl_3$):

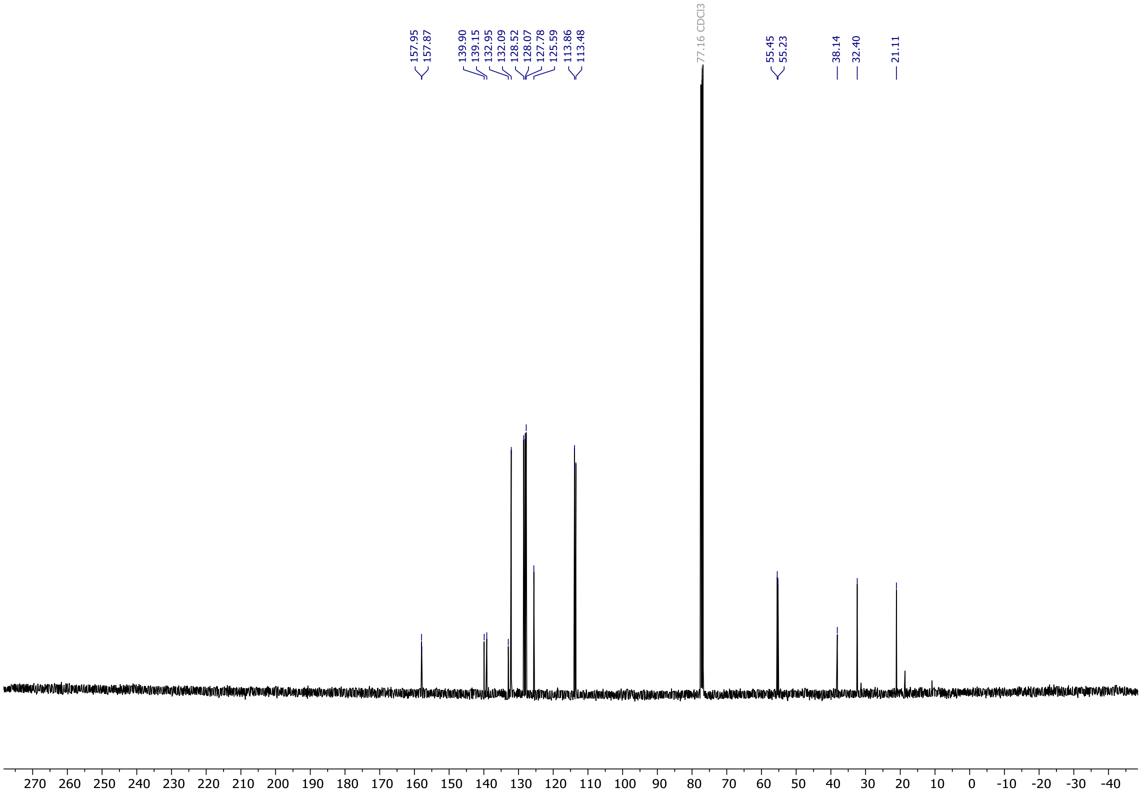

S29: ^{1}H NMR (400 MHz, $CDCl_3$):

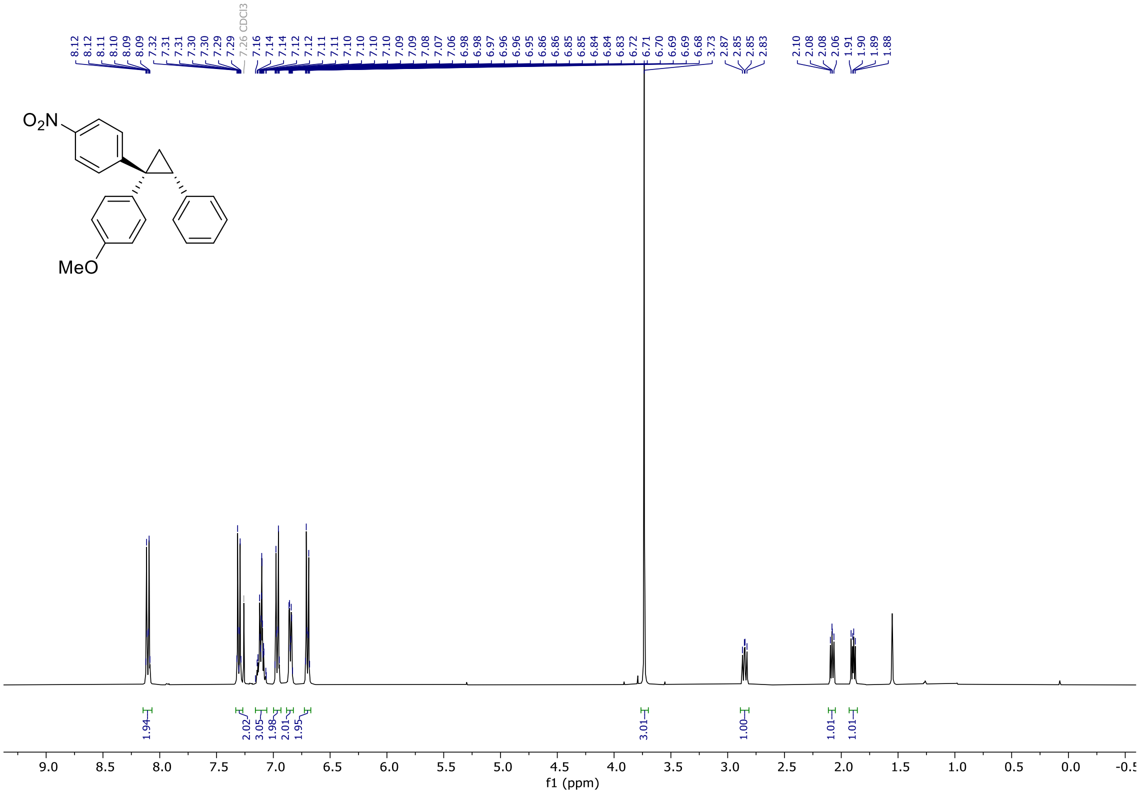

S29: ^{13}C NMR (101 MHz, $CDCl_3$):

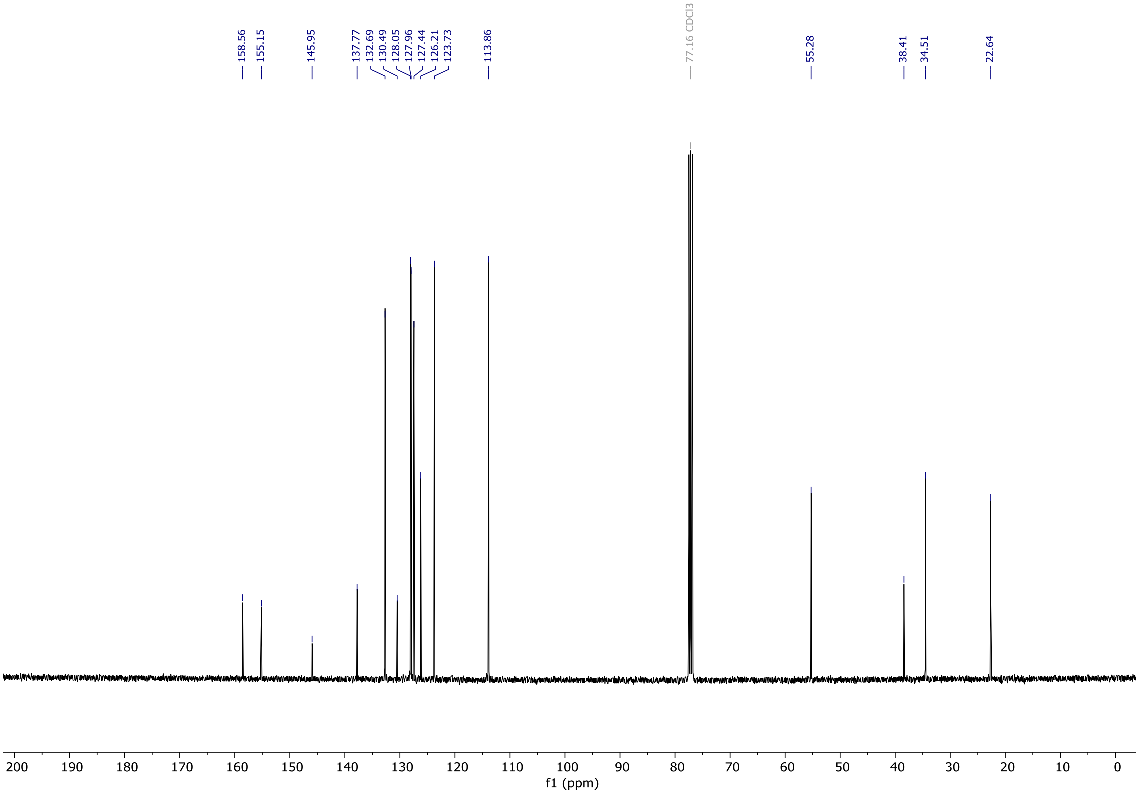

S30: ^{1}H NMR (400 MHz, $CDCl_3$):

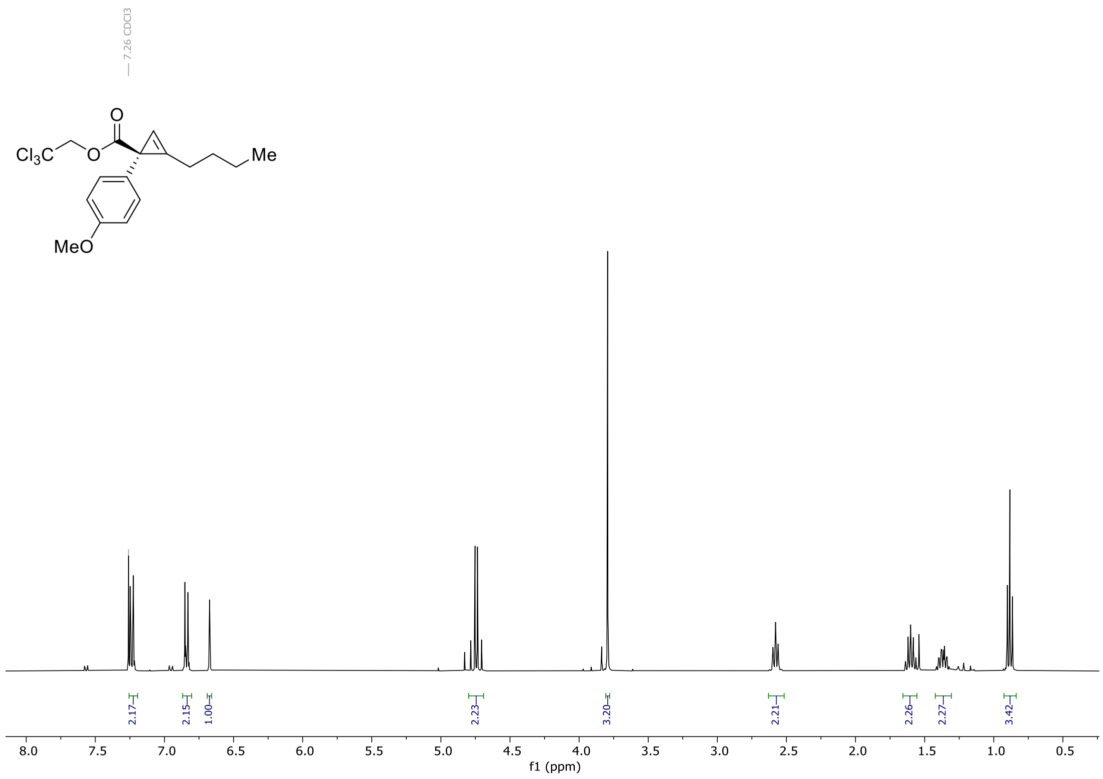

S30: ^{13}C NMR (101 MHz, $CDCl_3$):

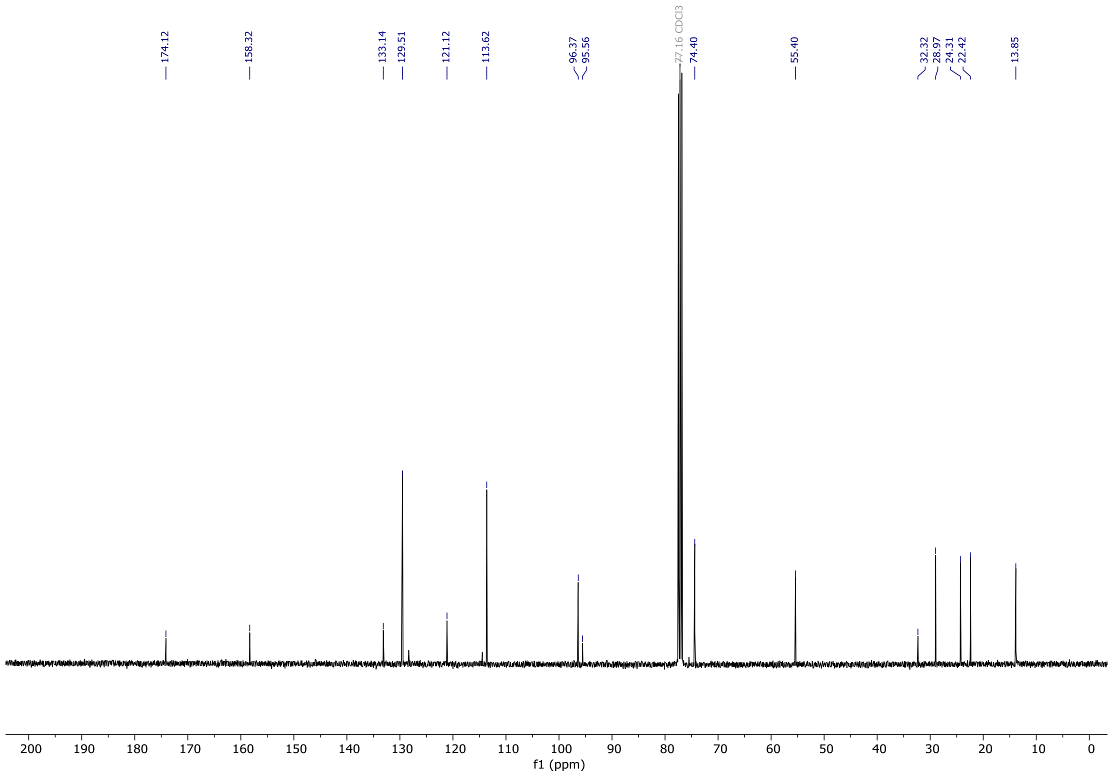

S31: ^{1}H NMR (400 MHz, $CDCl_3$):

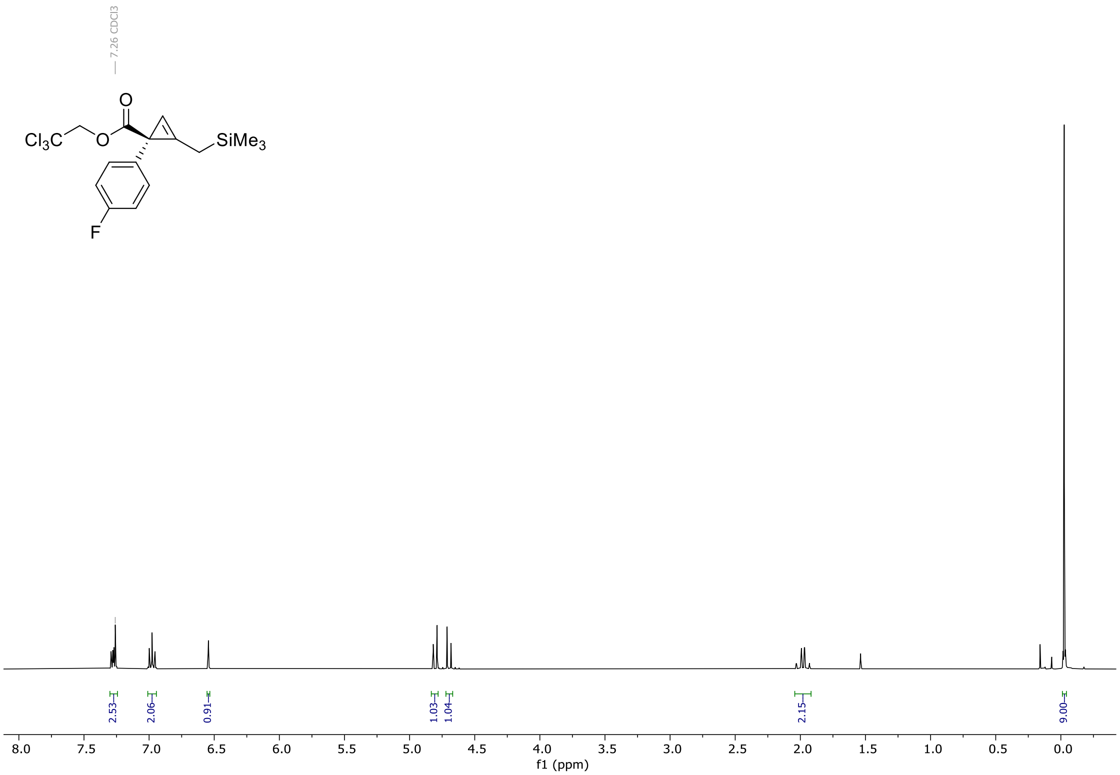

S31: ^{13}C NMR (101 MHz, $CDCl_3$):

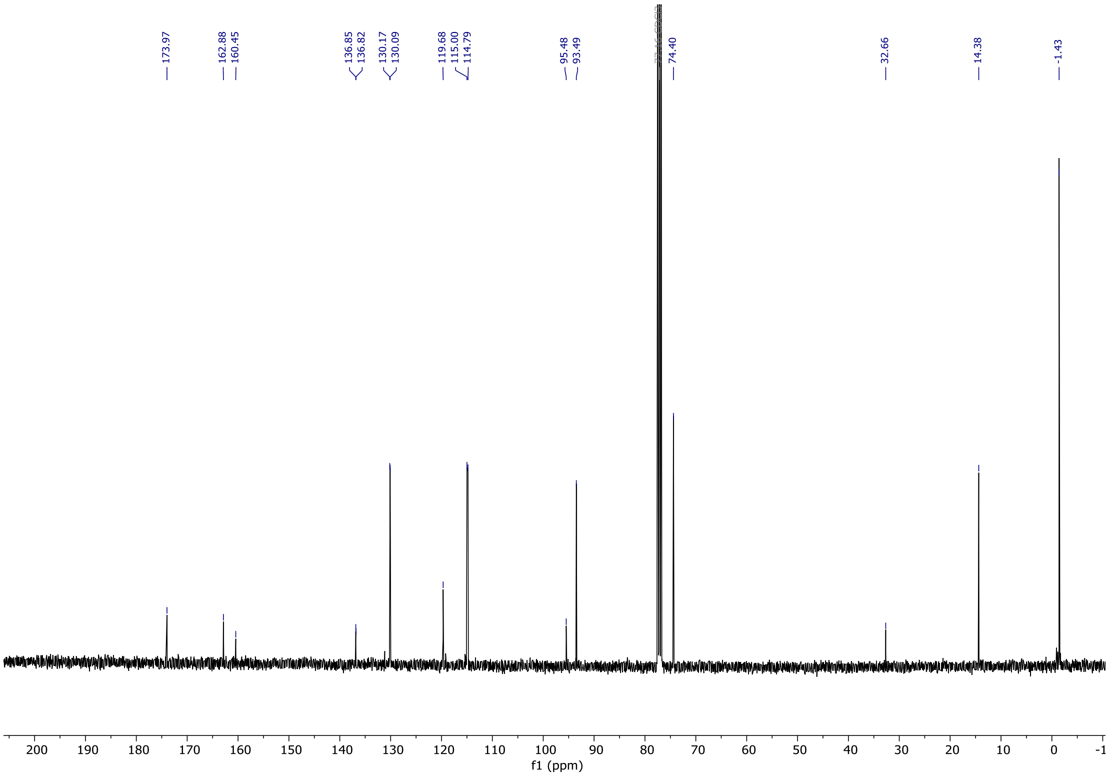

S31: ^{19}F NMR (282 MHz, $CDCl_3$):

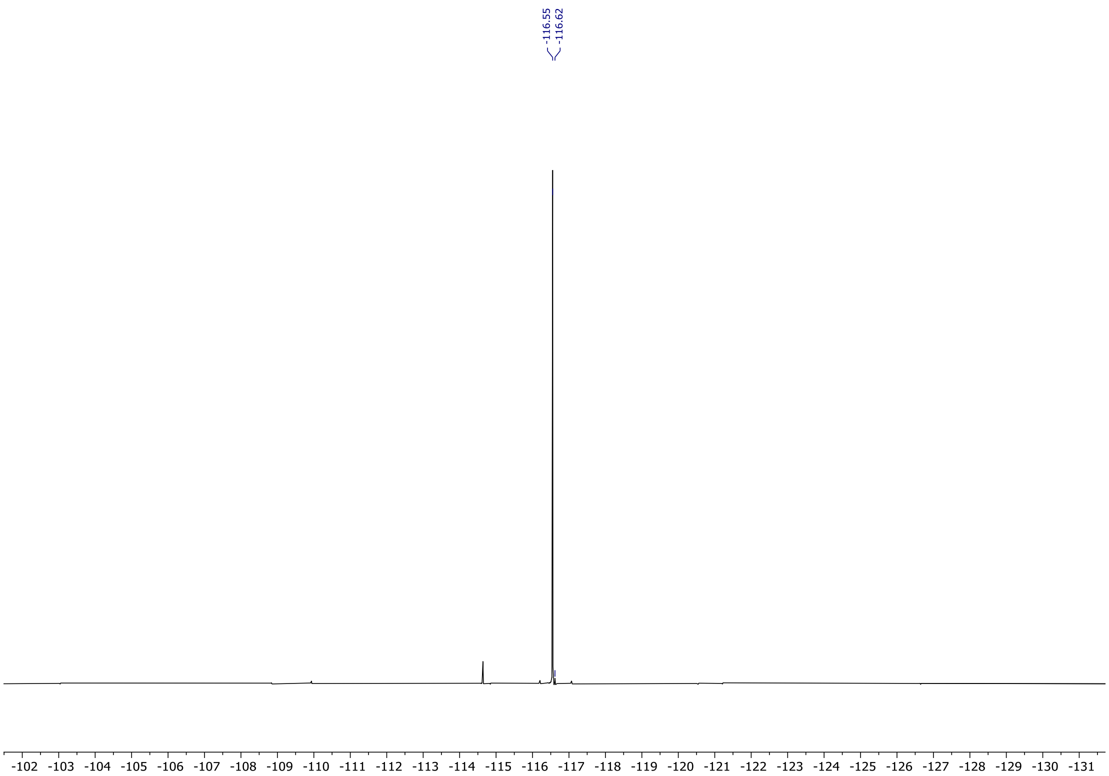

S31: ^{29}Si NMR (79 MHz, $CDCl_3$):

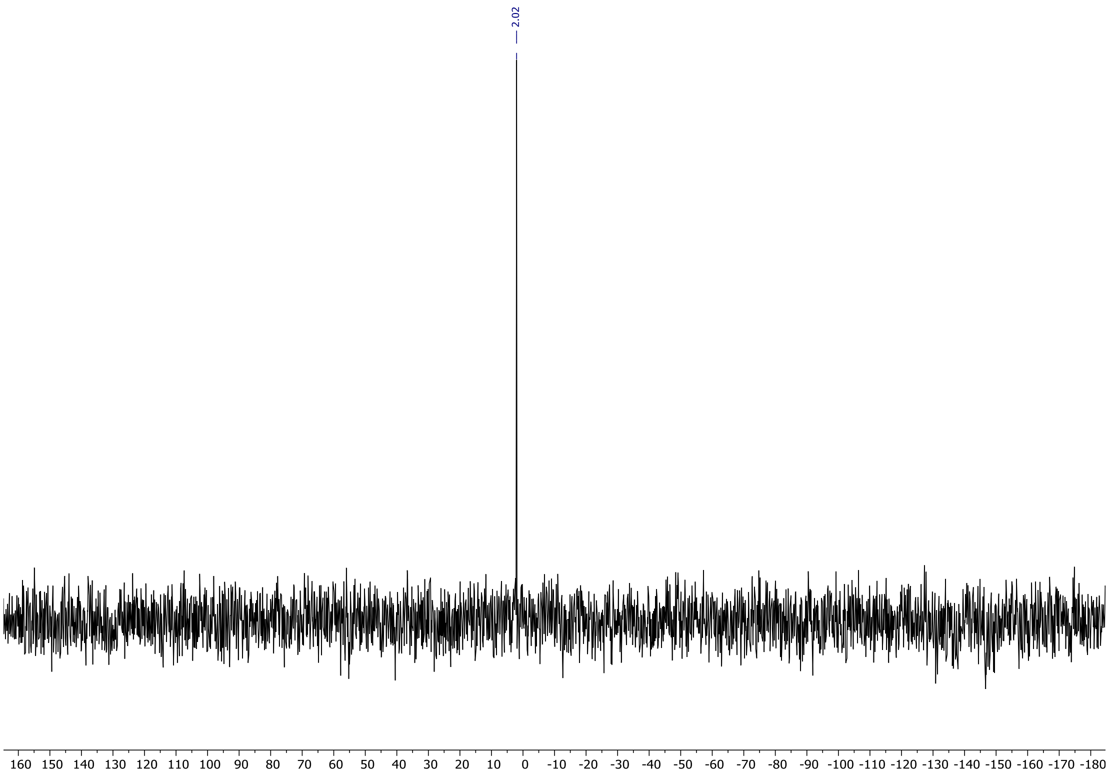

S32: ^{1}H NMR (400 MHz, $CDCl_3$):

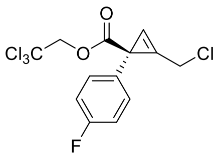

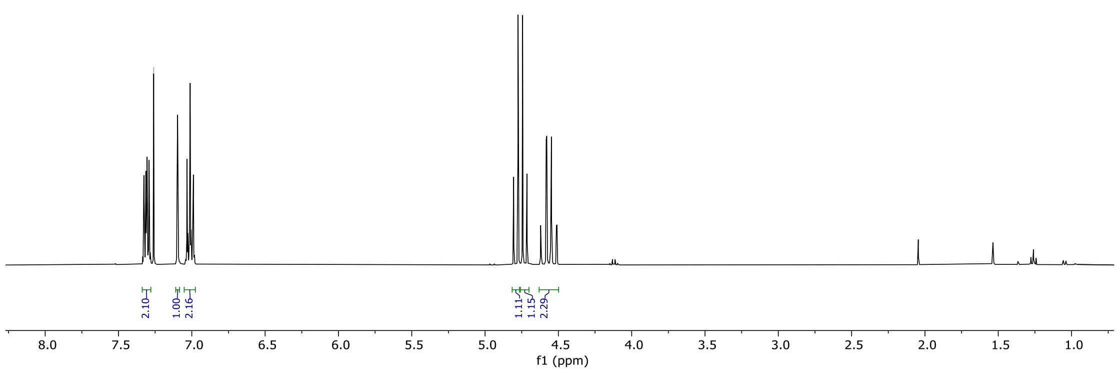

S32: ^{13}C NMR (101 MHz, $CDCl_3$):

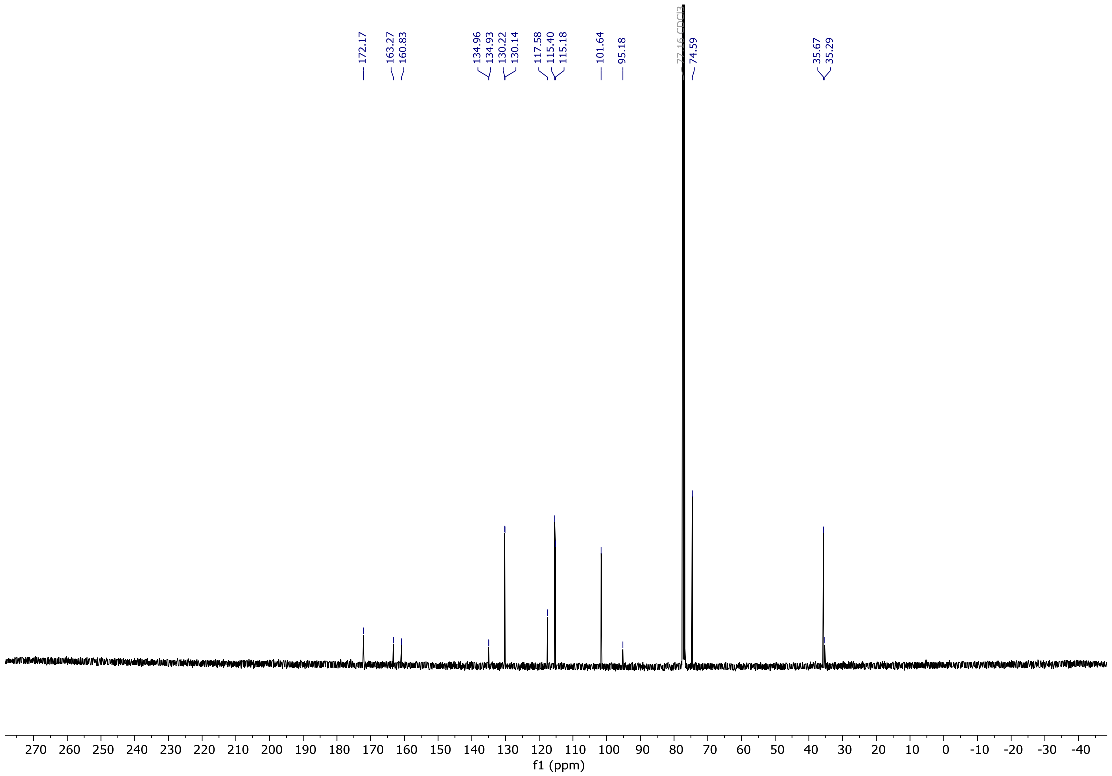

S32: ^{19}F NMR (282 MHz, $CDCl_3$):

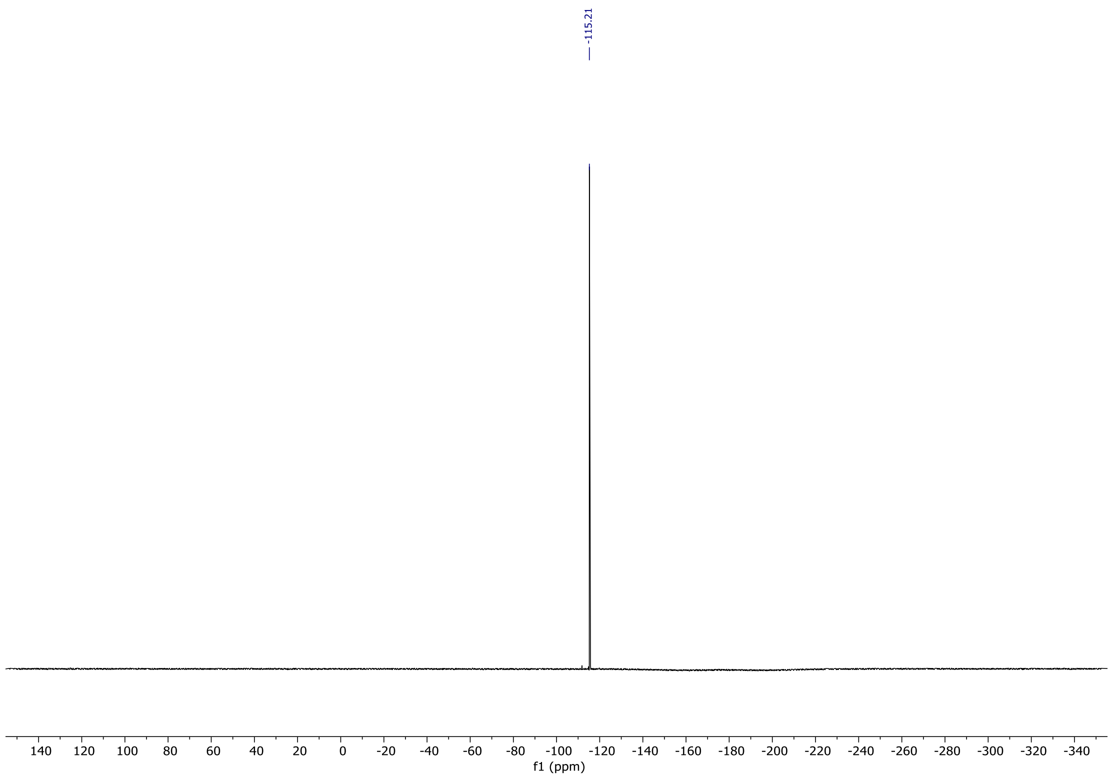

S33: ^{1}H NMR (400 MHz, $CDCl_3$):

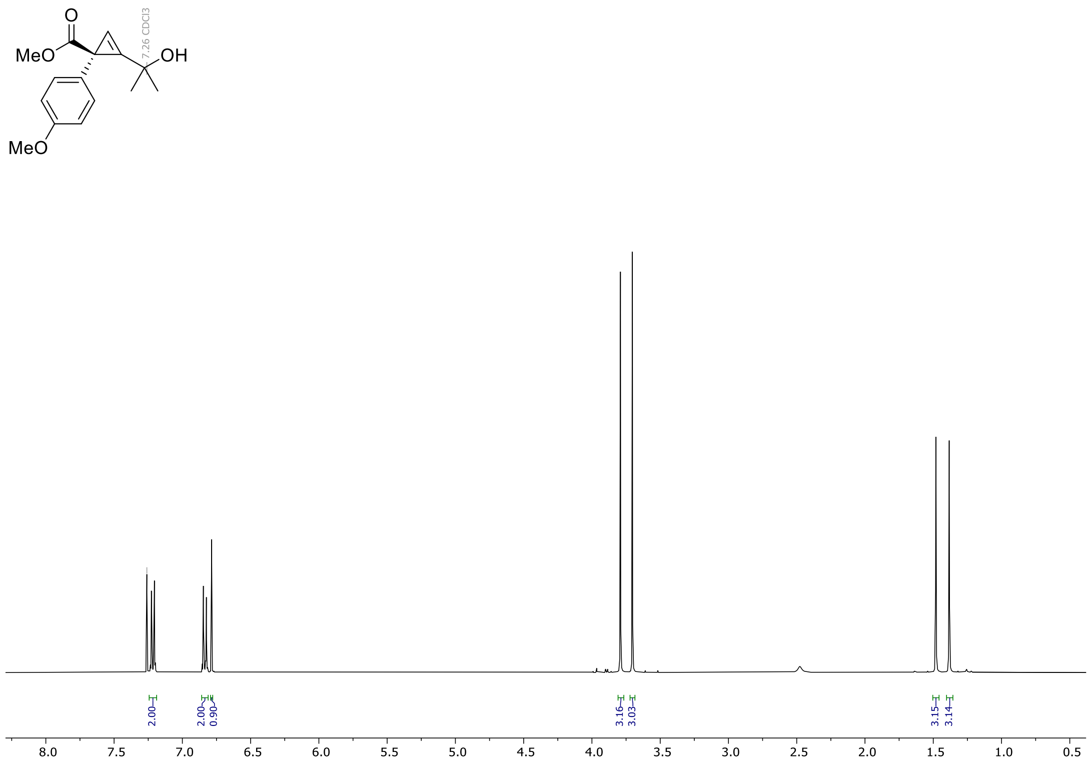

S33: ^{13}C NMR (101 MHz, $CDCl_3$):

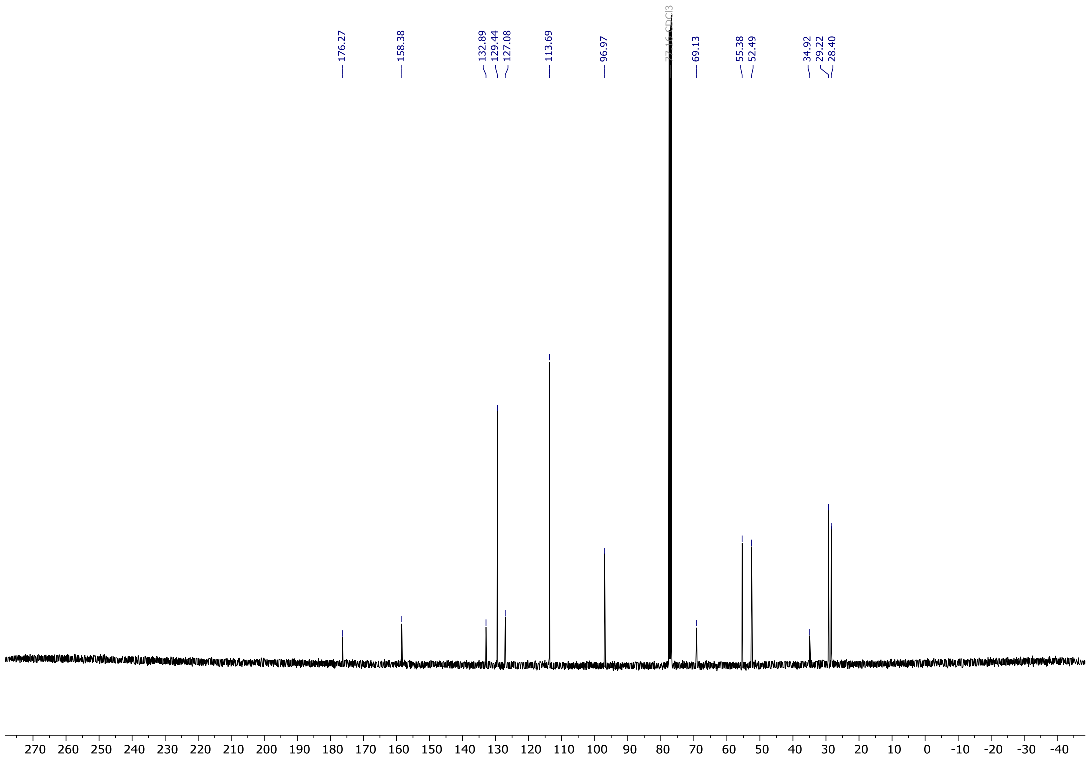

S34: ^{1}H NMR (400 MHz, $CDCl_3$):

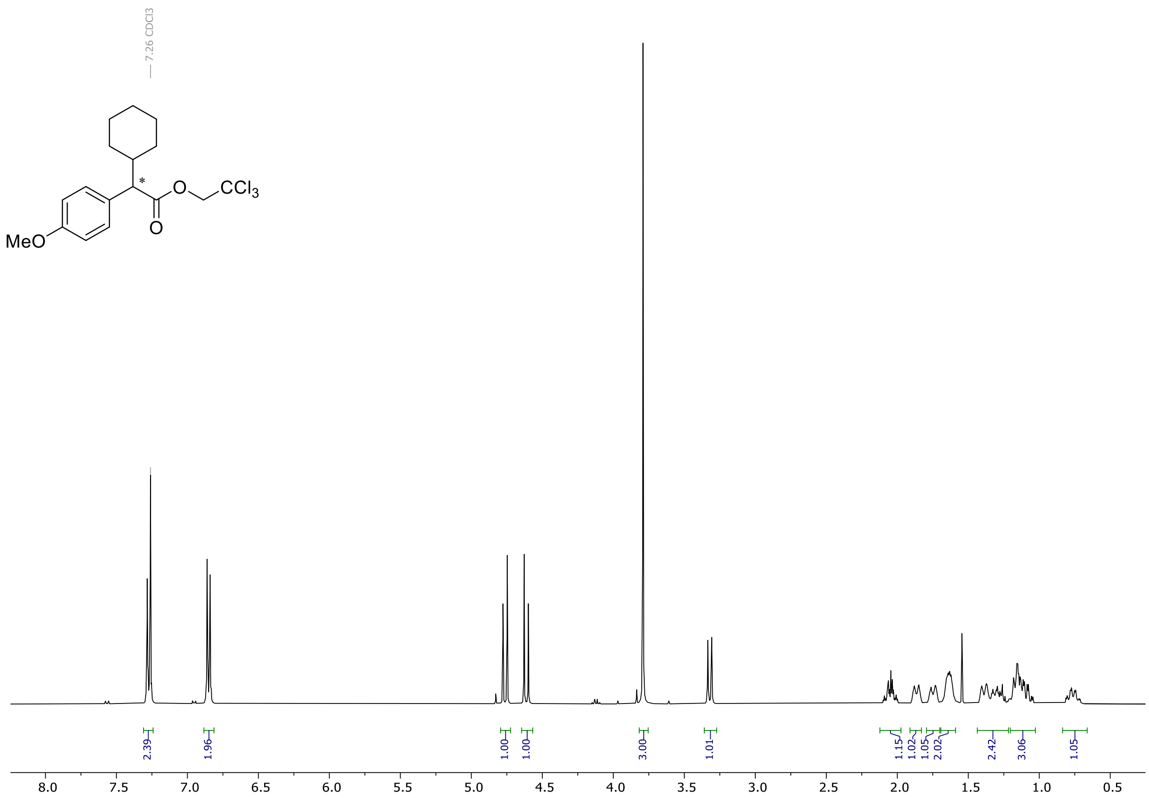

S34: ^{13}C NMR (101 MHz, $CDCl_3$):

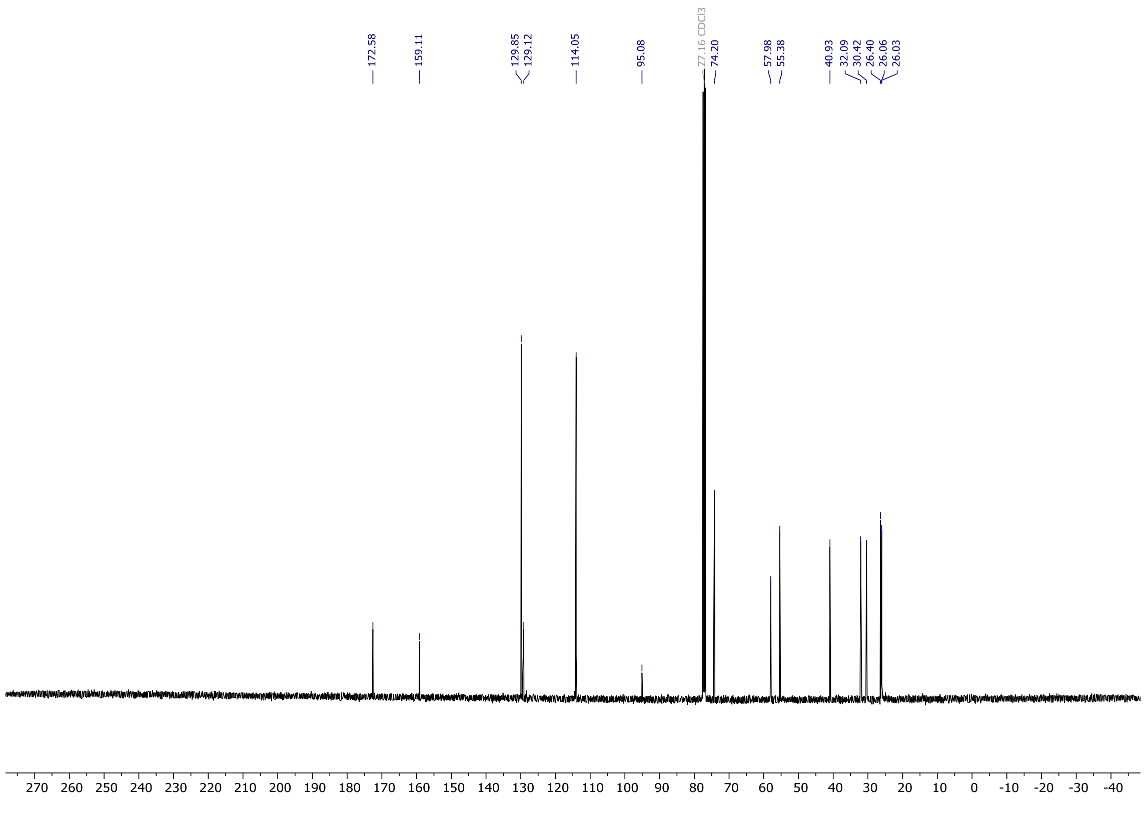

S35: ^{1}H NMR (400 MHz, $CDCl_3$):

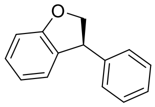

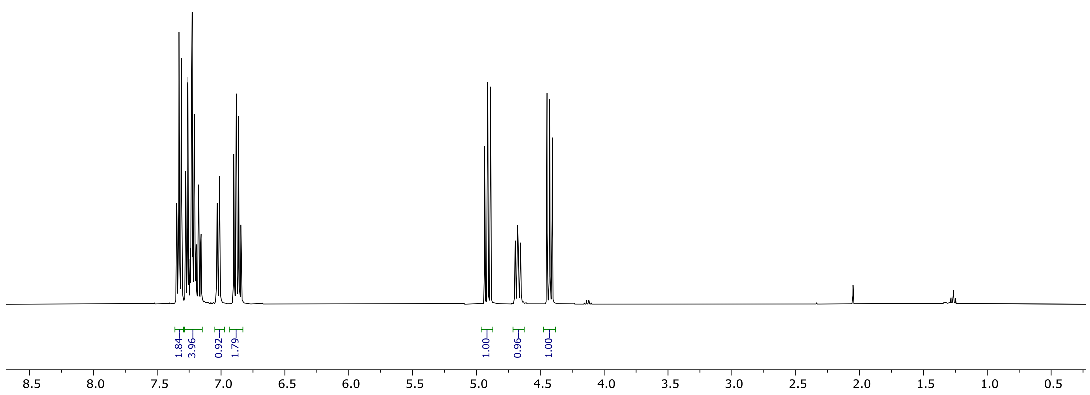

S35: ^{13}C NMR (101 MHz, $CDCl_3$):

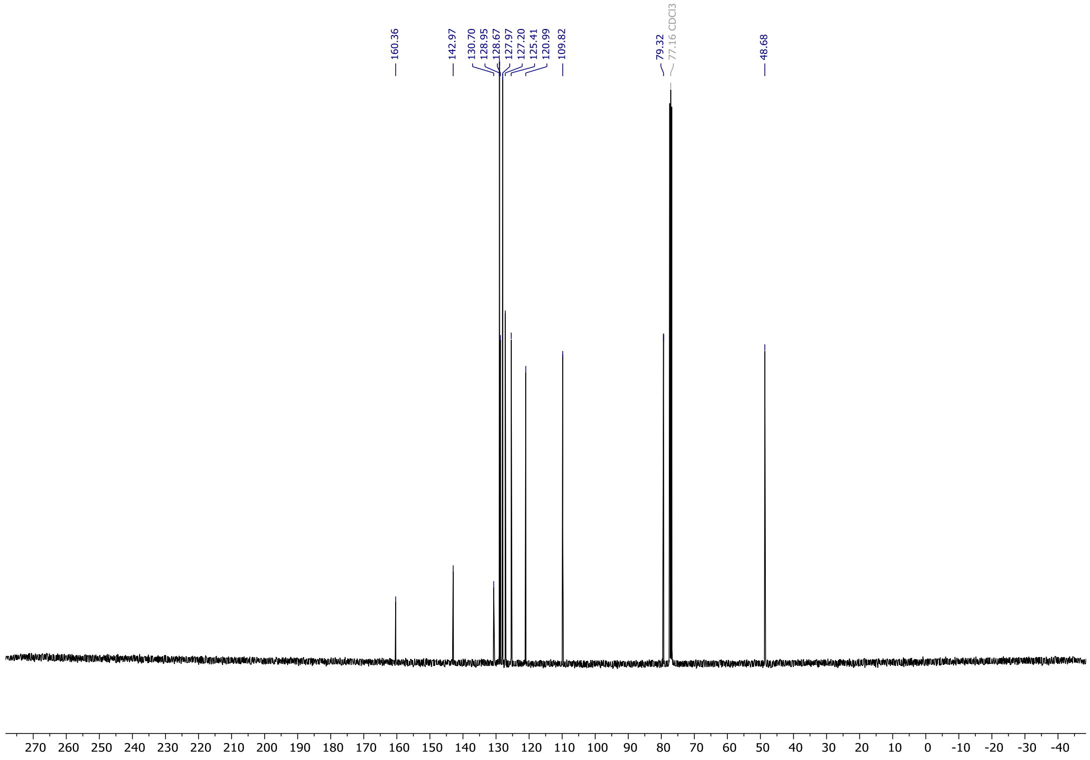

S36: ^{1}H NMR (400 MHz, $CDCl_3$):

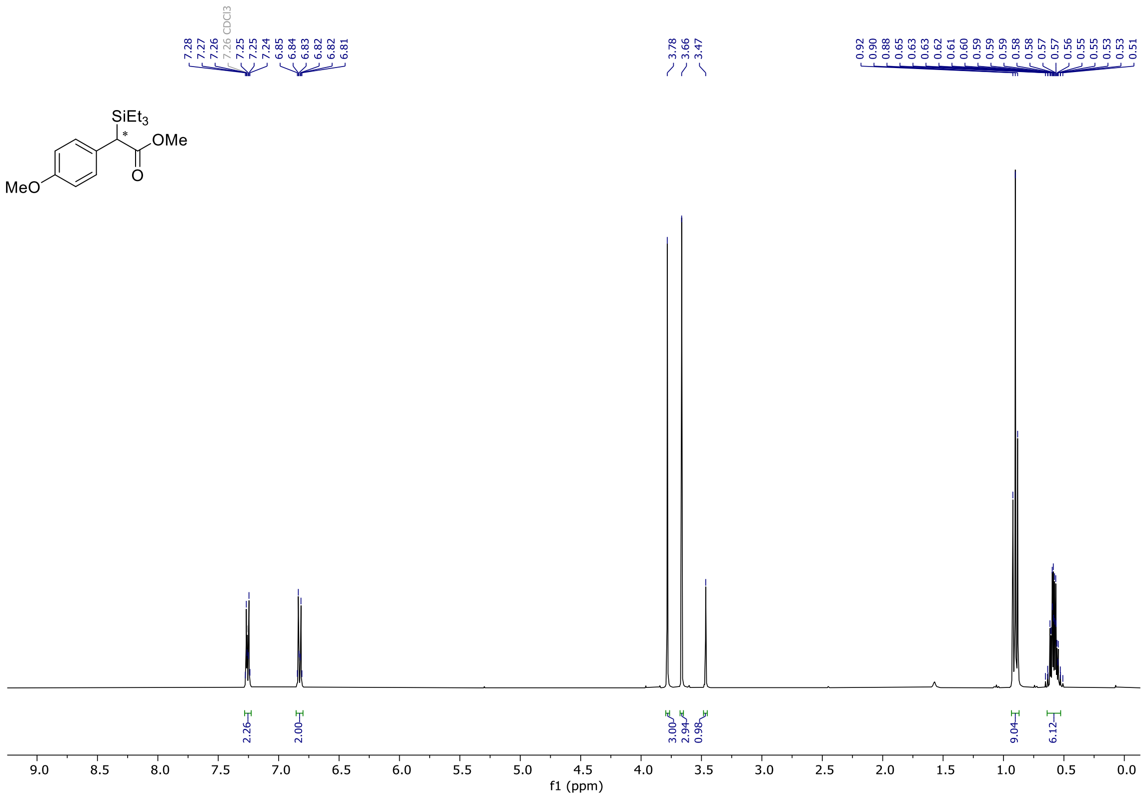

S36: ^{13}C NMR (101 MHz, $CDCl_3$):

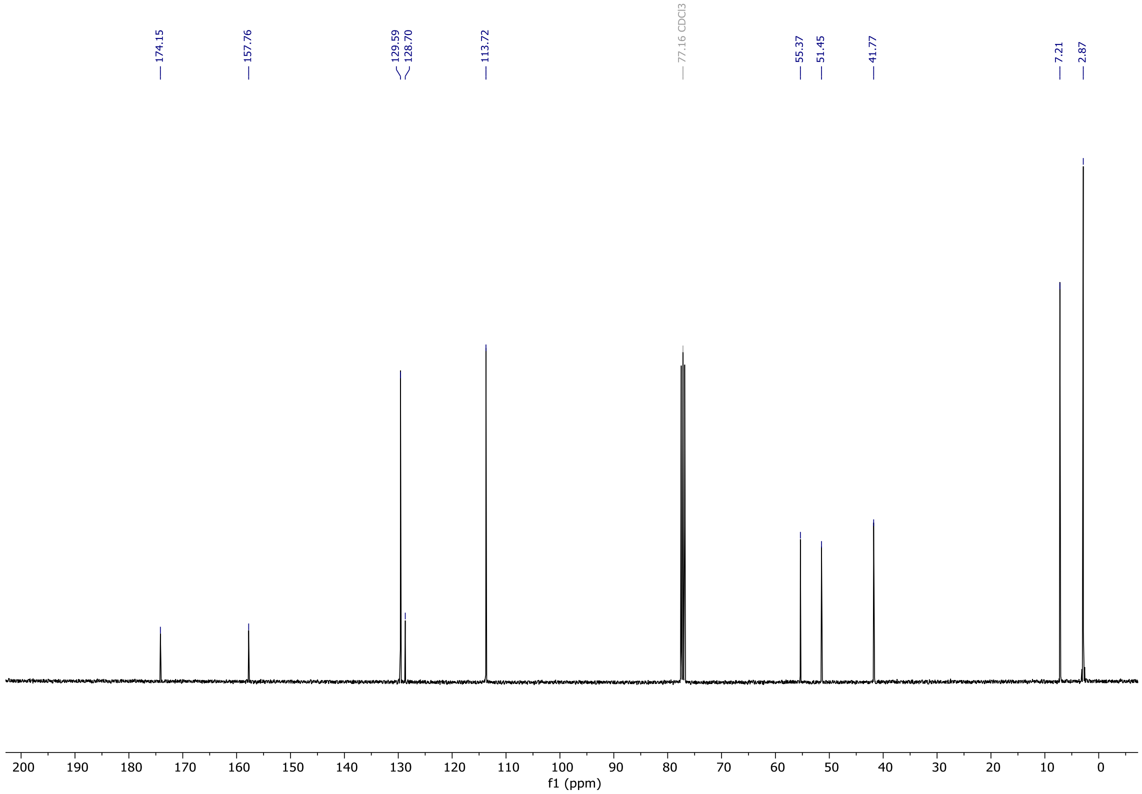

S37: ^{1}H NMR (400 MHz, $CDCl_3$):

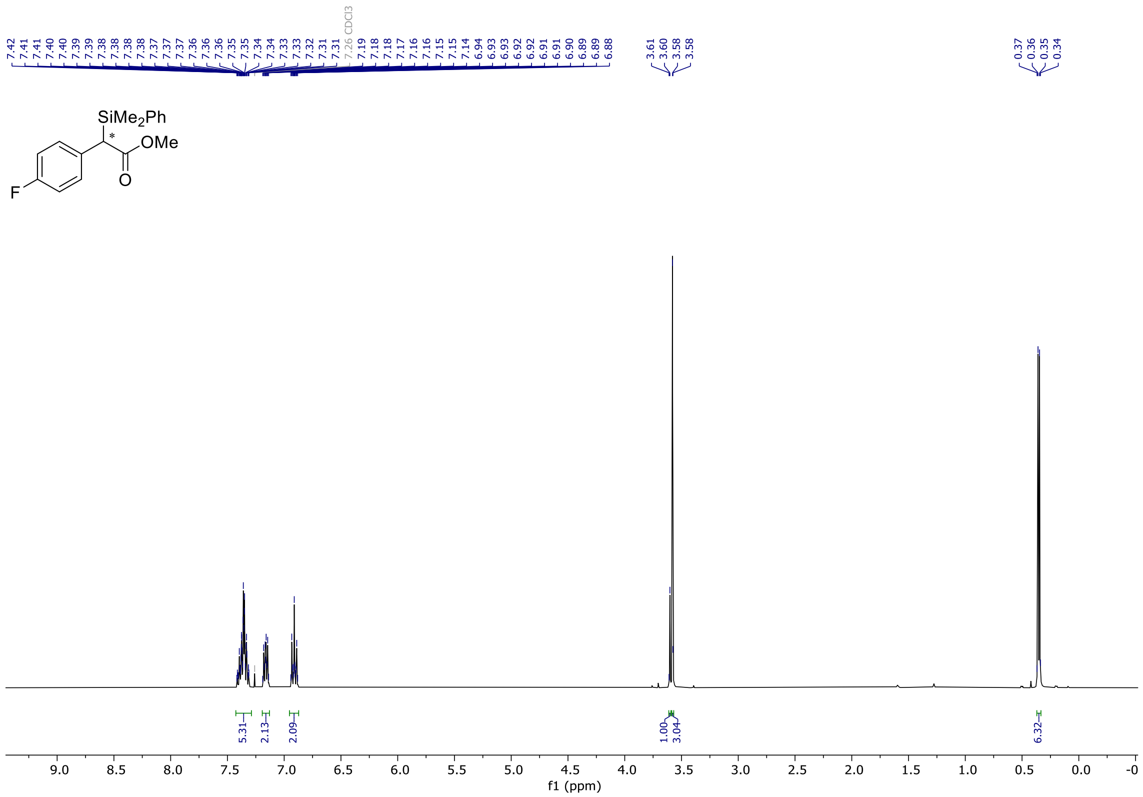

S37: ^{13}C NMR (101 MHz, $CDCl_3$):

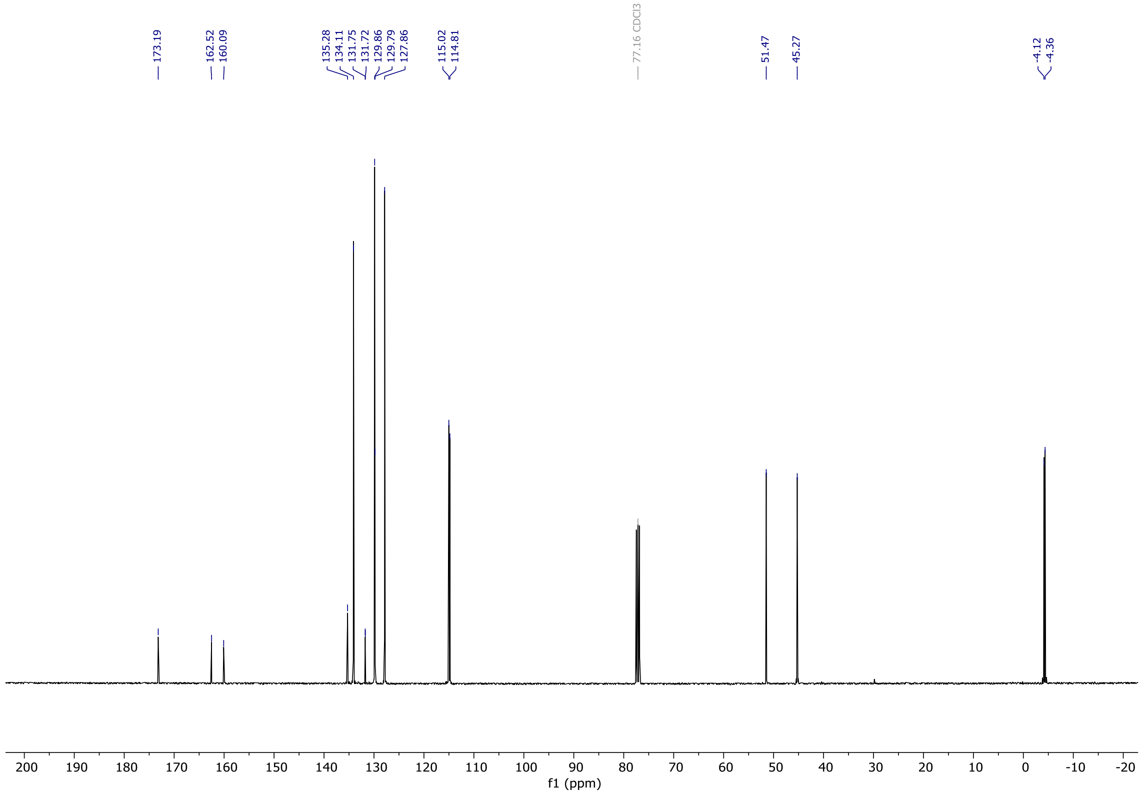

S37: ^{19}F NMR (282 MHz, $CDCl_3$):

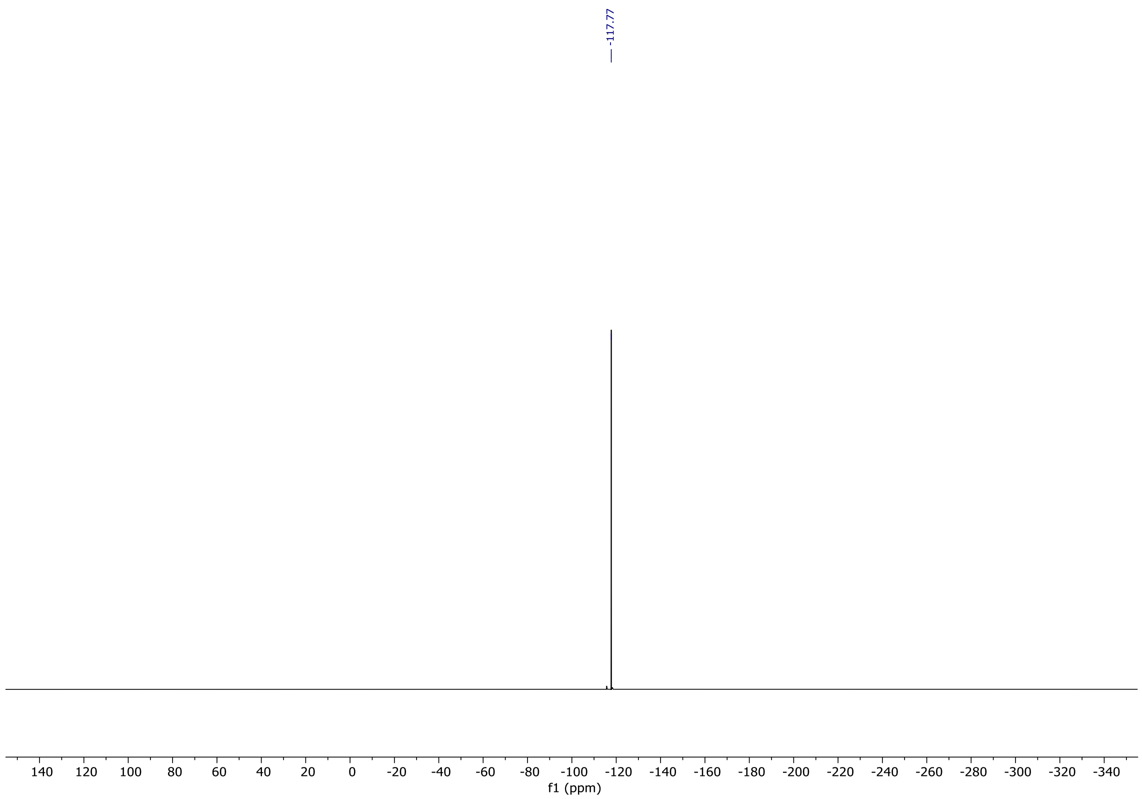

S38: ^{1}H NMR (400 MHz, $CDCl_3$):

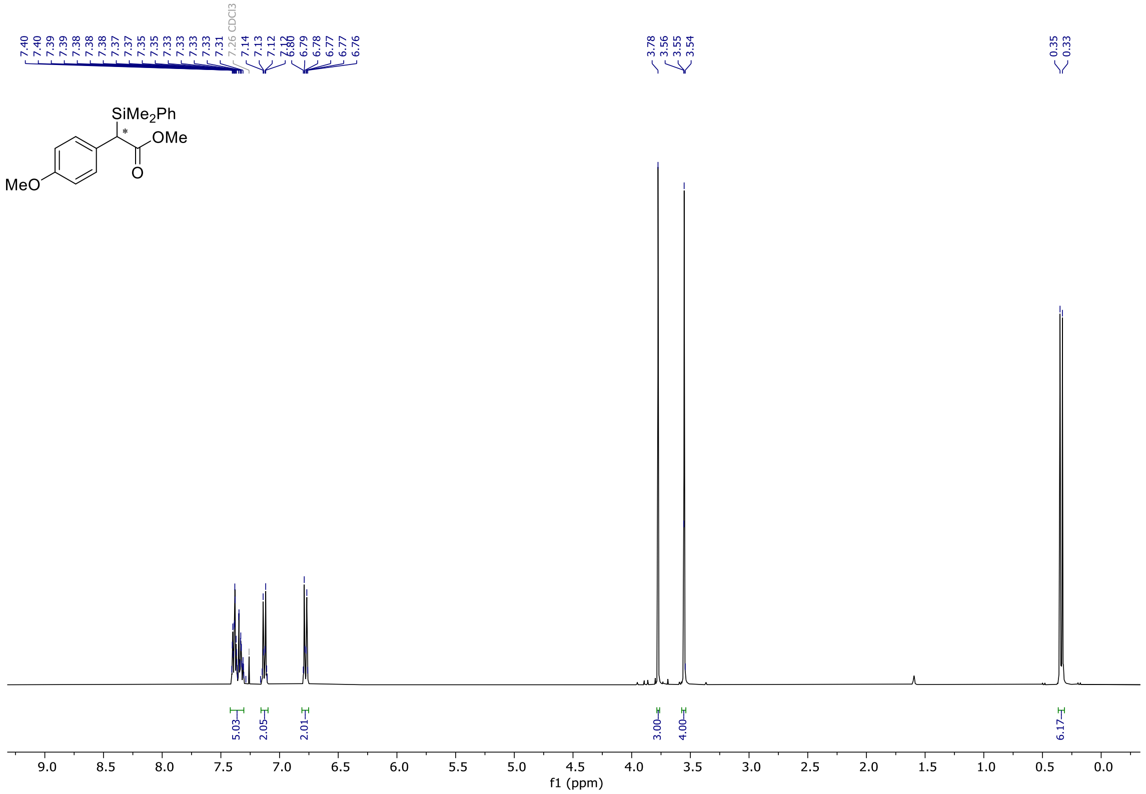

S38: ^{13}C NMR (101 MHz, $CDCl_3$):

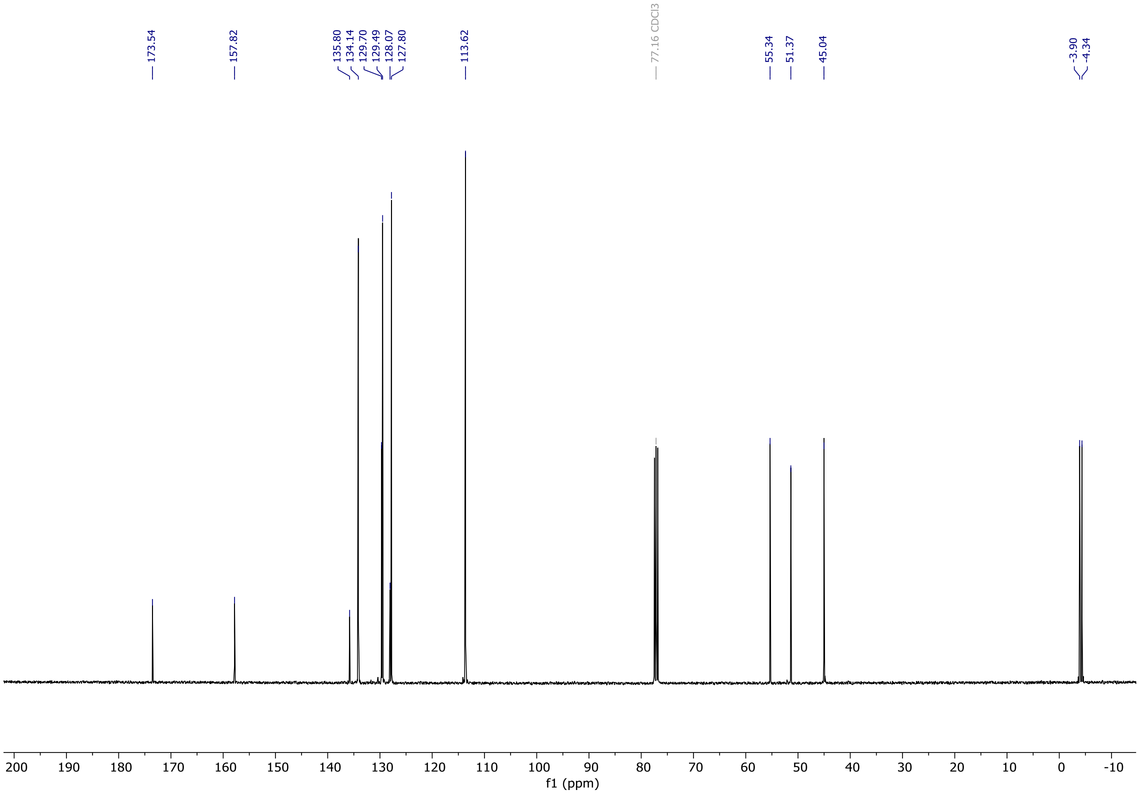

S39: ^{1}H NMR (400 MHz, $CDCl_3$):

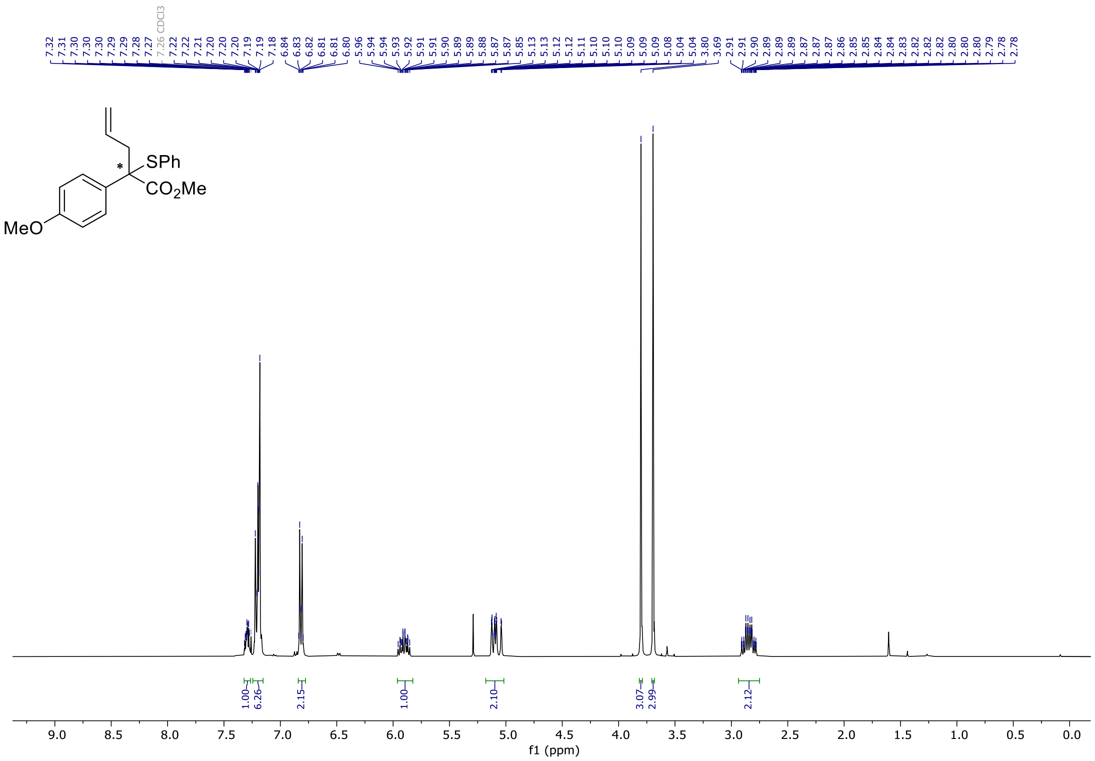

S39: ^{13}C NMR (101 MHz, $CDCl_3$):

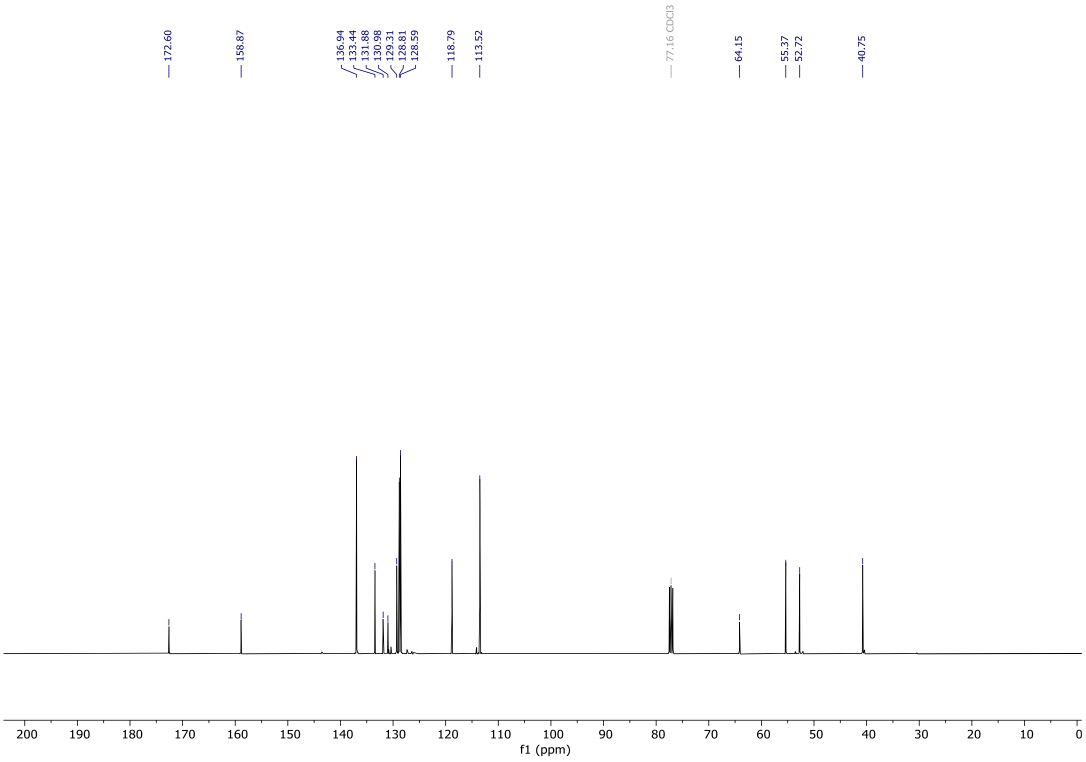

S40: ^{1}H NMR (400 MHz, $CDCl_3$):

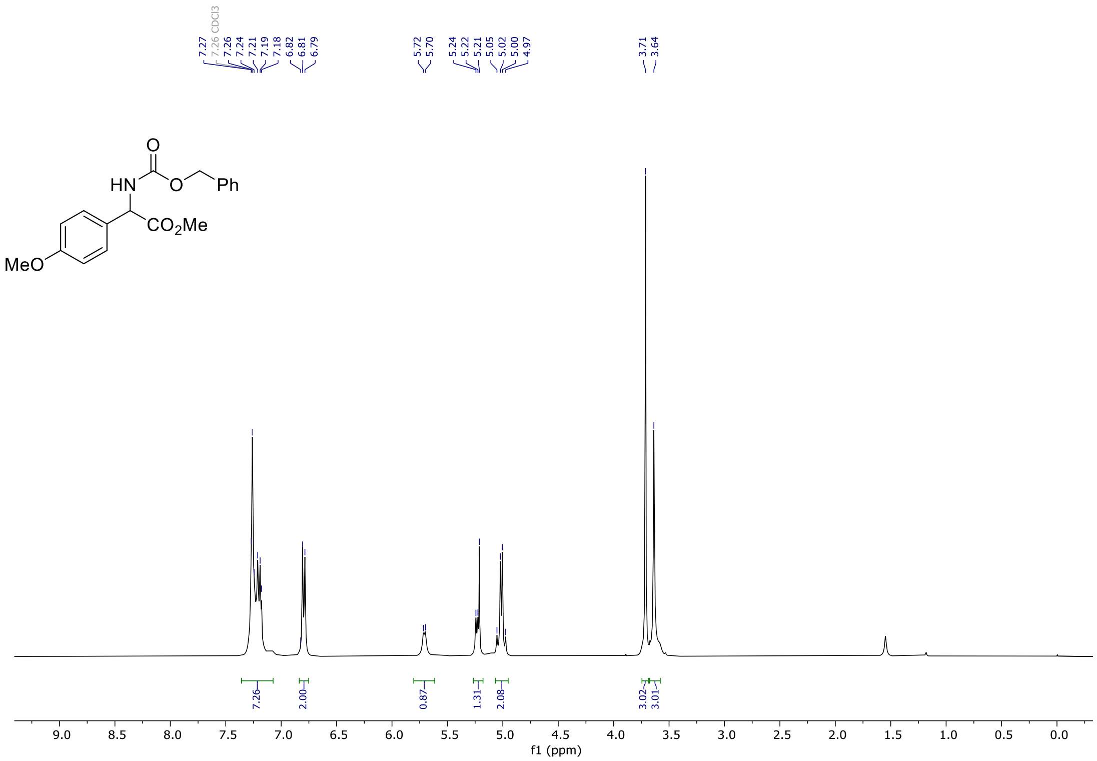

S40: ^{13}C NMR (101 MHz, $CDCl_3$):

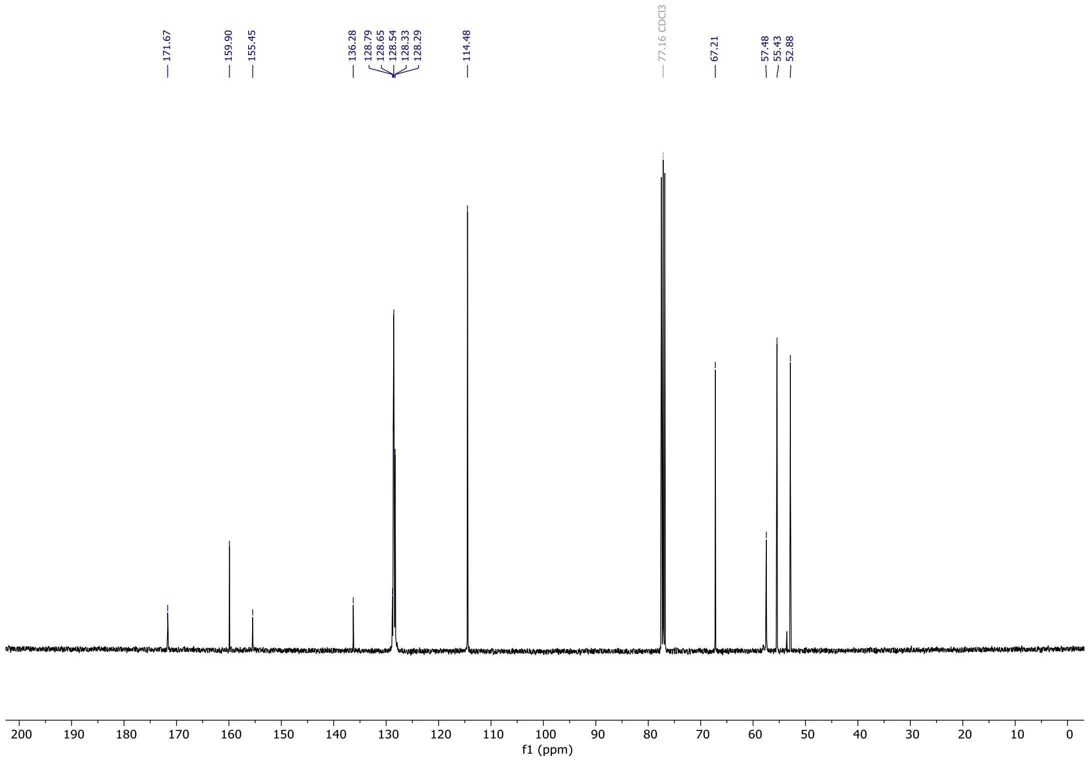

References

[1] Jana, S.; Guo, Y.; Koenigs, R. M. Recent Perspectives on Rearrangement Reactions of Ylides via Carbene Transfer Reactions, *Chem. Eur. J.* **2021**, *27*, 1270-1281.

[2] Ren, Y.-Y.; Zhu, S.-F.; Zhou, Q.-L. Chiral Proton-Transfer Shuttle Catalysis for Carbene Insertion Reactions. *Org. Biomol. Chem*. **2018**, *16*, 3087-3094.

[3] For interesting exceptions, see ref. 4 and the following: Zhang, Z.; Sheng, Z.; Yu, W.; Wu, G.; Zhang, R.; Chu, W.-D.; Zhang, Y.; Wang, J. Catlytic Asymmetric Trifluorothiomethylation via Enantioselective [2,3]-Sigmatropic Rearrangement of Sulfonium Ylides, *Nature Chem*. **2017**, *9*, 970-976.

[4] Li, Z., Boyarskikh, V.; Hansen, J. H.; Autschbach, J.; Musaev, D. G.; Davies, H. M. L., Scope and Mechanistic Analysis of the Enantioselective Synthesis of Allenes by Rhodium-Catalyzed Tandem Ylide Formation/[2,3]-Sigmatropic Rearrangement between Donor/Acceptor Carbenoids and Propargyl Alcohols, *J. Am. Chem. Soc*. **2012**, *134*, 15497-15504.

[5] Liang, Y.; Zhou, H.; Yu, Z.-X., Why is Copper(I) Complex More Competent Than Dirhoidum(II) Complex in Catalytic Asymmetric O–H Insertion Reactions? A Computational Study of the Metal Carbenoid O–H Insertion into Water, *J. Am. Chem. Soc*. **2009**, *131*, 17783-17785.

[6] Huang, M.-Y.; Yang, J.-M.; Zhao, Y.-T.; Zhu, S.-F. Rhodium-Catalyzed Si–H Bond Insertion Reactions Using Functionalized Alkynes as Carbene Precursors, *ACS Catal*. **2019**, *9*, 5353-5357.

[7] Collins, L. R.; Auris, S.; Goddard, R.; Fürstner, A. Chiral Heterobimetallic Bismuth-Rhodium Paddlewheel Catalysts: A Conceptually New Approach to Asymmetric Cyclopropanation. *Angew. Chem. Int. Ed.* **2019**, *58*, 3557-3561.

[8] Reiß, G. J.; Frank, W.; Schneider, J. Synthesis and Crystal Structure of Bismuth(III) Trifluoroacetate Trifluoroacetic Acid Adduct, $Bi(OOCCF_3)_3 \cdot HOOCCF_3$, *Main Group Met. Chem.* **1995**, *18*, 287-294.

[9] Adamek, J.; Mazurkiewicz, R.; Węgrzyk, A.; Erfurt, K. 1-Imidoalkylphosphonium Salts with Modulated C $_{\alpha}$-P $^{+}$ Bond Strength: Synthesis and Application as New Active α-Imidoalkylating Agents. *Beilstein J. Org. Chem.* **2017**, *13,* 1446–1455.

[10] Keipour, H.; Ollevier, T., Iron-Catalyzed Carbene Insertion Reactions of α-Diazoesters into Si–H Bonds. *Org. Lett.* **2017,** *19*, 5736-5739.

[11] Tortoreto, C.; Rackl, D.; Davies, H. M. L. Metal-Free C–H Functionalization of Alkanes by Aryldiazoacetates. *Org. Lett.* **2017**, *19*, 770-773.

[12] Fu, L.; Mighion, J. D.; Voight, E. A.; Davies, H. M. L. Synthesis of 2,2,2,-Trichloroethyl Aryl- and Vinyldiazoacetates by Palladium-Catalyzed Cross-Coupling. *Chem. Eur. J.* **2017**, 23, 3272 – 3275.

[13] Denton, J. R.; Davies, H. M. L. Enantioselective Reactions of Donor/Acceptor Carbenoids Derived from α-Aryl-α-Diazoketones. *Org. Lett.* **2009**, *11*, 787-790.

[14] Lee, M.; Ren, Z.; Musaev, D. G.; Davies, H. M. L., Rhodium-Stabilized Diarylcarbenes Behaving as Donor/Acceptor Carbenes. *ACS Catal.* **2020,** *10*, 6240-6247.

15 Adly, F. G.; Gardiner, M. G.; Ghanem, A., Design and Synthesis of Novel Chiral Dirhodium(Ii) Carboxylate Complexes for Asymmetric Cyclopropanation Reactions. *Chem. Eur. J.* **2016,** *22*, 3447-3461.

16 Jagannathan, J. R.; Fettinger, J. C.; Shaw, J. T.; Franz, A. K. Enantioselective Si–H Insertion Reactions of Diarylcarbenes for the Synthesis of Silicon-Stereogenic Silanes, *J. Am. Chem. Soc.* **2020**, *142*, 11674-11679.

17 Neese, F., Software Update: The Orca Program System, Version 4.0. *WIREs Comput. Mol. Sci.* **2018**, *8*, e1327.

18 Grimme, S.; Hansen, A.; Brandenburg, J. G.; Bannwarth, C., Dispersion-Corrected Mean-Field Electronic Structure Methods. *Chem. Rev.* **2016**, *116*, 5105-5154.

19 Becke, A. D.; Johnson, E. R., A Density-Functional Model of the Dispersion Interaction. *J. Chem. Phys.* **2005**, *123*, 154101.

20 Weigend, F.; Ahlrichs, R., Balanced Basis Sets of Split Valence, Triple Zeta Valence and Quadruple Zeta Valence Quality for H to Rn: Design and Assessment of Accuracy. *Phys. Chem. Chem. Phys.* **2005**, *7*, 3297-3305.

21 Bistoni, G., Finding Chemical Concepts in the Hilbert Space: Coupled Cluster Analyses of Noncovalent Interactions. *WIREs Comput. Mol. Sci.* **2020**, *10*, e1442.

22 Schneider, W. B.; Bistoni, G.; Sparta, M.; Saitow, M.; Riplinger, C.; Auer, A. A.; Neese, F., Decomposition of Intermolecular Interaction Energies within the Local Pair Natural Orbital Coupled Cluster Framework. *J. Chem. Theory Comput.* **2016**, *12*, 4778-4792.
